# The First Functional Traits Dataset for the Endemic Flora of Greece: morphology, ecology and ecosystem services

**DOI:** 10.3897/BDJ.14.e180342

**Published:** 2026-02-10

**Authors:** Alexian Cheminal, Elpida Karadimou, Elisa Aubourg, Ioannis P. Kokkoris, Athanasios Kallimanis, Panayotis Dimopoulos

**Affiliations:** 1 Laboratory of Botany, Department of Biology, University of Patras, 26504, Patras, Greece Laboratory of Botany, Department of Biology, University of Patras, 26504 Patras Greece; 2 L’Institut Agro Dijon (ex-Agrosup Dijon), University of Burgundy, 21000, Dijon, France L’Institut Agro Dijon (ex-Agrosup Dijon), University of Burgundy, 21000 Dijon France; 3 Department of Sustainable Agriculture, University of Patras, 2 G. Seferi St., 30131, Agrinio, Greece Department of Sustainable Agriculture, University of Patras, 2 G. Seferi St., 30131 Agrinio Greece; 4 School of Biology, Aristotle University of Thessaloniki, 54124, Thessaloniki, Greece School of Biology, Aristotle University of Thessaloniki, 54124 Thessaloniki Greece

**Keywords:** Greek flora, endemic taxa, functional traits, dataset, ecosystem services, Greece

## Abstract

**Background:**

Endemic plant taxa, which are often understudied due to their restricted populations and limited distribution, are a key focus in Greece, which is a major hotspot for plant diversity and endemism within the Mediterranean biodiversity hotspot region. This work contributes to national efforts to enhance understanding of Greek endemic diversity by: (a) summarizing existing knowledge on karyology, morphology, ecology, and plant uses through a comprehensive literature review, and (b) providing a complete data bank of selected functional traits for the 1,636 understudied endemic taxa of the Greek flora.

**New information:**

This dataset is the first comprehensive compilation of its kind for Greek endemic flora. It includes values for eight functional traits, two ecological characteristics, and one genetic characteristic for all 1,636 endemic taxa, alongside data on the presence or absence of ecosystem services across six categories. The dataset enables further study of Greek endemic flora at various scales, offering insights into the characteristics of these habitat-specialized, unique taxa. It will be used by our team to analyze distribution patterns and relationships between functional traits and habitats, particularly in the context of climate change.

## Introduction

Greece is widely acknowledged for the richness of its flora. As part of the Mediterranean Basin plant diversity hotspot, it is home to 5,987 species, and 2,011 subspecies (native and naturalized), representing 6,867 taxa (Dimopoulos et al. 2013, Dimopoulos et al. 2016, Dimopoulos et al. 2024). The topographical complexity of this Mediterranean country has led to a high endemicity level with 1,636 endemic taxa recorded, about 20% of the Greek native plant taxa (Georghiou and Delipetrou 2010, Kougioumoutzis et al. 2021b).

Today, we recognize that unique taxa like endemics—specifically adapted to particular regions—are adversely affected by climate change to a greater extent (Vincent et al. 2020, Dirnböck et al. 2011, Manes et al. 2021), and are highly vulnerable to anthropogenic land use changes leading to habitat loss, fragmentation and degradation (Lavergne et al. 2005, Crooks et al. 2017, Hu et al. 2020). Recent findings have sparked significant scientific interest in studying Greek endemic flora's characteristics and vulnerability, both nationally (Georghiou and Delipetrou 2010, Kougioumoutzis et al. 2021a, Kougioumoutzis et al. 2025, Liveri et al. 2024) and regionally (Kagiampaki et al. 2011, Lazarina et al. 2019, Panitsa et al. 2021).

The concept of functional traits has emerged to describe how species respond to their environment and disturbances (response traits) through morphology, physiology, phenology, ecology, or behavior, and to quantify their impact on ecosystems (effect traits) (Violle et al. 2007, Nock et al. 2016, Levine 2015). In plant ecology, it serves as a proxy to assess correlations between environmental changes (e.g., altitude, ecological succession, pollution, climate change) and plant taxa responses, evaluating their adaptation to conditions and changes (Díaz et al. 2016, Wang et al. 2023), and their influence on ecosystem processes and services (de Bello et al. 2010, Lavorel 2012, Carlucci et al. 2020). As interest in functional traits grows (Tavşanoğlu and Pausas 2018), the study of those of rare and understudied plants should be prioritized due to their unique roles in ecosystems and benefits to humanity (Mouillot et al. 2013, Jain et al. 2013).

In Greece, functional traits are becoming increasingly important for studying plant diversity (Raus et al. 2019, Mastrogianni et al. 2024), with growing interest in the functional traits of endemic flora as they link species-level biodiversity to ecosystem processes (Mermygkas et al. 2021, Cheminal et al. 2022). Endemics significantly enhance potential functions and ecosystem services across scales (Mouillot et al. 2013), offering resilience against climate change and anthropogenic pressures. However, data on the functional traits of Greek endemics remain limited, with a comprehensive nationwide dataset still needed.

To address this knowledge gap, we compiled a dataset on the traits of Greek endemic flora by combining diverse literature sources. It includes (a) eleven genetic, ecological, and morphological functional traits, and (b) presence/absence of ecosystem services across six use categories. This dataset is a valuable tool for exploring ecological patterns, with functional trait analysis of endemics offering insights into: (a) interspecific interactions among endemic and non-endemic species and their environmental relationships, and (b) ecosystem responses to future changes or disturbances, such as climate change or significant land-use shifts.

## General description

### Purpose

To provide a standardized, literature-derived dataset describing functional traits, genetic and ecological characteristics, and documented ecosystem services (ES) for all 1,636 endemic vascular plant taxa of Greece, facilitating reuse in ecology, conservation and biogeography.

### Additional information

The dataset comprises eight functional traits, two ecological characteristics and one genetic characteristic across 41 subtypes, alongside six ES categories.

## Project description

### Study area description

Greece, including mainland and islands as defined in the Vascular Plants of Greece checklist (Dimopoulos et al. 2024), spanning high environmental heterogeneity from sea level to alpine zones.

### Design description

Systematic extraction and standardization of trait and ecosystem-service information from floras, monographs, species descriptions and reviews, followed by harmonization into a machine-readable tabular dataset. The study covers all 1,636 endemic taxa (species and subspecies) recognized for Greece (Dimopoulos et al. 2024), comprising 66 families and 280 genera.

## Sampling methods

### Study extent

The dataset on the functional traits Greek endemic flora was compiled from various literature sources, including floras, atlases, articles on species new to science, and genus review articles. A complete bibliography is provided in Suppl. material 1.

### Sampling description

**Description of functional traits data collection** : The review process followed these steps:

1. Consultation of floras and atlases for trait attributes, including Atlas of the Hellenic Flora (Strid 2024a, Strid 2024b, Strid 2024c), Flora Europaea (Tutin et al. 1993, Tutin et al. 1968, Tutin et al. 1972, Tutin et al. 1976, Tutin et al. 1980), Flora Hellenica (Strid and Tan 1997, Strid and Tan 2002), Mountain Flora of Greece (Strid 1986, Strid and Tan 1991), Shrubs and Trees of Greece (Arambatzis 1998, Arambatzis 2001), Vascular Plants of Greece (Dimopoulos et al. 2013, Dimopoulos et al. 2016, Dimopoulos et al. 2024), and the FloraVeg database (Chytrý et al. 2024).
2. When traits were missing, Plants of the World Online (POWO 2025) was used to check for synonymy (e.g., basionym, taxonomic changes) and to identify initial or complementary publications. Research engines (Google Scholar) and the Library of the Laboratory of Botany at the University of Patras were searched for relevant references, extracting trait attributes when available.

Literature sources used for both steps are presented in Suppl. material 1.

**Ecosystem Services (ES) Data Sampling**: Data on six categories of ES (see Ecosystem services coverage) provided by Greek endemics were collected using search engines (Google Scholar, Science Direct) with keyword combinations [“plant name” + “medicinal”, “medical”, “aromatic”, “properties”, “uses”, or “activity”], following the methodology of Cheminal et al. (2022). As with previous traits, this availability of this data relies on the existing literature. The current absence of an ES for a taxon does not necessarily mean that it does not provide any ES in its natural habitat, but that the taxon has yet to be documented for its services. Literature sources on ES provision by Greek endemic taxa are presented in Suppl. material 2.

### Quality control

Most of the functional trait data were sourced from the original taxon descriptions or expert revisions. When functional trait data was unavailable, two approximation methods were used: (a) traits of a related taxon, as described in floras, were applied to the endemic taxon; (b) flowering periods or altitudinal ranges were estimated from the sampling periods and sites of the type material, if these were not specified. Unavailable or inapproximable traits were left null: approximately 11.0% of the dataset remains incomplete, with the percentage of missing data varying between traits. The link to the dataset is provided in Data resources.

## Geographic coverage

### Description

The dataset focuses on the endemic flora of Greece (mainland and islands), as described by Dimopoulos et al. 2024). As part of the Mediterranean biodiversity hotspot, Greece hosts 6,867 vascular plant taxa, 1,636 of which are endemic, including 1,077 taxa unique to a single Greek floristic region. The majority of these endemic taxa are found in Southern Greece, particularly in the Peloponnese (510 taxa, including 203 regional endemics) and Crete-Karpathos (429 taxa, 283 regional endemics): the two regions account for 57.4% of Greek endemics. The dataset includes the distribution of each taxon among Greek floristic regions (x: presence, ?: doubtful presence).

### Coordinates

34.8° and 41.8° Latitude; 28.3° and 19.4° Longitude.

## Taxonomic coverage

### Description

The dataset includes 1,636 endemic taxa (species and subspecies), spanning 66 families and 280 genera. The dominant families are Asteraceae (23% of the endemic taxa), Caryophyllaceae (11%), Lamiaceae (7%), and Brassicaceae (6%). Key genera with high endemism are *Hieracium* (6.5% of the endemic taxa), *Centaurea* (5.2%), *Limonium* (5%) and *Allium* (4.4%) (Dimopoulos et al. 2024).

## Traits coverage

### Plant traits: Description and ecological meaning

**Description.** The dataset includes eight functional (morphology, phenology), two ecological traits (topography, environment), and one genetic trait (karyology), across 41 trait subtypes (see Table 1). These traits were chosen according to their importance for plant metabolism and ecology, as documented in literature (see Suppl. material 3).

### Traits summary statistics

**Figure 1.**
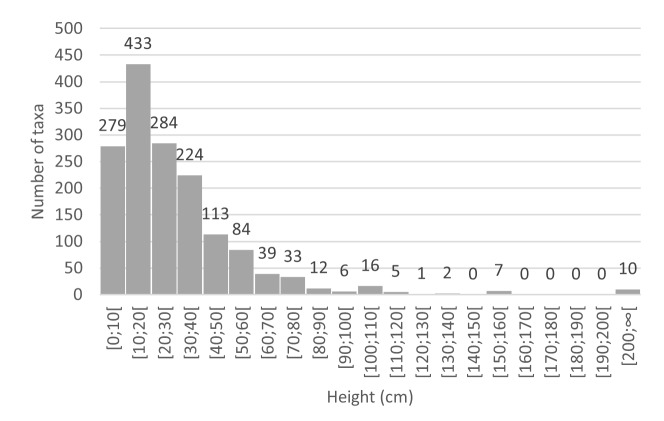
Distribution of Plant Height (H) values for the endemic taxa of Greece (average values without extremes).

**Adult plant height (H)**: Values of H were recorded for 1,548 taxa (94.62% of the 1,636 endemic taxa). Average H is 31.33cm (Standard Deviation (SD) = 91.32cm), or 32.96cm (SD=102.47cm) including extreme measurements (*IEM*). H varies from 0.95cm to 3,000.00cm (Fig. 1).

**Figure 2.**
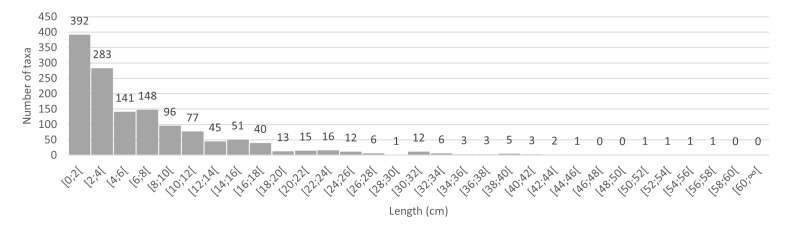
Distribution of Leaf Length (LL) values for the endemic taxa of Greece (average values without extremes).

**Leaf length (LL)**: Values of LL were recorded for 1,375 taxa (84.05%). Average LL is 6.77cm (SD = 7.67cm), or 6.89cm *IEM* (SD=7.79cm). LL varies from 0cm (no leaf) to 57.50cm (62.50cm *IEM*) (Fig. 2).

**Figure 3.**
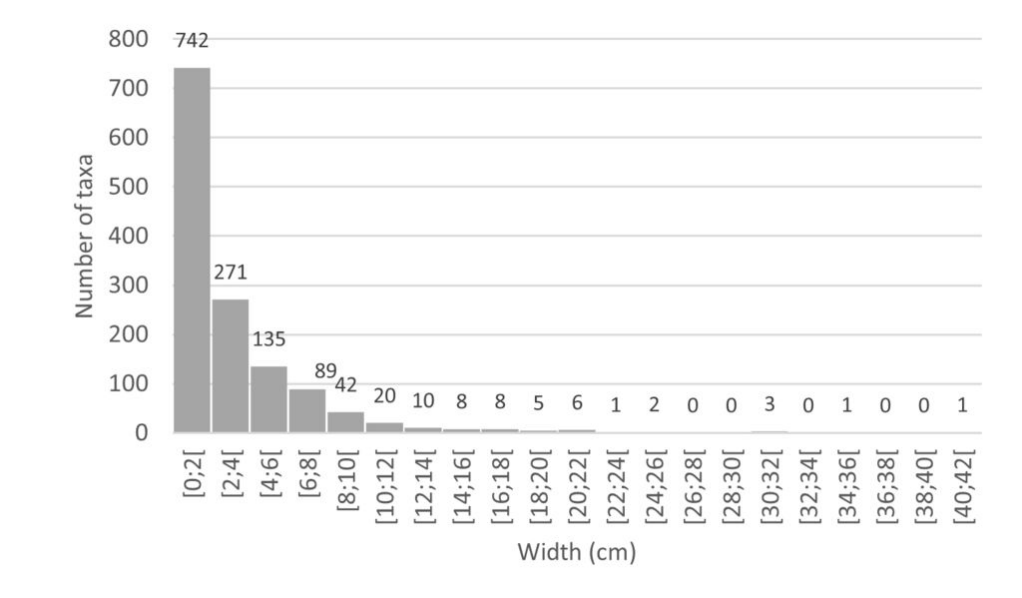
Distribution of Leaf Width (LW) values for the endemic taxa of Greece (average values without extremes).

**Leaf width (LW)**: Values of LW were recorded for 1,344 taxa (82.15%). Average LW is 1.45cm (SD = 1.97cm), or 1.47cm *IEM* (SD=2.02cm). LW varies from 0cm (no leaf) to 22.75cm (25.00cm *IEM*) (Fig. 3).

**Figure 4.**
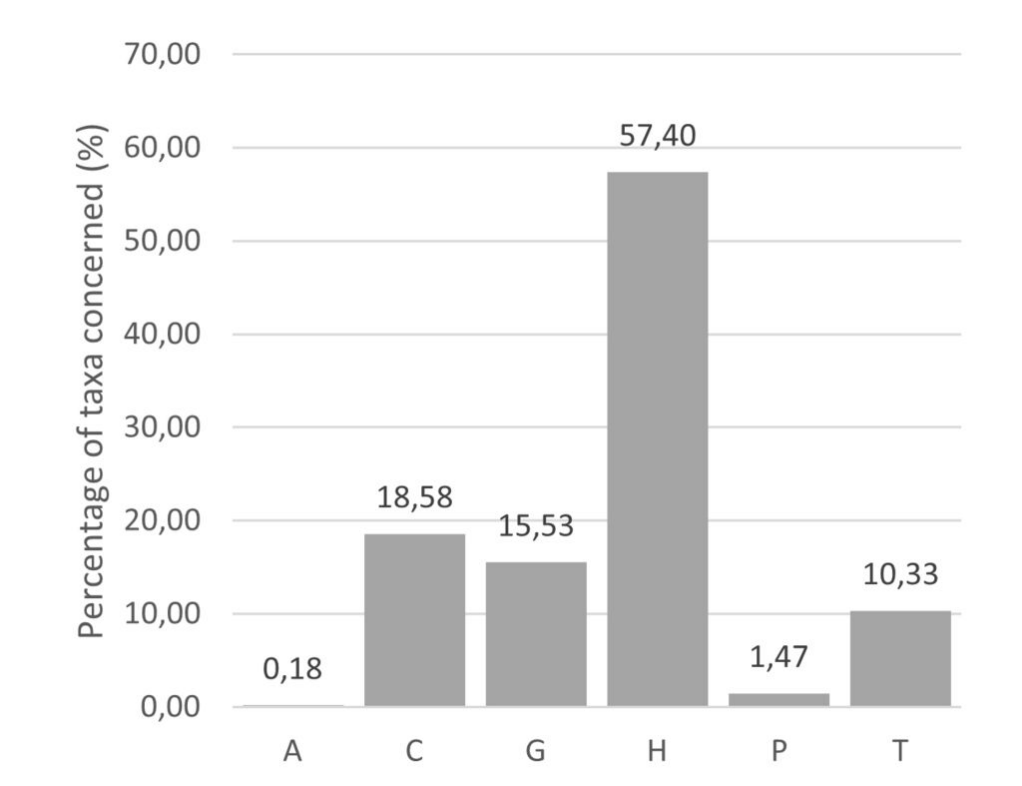
Distribution of Life Form (LF) values for the endemic taxa of Greece (A: Aquatic, C: Chamaephyte, G: Geophyte, H: Hemicryptophyte, P: Phanerophyte, T: Therophyte).

**Life-form (LF)**: Values of LF were recorded for all 1,636 taxa (100%). Taxa have been associated with either one or two LF (average of 1.03 life-forms per taxon). The most frequently recorded LF were hemicryptophytes (57.40%), chamaephytes (18.58%) and geophytes (15.53%) (Fig. 4).

**Figure 5.**
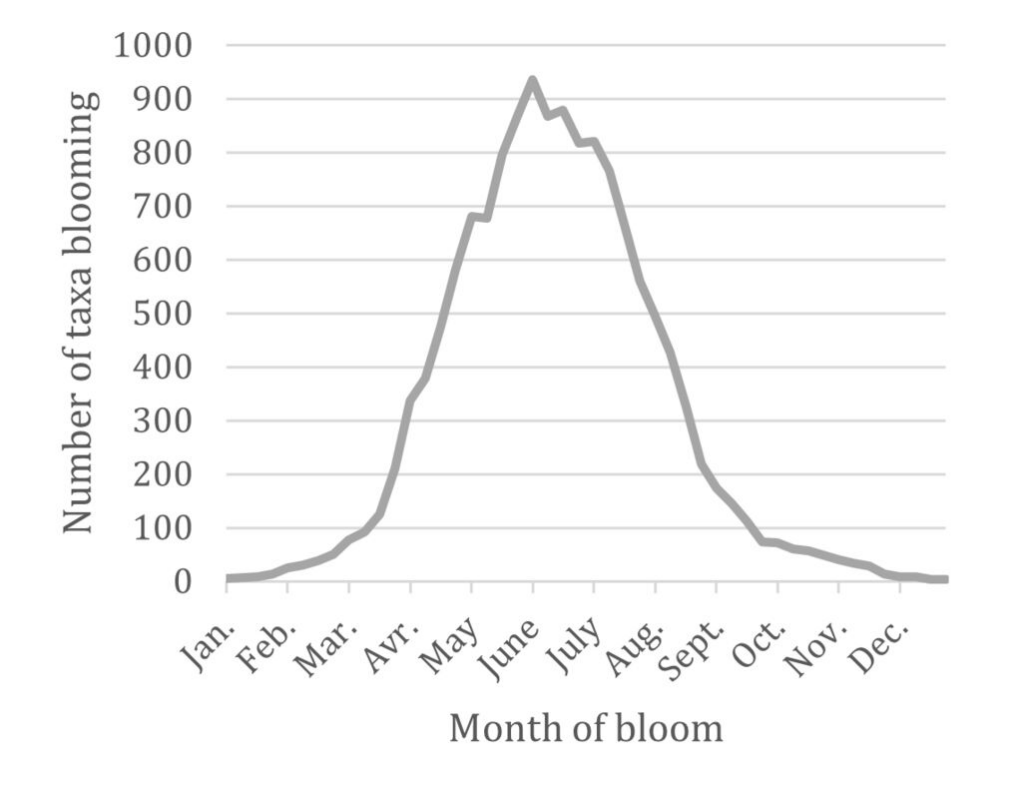
Distribution of Flowering Periods (FP) for the endemic taxa of Greece: number of taxa in their blooming period per time increment (early, mid, late or full month).

**Flowering period (FP)**: A main FP was recorded for 1,615 taxa (98.72%). The second FP was recorded for five taxa (0.31%). Main FP were on average reaching their peak in early June (SD=1.45 months), with an average duration of two months (SD=2.05 months), from early May to early July. The earliest flowering peak was recorded for mid-January and the latest for mid-November. Second FP peak is observed in early October (SD=0.40 months) and lasts an average of 1.44 months (SD= 0.38 months), from mid-September to late October (Fig. 5).

**Figure 6.**
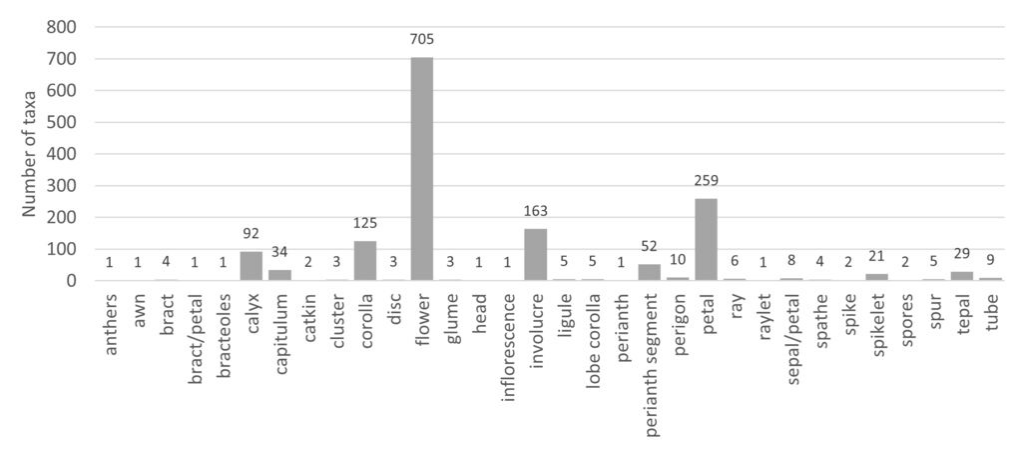
Plant parts measured for Flower Size (FS) values for the endemic taxa of Greece

**Figure 7.**
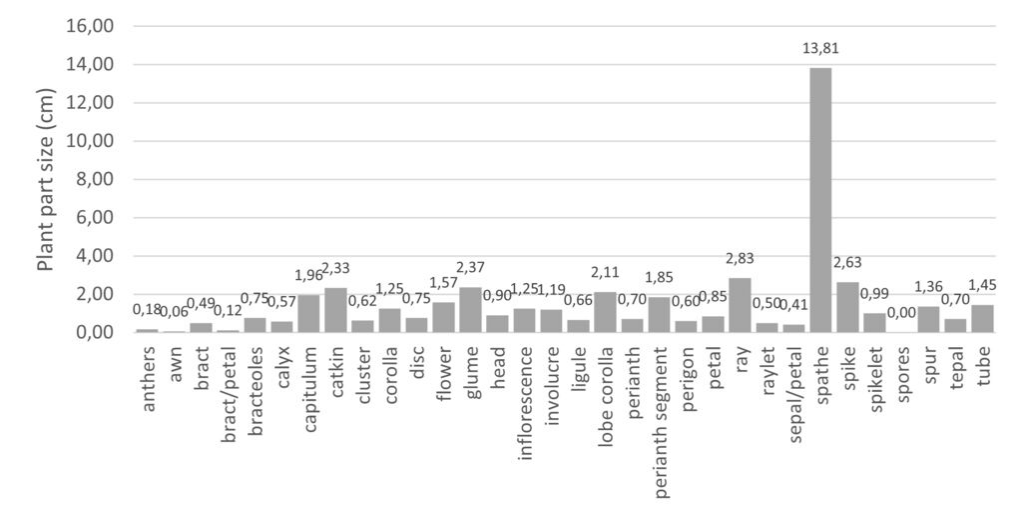
Average measurement of the plant parts described for Flower Size (FS) values, for the endemic taxa of Greece.

**Flower size (FS)**: Recorded for 1,559 taxa (95.29%). Due to the diversity of flower morphologies (e.g., simple/compound, sympetalous/separate petals, etc.), FS was characterized by the measured reproductive organ. Of the 32 identified plant parts, the most common were petals (15.83% of the 1,636 taxa), involucres (9.96%), and corollas (7.64%), while 43.1% of records described the general reproductive structure (“flower”). Measurements included diameter (30.44%), length (20.48%), or unspecified dimensions (49.08%). FS values ranged from 0 (no flower) to 22.50 cm (longest spathe). Average sizes were 0.85cm for petals, 1.19cm for involucres, 1.25cm for corollas, and 1.57cm for “flowers”, with organ sizes ranging from 0.06 cm (awns, one taxon) to 13.80cm (spathes, four taxa) (Figs 6, 7).

**Figure 8.**
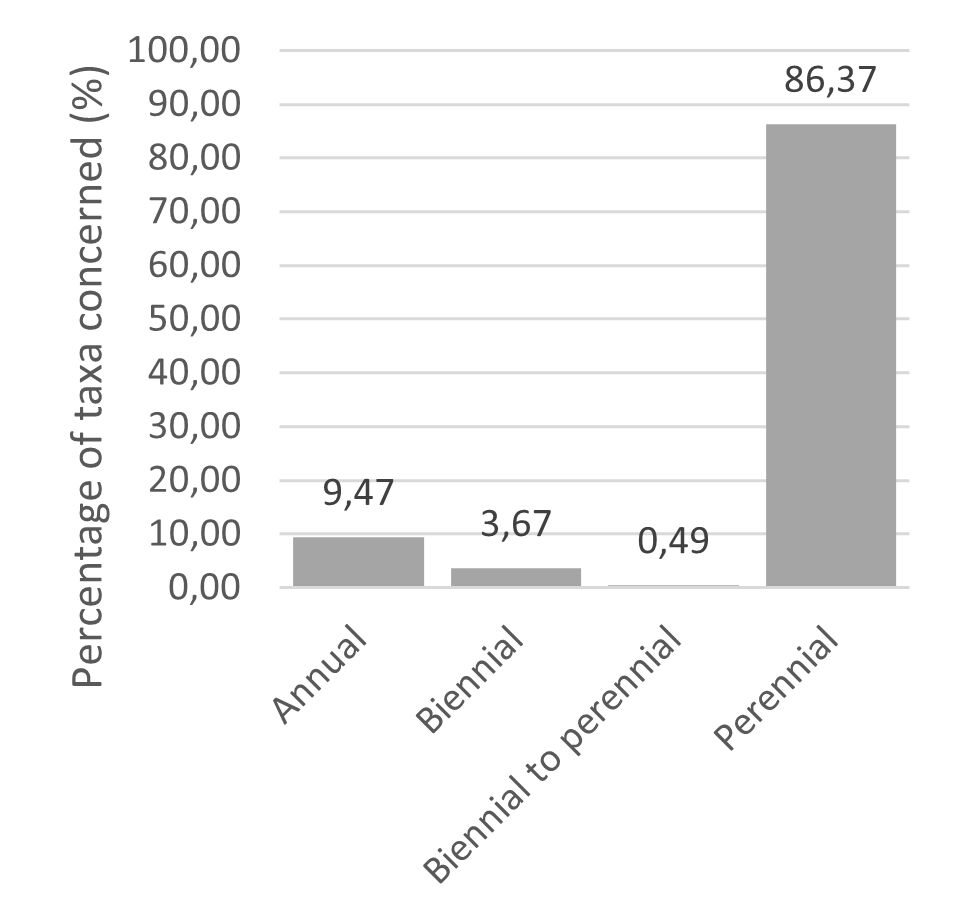
Distribution of Longevity (L) values for the endemic taxa of Greece.

**Longevity (L)**: Recorded for all taxa (100%). Most taxa are perennial (86.37%), over annual (9.47%) (Fig. 8).

**Figure 9.**
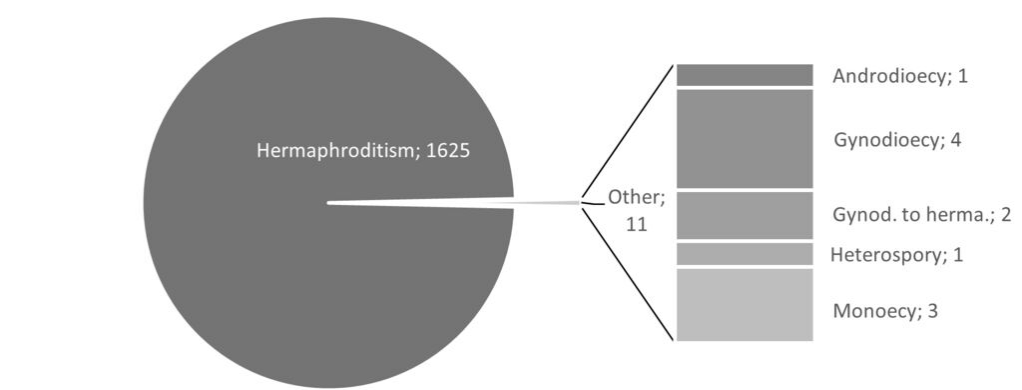
Distribution of Reproduction Strategy (RS) values for the endemic taxa of Greece.

**Reproduction strategy (RS)**: Recorded for all taxa (100%). The majority are hermaphrodite (99.33%), all other strategies minor (0.67%) (Fig. 9).

**Figure 10.**
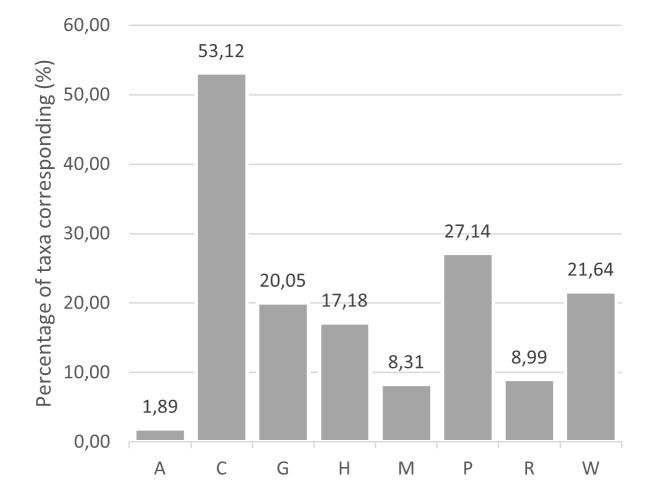
Distribution of the endemic taxa of Greece among Habitats (Ha) (A: Aquatic, C: Cliffs and rocks, G: Grasslands, H: High mountains, M: Maritime and coastal, P: Phrygana, R: Ruderal, W: Woods and scrubs).

**Habitat (Ha)**: Native habitats were recorded for 1,619 taxa (98.96%), with up to four habitats per taxon (average of 1.60 habitats per taxon, SD=0.58). The most common habitats were cliffs and rocky habitats (53.12%) and phrygana (27.14%), while the least common were freshwater (aquatic) habitats (1.89%) (Fig. 10).

**Figure 11.**
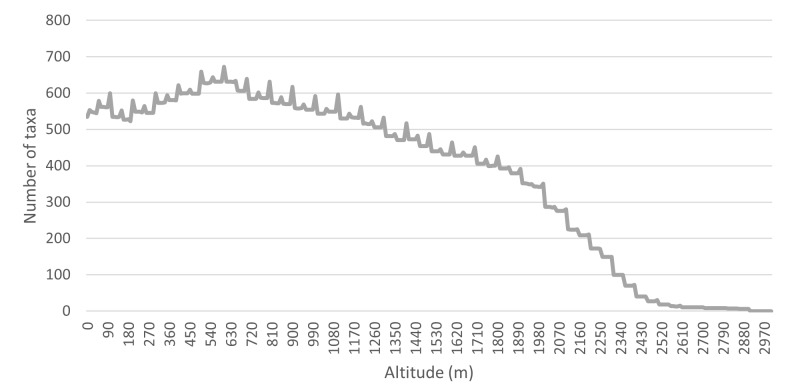
Number of endemic taxa of Greece per Altitude (A)

**Altitude (A)**: Altitudinal range was recorded for 1,607 taxa (98.23%). On average, taxa occur from 562 m (SD=614 m) to 1,260 m (SD=732 m), with a mean altitude of 906 m (SD=631 m). Individual ranges vary from 1 m (coastal taxa) to 2,750 m (high mountain endemics) (Fig. 11).

**Figure 12.**
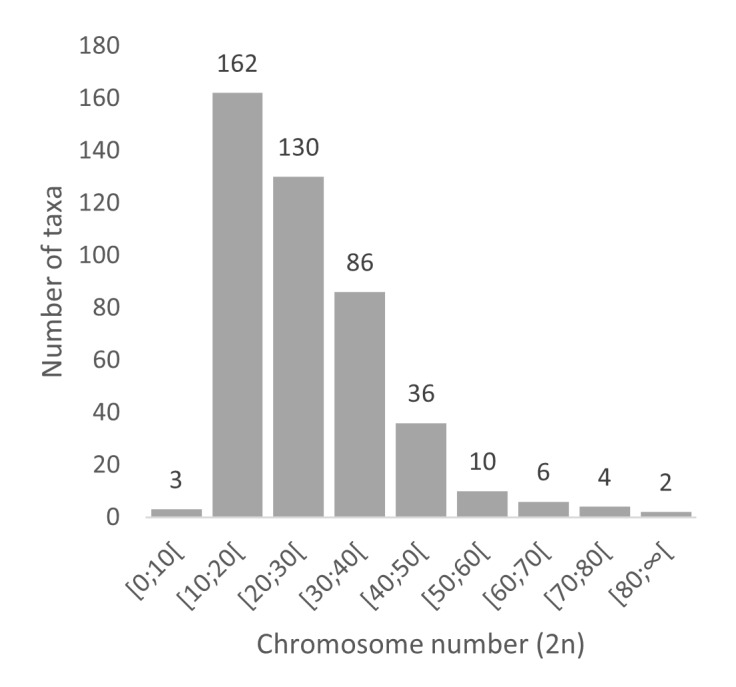
Distribution of Chromosome number (2n) values for the endemic taxa of Greece

**Chromosome number (2n)**: Recorded for 439 taxa (26.83%). The average chromosome number is 2n=27 (SD=15), ranging from 2n=6 (*Hypochaeris tenuiflora*) to 2n=150 (*Prospero talosii*) (Fig. 12).

### Ecosystem services (ES) coverage

**Figure 13.**
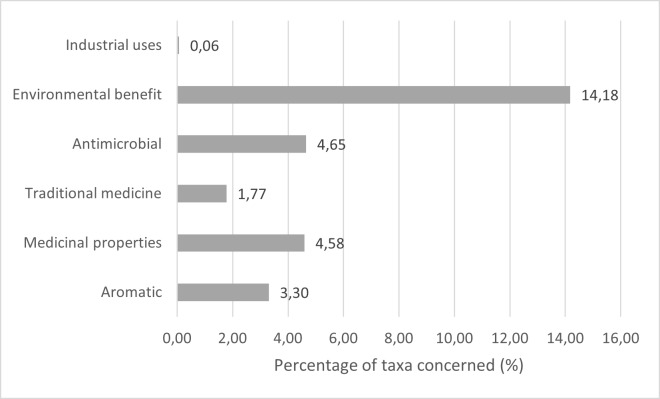
Ecosystem services (ES) documented for the endemic flora of Greece.

**Description**: The dataset includes six categories of ES (Table 2). From the 1,636 endemic taxa, 324 taxa (19.80%) were documented as providing ES. The most common are environmental benefits (e.g., ornamental, bioaccumulator, etc.) (14.18%), followed by antimicrobial properties (4.65%) and other medicinal properties (4.58%) (Fig. 13).

## Temporal coverage

### Notes

The dataset was built in three phases: (a) a preliminary dataset on Peloponnese endemics by Elpida Karadimou, (b) its completion by Elisa Aubourg in 2021, and (c) its update and expansion to all Greek endemics by Alexian Cheminal in 2024-2025. Literature sources for functional traits span from 1753 to 2025 (see Suppl. material 1).

## Usage licence

### Usage licence

Other

### IP rights notes

Usage licence: Creative Commons Attribution 4.0 International (CC-By)

All data can be freely used with attribution. Please cite this data paper and the resource when reusing the dataset.

## Data resources

### Data package title

The First Functional Traits Dataset for the Endemic Flora of Greece: morphology, ecology and ecosystem services.

### Resource link

https://zenodo.org/records/17707927

### Alternative identifiers

https://doi.org/10.5281/zenodo.17707927

### Number of data sets

1

### Data set 1.

#### Data set name

Functional traits Greek endemic plant taxa- Cheminal et al.

#### Description

Attributes for distribution, for eight functional traits, two ecological characteristics, one genetic characteristic and six ecosystem service categories for the 1,636 endemic taxa of Greece.

**Data set 1.** 

Column label	Column description
Family	Full scientific name of the taxon’s family
Genus	Full scientific name of the taxon’s genus
Species	Full scientific name of the taxon’s species
Subspecies	Full scientific name of the taxon’s subspecies (facultative)
IoI	Presence of the taxon in the floristic region of Ionian Islands
NPi	Presence in Northern Pindos
SPi	Presence in Southern Pindos
Pe	Presence in Peloponnese
StE	Presence in Sterea Ellas
EC	Presence in East-Central Greece
NC	Presence in North-Central Greece
NE	Presence in North-Eastern Greece
NAe	Presence on Northern Aegean islands
WAe	Presence on Western Aegean islands
KiK	Presence on Cyclades Islands
KK	Presence on Crete/Karpathos islands
EAe	Presence on Eastern Aegean islands
Chrmsm_min	Smallest chromosome number documented in the literature for the taxon (2n), if several
Chrmsm_mode	Main or only chromosome number documented (2n)
Chrmsm_max	Highest chromosome number documented (2n), if several
Plant_height_extr_min_cm	Lowest observed minimal plant height for the taxon in its description.(e.g., (*2*-)3-10(-16) cm)
Plant_height_aver_min_cm	Average observed minimal plant height.(e.g., (2-)*3*-10(-16) cm)
Plant_height_aver_max_cm	Average observed maximal plant height.(e.g., (2-)3-*10* (-16) cm)
Plant_height_extr_max_cm	Highest observed maximal plant height.(e.g., (2-)3-10(-*16*) cm)
Leaf_length_extr_min_cm	Lowest observed minimal leaf length for the taxon in its description.
Leaf_length_aver_min_cm	Average observed minimal leaf length.
Leaf_length_aver_max_cm	Average observed maximal leaf length.
Leaf_length_extr_max_cm	Highest observed maximal leaf length.
Leaf_width_extr_min_cm	Lowest observed minimal leaf width.
Leaf_width_aver_min_cm	Average observed minimal leaf width.
Leaf_width_aver_max_cm	Average observed maximal leaf width.
Leaf_width_extr_max_cm	Highest observed maximal leaf width.
Life_form_1	Life form observed for the taxon (A: Aquatic, C: Chamaephyte, G: Geophyte, H: Hemicryptophyte, P: Phanerophyte, T: Therophyte).
Life_form_2	Second life form observed for the taxon. (facultative)
Flw_per_extr_min_month	Earliest month/period recorded for the beginning of the main bloom of the taxon.
Flw_per_aver_min_month	Average period for the beginning of the main bloom.
Flw_per_aver_max_month	Average period for the end of the main bloom.
Flw_per_extr_max_month	Latest period for the end of the main bloom.
SECOND_Flw_per_extr_min_month	Earliest period for the beginning of the second bloom of the taxon. (facultative)
SECOND_Flw_per_aver_min_month	Average period for the beginning of the second bloom. (facultative)
SECOND_Flw_per_aver_max_month	Average period for the end of the second bloom. (facultative)
SECOND_Flw_per_extr_max_month	Latest period for the end of the second bloom. (facultative)
Flw_size_extr_min_cm	Lowest observed minimal flower/inflorescence’s dimension for the taxon in its description.
Flw_size_aver_min_cm	Average observed minimal flower’s dimension.
Flw_size_aver_max_cm	Average observed maximal flower’s dimension.
Flw_size_extr_max_cm	Highest observed maximal flower’s dimension.
Flw_size_dimension	Measurement (diam./length) significative for the description of the taxon’s flower/inflorescence.
Flw_size_organ	Floral organ measured for the description of the taxon.
Longevity	Longevity of the taxon in its natural habitat.
Flower_sex	Reproduction strategy applied by the taxon (*e.g.*, hermaphrodite, etc.).
Habitat_1	Habitat type suitable for the taxon (A: Aquatic, C: Cliffs and rocks, G: Grasslands, H: High mountains, M: Maritime and coastal, P: Phrygana, R: Ruderal, W: Woods and scrubs).
Habitat_2	Second habitat suitable for the taxon. (facultative)
Habitat_3	Third habitat suitable for the taxon. (facultative)
Habitat_4	Fourth habitat suitable for the taxon. (facultative)
Altitude_extr_min (m)	Lowest minimal elevation to which the taxon has been observed.
Altitude_aver_min (m)	Average minimal elevation.
Altitude_aver_max (m)	Average maximal elevation.
Altitude_extr_max (m)	Highest maximal elevation.
Aromatic	Documented provision of ecosystem services (ES) relevant to food, spices, aromas, perfumes, etc.
Medicinal_properties	Documented provision of ES relevant to modern medicine.
Traditional_medicine	Documented provision of ES as part of folk medicine.
Antimicrobial	Documented provision of ES: efficient against microorganisms/parasites.
Environmental_benefit	Documented provision of ES for environment conservation/enhancement.
Industrial_use	Documented provision of ES: applications in craftmanship or industry.

## Supplementary Material

3576B0B6-F032-5B01-AC98-3E030BDCE218 10.3897/BDJ.14.e180342.suppl1 0 Supplementary material 1 Functional traits of the Greek endemic taxa – Bibliography per family Data type Bibliography Brief description This supplementary material lists the literature relevant to the review of the functional, ecological and genetic traits of the endemic taxa of Greece. File: oo_1457320.pdf https://binary.pensoft.net/file/1457320 Alexian Cheminal, Elpida Karadimou, Elisa Aubourg, Ioannis P. Kokkoris, Athanasios Kallimanis, Panayotis Dimopoulos

0F42FF58-2104-5972-96D7-9763A1D19517 10.3897/BDJ.14.e180342.suppl2 0 Supplementary material 2 Ecosystem services of the Greek endemic taxa – Bibliography per family and genus Data type Bibliography Brief description This supplementary material lists the literature relevant to the review of the provision of ecosystem services by the endemic taxa of Greece. File: oo_1457323.pdf https://binary.pensoft.net/file/1457323 Alexian Cheminal, Elpida Karadimou, Elisa Aubourg, Ioannis P. Kokkoris, Athanasios Kallimanis, Panayotis Dimopoulos

75A759B5-EE0E-5559-9D59-18FE02F2318F 10.3897/BDJ.14.e180342.suppl3 0 Supplementary material 3 Bibliography Traits & Ecology Data type Bibliography Brief description This supplementary material lists resources on the impact of functional traits on plants’ metabolism and ecology. File: oo_1465752.pdf https://binary.pensoft.net/file/1465752 Alexian Cheminal, Elpida Karadimou, Elisa Aubourg, Ioannis P. Kokkoris, Athanasios Kallimanis, Panayotis Dimopoulos

## Figures and Tables

**Table 1.** The 11 functional, ecological and genetic traits values collected for the endemic plant taxa of Greece and their description.

**Trait and characteristics types**	**Functional traits and characteristics**	**Trait sub-type**	**Description**	**Range / Unit**	**Ecological function**
Morphological functional trait	**Plant height (H)**	Plant_height_extr_min Plant_height_aver_min Plant_height_aver_max Plant_height_extr_max	Standard height of the plant, from floor level to highest vegetative or flowering part. The four columns correspond to the four values of average and extreme ranges (*e.g.*, (2-)3-10(-16) cm).	Continuous values (cm)	Determines light capture, competition, dispersal potential, and habitat dominance.
Morphological functional trait	**Leaf length (LL)**	Leaf_length_extr_min Leaf_length_aver_min Leaf_length_aver_max Leaf_length_extr_max	Length of the full limb (for both simple and composed leaves). The columns correspond to the interval (*e.g.*, (1.5-)1.7-3.0(-3.5) cm).	Continuous values (cm)	Relates to photosynthetic capacity, water use efficiency, and stress tolerance.
Morphological functional trait	**Leaf width (LW)**	Leaf_width_extr_min Leaf_ width_aver_min Leaf_ widt _aver_max Leaf_ width_extr_max	Width of the full limb (for both simple and composed leaves), at its widest point. The columns correspond to the interval (*e.g.*, (1.3-) 1.7-3.0(-3.1) cm).	Continuous values (cm)	Indicates resource acquisition strategy (broad leaves: high photosynthesis, narrow leaves: drought/cold adaptation).
Morphological functional trait	**Life form (LF)**	Life_form_1 Life_form_2	Type of life form the taxon can correspond to. As some taxa can vary in life forms, two columns are included.	Discreet values (6): A: Aquatic; C: Chamaephyte; G: Geophyte; H: Hemicryptophyte; P: Phanerophyte; T: Therophyte	Determines survival strategy, persistence, and regeneration under disturbance (e.g., therophytes = fast colonizers, geophytes = resprouters).
Phenological functional trait	**Flowering period (FP)**	Flw_per_extr_min Flw_per_aver_min Flw_per_aver_max Flw_per_extr_max SECOND_Flw_per_extr_min SECOND_Flw_per_aver_min SECOND_Flw_per_aver_max SECOND_Flw_per_extr_max	Season of bloom described as one or two intervals (in case of a second flowering period). Each group of four columns corresponds to the time range of the flowering period (*e.g.*, (5.5-)6-8(-9)) .	Discreet values (48), corresponding to months from January (1) to December (12), with subdivisions for early (.25), mid (.5) or late (.75) start/end of bloom within the month (when known). *Ex*: Early February coded as 2.25, Late September as 9.75.	Influences phenology, pollinator interactions, reproductive success, and temporal niche partitioning.
Morphological functional trait	**Flower size (FS)**	Flw_size_extr_min Flw_size_aver_min Flw_size_aver_max Flw_size_extr_max Flw_size_dimension Flw_size_organ	Dimension of the flower (or inflorescence) at its most characteristic feature. (a) The four first columns correspond to the interval of measurement values (*e.g.*, (0.75-)1-2.5(-3.5) cm). (b) The ‘dimension’ column describes the measurement type (width or length of the organ). (c) The ‘organ’ column precises the part of the flower/inflorescence that was measured in the description of the taxon.	(a) Continuous values (cm) (b) Discreet values (3 dimensions: diameter (diam); length; not_precised) (c) Discreet values (32 organs: anthers; awn; bract; bract/petal; bracteoles; calyx; capitulum; catkin; cluster; corolla; disc; flower; glume; head; inflorescence; involucre; ligule; lobe corolla; perianth; perianth segment; perigon; petal; ray; raylet; sepal/petal; spathe; spike; spikelet; spores; spur; tepal; tube)	Governs pollination mode, attractiveness to pollinators, dispersal potential, and reproductive output.
Phenological functional trait	**Longevity (L)**	Longevity	Longevity of the taxon in its natural habitat.	Discreet values (4): perennial; biennial; biennial to perennial; annual.	Shapes life-history strategy, population turnover, and resilience.
Morphological functional trait	**Reproduction strategy (RS)**	Flower_sex	Reproduction strategy applied by the taxon.	Discreet values (5): hermaphrodite; monoecious; gynoecious; androdioecious; heterospory.	Determines mating system, gene flow, genetic diversity, and population persistence.
Ecological characteristic	**Habitat (Ha)**	Habitat_1 Habitat_2 Habitat_3 Habitat_4	Habitat types the taxon can be found in. As some taxa can be adapted to various habitats, four columns are included.	Discreet values (8): A: Aquatic; C: Cliffs, ravines and rocks; G: Grasslands; H: High mountains; M: Marine and coastal ecosystems; P: Phrygana; R: Ruderal and agricultural; W: Woods and scrubs	Defines ecological niche, stress tolerance, and role in community assembly.
Ecological characteristic	**Altitude (A)**	Altitude_extr_min Altitude_aver_min Altitude_aver_max Altitude_extr_max	Elevation range within which the taxon can be found. Each column corresponds to a parameter of the interval (*e.g.*, (750-)1600-2500(-2800) m).	Continuous values (m)	Relates to climatic tolerance, stress adaptation, and biogeographic distribution.
Genetic characteristic	**Chromosome number (2n)**	Chrmsm_min Chrmsm_mode Chrmsm_max	Chromosome number (2n) for the taxon when available. The various columns correspond to various numbers measured.	Continuous values (no unit)	Reflects genetic diversity, evolutionary history, and adaptability to environmental pressures.

**Table 2.** Details on ecosystem services data collected for the endemic plant taxa of Greece and their assigned functions.

**Characteristic**	**Sub-types**	**Description**	**Range / Unit**	**Ecological function**
**Ecosystem services provision**	Aromatic, Medicinal, Traditional_medicine, Antimicrobial, Environmental_benefit, Industrial_use	Category of ecosystem services documented for the taxon.	Discreet values (3): Yes (x): documented use. No (No): absence of use or toxicity. Maybe (M): suggested use in literature without scientific proof.	Reflects human–ecosystem interactions: provisioning, regulating, cultural and supporting.
