# Supplementary material for: The First Functional Traits Dataset for the Endemic Flora of Greece: morphology, ecology and ecosystem services
Source: Biodivers Data J. 2026 Feb 10;14:e180342. doi: 10.3897/BDJ.14.e180342 (PMC13291638; doi:10.3897/BDJ.14.e180342)
Supplement: Supplementary material 1 — Functional traits of the Greek endemic taxa – Bibliography per family [file bdj-14-e180342-s001.pdf]

The First Functional Traits Dataset for the Endemic Flora of Greece: morphology, ecology and ecosystem services

Article type: Data Paper (Biodiversity)

Authors: Alexian Cheminal[1]*, Elpida Karadimou[1], Elisa Aubourg[2], Ioannis P. Kokkoris[3], Athanasios Kallimanis[4], Panayotis Dimopoulos[1]

[1] Laboratory of Botany, Department of Biology, University of Patras, 26504 Patras, Greece

[2] L'Institut Agro Dijon (ex-Agrosup Dijon), University of Burgundy, 21000 Dijon, France

[3] Department of Sustainable Agriculture, University of Patras, 2 G. Seferi St., 30131 Agrinio, Greece

[4] School of Biology, Aristotle University of Thessaloniki, 54124 Thessaloniki, Greece

Corresponding author: Alexian Cheminal (alexian.cheminal@upatras.gr)

Supplementary material 1: Functional traits of the Greek endemic taxa – Bibliography per family

General

Arambatzis, Theodoros I. 1998. *Θάμνοι Και Δέντρα Στην Ελλάδα*. Vol. 1. 2 vols.

———. 2001. *Θάμνοι Και Δέντρα Στην Ελλάδα*. Vol. 2. 2 vols.

Chytrý, M., M. Řezníčková, P. Novotný, D. Holubová, Z. Preislerová, F. Attorre, I. Biurrun, et al. 2024. "FloraVeg.EU – an Online Database of European Vegetation, Habitats and Flora." *Applied Vegetation Science* 27 (e12798). https://doi.org/10.1111/avsc.12798.

Strid, Arne. 2024a. *Atlas of the Hellenic Flora*. Vol. 3. 3 vols. Broken Hill Publishers.

———. 2024b. *Atlas of the Hellenic Flora*. Vol. 2. 3 vols. Broken Hill Publishers.

———. 2024c. *Atlas of the Hellenic Flora*. Vol. 1. 3 vols. Broken Hill Publishers.

Strid, Arne, and Kit Tan. 1991a. *Mountain Flora of Greece*. Vol. 1. 2 vols. Edinburgh University Press.

———. 1991b. *Mountain Flora of Greece*. Vol. 2. 2 vols. Edinburgh University Press.

Tutin, T.G., N.A. Burges, A.O. Chater, J.R. Edmondson, V.H. Heywood, D.M. Moore, D.H. Valentine, S.M. Walters, and D.A. Webb. 1993a. *Flora Europaea*. 2nd ed. Vol. 5. 5 vols. Cambridge University Press.

———. 1993b. *Flora Europaea*. 2nd ed. Vol. 4. 5 vols. Cambridge University Press.

———. 1993c. *Flora Europaea*. 2nd ed. Vol. 3. 5 vols. Cambridge University Press.

———. 1993d. *Flora Europaea*. 2nd ed. Vol. 2. 5 vols. Cambridge University Press.

———. 1993e. *Flora Europaea*. 2nd ed. Vol. 1. 5 vols. Cambridge University Press.

Alliaceae

Acar, Sevgi, Sezgin Ayan, and Barış Bani. 2015. "Morphological and Taxonomic Investigations on a Local Endemic Species: Allium Ilgazense N. Özhatay." *Australian Journal of Crop Science* 9 (3): 175–84.

Biel, Burkhard, Kit Tan, and Dimitrios Tzanoudakis. 2006. "A New Autumn-Flowering Species of *Allium (Liliaceae)* from the Island of Sifnos (Cyclades, Greece)." *Willdenowia* 36 (1): 367–72. https://doi.org/10.3372/wi.36.36132.

Bogdanovic, Sandro, Cristian Brullo, Salvatore Brullo, Gianpietro Giusso del Galdo, Carmelo Maria Musarella, and Cristina Salmeri. 2011. "Allium Achaium Boiss.(Alliaceae), a Critical Species of Greek Flora." *Candollea* 66 (1): 57–64.

Bogdanović, Sandro, Cristian Brullo, Salvatore Brullo, Gianpietro Giusso Del Galdo, Carmelo Maria Musarella, and Cristina Salmeri. 2011. "Allium Cithaeronis Bogdanović, C. Brullo, Brullo, Giusso, Musarella & Salmeri (Alliaceae), a New Species from Greece." *Candollea* 66 (2): 377–82. https://doi.org/10.15553/c2011v662a15.

Bogdanović, Sandro, Salvatore Brullo, Božena Mitić, and Cristina Salmeri. 2008. "A New Species of *Allium* (Alliaceae) from Dalmatia, Croatia." *Botanical Journal of the Linnean Society* 158 (1): 106–14. https://doi.org/10.1111/j.1095-8339.2008.00790.x.

Boissier, Edmond. 1859a. *Diagnoses plantarum novarum praesertim Orientalium nonnullis Europaeis Boreali-Africanisque additis*. Vol. 4. 2. Soc. Phys. Genev. Sodali. https://bibdigital.rjb.csic.es/records/item/10731-diagnoses-plantarum-orientalium-novarum-series-secunda-n-ordm-4.

———. 1859b. “Diagnoses plantarum Orientalium novarum [...] Series secunda. N.º 4.” *Diagnoses Plantarum Orientalium Novarum, Series Secunda* 4:118.

Brullo, Cristian, Salvatore Brullo, Gian Pietro Giusso del Galdo, and Cristina Salmeri. 2010. “Allium Makrianum (Alliaceae), a New Autumnal Species from Greece.” *Phyton (Horn, Austria)* 49 (2): 267–78.

Brullo, S., A. Guglielmo, P. Pavone, and C. Salmeri. 2001. “Cytotaxonomical Notes on Some Rare Endemic Species of Allium (Alliaceae) from Greece.” *Caryologia* 54 (1): 37–57. https://doi.org/10.1080/00087114.2001.10589212.

Brullo, S, A Guglielmo, P Pavone, and C Salmeri. 2007. “Cytotaxonomic Considerations on Allium Stamineum Boiss. Group (Alliaceae).”

Brullo, S., P. Pavone, C. Salmeri, and M. C. Terrasi. 2009. “*Allium Garganicum* (Alliaceae), a New Species from Apulia (SE Italy).” *Plant Biosystems - An International Journal Dealing with All Aspects of Plant Biology* 143 (sup1): S78–84. https://doi.org/10.1080/11263500903487765.

Brullo, S., and C. Salmeri. 2021. “Taxonomic Investigation on Allium Hirtovaginum Group (Amaryllidaceae) from East Mediterranean Area.” *Flora Mediterranea* 31 (Special Issue). https://doi.org/10.7320/FlMedit31SI.169.

Brullo, Salvatore, Gianpietro Giusso del Galdo, and Maria Carmen Terrasi. 2008. “Allium Aeginiense Brullo, Giusso & Terrasi (Alliaceae), a New Species from Greece.” *Candollea* 63:197–203.

Brullo, Salvatore, Anna Guglielmo, Pietro Pavone, Cristina Salmeri, and M. Carmen Terrasi. 2003. “Three New Species of *Allium* Sect. *Codonoprasum* from Greece.” *Plant Biosystems - An International Journal Dealing with All Aspects of Plant Biology* 137 (2): 131–40. https://doi.org/10.1080/11263500312331351391.

Brullo, Salvatore, Pietro Pavone, and Cristina Salmeri. 1992a. “Allium Rhodiacum (Liliaceae), a New Species from Rhodos (Greece).” *Willdenowia* 22 (1/2): 89–95.

———. 1992b. “Allium Rhodiacum (Liliaceae), a New Species from Rhodos (Greece).” *Willdenowia* 22 (1/2): 89–95.

———. 1997. “Allium Karistanum (Liliaceae), a New Species from Evvia (Greece).”

———. 1999. “Allium Archeotrichon (Alliaceae), a New Species from Rhodos (Dodekannìsos, Greece).” *Nordic Journal of Botany* 19 (1): 41–46. https://doi.org/10.1111/j.1756-1051.1999.tb01901.x.

———. 2015. “Biosystematic Researches on Allium Cupani Group (Amaryllidaceae) in the Mediterranean Area.” *Flora Mediterranea* 25 (Special Issue). https://doi.org/10.7320/FlMedit25SI.209.

Brullo, Salvatore, Pietro Pavone, Cristina Salmeri, and Dimitris Tzanoudakis. 1994. “Cytotaxonomical Revision or the Allium Obtusiflorum Group (Alliaceae).” *Flora Mediterranea*.

Brullo, Salvatore, Pietro Pavone, and Dimitris Tzanoudakis. 1993. “Allium Lagarophyllum (Liliaceae), a New Species from Greece.” *Willdenowia* 23 (1/2): 107–11.

Brullo, Salvatore, and Cristina Salmeri. 2021a. “Taxonomic Investigation on Allium Hirtovaginum Group (Amaryllidaceae) from East Mediterranean Area.” *Flora Mediterranea* 31 (Special Issue). https://doi.org/10.7320/FlMedit31SI.169.

———. 2021b. “Taxonomic Investigation on Allium Hirtovaginum Group (Amaryllidaceae) from East Mediterranean Area.” *Flora Mediterranea* 31 (Special Issue): 169–211. https://doi.org/10.7320/FlMedit31SI.169.

Brullo, Salvatore, and Dimitris Tzanoudakis. 1994. “Allium Ionicum (Liliaceae), a New Species from the Ionian Islands (W Greece).” *Willdenowia* 24 (1/2): 53–57.

Cattaneo, Cristina. 2020. “Description of a New Allium Species from Symi Island, (SE Aegean, Greece): Allium Carlstroemi Sp. Nov. (Amaryllidaceae).” *Parnassiana Archives* 8:103–11.

De Montmollin, Bertrand, and Wendy Strahm. 2005. *The Top 50 Mediterranean Island Plants: Wild Plants at the Brink of Extinction, and What Is Needed to Save Them*. IUCN.

Galanos, Christos J., and Dimitris Tzanoudakis. 2017. “Allium Symiacum (Amaryllidaceae), a New Species from Symi Island (SE Aegean, Greece).” *Willdenowia* 47 (2): 107–13. https://doi.org/10.3372/wi.47.47202.

Galanos, Christos, and Dimitrios Tzanoudakis. 2019. “Allium Panormitisi (Amaryllidaceae), a New Autumn-Flowering Species from Symi Island, SE Aegean, Greece.” *Botanica Serbica* 43 (2): 197–203. https://doi.org/10.2298/BOTSERB1902197G.

Greuter, Werner. 2024. “The Greuter Herbarium in Palermo: An Inventory of Its Type Specimens Available Online, with Some Thoughts on Type Terminology (Occasional Papers from the Herbarium Greuter, N° 5).” *Plants* 13 (8): 1086.

Greuter, Werner, and Thomas Raus. 2010. “Med-Checklist Notulae, 28.” *Willdenowia* 39 (2): 335–45.

Ioannidis, Vasilis, and Dimitris Tzanoudakis. 2020. “Allium Arampatzisii (Amaryllidaceae) – a New Species from Northern Continental Greece.”

Kalpoutzakis, Eleftherios, Panayiotis Trigas, and Theophanis Constantinidis. 2012. “Allium Orestis Sp. Nov. (Amaryllidaceae) from Parnon and Taigetos Mountains, South Peloponnisos, Greece.” *Nordic Journal of Botany* 30 (2): 195–200. https://doi.org/10.1111/j.1756-1051.2011.01490.x.

Kobrlová, Lucie, Michaela Jandová, Kateřina Vojtěchová, Lenka Šafářová, and Martin Duchoslav. 2024. “New Estimates and Synthesis of Chromosome Number, Ploidy Level and Genome Size Variation in Allium Sect. Codonoprasum: A Step towards Understanding the Hitherto Unresolved Diversification and Evolution of the Section.” https://doi.org/10.21203/rs.3.rs-4972960/v1.

Musarella, Carmelo Maria, Salvatore Brullo, and Gianpietro Giusso Del Galdo. 2020. “Contribution to the Orophilous Cushion-like Vegetation of Central-Southern and Insular Greece.” *Plants* 9 (12): 1678.

Ohri, D., R. M. Fritsch, and P. Hanelt. 1998. “Evolution of Genome Size inAllium (Alliaceae).” *Plant Systematics and Evolution* 210 (1–2): 57–86. https://doi.org/10.1007/BF00984728.

Rechinger, K. H. 1961. “Die Flora von Euboea.” *Botanische Jahrbücher Für Systematik, Pflanzengeschichte Und Pflanzengeographie* 80 (4): 435.

Salmeri, Cristina. 1998. “Allium Brulloi (Alliaceae), a New Species from Astypalea (Aegean Islands, Greece).” *Willdenowia* 28 (1/2): 69–75.

Seregin, A P. 2004. “Additions to Allium Sect. Allium (Alliaceae) from North Africa.”

Trigas, Panayiotis, and Pepy Bareka. 2020. “Allium

Stamatiadae, a New Species of Sect. Codonoprasum (Amaryllidaceae) from Andros Island (Aegean Archipelago, Greece).” *Phytotaxa* 443 (3): 287–94. https://doi.org/10.11646/phytotaxa.443.3.5.
Trigas, Panayiotis, Gregoris Iatrou, and Dimitris Tzanoudakis. n.d. “Allium Apergii Sp. Nov. (Alliaceae, A. Sect. Codonoprasum) from Evvia Island, Greece.”
Trigas, Panayiotis, Eleftherios Kalpoutzakis, and Theophanis Constantinidis. 2017. “Two New Allium (A. Sect. Cupanioscordum, Amaryllidaceae) Species from Greece.” *Phytotaxa* 297 (2): 179. https://doi.org/10.11646/phytotaxa.297.2.5.
Trigas, Panayiotis, Eleftherios Kalpoutzakis, Epaminondas Kalogiannis, Anna-Thalassini Valli, Konstantinos Kougioumoutzis, Konstantinos Katopodis, and Theophanis Constantinidis. 2021. “Noteworthy New Floristic Records from Greece.” *Botanica Serbica* 45 (2): 321–31. https://doi.org/10.2298/BOTSERB2102321T.
Trigas, Panayiotis, and Dimitris Tzanoudakis. 2000. “Allium Runemarkii (Liliaceae), a New Species from the Island of Ewia (W Aegean, Greece).” *Nordic Journal of Botany* 20 (1): 89–92. https://doi.org/10.1111/j.1756-1051.2000.tb00737.x.
Tsakiri, Maria, Konstantinos Kougioumoutzis, and Gregoris Iatrou. 2016. “Contribution to the Vascular Flora of Chalki Island (East Aegean, Greece) and Biomonitoring of a Local Endemic Taxon.” *Willdenowia* 46 (1): 175–90. https://doi.org/10.3372/wi.46.46114.
Tzanoudakis, D. 1986. “Chromosome Studies in the Greek Flora. II. Karyotypes of Four Aegean Endemics of Allium Sect. Codonoprasum (Liliaceae).” *Willdenowia* 16 (1): 203–11.
Tzanoudakis, Dimitris. 1983a. “Karyotypes of Ten Taxa of *Allium* Section *Scorodon* from Greece.” *Caryologia* 36 (3): 259–84. https://doi.org/10.1080/00087114.1983.10797667.
———. 1983b. “New Taxa of Allium from Greece.” *Candollea: Journal International de Botanique Systématique = International Journal of Systematic Botany* 38 (1): 317. https://doi.org/10.5169/seals-879865.
Tzanoudakis, Dimitris, and Fania Kollmann. 1991. “Allium Chalkii (Liliaceae), a New Species from the Eastern Aegean Island of Chalki (Greece).” *Israel Journal of Botany* 40 (1): 61–64.
Tzanoudakis, Dimitris, and Kit Tan. 2000. “Allium Samothracicum Tzanoudakis, Strid & Kit Tan, a New Species from the North Aegean Area, Greece.” *Portugaliae Acta Biologica* 19 (1): 355–60.
Tzanoudakis, Dimitris, and Panayiotis Trigas. 2015a. “Allium Occultum, a New Species of A. Sect. Codonoprasum (Amaryllidaceae) from Skiros Island (W Aegean, Greece).” *Phytotaxa* 202 (2): 135–42. https://doi.org/10.11646/phytotaxa.202.2.5.
———. 2015b. “Allium Occultum, a New Species of A. Sect. Codonoprasum (Amaryllidaceae) from Skiros Island (W Aegean, Greece).” *Phytotaxa* 202 (2): 135. https://doi.org/10.11646/phytotaxa.202.2.5.
Tzanoudakis, Dimitris, Maria Tsakiri, and Thomas Raus. 2019. “What Is Allium Achaium Boiss. & Orph.? Disentangling the Taxonomy of a Greek Mountain Species.” *Willdenowia* 49 (2): 231–39.

Amaryllidaceae

Alpine Garden Society. 2018. “Galanthus Ikariae.” Alpine Garden Society Plant Encyclopaedia. 2018. http://encyclopaedia.alpinegardensociety.net/plants/Galanthus/ikariae.
Gage, Ewan, and Paul Wilkin. 2008. “A Morphometric Study of Species Delimitation in Sternbergia Lutea (Alliaceae, Amaryllidoideae) and Its Allies S. Sicula and S. Greuteriana.” Botanical Journal of the Linnean Society 158 (3): 460–69. https://doi.org/10.1111/j.1095-8339.2008.00903.x.
Missouri Botanical Garden. 2024. “Galanthus Ikariae.” Missouri Botanical Garden - Plant Finder. 2024. https://www.missouribotanicalgarden.org/PlantFinder/PlantFinderDetails.aspx?taxonid=275869.
Tan, Kit. 2014a. “Galanthus Samothracicus (Amaryllidaceae) from the Island of Samothraki, Northeastern Greece.”
———. 2014b. “Galanthus Samothracicus (Amaryllidaceae) from the Island of Samothraki, Northeastern Greece.”

Apiaceae

Boissier, Edmond, Edmond Boissier, and R. Buser. 1872. *Flora Orientalis : Sive, Enumeratio Plantarum in Oriente a Graecia et Aegypto Ad Indiae Fines Hucusque Observatarum*. Vol. 2. Basileae: H. Georg. https://doi.org/10.5962/bhl.title.20323.
Conti, Fabio, Fabrizio Bartolucci, Gianluigi Bacchetta, Riccardo Pennesi, Dmitar Lakušić, and Marjan Niketić. 2021. “A Taxonomic Revision of the Siler Montanum Group (Apiaceae) in Italy and the Balkan Peninsula.” *Willdenowia* 51 (3): 321–47. https://doi.org/10.3372/wi.51.51301.
Cretan Flora. 2010. “Eryngium Amorginum - Species Description.” Cretan Flora. 2010. https://www.cretanflora.com/Flowers_Protection_text.html.
Duran, A., and H. Duman. 1999. “Two New Species of Umbelliferae from Southern Turkey.” *Edinburgh Journal of Botany* 56 (1): 47–53.
Egli, Bernhard, Pedro Gerstberger, Werner Greuter, and Horst Risse. 1990. “Horstrissea Dolinicola, a New Genus and Species of Umbels (Umbelliferae, Apiaceae) from Kriti (Greece).” *Willdenowia* 19 (2): 389–99.
Hadaček, Franz, and Rosabelle Samuel. 1994. “Chromosome Counts and Chemotaxonomy in Peucedanum Sect. Peucedanum (Apiaceae, Apioideae) from the Balkan Peninsula.” *Willdenowia* 24 (1/2): 33–48.
Hartvig, P. 1984. “Two New Species of Apiaceae from Greece.” *Willdenowia* 13 (2): 289–93.
Heywood, V. H. 1968. “Flora Europaea Notulae Systematicae Ad Floram Europaeam Spectantes.” *Feddes Repertorium* 79 (1–2): 1–68. https://doi.org/10.1002/fedr.19680790102.
“IUCN Top 50 - Bupleurum Kakiskalae.” n.d. Accessed January 17, 2025. https://top50.iucn-mpsg.org/species/17.
Lamarck, Jean Baptiste Pierre Antoine de Monet de, and Jean Louis Marie Poiret. 1810. *Encyclopédie méthodique : Botanique : Supplément*. Paris : Agasse. http://archive.org/details/encyclopdiemsup04lama.

Nymas, Carol F. 1855. *Sylloge florae europaeae seu plantarum vascularium Erupae indigenarum enumeratio*. https://bibdigital.rjb.csic.es.
Polymenakos, Kostas, Kit Tan, and Vasilis Pantavos. 2024. "Torilis Samia (Apiaceae) and Ehrharta Erecta (Poaceae), Two New Species for Greece." *Phytologia Balcanica* 30 (2): 197–202.
Prokopiou, Lydia, Abealghani Halahlah, Spyros Grigorakis, Christini Fournaraki, Eugene Kokkalou, and Anastasia Karioti. 2022. "Threatened Cretan Species *Chaerophyllum Creticum* Boiss. & Heldr.: Phenolic Profile by HPLC-PDA-MS and *in Vitro* Antioxidant Capacity." *Natural Product Research* 36 (15): 3992–98. https://doi.org/10.1080/14786419.2021.1889545.
Rechinger, K. H. 1934. "Dreizehn Neue Pflanzenarten Aus Griechenland." *Magyar Botanikai Lapok* 33 (1–12): 9.
———. 1965. "Zwei Neue Arten Aus Der Griechischen Flora." *Österreichische Botanische Zeitschrift* 112 (1/2): 186–87.
Snogerup, Sven, and Britt Snogerup. 2001. "Bupleurum L.(Umbelliferae) in Europe—1. The Annuals, B. Sect. Bupleurum and Sect. Aristata." *Willdenowia* 31 (2): 205–308.

Araceae

Boyce, P. C. 1987. "A New Species of Arum L. from Crete." *Aroideana* 10 (1): 6–8.
Cousturier, MM. Paul, and Michel Gandoger. 1916. "Herborisations En Crète (1913-1914), Première Partie." In *Bulletin de La Société Botanique de France*, v.63-65 (1916-1918):1–734. Paris: La Société. https://www.biodiversitylibrary.org/item/273147.

Asparagaceae

Karlén, T. 1984. "Muscari Pulchellum (Liliaceae) and Associated Taxa in Greece and W Turkey." *Willdenowia* 14 (1): 89–118.
Speta, F. 2000. "Beitrag zur Kenntnis der Gattung Prospero SALISB. (Hyacinthaceae) auf der griechischen Insel Kreta - Contribution to the knowledge of the genus Prospero Salisb. (Hyacinthaceae) on the Greek island of Crete." *Linzer Biol. Beitr.* 32 (2): 1323–26.
Tzanoudakis, Dimitris, and Zaharias Kypriotakis. 1998. "A New Polyploid Scilla (Liliaceae) from the Cretan Area (Greece)." *Folia Geobotanica* 33 (1): 103–8.

Asteraceae

Ainouche, Malika L., Randall J. Bayer, Jean-Pierre Gourret, Alain Defontaine, and Marie-Thérèse Misset. 1999. "The Allotetraploid Invasive Weed Bromus Hordeaceus L. (Poaceae): Genetic Diversity, Origin and Molecular Evolution." *Folia Geobotanica* 34 (4): 405–19.
Akademie der Wissenschaften in Wien. 1879. *Denkschriften Der Kaiserlichen Akademie Der Wissenschaften / Mathematisch-Naturwissenschaftliche Classe*. Vol. Bd.61;Index Bd.41-61 (1879-1894). Wien: Aus der Kaiserlich-Königlichen Hof- und Staatsdruckerei. https://www.biodiversitylibrary.org/item/31617.
———. 1884. *Denkschriften Der Kaiserlichen Akademie Der Wissenschaften / Mathematisch-Naturwissenschaftliche Classe*. Vol. Bd.48 (1884). Wien: Aus der Kaiserlich-Königlichen Hof- und Staatsdruckerei. https://www.biodiversitylibrary.org/item/31429.
Aldén, Björn. 1976. "Floristic reports from the high mountains of Pindhos, Greece." *Botaniska notiser* 129 (3): 303.
Anagnostopoulos, Anastasios. 1997. "Karyotype Variation in Crepis Fraasii and C. Reuteriana (Asteraceae) in Greece," 6.
Bergmeier, Erwin. 2010. "Filago Wagenitziana (Asteraceae, Gnaphalieae), a New Species from Western Crete, Greece." *Willdenowia* 40 (2): 183–88.
Bergmeier, Erwin, and Arne Strid. 2019. "Centaurea Devasiana – a New Species from Prespa, NW Greece."
Boissier, Edmond. 1849a. *Diagnoses Plantarum Orientalium Novarum.* Vol. 11. Parisiis : Typis Marci Ducloux et Cons. Via École-de-Médecine, 80. https://www.biodiversitylibrary.org/bibliography/72074.
———. 1849b. *Diagnoses plantarum Orientalium novarum.* Vol. 10. https://bibdigital.rjb.csic.es.
———. 1854. *Diagnoses Plantarum Orientalium Novarum, Series 2, Vol. 3, N.º 1 - 6*. http://archive.org/details/e.-boissier-diagnoses-plantarum-orientalium-novarum-series-secunda.-n.o-1-1854.
———. 1856. *Diagnoses plantarum Orientalium novarum.* Vol. 5. 2. https://bibdigital.rjb.csic.es.
———. 1859. *Diagnoses plantarum Orientalium novarum.* Vol. 6. 2. https://bibdigital.rjb.csic.es.
———. 1893a. *Bulletin de l'Herbier Boissier*. Genève : Impr. Romet. http://archive.org/details/bulletindelherbi06herb.
———. 1893b. *Bulletin de l'Herbier Boissier*. Vol. v.6 1898. Genève: Impr. Romet. https://www.biodiversitylibrary.org/item/105008.
———. 1893c. *Bulletin de l'Herbier Boissier*. Vol. v.5 1897. Genève: Impr. Romet. https://www.biodiversitylibrary.org/item/105007.
Boissier, Edmond, Edmond Boissier, and R. Buser. 1875. *Flora Orientalis : Sive, Enumeratio Plantarum in Oriente a Graecia et Aegypto Ad Indiae Fines Hucusque Observatarum*. Vol. 3. Basileae: H. Georg. https://doi.org/10.5962/bhl.title.20323.
Bornmüller, J. 1921. "Zur Gattung Centaurea." *Beihefte zum Botanischen Centralblatt. Original-Arbeiten. [...] Zweite Abteilung: Systematik, Pflanzengeographie, angewandte Botanik etc.* 38:459.
Bräutigam, Siegfried, and Werner Greuter. 2007. "A New Treatment of Pilosella for the Euro-Mediterranean Flora [Notulae Ad Floram Euro-Mediterraneam Pertinentes 24]." *Willdenowia* 37 (1): 123–37.
Constantinidis, Theophanis, Georgia Kamari, and Dimitrios Phitos. 1997. "A Cytological Study of 28 Phanerogams from the Mountains of SE Sterea Ellas, Greece." *Willdenowia* 27 (1/2): 121–42.
Degen, Árpád von, and Ignaz Dörfler. 1897. "Zur Flora Albaniens Und Macedoniens." *Denkschriften Der Kaiserlichen Akademie Der Wissenschaften / Mathematisch-Naturwissenschaftliche Classe* 64:726.

Dörfler, Ignaz. 1894. “Bericht Über Das Laufende Tauschjahr.” *Jahres-Katalog pro 1894 Des Wiener Botanischen Tauschvereins*, 6.

Engler, Adolf. 1922. *Das Pflanzenreich :Regni Vegetablilis Conspectus*. Vol. Heft 79 (1922). Leipzig: W. Engelmann. https://www.biodiversitylibrary.org/item/61956.

Engler, Adolf, and Adolf Engler. 1921a. *Das Pflanzenreich :Regni Vegetablilis Conspectus*. Vol. Heft 75-77;79 (1921-1923). Leipzig: W. Engelmann. https://www.biodiversitylibrary.org/item/176822.

———. 1921b. “Euhieracium - Sect. X. Pannosa; n.212.” In *Das Pflanzenreich :Regni Vegetablilis Conspectus*, Heft 75-77;79 (1921-1923):577 + 1043. Leipzig: W. Engelmann. https://www.biodiversitylibrary.org/item/176822.

Fedde, Friedrich. 1919. *Repertorium Specierum Novarum Regni Vegetabilis*. Vol. v.16 (1919-1920). Berlin: Selbstverlag des Herausgebers. https://www.biodiversitylibrary.org/item/7040.

Franzén, Roy. 1986. “Taxonomy of the Achillea Clavennae Group and the A. Ageratifolia Group (Asteraceae, Anthemideae) on the Balkan Peninsula.” *Willdenowia* 16 (1): 13–33.

Gamal-Eldin, Elsayeda, and G. Wagenitz. 1984. “Eine Neue Centaurea-Art Der Sektion Phalolepis (Compositae) Aus Nordgriechenland (Chalkidike) - A New Species of Centaurea of the Phalolepis Section (Compositae) from Northern Greece (Chalkida).” *Willdenowia* 13 (2): 323–27.

Georgiadis, Th, and D Phitos. 1977. “Deux Centaurea Nouvelles de La Section Phalolepis (Cass.) D.C. de La Grèce.” *Biologie et Ecologie Mediterraneenne* 4 (1): 3–9.

Georgiadis, Theodore. 1981. “A propos de Centaurea ipsaria Stoj. & Kit. et de C. thasia Hayek (sectio Acrolophus (Cass.) D.C.) de l’île de Thasos (Grèce).” *Botanika Chronika* 1 (1): 14–17.

Georgiadis, Theodoros. 1979. “Centaurea Musakii: A New Species from Thessalia (Greece).” *Bot. Not.* 132 (3): 311–12.

Georgiadis, Theodoros, Georgios Dimitrellos, and Eugenia Routsi. 1996. “Centaurea Messenicolasiana (Asteraceae), A New Species of C. Sect. Phalolepis (Cass.) DC. from Greece.” *Willdenowia* 25 (2): 561–69.

Gottschlich, G. 2023. “Four New Hieracium Taxa (Compositae) from the Balkans (North Macedonia, Montenegro) and Greece and One New Hieracium Record for Europe.” *Annalen Des Naturhistorischen Museums in Wien. Serie B Für Botanik Und Zoologie* 125:143–54.

Gottschlich, Günter, and Franz G Dunkel. 2018. “New Taxa of Hieracium and Pilosella (Asteraceae) - from Northern and Central Greece.” *Stapfia* 109:3–24.

———. 2019. “New Taxa of Hieracium and Pilosella (Asteraceae) from Northern Greece II.” *STAPFIA* 111:5–32.

Gottschlich, Günter, Konstantina Melikoki, Eleni Eleftheriadou, and Konstantinos Theodoropoulos. 2013. “Three New Hieracium Taxa (Asteraceae, Cichorieae) from Mt Cholomon, Chalkidiki, Central Makedonia, Greece.” *Willdenowia* 43 (1): 59–64.

Goula, Katerina, and Theophanis Constantinidis. 2021a. “<p><strong>Taxonomic Diversity and Karyology of <em>Anthemis Rigida</Em> (Anthemideae, Asteraceae) in the Aegean, Greece</Strong></P>.” *Phytotaxa* 484 (1): 129–43. https://doi.org/10.11646/phytotaxa.484.1.7.

———. 2021b. “Taxonomic Diversity and Karyology of Anthemis Rigida (Anthemideae, Asteraceae) in the Aegean, Greece.” *Phytotaxa* 484 (1): 129–43. https://doi.org/10.11646/phytotaxa.484.1.7.

———. 2023. “Anthemissect.Hiorthia (Asteraceae) on Kriti Island, Greece: High Ploidy Levels and a New Species.” *PhytoKeys* 229 (July):113. https://doi.org/10.3897/phytokeys.229.102703.

Goula, Katerina, Konstantinos Touloumis, Panayotis Dimopoulos, and Theophanis Constantinidis. 2022. “A Morphometric and Karyological Study of the Anthemis Macedonica Group (Asteraceae, Anthemideae) Reveals a New Species from Greece.” *Plants* 11 (21): 3006. https://doi.org/10.3390/plants11213006.

Greuter, Werner. 1967. “Beiträge zur Flora der Südägäis 8 - 9.” *Bauhinia* 3 (2): 243–24.

———. 1979. “Quisquiliae Floristicae Graecae, 4. Lamyropsis Carpini (Compositae), a New Species from NW.Greece.” *Willdenowia* 9 (1): 57–66.

Greuter, Werner, and Kostas Papanicolaou. 1979. “A new species of Centaurea from Mt Pangaion.” *Botaniska notiser* 132 (4): 471.

Greuter, Werner, and Eckhard Von Raab-Straube. 2007. “Euro+Med Notulae, 3 [Notulae Ad Floram Euro-Mediterraneam Pertinentes 24].” *Willdenowia* 37 (1): 139–90.

Greuter, Werner, and Karl Heinz Rechinger. 1967. “Flora der Insel Kythera.” *Boissiera* 13:142.

Gustafsson, M., and Sven Snogerup. 1972. “Scorzonera Scyria, a New Chasmophytic Species from Greece.” *Bot. Not.* 125 (4): 323–28.

Halácsy, Eugen v., and Eugen v Halácsy. 1901. *Conspectus Florae Graecae*. Vol. v.1. Lipsiae: Sumptibus Guilelmi Engelmann. https://doi.org/10.5962/bhl.title.9919.

Halacsy, Eugen von. 1894. “Flora von Aetolien Und Acarnanien.” In *Denkschriften Der Kaiserlichen Akademie Der Wissenschaften / Mathematisch-Naturwissenschaftliche Classe*, Bd.61;Index Bd.41-61 (1879-1894):315. Wien: Aus der Kaiserlich-Königlichen Hof- und Staatsdruckerei. https://www.biodiversitylibrary.org/item/31617.

Hayek, A. de. 1913. “XXII. Centaureae Novae et Combinationes Nominum Centaurearum Novae.” *Repertorium Novarum Specierum Regni Vegetabilis* 12 (4–8): 123–26. https://doi.org/10.1002/fedr.19130120409.

Hayek, August v. 1914. “Neue Orientalische Pflanzenarten.” *Österreichische Botanische Zeitschrift* 64 (8): 358–60.

HEYWOOD, V. H. 1975. “Flora Europaea. Notulae Systematicae Ad Floram Europaeam Spectantes: No. 18.” *Botanical Journal of the Linnean Society* 71 (3): 191–210. https://doi.org/10.1111/j.1095-8339.1975.tb02535.x.

Holub, Josef. 1977. “New Names in Phanerogamae 5.” *Folia Geobotanica & Phytotaxonomica* 12 (3): 293–311.

Inceer, Huseyin. 2021. “Lectotypification of the Name Chamaemelum Heterolepis, the Basionym of Tripleurospermum Heterolepis and Taxonomic Notes on T. Tempskyanum (Asteraceae).” *Botanica Serbica* 45 (2): 347–52.

Inceer, Huseyin, and Sema Hayirlioglu-Ayaz. 2010. “Chromosome Numbers in Tripleurospermum Sch. Bip. (Asteraceae) and Closely Related Genera: Relationships between Ploidy Level and Stomatal

Length.” *Plant Systematics and Evolution* 285 (3): 149–57. https://doi.org/10.1007/s00606-009-0266-5.

Kamari, G. 1992. “Karyosystematic Studies on Three Crepis Species (Asteraceae) Endemic to Greece.” *Plant Systematics and Evolution* 182 (1): 1–19. https://doi.org/10.1007/BF00941411.

Kamari, Georgia, Pepy Bareka, Theophanis Constantinidis, and Dimitrios Phitos. 2003. “Karyosystematic Studies of Plant Taxa from the East Mediterranean Region (Grece, Cyprus, Syria).” *Phytologia Balcanica* 9 (3): 487–502.

Kamari, Georgia, and Per Hartvig. 1988. “Crepis Merxmuelleri (Asteraceae), a New Species from NW Greece.” *Willdenowia* 18:63–66.

Kamari, Georgia, and Ursula Matthas. 1986. “Cytotaxonomical Contributions on the Flora of Crete. III.” *Willdenowia* 15 (2): 515–20.

Kamari, Georgia, and Arne Strid. 1989. “Crepis Arcuata (Asteraceae), a New Species from N Greece.” *Willdenowia* 19 (1): 79–82.

Kirschner, Jan, C. E. Sonck, and Jan Štěpánek. 1989. “Four New Taraxacum Species of Sect. Palustria from Greece.” *Annales Botanici Fennici* 26 (3): 343–48.

Kirschner, Jan, and Jan Štěpánek. 1998. “A Revision of Taraxacum Sect. Piesis (Compositae).” *Folia Geobotanica* 33 (4): 391–414.

Le M. Moore, S. 1878. “Alabastra Diversa, Pars Secunda.” In *Journal of Botany, British and Foreign*, by Henry Trimen, James Britten, John Ramsbottom, Berthold Seemann, and A. B. Rendle, 16:1–418. London: Robert Hardwicke. https://www.biodiversitylibrary.org/item/35887.

Linné, Carl von. 1753. *Caroli Linnaei ... Species Plantarum :Exhibentes Plantas Rite Cognitas, Ad Genera Relatas, Cum Differentiis Specificis, Nominibus Trivialibus, Synonymis Selectis, Locis Natalibus, Secundum Systema Sexuale Digestas...* Vol. 2. Holmiae: Impensis Laurentii Salvii. https://doi.org/10.5962/bhl.title.669.

Linné, Carl von, and Curtio Sprengel. 1826. *Systema Vegetabilium*. 16th ed. Vol. 3. Gottingae: sumtibus Librariae Dieterichianae. https://doi.org/10.5962/bhl.title.822.

Löve, Askell. 1983. “IOPB Chromosome Number Reports LXXVIII.” *Taxon* 32 (1): 138–41.

Marhold, Karol, and Jaromír Kučera. 2016. “IAPT/IOPB Chromosome Data 21.” *Taxon* 65 (3): 673–76.

Musiał, Krystyna, Vladimir Vladmirov, and Zbigniew Szeląg. 2020. “Chromosome Numbers in Hieracium (Asteraceae) from Central and Southeastern Europe VI.” *Acta Biologica Cracoviensia s. Botanica* 62 (2): 43–50. https://doi.org/10.24425/ABCSB.2020.131672.

Nymas, Carol F. 1855. *Sylloge florae europaeae seu plantarum vascularium Erupae indigenarum enumeratio*. https://bibdigital.rjb.csic.es.

Pasta, S., A. Perez-Graber, L. Fazan, and B De Montmollin. 2017. “The Top 50 Mediterranean Island Plants, Update 2017.” Neufchâtel (Switzerland): IUCN/SSC/Mediterranean Plant Specialist Group. https://top50.iucn-mpsg.org/book.

Pelser, Pieter B., Jan Frits Veldkamp, and Ruud van der Meliden. 2006. “New Combinations in Jacobaea Mill. (Asteraceae - Senecioneae).” *Compositae Newsletter* 44:6.

Phitos, D. 1963. “Eine Neue Art Der Gattung Centaurea Aus Der Ägäis - A New Species of the Genus Centaurea from the Aegean.” *Annalen Des Naturhistorischen Museums in Wien* 67:165–67.

Phitos, Dimitrios, and Theophanis Constantinidis. 1993a. “A New Species of Centaurea Sect. Phalolepis from Greece.” *Flora Mediterranea* 3:273–75.

———. 1993b. “A New Species of Centaurea Sect. Phalolepis from Greece.” *Flora Mediterranea* 3:273–75.

Phitos, Dimitrios, and Theodore Georgiadis. 1981. “A Propos Du Groupe de Centaurea Achaia Boiss. et Heldr. de La Section Acrocentron (Cass.) DC.” *Botanika Chronika* 1 (2): 104–7.

Pinar, Süleyman Mesut, and Hüseyin Eroğlu. 2017. “First Turkish Record of a Species from Family Asteraceae (Compositae): Onopordum Carduiforme Boiss.” *Bitlis Eren University Journal of Science and Technology*.

Radon, Johann. 1956. “Sitzung Vom 14. Juni 1956.” *Anzeiger Der Oesterreichischen Akademie Der Wissenschaften Mathematisch-Naturwissenschaftliche Klasse, Abt. I* 93 (9): 101.

Rechinger, K. H. 1939. “Zur Flora von Ostmazedonien Und Westthrazien.” *Botanische Jahrbücher Für Systematik, Pflanzengeschichte Und Pflanzengeographie* 69 (4): 526.

Richards, Adrian J. 2015. “Taraxacum Limnoticum (Asteraceae) from the Aegean, Greece.”

Sibthorp, Johannes, and Jacobus Edvardus Smith. 1813. *Flora Graecae Prodromus*. Vol. 2.

Sonck, C. E. 1984. “New Taraxacum Species from Greece.” *Annales Botanici Fennici* 21 (2): 157–70.

———. 1985a. “New Taraxacum Species from Greece II.” *Annales Botanici Fennici* 22 (2): 139–48.

———. 1985b. “New Taraxacum Species from Greece III.” *Annales Botanici Fennici* 22 (3): 255–62.

———. 1985c. “New Taraxacum Species from Greece III.” *Annales Botanici Fennici* 22 (3): 255–62.

———. 1986. “Taraxacum Subolivaceum (Sect. Palustria), Species Nova, from Greece, and T. Vexatum, Nomen Novum.” *Annales Botanici Fennici* 23 (2): 165–68.

———. 1993. “New Taraxacum Species from Greece. IV.” *Annales Botanici Fennici* 30 (3): 205–10.

Sonck, Carl Eric. 1999. “New Taraxacum Species from Greece. V.” *Annales Botanici Fennici* 36 (3): 211–17.

Štěpánek, Jan, and Jan Kirschner. 2022. “<p><strong><em>Taraxacum</Em> Sect. <em>Erythrocarpa</Em> in Europe in the Alps and Eastwards: A Revision of a Precursor Group of Relicts</Strong></P>.” *Phytotaxa* 536 (1): 7–52. https://doi.org/10.11646/phytotaxa.536.1.2.

Strid, Arne, and Kit Tan. 1991. *Moutain Flora of Greece*. Vol. 2. 2 vols. Edinburgh University Press.

———. 2003. “New Species of Erodium (Geraniaceae), Onosma (Boraginaceae) and Centaurea (Asteraceae) from Northern Greece.” *Phytologia Balcanica* 9 (3): 471–78.

———. 2009. “A New Species of Centaurea (Asteraceae) from the Island of Samothraki (NE Greece).” *Phytologia Balcanica* 15 (2): 185–89.

Sutorý, Karel. 2021. “<p><strong>Taxonomic and Nomenclatural Notes on <em>Jacobaea Othonnae</Em> (Asteraceae—Senecioneae—Senecioninae)</Strong></P>.” *Phytotaxa* 510 (1): 61–68. https://doi.org/10.11646/phytotaxa.510.1.6.

Sutorỳ, Karel. 2021. “Taxonomic and Nomenclatural Notes on Jacobaea Othonnae (Asteraceae-Senecioneae-Senecioninae).” *Phytotaxa* 510 (1): 61–68.

Szelag, Z., and V. Vladimirov. 2019. “The Species Intermediate between Hieracium Petrovae and H. Olympicum

(Asteraceae): A Treatment of H. Kritschimanum and Description of a New Species from Greece." *Phytotaxa* 402 (2): 107–13.

Trigas, P., Th. Constantinidis, and T. Touloumenidou. 2008. "A New Hexaploid Species of Centaurea Section Acrolophus (Asteraceae) from Evvia Island, Greece." *Botanical Journal of the Linnean Society* 158 (4): 762–74. https://doi.org/10.1111/j.1095-8339.2008.00870.x.

Trigas, Panayiotis, and Gregoris Iatrou. 2006. "The Local Endemic Flora of Evvia (W Aegean, Greece)." *Willdenowia* 36 (1): 257–70. https://doi.org/10.3372/wi.36.36121.

Turland, Nicholas J. 2008. "Anthemis Samariensis (Asteraceae, Anthemideae), a New Species from the Mountains of W Kriti (Greece)." *Willdenowia* 38 (1): 61. https://doi.org/10.3372/wi.38.38103.

Turrill, W. B. 1957. "Serratula in Crete." *Kew Bulletin* 12 (3): 391–92. https://doi.org/10.2307/4113700.

Uysal, Tuna, Kuddisi Ertugrul, Alfonso Susanna, and Núria Garcia-Jacas. 2009. "New Chromosome Counts in the Genus Centaurea (Asteraceae) from Turkey." *Botanical Journal of the Linnean Society* 159 (2): 280–86. https://doi.org/10.1111/j.1095-8339.2008.00939.x.

Van Soest, J. L. 1954. "Taraxacum Braun-Blanquettii et Quelques Autres Taraxaca de France." *Vegetatio* 5/6 (1): 524–33.

———. 1971. "Quelques Nouvelles Especes de Taraxacum, Natives d'Europe II - Some New Species of Taraxacum, Native from Europe II." *Acta Botanica Neerlandica* 20 (1): 141–56.

Vandas, Karel. 1909. *Reliquiae Formánekianae : Enumeratio Critica Plantarum Vascularium, Quas Itineribus in Haemo Peninsula et Asia Minore (Bithynia) Factis Collegit Dr. Ed. Formánek*. Brunae: Comitiorum Marchionatus Moraviae. https://doi.org/10.5962/bhl.title.9969.

Vladimirov, Compiled Vladimir, Mehmet Aybeke, and Kit Tan. 2020. "New Floristic Records in the Balkans: 42."

———. 2021a. "New Floristic Records in the Balkans: 44."

———. 2021b. "New Floristic Records in the Balkans: 45."

Vladimirov, Vladimir, Mehmet Aybeke, and Kit Tan. 2022. "New Floristic Records in the Balkans: 48." *Phytologia Balcanica* 28 (2): 249–79. https://doi.org/10.7546/PhB.28.2.2022.9.

Vladimirov, Vladimir, Kamil Çoşkunçelebi, and Kit Tan. 2015. "A New Diploid Species of Pilosella (Asteraceae) from Turkey." *TURKISH JOURNAL OF BOTANY* 39:70–75. https://doi.org/10.3906/bot-1401-92.

Vonica, Ghizela, and Maria Cantor. 2012. "Centaurea Species Attractive Transylvanian (Romania) Endemic and Rare Plants with High Potential as Flowering Garden Plant." *Bulletin UASVM Horticulture* 69 (1): 354–62.

Wagenitz, G, and E Gamal-Eldin. 1985. "Zur Kenntnis der griechischen Centaurea-Arten der Sektion Acrocentron - About the Greek Centaurea species of the Acrocentron section." *Bot. Jahrb. Syst.* 107 (14): 95–127.

Wagenitz, Gerhard. 1971. "Centaurea Pseudocadmea, Eine Neue Art Der Sektion Phalolepis Aus Griechenland." *Annalen Des Naturhistorischen Museums in Wien* 75:243–47.

Wagenitz, Gerhard, ERWIN Bergmeier, THOMAS Gregor, LENZ Meierott, Lulezim Shuka, and Kit Tan. 2018. "A Synopsis of the Centaurea Soskae and Triniifolia Group (Centaurea Sect. Acrolophus) in the Prespa Area and Northern Pindos." *Phytotaxa* 348 (2): 77–89.

Wagenitz, Gerhard, and H. Runemark. 1970. "Die Gattung Filago L. s. l. (Compositae-Inuleae) in Der Ägäis." *Willdenowia* 6 (1): 115–38.

Zografidis, Aris, Svetlana Bancheva, and Kit Tan. 2014. "A New Yellow-Flowered Species of Centaurea (Asteraceae) from Mt Imittos, Attica, Greece." *Phytologia Balcanica* 20 (1): 57–63.

Boraginaceae

Boissier, Edmond. 1849. *Diagnoses Plantarum Orientalium Novarum.* Vol. 11. Parisiis : Typis Marci Ducloux et Cons. Via École-de-Médecine, 80. https://www.biodiversitylibrary.org/bibliography/72074.

Greuter, Werner. 1981. "Med-Checklist Notulae, 3." *Willdenowia* 11 (1): 23–43.

Rechinger, K. H. 1971. "Lithospermum goulandriorum Rech. f., eine neue, bemerkenswerte Art der griechischen Flora." *Botaniska notiser* 124:355–58.

Rechinger, Von K H. 1965. "Zur Kenntnis der europäischen Arten der Gattung Alkanna - About the European species of the genus Alkanna." *Ann. Naturhistor. Mus. Wien* 68 (November):191–220.

Selvi, Federico, and Massimo Bigazzi. 2003. "Revision of Genus Anchusa (Boraginaceae-Boragineae) in Greece." *Botanical Journal of the Linnean Society* 142 (4): 431–54. https://doi.org/10.1046/j.1095-8339.2003.00206.x.

Stearn, W.T. 1986. "The Greek Species of Symphytum (Boraginaceae)." *Annales Musei Goulandris* 7:175–220.

Stevanović, V., Kit Tan, and G. Iatrou. 2003. "Distribution of the Endemic Balkan Flora on Serpentine I. – Obligate Serpentine Endemics." *Plant Systematics and Evolution* 242 (1): 149–70. https://doi.org/10.1007/s00606-003-0044-8.

Teppner, Herwig. 1988a. "Onosma Epiroticum Spec. Nova (Boraginaceae) Aus Griechenland." *Phyton (Horn, Austria)* 28 (1): 51–54.

———. 1988b. "Onosma Stridii Spec. Nova (Boraginaceae) Aus Griechenland." *Phyton (Horn, Austria)* 28 (2): 271–75.

Teppner, Herwig, and Rainer Karl. 2013. "Onosma Pseudoeuboica Spec. Nova, O. Euboica and Notes on O. Heterophylla (Boraginaceae-Lithospermeae) from Central Greece." *Phyton* 53 (2): 185–220.

Brassicaceae

Al-Shehbaz, Ihsan A. 2014. "A Synopsis of the Genus Noccaea (Coluteocarpeae, Brassicaceae)." *Harvard Papers in Botany* 19 (1): 25–51. https://doi.org/10.3100/hpib.v19iss1.2014.n3.

Ball, P. W. 1963. "A Review of Malcolmia Maritima and Allied Species." *Repertorium Novarum Specierum Regni Vegetabilis* 68 (3): 179–86. https://doi.org/10.1002/fedr.19630680306.

Barbey, W. 1885. "Additions à La Flore de Carpathos et de Lycie." *Bulletin de La Société Vaudoise Des Sciences Naturelles* 21 (93): 219. https://doi.org/10.5169/seals-260543.

Boissier, E. 1896. "Séance Du 12 Juin 1896. Quelques Notes Sur La Flore d'Orient." *Bulletin de La Société Botanique*

de France 43:1–863. https://www.biodiversitylibrary.org/item/8665.
Boissier, Edmond. 1849. *Diagnoses Plantarum Orientalium Novarum, Series 1, Vol. 2, N.º 8-13*. http://archive.org/details/e.-boissier-diagnoses-plantarum-orientalium-novarum-series-secunda.-n.o-8-13-1842-1854.
———. 1854. *Diagnoses plantarum novarum praesertim Orientalium nonnullis Europaeis Boreali-Africanisque additis*. Vol. 1. 2. Soc. Phys. Genev. Sodali. https://bibdigital.rjb.csic.es/records/item/10731-diagnoses-plantarum-orientalium-novarum-series-secunda-n-ordm-4.
Burtt, B. L. 1951. "The Genus Ricotia." *Kew Bulletin* 6 (1): 123–32. https://doi.org/10.2307/4120293.
Cecchi, Lorenzo, Stanislav Španiel, Elisabetta Bianchi, Andrea Coppi, Cristina Gonnelli, and Federico Selvi. 2020. "Odontarrhena Stridii (Brassicaceae), a New Nickel-Hyperaccumulating Species from Mainland Greece." *Plant Systematics and Evolution* 306 (4): 69. https://doi.org/10.1007/s00606-020-01687-3.
Dudley, T. R. 1964. "Studies in Alyssum: Near Eastern Representatives and Their Allies, I." *Journal of the Arnold Arboretum.* 45 (1): 57–100. https://doi.org/10.5962/p.185679.
Edmond Boissier. 1843. *Diagnoses Plantarum Orientalium Novarum, Series 1, Vol. 1, No. 1 - 7.* http://archive.org/details/e.-boissier-diagnoses-plantarum-orientalium-novarum-series-1-vol.-1-no.-1-7-1843-1846.
Greuter, Werner, and Thomas Raus. 1985. "Med-Checklist Notulae, 11." *Willdenowia* 15 (1): 61–84.
Grisebach, A. (August). 1843. *Spicelegium florae rumelicae et bithynicae exhibens synopsin plantarum quas aest. 1839 legit*. Brunsvigae, prostat apud Fridericum Vieweg et filium. http://archive.org/details/bub_gb_9RxDAAAAYAAJ.
Halácsy, E. von. 1910. "Aufzählung Der von Dr. B. Tuntas Auf Der Insel Scyros Der Nördlichen Sporaden Im Juni 1908 Gesammelten Arten." *Oesterreichische Botanische Zeitschrift* 60:115.
Katsakiori, Maria. 2015. "Κείμενα έκθεσης του Κέντρου Πληροφόρησης` Επισκεπτών Εθνικού Δρυμού Ολύμπου στην αγγλική γλώσσα - Exhibition texts of the Olympus National Forest Visitor Information Center in English." National Park of Mount Olympus, Greece: Φορεας Διαχειρισης Εθνικου Δρυμου Ολυμπου. http://repository.biodiversity-info.gr/bitstream/11340/1933/1/1680.pdf.
Löve, Áskell. 1982. "IOPB Chromosome Number Reports LXXV." *Taxon* 31 (2): 342–68.
Meyer, Friedrich Karl. 1973a. "Conspectus der „Thlaspi"-Arten Europas, Afrikas und Vorderasiens." *Feddes Repertorium* 84 (5–6): 449–69. https://doi.org/10.1002/fedr.19730840503.
———. 1973b. "Conspectus der „Thlaspi"-Arten Europas, Afrikas und Vorderasiens." *Feddes Repertorium* 84 (5–6): 449–69. https://doi.org/10.1002/fedr.19730840503.
———. 1986. "Eine Neue Noccaea-Art (Cruciferae) von Kreta - A New Species of Noccaea (Cruciferae) from Crete." *Willdenowia* 15 (2): 389–91.
———. 2006. "Kritische Revision Der 'Thlaspi'-Arten Europas, Afrikas Und Vorderasiens: Spezieller Teil. VIII. Raparia F.K. Mer." *Haussknechtia* 11:195.
Muhammed, Jotyar Jassim. 2017. "Systematic and Genomic Studies in the Genus Aubrieta (Brassicaceae)." PhD Thesis, Leicester, UK: University of Leicester.
Nyarady, Erasmus Iuliu. 1927. "Studiu preliminar asupra unor specii de Alyssum din sectia Odontarrhena - Vorstudium über einige Arten der Sektio Odontarrhena der Gattung Alyssum." *Buletinul Grădinii botanice şi al Muzeului botanic de la Universitatea din Cluj = Bulletin du Jardin et du Musee botaniques de l'Universite de Cluj Roumanie* VII (3–4): 97.
Orcan, Nermin, and Riza Binzet. 2006. "A New Record for the Flora of Turkey: Alyssum Idaeum Boiss. & Heldr." *Pak. J. Bot.* 38 (4): 931–33.
Papanicolaou, Kostas. 1983. "Isatis Vermia Sp.Nov. from North Central Greece Materials for the Mountain Flora of Greece, 14." *Nordic Journal of Botany* 2 (6): 553–56. https://doi.org/10.1111/j.1756-1051.1983.tb01048.x.
Pasta, S., A. Perez-Graber, L. Fazan, and B De Montmollin. 2017. "The Top 50 Mediterranean Island Plants, Update 2017." Neufchâtel (Switzerland): IUCN/SSC/Mediterranean Plant Specialist Group. https://top50.iucn-mpsg.org/book.
Perný, Marián, Andreas Tribsch, and Mincho E. Anchev. 2004. "Infraspecific Differentiation in the Balkan diploidCardamine Acris (Brassicaceae): Molecular and Morphological Evidence." *Folia Geobotanica* 39 (4): 405–29. https://doi.org/10.1007/BF02803211.
Polatschek, Adolf. 1982. "Erysimum Canum Und E. Hayekii (Brassicaceae) / Erysimum Canum and E. Hayekii (Brassicaceae)." *Plant Systematics and Evolution* 140 (4): 321–23.
Rechinger, K. H. 1929. "Beitrag Zur Kenntnis Der Flora Der Ägäischen Inseln Und Ost-Griechenlands." *Annalen Des Naturhistorischen Museums in Wien* 43:269–340.
Runemark, H. 1963. "Studies in the Aegean Flora, V. Iberis arbuscula sp. nov." *Botaniska notiser* 116 (Fasc. 2): 323–26.
Šlenker, Marek, Marián Perný, Judita Zozomová-Lihová, and Karol Marhold. 2021. "Taxonomic Position and Circumscription of Cardamine Barbaraeoides (Brassicaceae), a Systematically Challenging Taxon from the Balkan Peninsula." *Phytotaxa* 502 (2): 111–32. https://doi.org/10.11646/phytotaxa.502.2.1.
Sweet, Robert, and Robert Sweet. 1826. *Sweet's Hortus Britannicus : Or a Catalogue of Plants Cultivated in the Gardens of Great Britain, Arranged in Natural Orders*. Vol. pt.2 (1826). London: J. Ridgway. https://doi.org/10.5962/bhl.title.43792.
Tan, Kit, Konstantinos Giannopoulos, and Gert Vold. 2013. "Cardamine Calliphaea Sp. Nov. (Brassicaceae) from Southwestern Greece." *Nordic Journal of Botany* 31 (3): 282–85. https://doi.org/10.1111/j.1756-1051.2012.00045.x.
Trigas, Panayiotis, and Gregoris Iatrou. 2006. "The Local Endemic Flora of Evvia (W Aegean, Greece)." *Willdenowia* 36 (1): 257–70. https://doi.org/10.3372/wi.36.36121.

Campanulaceae

Aldén, Björn. 1976. “Floristic Reports from the High Mountains of Pindhos, Greece.” *Botaniska Notiser* 129 (3): 297–322.

Beauverd, G., and Sophia-P. Topali. 1937. “Plantae Novae Graecae.” *Candollea* 7:266.

Beddome, Col. R.H. 1907. “An Annotated List of the Species of Campanula.” In *Journal of the Royal Horticultural Society of London*, new ser.:v.32 (1907):1–674. London: Printed for the Royal Horticultural Society by Spottiswoode & Co. https://www.biodiversitylibrary.org/item/165148.

Boissier, Edmond. 1844. *Diagnosis Plantarum Orientalium Novarum*. Vol. 4. https://bibdigital.rjb.csic.es.

Candolle, Alphonse de. 1830. *Monographie des campanulées*. Paris, Veuve Desray. http://archive.org/details/monographiedesc00candgoog.

Carlström, Annette. 1986. “A Revision of the Campanula Drabifolia Complex (Campanulaceae).” *Willdenowia* 15 (2): 375–87.

Christodoulou, Charalambos S, Ralf Hand, and Kontantinos Iosif. 2020. “Solenopsis Annua Comb. Nov., a New Taxon for Cyprus.” *Cypricola* 17.

Contandriopoulos, J., P. Quézel, and J. Zaffran. 1973. “A Propos Des Campanules Du Groupe Aizoon En Grèce Méridionale et En Crète.” *Bulletin de La Société Botanique de France* 120 (7–8): 331–40.

Degen, Arpad v. 1891. “Ergebnisse Einer Botanischen Reise Nach Der Insel Samothrake. (Schluss.).” *Österreichische Botanische Zeitschrift* 41 (10): 329–38.

Greuter, Werner, Ursula Matthäs, and Horst Risse. 1984. “Additions to the Flora of Crete, 1973-1983: I.” *Willdenowia* 14 (1): 27–36.

Greuter, Werner, and K. H. Rechinger. 1967a. “Flora der Insel Kythera gleichzeitig Beginn einer nomenklatorischen Überprüfung der grichischen Gefässpflanzenarten.” *Boissiera* 13:134.

Greuter, Werner, and Karl Heinz Rechinger. 1967b. “Chloris Kythereia.” *Boissiera* 13:133.

Grisebach, A. (August). 1843. *Spicelegium florae rumelicae et bithynicae exhibens synopsin plantarum quas aest. 1839 legit*. Vol. 1. Brunsvigae, prostat apud Fridericum Vieweg et filium. http://archive.org/details/bub_gb_9RxDAAAAYAAJ.

Halacsy, E. von. 1895. “Beitrage Zur Flora von Griechenland.” *Oesterreichische Botanische Zeitschrift* 45:461.

Halácsy, E. von. 1896. “Beitrag Zur Flora von Griechenland. (Schluss.).” *Österreichische Botanische Zeitschrift* 46 (1): 11–19.

Halácsy, Eugen v., and Eugen v Halácsy. 1902. *Conspectus Florae Graecae*. Vol. v.2. Lipsiae: Sumptibus Guilelmi Engelmann. https://doi.org/10.5962/bhl.title.9919.

Hartvig, Per. 1998. “Campanula Pangea, a New Species of C. Sect. Involucratae from Mt Pangeon, NE Greece.” *Willdenowia* 28 (1/2): 65–68.

Lamarck, Jean Baptiste Pierre Antoine de Monet de, and Jean Louis Marie Poiret. 1783. *Encyclopédie méthodique botanique, ou par ordre de matières*. Paris,Lie?ge : Panckoucke;Plomteux,. http://archive.org/details/mobot31753000215175.

Liveri, Eleni, Pepy Bareka, and Georgia Kamari. 2020. “Karyosystematic Study of Some Taxa from Campanula Section Quinqueloculares (Campanulaceae). I.” *Flora Mediterranea* 30. https://doi.org/10.7320/FlMedit30.440.

Persoon, C. H., and C. H. Persoon. 1805. *Synopsis Plantarum,Seu Enchiridium Botanicum, Complectens Enumerationem Systematicam Specierum Hucusque Cognitarum*. Vol. v.1. Parisiis Lutetiorum: C.F. Cramerum. https://doi.org/10.5962/bhl.title.638.

Phitos, D. 1964a. “Beiträge Zur Flora Hellenica.” *Verhandlungen Der Zoologisch-Botanischen Gesellschaft in Wien* 103–104:223–30.

———. 1964b. “Trilokuläre Campanula-Arten Der Ägäis.” *Österreichische Botanische Zeitschrift* 111 (2/3): 208–30.

———. 1965. “Die Quinquelokulären Campanula-Arten.” *Österreichische Botanische Zeitschrift* 112 (4): 449–98.

Phitos, Dimitrios. 1963. “Eine Neue Art Der Gattung Campanula Aus Der Ägäis.” *Mitteilungen Der Botanischen Staatssammlung München* v.5 (1963-1965):121–24.

———. 2021. “Reinstating Campanula Nisyria as a Distinct Species of Sect. Quinqueloculares (Campanulaceae).” *Flora Mediterranea* 31:101–7. https://doi.org/10.7320/FlMedit31.101.

Podlech, Dieter. 1965. “Revision Der Europäischen Und Nordafrikanischen Vertreter Der Subsect. Heterophylla (Wit.) Fed. Der Gattung Campanula L.” *Feddes Repertorium* 71 (1–3): 50–187. https://doi.org/10.1002/fedr.19650710103.

Sibthorp, Johannes, and Jacobus Edvardus Smith. 1806. *Flora Graecae Prodromus*. Vol. 1.

Siljak-Yakovlev, Sonja, Perla Farhat, Nicolas Valentin, Pepy Bareka, and Georgia Kamari. 2019. “New Estimates of Nuclear DNA Amount for 25 Taxa from Kefallinia Island,” 23.

Tan, Kit, and Burkhard Biel. 2011. “Nomenclatural Note.” *Phytologia Balcanica* 17 (2): 265.

Trigas, Panayiotis, and Gregoris Iatrou. 2006. “The Local Endemic Flora of Evvia (W Aegean, Greece).” *Willdenowia* 36 (1): 257–70. https://doi.org/10.3372/wi.36.36121.

Turrill, W. B. 1955. “Some New Plants from Greece.” *Kew Bulletin* 10 (3): 353–57. https://doi.org/10.2307/4109221.

Vierhapper, Friedrich, and Karl Heinz Rechinger. 1935. “Bearbeitung Der von Ignaz Dörfler Im Jahre 1904 Auf Kreta Gesammelten Blüten- Und Farnpflanzen. (Schluß).” *Österreichische Botanische Zeitschrift* 84 (3): 161–97.

Vladimirov, Compiled Vladimir, Mehmet Aybeke, and Kit Tan. 2021. “New Floristic Records in the Balkans: 46.”

Von Raab-Straube, Eckhard, and Thomas Raus. 2014. “Notulae Ad Floram Euro-Mediterraneam Pertinentes No. 32: Euro+Med-Checklist Notulae, 3.” *Willdenowia* 44 (2): 287–99.

Caryophyllaceae

Boissier, Edmond. 1842. *Diagnoses plantarum orientalium novarum: ser. 1, fasc. 8-13*. B. Herrmann.

———. 1859. *Diagnoses plantarum Orientalium novarum.* Vol. 6. 2. https://bibdigital.rjb.csic.es.

———. 1888. *Flora Orientalis sive Enumeratio Plantarum in Oriente a Graecia et Aegypto ad Indiae Fines*. Vol. Supplementum. https://bibdigital.rjb.csic.es.

Bornmüller, J. 1908. “Florula Lydiae.” *Mitteilungen Des Thüringischen Botanischen Vereins. n. F* 24:20.

British Museum (Natural History). 1963. *Bulletin of the British Museum (Natural History) Botany*. Vol. v.3 (1963-1965). London: The Museum. https://www.biodiversitylibrary.org/item/19380.

Chater, A. O. 1988. "Flora Europaea: Notulae Systematicae Ad Floram Europaeam Spectantes. Series 2. No. 2." *Botanical Journal of the Linnean Society* 97 (4): 335–55. https://doi.org/10.1111/j.1095-8339.1988.tb01065.x.

Chowdhuri, P.K. 1957. "Studies in the Genus Silene." *Notes from the Royal Botanic Garden, Edinburgh* 22 (3): 221–78.

Constantinidis, Th. 1999. "Dianthus Haematocalyx Subsp. Phitosianus (Caryophyllaceae), a New Serpentine Endemic from Greece (Engl.)." *Phyton (Horn, Austria)* 39 (2): 277–92.

Edmond Boissier. 1849. *Diagnoses Plantarum Orientalium Novarum, Series 1, Vol. 2, N.º 8-13*. Vol. 2, N°8-13. 1. http://archive.org/details/e.-boissier-diagnoses-plantarum-orientalium-novarum-series-secunda.-n.o-8-13-1842-1854.

———. 1854. *Diagnoses Plantarum Orientalium Novarum, Series 2, Vol. 3, N.º 1 - 6*. Vol. 3. http://archive.org/details/e.-boissier-diagnoses-plantarum-orientalium-novarum-series-secunda.-n.o-1-1854.

Fenzl, Eduard. 1842. "CL. EDM. BOISSIER S.P.D." In *Pugillus plantarum novarum Syriæ et Tauri occidentalis primus*, 8. Apud Fridericum Beck.

Greuter, Werner. 1995. "Studies in Greek Caryophylloideae: Agrostemma, Silene, and Vaccaria." *Willdenowia* 25:105–42.

Greuter, Werner, Niels Böhling, and Ralf Jahn. 2002. "The Cerastium Scaposum Group (Caryophyllaceae): Three Annual Taxa Endemic to Crete (Greece), Two of Them New." *Willdenowia* 32 (1): 45–54. https://doi.org/10.3372/wi.32.32103.

Greuter, Werner, and Thomas Raus. 1982. "Med-Checklist Notulae, 6." *Willdenowia* 12 (2): 190.

———. 1984. "Med-Checklist Notulae, 9." *Willdenowia* 14 (1): 45+47.

———. 1987. "Med-Checklist Notulae, 14." *Willdenowia* 16 (2): 439–52.

Halácsy, Eugen v., and Eugen v Halácsy. 1901. *Conspectus Florae Graecae*. Vol. v.1. Lipsiae: Sumptibus Guilelmi Engelmann. https://doi.org/10.5962/bhl.title.9919.

Haussknecht, C. 1893. "Symbolae Ad Floram Graecam. Aufzählung Der Im Sommer 1885 in Griechenland Gesammelten Pflanzen." *Mittheilungen Des Thüringischen Botanischen Vereins* 5:41–126.

Heldreich, Theodor von. 1882. *Flore de l'île de Céphalonie, ou, Catalogue des plantes qui croissent naturellement et se cultivent le plus fréquemment dans cette île*. Lausanne, G. Bridel. http://archive.org/details/floredelledec1882held.

Oxelman, Bengt. 1995. "A Revision of the Silene Sedoides-Group (Caryophyllaceae)." *Willdenowia* 25 (1): 143–69.

Papanastasis, V P, S Kyriakakis, and G Kazakis. 2002. "Plant Diversity in Relation to Overgrazing and Burning in Mountain Mediterranean Ecosystems," no. 2.

Phitos, Dimitrios. 1981. "Το Γενος Bolanthus (Caryophyllaceae) Στην Ελλαδα." *Botanika Chronika* 1 (1): 35–45.

———. 1982. "Silene Greuteri (Caryophyllaceae), Species Nova." *Botanika Chronika* 2 (1): 53–54.

Rechinger, K. H. 1939. "Repertorium Europaeum et Mediterraneum (Nr.3738, V. Band) - VII. (LXXVI) K.H. Rechinger fil.: Plantae novae Aegaeae II)." *Repertorium specierum novarum regni vegetabilis [...] Band XLVII (1939)* 47:57.

Società toscana di orticulture, Florence. 1876. *Atti del Congresso internazionale botanico tenuto in Firenze nel mese di ...* http://archive.org/details/attidelcongress02unkngoog.

Stojanoff, N. 1938. "Botanische Studien Auf Dem Thessalischen Olymp." *Годишник На Софийския Университет. Физико-Математически Факултет. Книга 3 - Естествена История* Том 34-Университетска библиотека "Св. Климент Охридски." https://digital.libsu.uni-sofia.bg/bg/v/36331?q%3Amain.publishing.year=1938.

Tan, Kit, and Arne Strid. n.d. "Paronychia Manfrediana (Caryophyllaceae), a New Species from Northeast Greece."

Trigas, Panayiotis, and Gregoris Iatroú. 2003. "A New Species of Minuartia (Caryophyllaceae) from the Island of Evvia (Greece)." *Nordic Journal of Botany* 23 (4): 415–25. https://doi.org/10.1111/j.1756-1051.2003.tb00414.x.

Vladimirov, Compiled Vladimir, Mehmet Aybeke, and Kit Tan. 2020. "New Floristic Records in the Balkans: 41." *Phytologia Balcanica* 26 (1): 163–79.

Colchicaceae

Persson, Karin. 1999. "New and Revised Species ofColchicum (Colchicaceae) from the Balkan Peninsula." *Plant Systematics and Evolution* 217 (1): 55–80. https://doi.org/10.1007/BF00984922.

Tsiftsis, Spyros, Thomas Giannakis, Sampson Panajiotidis, Eleni Eleftheriadou, and Konstantinos Theodoropoulos. 2021. "Colchicum Tulakii (Colchicaceae), a New Species from Central Macedonia, Northeastern Greece." *Nordic Journal of Botany* 39 (6). https://doi.org/10.1111/njb.03207.

Convolvulaceae

Greuter, Werner. 1967. "Beiträge Zur Flora Der Südägäis 8-9." *Bauhinia* 3:250–52.

Crassulaceae

Carlström, Annette. 1984. "New Species of Alyssum, Consolida, Origanum and Umbilicus from the SE Aegean Sea." *Willdenowia* 14 (1): 15–26.

Hart, H. 't. 1984. "Sedum Apoleipon, a New Species of the Sedum Acre Group (Crassulaceae) from Central Greece." *Willdenowia* 13 (2): 309–19.

Vladimirov, Compiled Vladimir. 2016. "New Floristic Records in the Balkans: 29."

Cyperaceae

Greuter, Werner, Ursula Matthäs, and Horst Risse. 1985. "Additions to the Flora of Crete, 1973-1983 (1984) - III." *Willdenowia* 15 (1): 25.

Dipsacaceae

Boissier, Edmond, Edmond Boissier, and R. Buser. 1875. *Flora Orientalis : Sive, Enumeratio Plantarum in Oriente a Graecia et Aegypto Ad Indiae Fines Hucusque Observatarum*. Vol. 3. Basileae: H. Georg. https://doi.org/10.5962/bhl.title.20323.

Contandriopoulos, J., and P. Quézel. 1973. "Deux Dipsacées Nouvelles de Grèce." *Biologia Gallo-Hellenica* 5 (1): 131–38.

Greuter, W., U. Matthäs, and H. Risse. 1985. "Additions to the Flora of Crete, 1973-1983 (1984) - III." *Willdenowia* 15 (1): 74–76.

Greuter, Werner. 1967. "Contributiones Floristicae Austro-Aegaeae 10-12." *Candollea: Journal International de Botanique Systématique = International Journal of Systematic Botany* 22 (2): 233. https://doi.org/10.5169/seals-880396.

Perdetzoglou, Dimitris K., and Kit Tan. 1995. "A New Species of Cephalaria (Dipsacaceae) from Northern Greece." *Annales Botanici Fennici* 32 (4): 227–28.

Strid, Arne. 1981. "New Species of Cephalaria (Dipsacaceae) and Stipa (Gramineae) from the Greek Mountains." *Willdenowia* 11 (2): 301–5.

Euphorbiaceae

Greuter, Werner. 1965. "Beiträge zur Fora der Südägäis 1-7." *Candollea* 20:170.

Strid, Arne, Bengt Bentzer, Roland von Bothmer, Lennart Engstrand, and Mats Gustafsson. 1989. "Euphorbia Sultan-Hassei (Euphorbiaceae), a New Species from SW Crete." *Willdenowia* 19 (1): 63.

Vladimirov, Vladimir, Feruzan Dane, and Kit Tan. 2013. "New Floristic Records in the Balkans: 23."

Fabaceae

Akkemik, Ünal. 2018. *Türkiye'nin Doğal-Egzotik Ağaç ve Çalıları (Gymnospermler, Angiospermler)*. İ.Ü.Orman Fakültesi Orman Botaniği Anabilim Dalı, Bahçeköy - İstanbul. https://acikerisim.bartin.edu.tr/bitstream/handle/11772/2409/T%C3%BCrkiye%E2%80%99nin%20Do%C4%9Fal-Egzotik%20A%C4%9Fa%C3%A7%20ve%20%C3%87al%C4%B1lar%C4%B1%20Genista%20L.%20%28Borcak%29.pdf?sequence=1&isAllowed=y.

Böhling, Niels, Werner Greuter, and Thomas Raus. 2000. "Trifolium Phitosianum (Leguminosae), a New Annual Clover Species from Crete." *Botanika Chronika* 13:37–44.

Boissier, Edmond, Edmond Boissier, and R. Buser. 1875. *Flora Orientalis : Sive, Enumeratio Plantarum in Oriente a Graecia et Aegypto Ad Indiae Fines Hucusque Observatarum*. Vol. 3. Basileae: H. Georg. https://doi.org/10.5962/bhl.title.20323.

Bornmüller, J.F.N. 1921. "Ein Beitrag zur Kenntnis der Gattung Asyneuma Griseb." *Beihefte zum Botanischen Centralblatt* 37 (2): 341.

Brullo, Salvatore, and Gianpietro Giusso del Galdo. 2001. "Astracantha Dolinicola (Fabaceae): A New Species from Crete." *Nordic Journal of Botany* 21 (5): 475–80. https://doi.org/10.1111/j.1756-1051.2001.tb00799.x.

Brullo, Salvatore, and Gian Pietro Giusso del Galdo. 2003. "Taxonomical Considerations of Astragalus Creticus Lam. Group (Fabaceae) from Crete." *Israel Journal of Plant Sciences* 51 (4): 307–13. https://doi.org/10.1560/P555-MNB1-EXET-5UYK.

Bunge, Al. 1868. "Generis Astragali Species Gerontogeae, Pars Prior: Claves Diagnosticae." *Mémoires de l'Académie Impériale Des Sciences de St.-Pétersbourg. 7e Série* Ser.7 : T.11 (N°16): 107.

Degen, Árpád von, and Árpád von Degen. 1911. *Magyar Botanikai Lapok = Ungarische Botanische Blätter*. Vol. v.10-11 (1911-1912). Budepest: Müller Karoly Könyvnyomdaja aja. https://www.biodiversitylibrary.org/item/201840.

Gibelli, G., and S. Belli. 1889. "Rivista Critica Delle Specie Di Trifolium Italiani Della Sezione Chronosemium Ser. in DC. Prod. II, p.204." *Malpighia* v.3 (1889) (3): 228.

Greuter, Werner. 1965. "Beiträge zur Fora der Südägäis 1-7." *Candollea* 20:170.

———. 1979. "The Flora and Phytogeography of Kastellorizo (Dhodhekanisos, Greece). 1. An Annotated Catalogue of the Vascular Plant Taxa." *Willdenowia* 8 (3): 531–611.

Greuter, Werner, Regina Pleger, and Thomas Raus. 1983. "The Vascular Flora of the Karpathos Island Group (Dodecanesos, Greece). A Preliminary Checklist." *Willdenowia* 13 (1): 43–78.

Lamarck, Jean-Baptiste-Pierre-Antoine de Monet de, Jean-Baptiste-Pierre-Antoine de Monet de Lamarck, and Jean-Louis-Marie Poiret. 1786. *Encyclopédie Méthodique. Botanique*. Vol. t.2 (1786). Paris,Liège: Panckoucke;Plomteux. https://doi.org/10.5962/bhl.title.824.

Lassen, P. 1996. "Trifolium Andricum (Fabaceae), a New Species from Greece." *Annalen Des Naturhistorischen Museums in Wien. Serie B Für Botanik Und Zoologie* 98:301–3.

Sirjaev, G. 1932. "Generis Ononis L. revisio critica." *Beihefte zum Botanischen Centralblatt. Zweite Abteilung: Systematik, Pflanzengeographie, angewandte Botanik etc. Band XLIX* 49:517.

Sobrino Vesperinas, Eduardo, A. Hervella, José Luis Ceresuela Soria, A. Barbado, A. Viviani, F. De Andrés, and José Luis Tenorio Pasamón. 2000. "Morfología y Taxonomía de la Sección Dendrotelis del género Medicado (Fabaceae)." *Portugaliae Acta Biologica* 19 (1): 225–37.

Vladimirov, Compiled Vladimir, Mehmet Aybeke, and Kit Tan. 2020. "New Floristic Records in the Balkans: 43." *Phytologia Balcanica* 26 (3): 537–72.

———. 2021. "New Floristic Records in the Balkans: 46." *Phytologia Balcanica* 27 (3): 373–411.

Vladimirov, Compiled Vladimir, Feruzan Dane, and Kit Tan. 2016. "New Floristic Records in the Balkans: 30." *Phytologia Balcanica* 22 (2): 259–92.

Fumariaceae

Boissier, Edmond. 1859. *Diagnoses plantarum Orientalium novarum.* Vol. 6. 2. https://bibdigital.rjb.csic.es.

Geraniaceae

Strid, Arne, and Kit Tan. 2003. "New Species of Erodium (Geraniaceae), Onosma (Boraginaceae) and Centaurea (Asteraceae) from Northern Greece." *Phytologia Balcanica* 9 (3): 471–78.

Hyacinthaceae

Bareka, P., Turland ,N.J., and G. and Kamari. 2015. "Bellevalia Juliana (Asparagaceae), a New Hexaploid Species from E Kriti (Greece)." *Plant Biosystems - An International Journal Dealing with All Aspects of Plant Biology* 149 (4): 703–9. https://doi.org/10.1080/11263504.2015.1057258.

Greuter, Werner, and Thomas Raus. 2012. "Med-Checklist Notulae, 31." *Willdenowia* 42 (2): 287–95.

Kypriotakis, Z, and D Tzanoudakis. 1998. "A New Species of Bellevalia from Eastern Crete and Its Confusion with Muscari Macrocarpum Sweet." *Botanica Helvetica* 109 (1): 85.

Kypriotakis, Zacharias, Eleftheria Antaloudaki, and Dimitris Tzanoudakis. 2018. "Ornithogalum Insulare (Hyacinthaceae): A New Species from the Cretan Area (S. Aegean, Greece)." *Botanica Serbica* 42 (1): 117–22.

Linné, Carl von, Joseph August Schultes, and Julius Hermann Schultes. 1829. *Caroli a Linné ... Systema Vegetabilium :Secundum Classes, Ordines, Genera, Species. Cum Characteribus, Differentiis et Synonymiis*. Vol. v.7 pt.1. Stuttgardtiae: Sumtibus J.G. Cottae. https://doi.org/10.5962/bhl.title.825.

Speta, F. 1998. "Die Scilla-Arten (Hyacinthaceae) der griechischen Inseln Kreta und Karpathos." *Linzer Biologische Beiträge* 30 (1): 431–37.

Speta, Franz. 1971. "Beitrag Zur Systematik von Scilla L. Subgen. Scilla (Inklusive Chionodoxa Boiss)." *Österreichische Botanische Zeitschrift* 119 (1–3): 6–18.

———. 1980. "Die Frühjahrsblühenden Scilla-Arten des Östlichen Mittelmeerraumes." *Naturk. Jahrb. Stadt Linz* 25:19–198.

———. 1991. "Zwei Neue Scilla-Arten (Hyacinthaceae) Aus Dem Östlichen Mittelmeerraum." *Phyton (Horn, Austria)* 31 (1): 27–33.

———. 2000. "Ornithogalum Sphaerolobum Und Seine Doppelgänger." *Preslia* 72 (2–4): 369–98.

———. 2006. "Die Gattung Loncomelos Raf. (Hyacinthaceae-Ornithogaloideae), Vorgestellt Anhand Dreier Neuer Arten." *Phyton* 46 (1): 1–25.

Turrill, W. B. 1940. "On the Flora of the Nearer East: XXII. New Records and New Species from Greece and the Greek Islands." *Bulletin of Miscellaneous Information (Royal Botanic Gardens, Kew)* 1940 (6): 262–66. https://doi.org/10.2307/4111593.

Von Raab-Straube, Eckhard, and Thomas Raus. 2017. "Euro+Med-Checklist Notulae, 8." *Willdenowia* 47 (3): 293–309.

Zahariadi, Constantin A. 1977. "Cinq Espèces Nouvelles Du Genre Ornithogalum (Liliaceae) Trouvées En Grèce." *Annales Musei Goulandris* 3:51–75.

Hypericaceae

Barbey, W. 1885. "Additions à La Flore de Carpathos et de Lycie." *Bulletin de La Société Vaudoise Des Sciences Naturelles* 21 (93): 219. https://doi.org/10.5169/seals-260543.

Gandoger, Michel. 1916. *Flora cretica /auctore Michaele Gandoger.* Parisiis : A. Hermann et fil,. http://archive.org/details/mobot31753000530409.

Hagemann, Isolde. 1987. "On Subspecific Taxa ofHypericum Empetrifolium (Hypericaceae) from Crete." *Plant Systematics and Evolution* 155 (1): 165–87. https://doi.org/10.1007/BF00936297.

Heywood, V. H. 1967. "Flora Europaea Notulae Systematicae Ad Floram Europaeam Spectantes." *Feddes Repertorium* 74 (1–2): 1–37. https://doi.org/10.1002/fedr.19670740102.

Penzig, O., O. Penzig, Antonino Borzi, and Romualdo Pirotta. 1895. *Malpighia :Rassegna Mensuale Di Botanica*. Vol. v.9 (1895). Messina: g. Capra & co. https://www.biodiversitylibrary.org/item/107904.

Tan, Kit, Gregoris Iatroú, Gert Vold, and Arne Strid. 2010. "Hypericum Boehlingraabei (Hypericaceae), a New Species from the Northern Peloponnese (Greece)."

Trigas, Panayiotis. 2018. "A New Hypericum (Sect. Drosocarpium, Hypericaceae) from the Cyclades Islands (Greece)." *Nordic Journal of Botany* 36 (11): e02205. https://doi.org/10.1111/njb.02205.

Zuerich, ETH-Bibliothek. 1965. "Beiträge zur Flora der Südägäis 1-7." *CANDOLLEA* 20 (215). https://www.e-periodica.ch//digbib/volumes?UID=can-002.

Iridaceae

Bulletin Des Sciences Naturelles et de Géologie. 1831. Vol. t.24-25 (1831). Paris. https://www.biodiversitylibrary.org/item/25919.

Harpke, Dörte, Lorenzo Peruzzi, Helmut Kerndorff, Theophanis Karamplianis, Theophanis Constantinidis, Vladimir Randelovic, Novica Randelovic, Marina Juskovic, Erich Pasche, and Frank R. Blattner. 2014. "Phylogeny, Geographic Distribution, and New Taxonomic Circumscription of the Crocus Reticulatus Species Group (Iridaceae)." TURKISH JOURNAL OF BOTANY 38 (6): 1182–98.

Papanicolaou, Kostas, and Evgenia Zacharof. 1980. "Crocus in Greece: new taxa and chromosome numbers." Botaniska notiser 133 (2): 161.

Ruksans, Janis. 2013. "Seven New Crocuses Described." The Alpine Gardener 81 (2): 188–93.

———. 2016. "Some New Crocus Species." International Rock Gardener 76 (April):16–43.

———. 2017. "Two New Crocus Species Allied to Crocus Nubigena from the Eastern Aegean Islands." International Rock Gardener, no. 90 (June). https://www.srgc.net/documents/irg/2017Jun211498039508IRG_90_June.pdf.

———. 2021a. "Three New Crocus (Iridaceae) Species from Turkey and the East Aegean Islands." International Rock Gardener 144.

———. 2021b. "Two New Crocus (Iridaceae) Species from Turkey and the East Aegean Islands." International Rock Gardener, no. 144 (December). https://www.srgc.org.uk/logs/logdir/2021Dec291640808762IRG144.pdf.

Rukšāns, Jānis, and Dimitri Zubov. 2023. "Four New Crocus Species (Iridaceae) from Mainland Greece and Western Turkey." International Rock Gardener, no. 162 (July). https://www.srgc.org.uk/logs/logdir/2023Jul271690480891IRG_July2023.pdf.

Vladimirov, Vladimir, Kit Tan, Aris Zografidis, and Dionysios Mermygkas. 2014. "New Floristic Records in the Balkans: 24. Report 131." Phytologia Balcanica 20 (1): 123–25.

Zubov, Dimitri. 2017. "New Crocus Species from the South-Eastern Aegean, Karpathos Island, Greece."

International Rock Gardener, no. 90 (June). *https://www.srgc.net/documents/irg/2017Jun211498039508IRG_90_June.pdf.*

Lamiaceae

Barbey, W. 1885. “Additions à La Flore de Carpathos et de Lycie.” *Bulletin de La Société Vaudoise Des Sciences Naturelles* v.21-22=no.92-95 (1885-1887):223.
Bothmer, Roland von. 1985. “Differentiation Patterns in the Scutellaria Albida Group (Lamiaceae) in the Aegean Area.” *Nordic Journal of Botany* 5 (5): 421–39.
Bräuchler, Christian, Harald Meimberg, and Günther Heubl. 2006. “New Names in Old World Clinopodium - the Transfer of the Species of Micromeria Sect. Pseudomelissa to Clinopodium.” *Taxon* 55 (4): 977–81.
Burnat, Emile. 1895. *Les Labiées des Alpes Maritimes (études monographiques sur les Labiées qui croissent spontanément dans la chaine des Alpes Maritimes et dans le département français de ce nom)*. Vol. 3. 3 vols. Georg & Co, Libraires-2diteurs. https://bibdigital.rjb.csic.es.
Burtt, B. L., and P. H. Davis. 1949. “On the Flora of the Nearer East: XXIII. Miscellaneous New Species and Records.” *Kew Bulletin* 4 (1): 97–115. https://doi.org/10.2307/4119046.
Carlström, Annette. 1984. “New Species of Alyssum, Consolida, Origanum and Umbilicus from the SE Aegean Sea.” *Willdenowia* 14 (1): 15–26.
Davis, P.H., R.R. Mill, and Kit Tan. 1988. “Addenda, Labiatae.” In *Flora of Turkey*, 10:236. University Press Edinburgh.
Formanek, Ed. 1897. “Neue Arten Aus Thessalien.” *Deutsche Botanische Monatsschrift : Organ Für Floristen, Systematiker Und Alle Freunde Der Heimischen Flora* v.15(1897):75.
Greuter, Werner, and Thomas Raus. 2012. “Med-Checklist Notulae, 31.” *Willdenowia* 42 (2): 287–95.
Halacsy, E. von. 1899. “Beiträge Zur Flora von Griechenland, Zweiter Theil.” *Verhandlungen Der Kaiserlich-Königlichen Zoologisch-Botanischen Gesellschaft in Wien* Bd.49 (1899):191.
Hartvig, Per. 1987. “A Taxonomical Revision ofThymus Sect.Teucrioides (Lamiaceae).” *Plant Systematics and Evolution* 155 (1): 197–213. https://doi.org/10.1007/BF00936299.
Radon, Johann. 1956. “Sitzung Vom 14. Juni 1956.” *Anzeiger Der Österreichischen Akademie Der Wissenschaften Mathematisch-Naturwissenschaftliche Klasse* 93 (9): 95.
Rechinger, K. H. 1934. “Dreizehn Neue Pflanzenarten Aus Griechenland.” *Magyar Botanikai Lapok* 33 (1–12): 9.
Rechinger, Karl Heinz. 1949. “Florae Aegaeae Supplementum.” *Phyton* 1:208.
Tan, Kit, Burkhard Biel, and Jerzy Zieliński. 2020. “Micromeria Zarkosii (Lamiaceae), an Unusual New Species from Naxos (Central Kiklades, Greece).” *Nordic Journal of Botany* 38 (11). https://doi.org/10.1111/njb.02975.
Tan, Kit, and Jerzy Zieliński. 2001. “Micromeria Browiczii (Labiatae), an Unusual New Species from Zakinthos (Ionian Islands, Greece).” *Polish Botanical Journal* 46 (1): 31–33.
Tümen, Gülendam, FatiH Satil, Hayri Duman, and Kemal Hüsnü Can Başer. 2000. “Two New Records for Turkey: Satureja Icarica P.H. Davis, Satureja Pilosa Velen.” *TURKISH JOURNAL OF BOTANY* 24 (3): 211–14.
Turrill, W. B. 1937. “On the Flora of the Nearer East: XVIII. New Species, New Records, and Notes.” *Bulletin of Miscellaneous Information (Royal Botanic Gardens, Kew)* 1937 (2): 79–86. https://doi.org/10.2307/4107196.
Wettstein, Richard R. von, and Erwin Janchen. 1915. *Oesterreichische Botanische Zeitschrift*. Vol. v.65 (1915). Wien: A. Skofitz. https://www.biodiversitylibrary.org/item/37243.

Liliaceae

Halácsy, Eugen v., and Eugen v Halácsy. 1904. *Conspectus Florae Graecae*. Vol. v.3. Lipsiae: Sumptibus Guilelmi Engelmann. https://doi.org/10.5962/bhl.title.9919.
Tison, Jean-Marc, Angela Peterson, Dörte Harpke, and Lorenzo Peruzzi. 2013. “Reticulate Evolution of the Critical Mediterranean Gagea Sect. Didymobulbos (Liliaceae) and Its Taxonomic Implications.” *Plant Systematics and Evolution* 299 (2): 413–38. https://doi.org/10.1007/s00606-012-0731-4.
Von Raab-Straube, Eckhard, and Thomas Raus. 2016. “Notulae Ad Floram Euro-Mediterraneam Pertinentes No. 35: Euro+Med-Checklist Notulae, 6.” *Willdenowia* 46 (3): 423–42.

Linaceae

Christodoulakis, D. 1995. “A New Subspecies of Linum Gyaricum (Linaceae) from Greece.” *Nordic Journal of Botany* 15 (2): 145–47. https://doi.org/10.1111/j.1756-1051.1995.tb00131.x.
Radon, Johann. 1956. “Sitzung Vom 14. Juni 1956.” *Anzeiger Der Österreichischen Akademie Der Wissenschaften Mathematisch-Naturwissenschaftliche Klasse* 93 (9): 96–97.
Strid, Arne, and Kit Tan. 1991. *Moutain Flora of Greece*. Vol. 1. 2 vols. Edinburgh University Press.
Vierhapper, Friedrich. 1919. “Beiträge Zur Kenntnis Der Flora Griechenlands.” *Verhandlungen Der Zoologisch-Botanischen Gesellschaft in Wien* 69:103.
Vierhapper, Friedrich, and Karl Heinz Rechinger. 1935. “Bearbeitung Der von Ignaz Dörfler Im Jahre 1904 Auf Kreta Gesammelten Blüten- Und Farnpflanzen.” *Österreichische Botanische Zeitschrift* 84 (2): 123–57.

Onagraceae

Snogerup, S. 1982. “A New Species of Epilobium (Onagraceae) from Northern Greece.” *Willdenowia* 12 (2): 227–29.

Orchidaceae

Bateman, Richard M., Attila Molnár V., and Gábor Sramkó. 2017. “*In Situ* Morphometric Survey Elucidates the Evolutionary Systematics of the Eurasian *Himantoglossum* Clade (Orchidaceae: Orchidinae).” *PeerJ* 5 (January):e2893. https://doi.org/10.7717/peerj.2893.

Baumann, Helmut, and Richard Lorenz. 2005. “Beiträge Zur Taxonomie Europäischer Und Mediterraner Orchideen.” *Jour Eur Orch* 37:939–74.

Boissier, E. 1853. *Diagnoses plantarum Orientalium novarum*. Vol. 13. https://bibdigital.rjb.csic.es.

Delforge, P. 2014. “Numéro spécial de la Section Orchidées d’Europe, Annexe 1. Nomenclature.” *Les Naturalistes Belges* 95 (Orchid. 27): 203.

Delforge, Pierre. 2007. “Coéditions En Anglais de La Troisième Édition Du ‘Guide Des Orchidées d’Europe...’ : Modifications Nomenclaturales et Rédactionnelles Ainsi Que Quelques Remarques Sur La Traduction.” *Natural. Belges* 88 (Orchid. 20): 1–17.

Heywood, V. H. 1978. “Flora Europaea: Notulae Systematicae Ad Floram Europaeam Spectantes: No. 20.” *Botanical Journal of the Linnean Society* 76 (4): 297–384. https://doi.org/10.1111/j.1095-8339.1978.tb01817.x.

Kreutz, C. A. J. 2000. “Notizen Zu Einigen Spätblühenden Orchideenarten Auf Kreta.” *Ber. Arbeitskreis. Heimi-Sche Orchid* 17:106–14.

Robatsch, K. 1990. “Beiträge Zur Kenntnis Der Europäischen Epipactis-Arten (Orchidaceae).” *Linzer Biologische Beiträge* 22 (1): 143–49.

Strid Arne, and Tan Kit. 2017. “Recent Progress In Plant Taxonomy And Floristic Studies In Greece,” October. https://doi.org/10.5281/ZENODO.1026649.

Vierhapper, Friedrich. 1919. “Beiträge Zur Kenntnis Der Flora Griechenlands.” *Verhandlungen Der Zoologisch-Botanischen Gesellschaft in Wien* 69:103.

Willing, Eckhard. 1989. “Dactylorhiza in Griechenland.” *<artikel Op Basis van de Lezing, Gehouden Op 24-10-1987 Te Amersfoort>*. https://natuurtijdschriften.nl/pub/1029566/EUR1989001001006.pdf.

Μινέτος, Παναγιώτης Μ. 2024. “Ενδιαιτήματα των σπάνιων και ενδημικών φυτών της Κεφαλονιάς και Ιθάκης,” October. http://dspace.aua.gr/xmlui/handle/10329/8328.

Papaveraceae

Dahl, Åslög E. 1989. “Taxonomic and Morphological Studies inHypecoum Sect.Hypecoum (Papaveraceae).” *Plant Systematics and Evolution* 163 (3): 227–80. https://doi.org/10.1007/BF00936517.

Plantaginaceae

Vladimirov, Vladimir, Mehmet Aybeke, and Kit Tan. 2023. “New Floristic Records in the Balkans: 51*.” *Phytologia Balcanica: International Journal of Balkan Flora and Vegetation* 29 (2): 259–310.

Plumbaginaceae

Apostolopoulos, Efstathios, and Theophanis Constantinidis. 2024. “Limonium Ophioides and L. Nichoriense (Plumbaginaceae), Two New Diploid Species from Peloponnisos, Greece.” *PHYTOTAXA* 655 (2): 159–72.

Artelari, R. 1984. “Two New Species of Limonium (Plumbaginaceae) from Zakynthos Island (Greece).” *Mitteilungen Der Botanischen Staatssammlung München* 20:429–40.

———. 1989. “Biosystematic Study of the Genus Limonium (Plumbaginaceae) in the Aegean Area (Greece). I. Some Limonium Species from the Kikladhes Islands.” *Willdenowia* 18 (2): 399–408.

Brullo, S., and R. Guarino. 2000. “Contribution to the Knowledge of Flora and Vegetation of Khrisi Islet (Crete, SE Mediterranean Sea).” *Flora Mediterranea* 10:265–82.

Brullo, Salvatore, and Matthias Erben. 2016. The Genus Limonium (Plumbaginaceae) in Greece.” *Phytotaxa* 240 (1): 1–212. https://doi.org/10.11646/phytotaxa.240.1.1.

Crespo, Manuel B., and Carolina Pena-Martin. 2013. “Two New Species of Limonium (Plumbaginaceae) from Rhodes Island (Eastern Aegean Area, Greece).” *Phytotaxa* 94 (2): 30–40.

Koutroumpa, Konstantina. 2024. “Limonium Artelariae (Plumbaginaceae), a New Endemic Species and Further Taxonomic and Floristic Notes on the Genus in the Island of Crete.” *Willdenowia* 54 (1): 65–79.

Papanicolaou, K., and S. Kokkini. 1982. “A New Species of Armeria (Plumbaginaceae) from Euboea, Greece.” *Willdenowia* 12 (2): 221.

Valli, Anna-Thalassini, and Rea Artelari. 2015. Limonium Korakonisicum (Plumbaginaceae), a New Species from Zakynthos Island (Ionian Islands, Greece.” *Phytotaxa* 217 (1): 63–72. https://doi.org/10.11646/phytotaxa.217.1.5.

Poaceae

Greuter, Werner, and Thomas Raus. 1998. “Med-Checklist Notulae, 17.” *Willdenowia* 28 (1/2): 171.

———. 2011. “Med-Checklist Notulae, 30.” *Willdenowia* 41 (2): 311–28.

Hayek, August. 1929. “Ein Beitrag zur Kenntnis der Vegetation und der Flora des thessalischen Olymp.” *Beiheft zum Botanischen Centralblatt* 45:307.

Heywood, V. H. 1978. “Flora Europaea: Notulae Systematicae Ad Floram Europaeam Spectantes: No. 20.” *Botanical Journal of the Linnean Society* 76 (4): 297–384. https://doi.org/10.1111/j.1095-8339.1978.tb01817.x.

J, Martinovský. n.d. “Studien Über Einige Submediterrane Federgrassippen. XXIV. Beitrag Zur Kenntnis Der Gattung Stipa” 44 (1). Accessed May 28, 2025. https://www.preslia.cz/article/10854.

Markgraf-Dannenberg, I. 1976. “Die Gattung Festuca in Griechenland.” *Veröffentlichungen des Geobotanischen Institutes der Eidg. Tech. Hochschule, Stiftung Rübel, in Zürich* 56:106.

Rechinger, Karl Heinz. 1936. “Neue Pflanzen Aus Der Aegaeis.” *Österreichische Botanische Zeitschrift* 85 (1): 56–64.

Scholz, H. 1983. “Poa- Studien 1. - Neue Taxa Des Poa Bulbosa - Komplexes Aus Dem Mittelmeergebiet.” *Botanika Chronika* 3 (1–2): 15–21.

———. 1986. “The Genus Poa (Gramineae) in Greece: Annotated Check-List and Key to the Species.” *Willdenowia* 15 (2): 393–400.

Scholz, H., and Th. Raus. 1997. “Zwei neue Unterarten des Hordeum murinum (Gramineae) aus Griechenland und Spanien.” *Feddes Repertorium* 108 (7–8): 527–31. https://doi.org/10.1002/fedr.19971080704.

Scholz, Hildemar. 1989. “Neue Taxa Der Gattung Stipa Sect. Stipa (Gramineae) Aus Dem Mittelmeergebiet.” *Willdenowia* 19 (1): 127–32.

———. 1993. “Festuca Rivularis, Poa Maroccana Und Stipa Monticola (Gramineae) in Griechenland.” *Willdenowia* 23:113–19.
———. 2000. “Alte Und Neue Taxa Der Gattung Catapodium (Gramineae).” *Botanika Chronika* 13:95–104.

Primulaceae

Greuter, Werner, and Thomas Raus. 2006. “Med-Checklist Notulae, 24.” *Willdenowia* 36 (2): 719–30.
Raus, Thomas. 1987. “Soldanella (Primulaceae) in Griechenland.” *Willdenowia* 16 (2): 335–42.
Schwarz, Otto, and Lothab Lepper. 1975. “Zwei neue Cyclamen aus dem östlichen Mittelmeergebiet.” *Feddes Repertorium* 86 (9–10): 491–97. https://doi.org/10.1002/fedr.19750860902.

Ranunculaceae

Böhling, Niels. 2000. “Ranunculus Veronicae (Ranunculaceae), a New Species from W Crete (Greece).” *Willdenowia* 30 (2): 245–50. https://doi.org/10.3372/wi.30.30203.
Dunkel, Franz G. 2015. “Ranunculus Pindicola Sp. Nov., the Only Species of the R. Auricomus Complex (Ranunculaceae) in Greece.” *Willdenowia* 45 (2): 223–30. https://doi.org/10.3372/wi.45.45208.
IUCN. 2017. “IUCN Top 50 - Consolida Samia.” 2017. https://top50.iucn-mpsg.org/species/23.
Jopek, Magdalena, Gerhard Wiegleb, Wiesław Babik, and Joanna Zalewska-Gałosz. 2023. “Ranunculus Dahlgreniae (Section Batrachium, Ranunculaceae), a New Species from Crete, Greece, with Remarks on Taxonomy and Phylogenetic Relations within the Section.” *Acta Societatis Botanicorum Poloniae* 92 (1, 167462): 4.
Nardi, Enio. 2014. “Nomenclatural Notes on Aquilegia L. (Ranunculaceae) from Europe.” *Webbia* 69 (1): 105–105. https://doi.org/10.1080/00837792.2014.895892.
Soó, Rudolf v. 1922. “Über Die Mitteleuropäischen Arten Und Formen Der Gattung Consolida (DC.) S. F. Gray.” *Österreichische Botanische Zeitschrift* 71 (10/12): 233–46.

Rosaceae

Boissier, Edmond. 1856. *Diagnoses plantarum Orientalium novarum*. Vol. 2. 2. https://bibdigital.rjb.csic.es.
Halacsy, Eugen v. 1888. “Beiträge Zur Flora Der Landschaft Doris, Insbesondere Des Gebirges Kiona in Griechenland.” *Verhandlungen Der Kaiserlich-Königlichen Zoologisch-Botanischen Gesellschaft in Wien* 38:751.
Hayek, August v. 1914. “Neue Orientalische Pflanzenarten.” *Österreichische Botanische Zeitschrift* 64 (8): 358–60.
Kurtto, Arto, and Torsten Eriksson. 2003. “Atlas Florae Europaeae Notes. 15. Generic Delimitation and Nomenclatural Adjustments in Potentilleae (Rosaceae).” *Annales Botanici Fennici* 40 (2): 135–41.
Soják, J. n.d. “Taxonomische Bemerkungen Zu Einigen Mediterranen Potentilla-Sippen.” *Preslia* 65 (2). Accessed May 26, 2025. https://www.preslia.cz/article/11439.
Wolf, Theodor. 1908. “Monographie Der Gattung Potentilla.” *Bibliotheca Botanica* 16 (Heft 71): 125.

Rubiaceae

Christodoulakis, Dimitris, and Theodoros Georgiadis. 1983. “Eine Neue Asperula-Art (Ubiaceae) von Der Insel Samos, Griechenland.” *Willdenowia* 13 (2): 281+341.
Greuter, W., U. Matthäs, and H. Risse. 1984. “Additions to the Flora of Crete, 1973-1983 (1984) - II.” *Willdenowia* 14 (2): 289.
Gutermann, Walter, and Friedrich Ehrendorfer. 2000. “Asperula Naufraga (Rubiaceae), a New Species from Zakinthos (Ionian Islands, Greece), with Notes on Its Ecology, Karyology and Relationships. (Material towards a Flora Ionica, 1).” *Botanika Chronika* 13:61–70.
Gutermann, Walter, Tae-Soo Jang, Arndt Kästner, David Prehsler, Dieter Reich, Andreas Berger, Ruth Flatscher, et al. 2024. “Thliphthisasapphus (Rubiaceae, Rubieae), a New Species from Lefkada (Ionian Islands, Greece) and Its Ecological Position.” *PhytoKeys* 241 (April):65–79. https://doi.org/10.3897/phytokeys.241.119144.
Krendl, F., and E. Vitek. 2006. “Galium Ionicum Krendl, Nomen Novum pro Galium Mixtum (Rubiaceae).” *Annalen Des Naturhistorischen Museums in Wien. Serie B Für Botanik Und Zoologie* 108:265–66.
Krendl, Franz. 1987. “Die Arten Der Galium Mollugo-Grupe in Griechenland.” *Botanika Chronika* 6–7:1–170.
Schönbeck-Temesy, Eva, and Friedrich Ehrendorfer. 1980. “New Endemic Taxa ofAsperula Sect.Cynanchicae (Rubiaceae) from the East Aegean Islands.” *Plant Systematics and Evolution* 134 (1): 133–35. https://doi.org/10.1007/BF00985035.
Trigas, Panayiotis, and Gregory Iatrou. 2003. “Asperula (Sect. Cynanchicae) Brachyphylla, Spec. Nova (Rubiaceae) from the Island of Evvia (Greece).” *Phyton (Horn, Austria)* 43 (1): 29–37.
Vladimirov, Vladimir, Mehmet Aybeke, and Kit Tan. 2019. “New Floristic Records in the Balkans: 39.”

Salicaceae

Cambria, Salvatore, Cristian Brullo, and Salvatore Brullo. 2019. “Salix Kaptarae Sp. Nov. (Salicaceae) from Crete.” *Nordic Journal of Botany* 37 (7). https://doi.org/10.1111/njb.02335.
Cambria, Salvatore, Cristian Brullo, Gianpietro Giusso Del Galdo, Saverio Sciandrello, Giuseppe Siracusa, and Salvatore Brullo. 2020. “Salix Aegaea (Salicaceae), a New Species from Island of Ikaria (Greece).” *Phytotaxa* 447 (2): 127–36. https://doi.org/10.11646/phytotaxa.447.2.5.

Santalaceae

Böhling, Niels, Werner Greuter, Thomas Raus, Britt Snogerup, and Sven Snogerup. 2002. “Notes on the Cretan Mistletoe, Viscum Album Subsp. Creticum Subsp. Nova (Loranthaceae/Viscaceae).” *Israel Journal of Plant Sciences* 50 (sup1): 77–84. https://doi.org/10.1560/RRJ4-HU15-8BFM-WAUK.

Scrophulariaceae

Edmond Boissier. 1854. *Diagnoses Plantarum Orientalium Novarum, Series 2, Vol. 3, N.º 1 - 6*. Vol. 3. http://archive.org/details/e.-boissier-diagnoses-plantarum-orientalium-novarum-series-secunda.-n.o-1-1854.

Fraas, C. 1845. *Synopsis Plantarum Florae Classicae, Oder: Uebersichtliche Darstellung Der in Den Klassischen Schriften Der Griechen Und Ro¨mer Vorkommenden Pflanzen, Nach Autoptischer Untersuchung Im Florengebiete, Entworfen Und Nach Synonymen Geordnet*. Mnchen: E. A. Fleischmann. https://doi.org/10.5962/bhl.title.6127.

Murbeck, Sv. 1933. "Monographie Der Gattung Verbascum." *Lunds Universitets Ärsskrift. N.F. Avd.2* 29 (2). https://bibdigital.rjb.csic.es.

Zografidis, Aris, Eleni Liveri, Vasilis Ioannidis, and Panayotis Dimopoulos. 2022. "Verbascum Salicifolium (Scrophulariaceae), a New Species from Central Macedonia, Greece." *PHYTOTAXA* 552 (3): 182–90.

Zografidis, Aris, and Arne Strid. 2017. "A Taxonomic Revision of the Verbascum Daenzeri Group (Scrophulariaceae)."

Tamaricaceae

Villar, Jose L., Nicholas J. Turland, Ana Juan, John F. Gaskin, Ángeles Alonso, and Manuel B. Crespo. 2015. "Tamarix Minoa (Tamaricaceae), a New Species from the Island of Crete (Greece) Based on Morphological and Plastid Molecular Sequence Data." *Willdenowia* 45 (2): 161–72.

Thymelaeaceae

Halda, J.J. 2001. "New Descriptions and Combinations." *Acta Musei Richnoviensis, Sect. Natur.* 8:34.

Tan, Kit, Ana Petrova, and Jerzy Zieliński. 2024. "Daphne Kosaninii (Thymelaeaceae) in the Balkan Peninsula." *Phytologia Balcanica* 30 (2): 151–60. https://doi.org/10.7546/PhB.30.2.2024.1.

Veronicaceae

Boissier, E. 1853. *Diagnoses plantarum Orientalium novarum.* Vol. 12. https://bibdigital.rjb.csic.es/records/item/10712-diagnoses-plantarum-orientalium-novarum-n-ordm-12.

Gustafsson, Lars-Åke. 1978. "Floristic reports from the high mountains of Sterea Ellas, Greece 1." *Botaniska notiser* 131:7–25.

Speta, F. 1986. "Heterokarpidy, Dehiscence, Heterospermy and Basifixed Seeds in Cymbalaria Hill (Scrophulariaceae) and Systematic Conclusions (Germ.)." *Phyton (Horn, Austria)* 26 (1): 50.

Violaceae

Degen, Arpad v. 1891. "Ergebnisse Einer Botanischen Reise Nach Der Insel Samothrake. (Schluss.)." *Österreichische Botanische Zeitschrift* 41 (10): 329–38.

Degen, Árpád von, and Ignaz Dörfler. 1897. "Beitrage Zur Flora Albaniens Und Macedoniens." *Denkschriften Der Kaiserlichen Akademie Der Wissenschaften / Mathematisch-Naturwissenschaftliche Classe* 64:710.

Erben, M. 1985. "Cytotaxonomische Untersuchungen an Süd-Osteuropäischen Viola-Arten Der Sektion Melanium." *Mitteilungen Der Botanischen Staatssammlung München* v.21 (1985):339–740.

Herbier Boissier., and Herbier Boissier. 1901a. *Bulletin de l'Herbier Boissier*. Vol. ser.2 v.2 1901-1902. Genève: Impr. Romet. https://www.biodiversitylibrary.org/item/105265.

———. 1901b. *Bulletin de l'Herbier Boissier*. Vol. ser.2 v.2 1901-1902. Genève: Impr. Romet. https://www.biodiversitylibrary.org/item/105265.

Merxmüller, H., and W. Lippert. 1977. "Veilchenstudien V-VII." *Mitteilungen Der Botanischen Staatssammlung München* 13:503–35.

Tiniakou, A. 1991. "Viola Dirphya (Violaceae), a New Species from Evvia Island, Greece." *CANDOLLEA* 46 (1): 119.

Trigas, Panayiotis, and Gregoris Iatrou. 2006. "The Local Endemic Flora of Evvia (W Aegean, Greece)." *Willdenowia*, 257–70.
