# Supplementary material for: The First Functional Traits Dataset for the Endemic Flora of Greece: morphology, ecology and ecosystem services
Source: Biodivers Data J. 2026 Feb 10;14:e180342. doi: 10.3897/BDJ.14.e180342 (PMC13291638; doi:10.3897/BDJ.14.e180342)
Supplement: Supplementary material 2 — Ecosystem services of the Greek endemic taxa – Bibliography per family and genus [file bdj-14-e180342-s002.pdf]

The First Functional Traits Dataset for the Endemic Flora of Greece: morphology, ecology and ecosystem services

Article type: Data Paper (Biodiversity)

Authors: Alexian Cheminal[1]*, Elpida Karadimou[1], Elisa Aubourg[2], Ioannis P. Kokkoris[3], Athanasios Kallimanis[4], Panayotis Dimopoulos[1]

[1] Laboratory of Botany, Department of Biology, University of Patras, 26504 Patras, Greece

[2] L'Institut Agro Dijon (ex-Agrosup Dijon), University of Burgundy, 21000 Dijon, France

[3] Department of Sustainable Agriculture, University of Patras, 2 G. Seferi St., 30131 Agrinio, Greece

[4] School of Biology, Aristotle University of Thessaloniki, 54124 Thessaloniki, Greece

Corresponding author: Alexian Cheminal (alexian.cheminal@upatras.gr)

Supplementary material 2: Ecosystem services of the Greek endemic taxa – Bibliography per family and genus

Aspleniaceae

Asplenium

Brownsey, P. J. 1976. 'The Origins of Asplenium Creticum and A. Haussknechtii'. *New Phytologist* 76 (3): 523–42. https://doi.org/10.1111/j.1469-8137.1976.tb01489.x.

Pinaceae

Abies

Nikolova, M., et D. Peev. 2008. « Preliminary Investigation of Antioxidant Potential and Flavonoid Content of Pinaceae Species Needles. » *Proceedings of the Fifth Conference on Medicinal and Aromatic Plants of Southeast European Countries, (5th CMAPSEEC), Brno, Czech Republic, 2-5 September, 2008.* https://www.cabdirect.org/cabdirect/abstract/20083299092.

Verykokidou, E., H. Skaltsa, M. Couladis, et A. Delitheos. 1995. « Antibacteriophage Properties of Some Greek Plant Extracts ». *International Journal of Pharmacognosy* 33 (4): 339-43. https://doi.org/10.3109/13880209509065389.

Alliaceae (now part of Amaryllidaceae)

Ahmet Sargin, Seyid. 2015. « Ethnobotanical Survey of Medicinal Plants in Bozyazı District of Mersin, Turkey ». Journal of Ethnopharmacology 173 (septembre): 105-26. https://doi.org/10.1016/j.jep.2015.07.009.

Aoba, Takashi. 1970. « Effect of Low Temperature on the Bulb or Corm Formation in Some Ornamental Plants ». Journal of the Japanese Society for Horticultural Science 39 (4): 369-74. https://doi.org/10.2503/jjshs.39.369.

Biel, Burkhard, Kit Tan, et Dimitrios Tzanoudakis. 2006. « A New Autumn-Flowering Species of Allium (Liliaceae) from the Island of Sifnos (Cyclades, Greece) ». Willdenowia 36 (1): 367-72.

Bogdanović, Sandro, Cristian Brullo, Salvatore Brullo, Gianpietro Giusso Del Galdo, Carmelo Maria Musarella, et Cristina Salmeri. 2011. « Allium Cithaeronis Bogdanović, C. Brullo, Brullo, Giusso, Musarella & Salmeri (Alliaceae), a New Species from Greece ». Candollea 66 (2): 377-82. https://doi.org/10.15553/c2011v662a15.

Brullo, Cristian, Salvatore Brullo, Gianpietro Giusso del Galdo, et Cristina Salmeri. 2010. « Allium makrianum (Alliaceae), a new autumnal species from Greece ». Phyton (Horn, Austria) 49 (2): 267-78.

Brullo, S, A Guglielmo, P Pavone, et C Salmeri. 2007. « Cytotaxonomic Considerations on Allium Stamineum Boiss. Group (Alliaceae) », 19.

Brullo, S., A. Guglielmo, P. Pavone, et C. Salmeri. 2001. « Cytotaxonomical Notes on Some Rare Endemic Species of Allium (Alliaceae) from Greece ». Caryologia 54 (1): 37-57. https://doi.org/10.1080/00087114.2001.10589212.

Brullo, S., Anna Guglielmo, Pietro Pavone, Cristina Salmeri, et M. Carmen Terrasi. 2003. « Three New Species of Allium Sect. Codonoprasum from Greece ». Plant Biosystems - An International

Journal Dealing with All Aspects of Plant Biology 137 (2): 131-40. https://doi.org/10.1080/11263500312331351391.

Brullo, S., et C. Salmeri. 2021. « Taxonomic Investigation on Allium Hirtovaginum Group (Amaryllidaceae) from East Mediterranean Area ». Flora Mediterranea 31 (Special Issue). https://doi.org/10.7320/FlMedit31SI.169.

Brullo, S., P. Pavone, et C. Salmeri. 2015. « Biosystematic Researches on Allium Cupani Group (Amaryllidaceae) in the Mediterranean Area ». Flora Mediterranea 25 (Special Issue). https://doi.org/10.7320/FlMedit25SI.209.

Brullo, Salvatore, et Dimitris Tzanoudakis. 1989. « Allium maniaticum (Liliaceae), a New Species from S Greece ». Willdenowia 19 (1): 111-14.

Brullo, Salvatore, et Dimitris Tzanoudakis. 1994. « Allium ionicum (Liliaceae), a New Species from the Ionian Islands (W Greece) ». Willdenowia 24 (1/2): 53-57.

Brullo, Salvatore, Gianpietro Giusso del Galdo, et Maria Carmen Terrasi. 2008. « Allium Aeginiense Brullo, Giusso & Terrasi (Alliaceae), a New Species from Greece ». Candollea 63 (2): 197-203.

Brullo, Salvatore, Pietro Pavone, Cristina Salmeri, et Dimitris Tzanoudakis. 1994. « Cytotaxonomical Revision or the Allium Obtusiflorum Group (Alliaceae) ». Flora Mediterranea 4: 12.

Brullo, Salvatore, Pietro Pavone, et Cristina Salmeri. 1992. « Allium rhodiacum (Liliaceae), a New Species from Rhodos (Greece) ». Willdenowia 22 (1/2): 89-95.

Brullo, Salvatore, Pietro Pavone, et Cristina Salmeri. 1997. « Allium Karistanum (Liliaceae), a New Species from Evvia (Greece) ». Bocconea 5: 756-64.

Brullo, Salvatore, Pietro Pavone, et Cristina Salmeri. 1999. « Allium Archeotrichon (Alliaceae), a New Species from Rhodos (Dodekannìsos, Greece) ». Nordic Journal of Botany 19 (1): 41-46. https://doi.org/10.1111/j.1756-1051.1999.tb01901.x.

Brullo, Salvatore, Pietro Pavone, et Cristina Salmeri. 2001. « Allium Brachyspathum (Alliaceae), a New Species from the Island of Karpathos (8 Aegean Area, Greece) ». Bocconea 13: 413-17.

Brullo, Salvatore, Pietro Pavone, et Dimitris Tzanoudakis. 1993. « Allium lagarophyllum (Liliaceae), a New Species from Greece ». Willdenowia 23 (1/2): 107-11.

Burton, Rodney. 2006. « A New Subspecies of Allium Circinnatum Sieber from S.W. Turkey », 349.

Cattaneo, Cristina. 2020. « Description of a New Allium Species from Symi Island (SE Aegean, Greece): Allium Carlstroemi Sp. Vov (Amaryllidaceae) ». Parnassia Archives 8: 103-11.

Chasapis, M., D. A. Samaras, K. Theodoropoulos, et E. Eleftheriadou. 2020. « The Vascular Flora of Mt Tzena (Northern Greece) ». Flora Mediterranea 30: 55-63. https://doi.org/10.7320/FlMedit30.055.

Galanos, Christos J, et Dimitrios Tzanoudakis. 2019. « Allium Panormitisi (Amaryllidaceae), a New Autumn- Flowering Species from Symi Island, SE Aegean, Greece ». Botanica Serbica 43 (2): 197-203.

Galanos, Christos J., et Dimitris Tzanoudakis. 2017. « Allium symiacum (Amaryllidaceae), a new species from Symi Island (SE Aegean, Greece) ». Willdenowia 47 (2): 107-13. https://doi.org/10.3372/wi.47.47202.

Giannopoulos, Konstantinos, Kit Tan, et Gert Vold. 2021. Contributions to the Bulb Flora of Ilias (NW Peloponnese, Greece): Alliaceae. 12.

Iatrou, Gregory, et Dimitris Tzanoudakis. 1995. « Allium Ritsii (Alliaceae) a New Autumn-Flowering Species from S. Peloponnisos (Greece) », janvier, 7.

Ioannidis, Vasilis, et Dimitris Tzanoudakis. 2020. Allium Arampatzisii (Amaryllidaceae) – a New Species from Northern Continental Greece. 8.

Ioannidis, Vasilis, et Dimitris Tzanoudakis. 2022. Allium goumenissanum a new autumnal species of Allium sect. Codonoprasum (Amaryllidaceae) from north Greece. https://www.cabidigitallibrary.org/doi/full/10.5555/20220311465.

Kalpoutzakis, Eleftherios, Panayiotis Trigas, et Theophanis Constantinidis. 2012. « Allium Orestis Sp. Nov. (Amaryllidaceae) from Parnon and Taigetos Mountains, South Peloponnisos, Greece ». Nordic Journal of Botany 30 (2): 195-200. https://doi.org/10.1111/j.1756-1051.2011.01490.x.

Kalpoutzakis, Eleftherios, Theophanis Constantinidis, et Panayiotis Trigas. 2019. « Chorological Additions for Some Noteworthy Taxa of the Greek Flora », 18.

Karetsos, George, Alexandra D. Solomou, Panayiotis Trigas, et Konstantinia Tsagari. 2018. « The Vascular Flora of Mt. Oiti National Park and the Surrounding Area in Greece ». Journal of Forest Science 64 (No. 10): 435-54. https://doi.org/10.17221/65/2018-JFS.

Kılıçaslan, Naşit, et Şirin Dönmez. 2016. « Göller bölgesinde doğal olarak yetişen soğanlı bitkilerin peyzaj mimarlığında kullanımı - Utilization of bulbous plants in landscape architecture growing in Lakes Region ». Turkish Journal of Forestry | Türkiye Ormancılık Dergisi 17 (1). https://doi.org/10.18182/tjf.36974.

Kougioumoutzis, Konstantinos, Panayiota Kotsakiozi, Efthalia Stathi, Panayiotis Trigas, et Aristeidis Parmakelis. 2021. « Conservation Genetics of Four Critically Endangered Greek Endemic Plants: A Preliminary Assessment ». Diversity 13 (4): 4. https://doi.org/10.3390/d13040152.

Libiad, Mohamed, Abdelmajid Khabbach, Mohamed El Haissoufi, et al. 2021. « Agro-Alimentary Potential of the Neglected and Underutilized Local Endemic Plants of Crete (Greece), Rif-Mediterranean Coast of Morocco and Tunisia: Perspectives and Challenges ». Plants 10 (9): 9. https://doi.org/10.3390/plants10091770.

Liveri, Eleni, E. Katopodi, et Georgia Kamari. 2019. « Karyosystematic Study of Some Taxa from the Ionian Floristic Region (Greece). II ». Flora Mediterranea 29. https://doi.org/10.7320/FlMedit29.308.

Löve, Áskell. 1981. « Chromosome Number Reports LXXIII ». Taxon 30 (4): 829-61.

Löve, Áskell. 1984. « Chromosome Number Reports LXXXII ». Taxon 33 (1): 126-34.

Maggioni, Lorenzo, J. Keller, D. Astley, et compilers. 2002. European Collections of Vegetatively Propagated Allium: Report of a Workshop, 21-22 May 2001, Gatersleben, Germany. International Plant Genetic Resources Institute.

Maxted, Nigel. 2008. Crop Wild Relative Conservation and Use. CABI.

Mertzanis, Aristeidis, Stylianos Syleounis, Konstantinos Mertzanis, Athanasios Skouras, et George Efthimiou. 2015. « Nature Trails Management and Enhancement: The Case of Hercules' Trail at the Oiti Mountain (Greece) ». Ecology & Safety, Journal of International Scientific Publications 9: 151-70.

Musarella, Carmelo Maria, Salvatore Brullo, et Gianpietro Giusso Del Galdo. 2020. « Contribution to the orophilous cushion-like vegetation of central-southern and insular Greece ». Plants 9 (12): 1678.

Panitsa, Maria, Ioannis Bazos, Panayotis Dimopoulos, Sevasti Zervou, Artemios Yannitsaros, et Dimitrios Tzanoudakis. 2004. « Contribution to the study of the flora and vegetation of the Kithira island group: Offshore islets of Kithira (S Aegean, Greece) ». Willdenowia 34 (1): 101-15. https://doi.org/10.3372/wi.34.34109.

Panitsa, Maria, Panayiotis Trigas, Dimitrios Kontakos, Anna-Thalassini Valli, et Gregoris Iatrou. 2021. « Natural and cultural heritage interaction: aspects of plant diversity in three East Peloponnesian castles (Greece) and conservation evaluation ». Plant Biosystems - An International Journal Dealing with all Aspects of Plant Biology 0 (0): 1-15. https://doi.org/10.1080/11263504.2021.1889701.

Pasta, S., A. Perez-Graber, L. Fazan, et B De Montmollin. 2017. The Top 50 Mediterranean Island Plants, Update 2017. IUCN/SSC/Mediterranean Plant Specialist Group. https://top50.iucn-mpsg.org/book.

Raab-Straube, Eckhard von, et Thomas Raus. 2021. « Euro+Med-Checklist Notulae, 13 ». Willdenowia 51 (1). https://doi.org/10.3372/wi.51.51112.

Ricroch, A, R Yockteng, S C Brown, et S Nadot. 2005. « Evolution of Genome Size across Some Cultivated Allium Species ». Genome 48 (3): 511-20. https://doi.org/10.1139/g05-017.

Salmeri, C., S. Brullo, A. Guglielmo, et P. Pavone. 2007. « Allium exile Boiss. & Orph. (Alliaceae), a misappreciated species of Greek flora ». Acts of XII OPTIMA Meeting, septembre 16, 119-119. https://iris.unipa.it/handle/10447/6611.

Salmeri, Cristina. 1998. « Allium brulloi (Alliaceae), a New Species from Astypalea (Aegean Islands, Greece) ». Willdenowia 28 (1/2): 69-75.

Samaras, Stelios. 2002. « Report on the Current Status of the Greek Allium Wild Taxa Collection Maintained at the Greek Gene Bank ». In European Collections of Vegetatively Propagated Allium: Report of a Workshop, 21-22 May 2001, Gatersleben, Germany, par Lorenzo Maggioni. Bioversity International.

Seregin, A P. 2004. « Additions to Allium Sect. Allium (Alliaceae) from North Africa ». Komarovia 4: 5.

Spanos, Ioannis, Panagiotis Platis, Ioannis Meliadis, et Alexandros Tsiontis. 2008. « A Review on the Ecology and Management of the Samaria Gorge, a Greek Biosphere Reserve ». Journal of Geography and Regional Planning 1 (2): 19-33.

Stevanovic, V., Kit Tan, et G. Iatrou. 2003. « Distribution of the Endemic Balkan Flora on Serpentine I. - Obligate Serpentine Endemics ». Plant Systematics and Evolution 242 (1-4): 149-70. https://doi.org/10.1007/s00606-003-0044-8.

Strid, Arne, et Kit Tan. 2017. « Recent Progress In Plant Taxonomy And Floristic Studies In Greece ». Botanica Serbica 41 (2): 123-52. https://doi.org/10.5281/ZENODO.1026649.

Tambets, Kersti. 2021. Index Seminum. Hortus Botanicus Universitatis Tartuensis. https://www.botaanikaaed.ut.ee/sites/botaanikaaed/files/pildid/IndexSeminum2021.pdf.

Trigas, P., et G. Iatrou. 1998. « Contribution to the Biodiversity (Botanical Diversity) and Phytogeography of the Island of Euboea. » In Progress in Botanical Research: Proceedings of the 1st Balkan Botanical Congress, édité par Ioannes Tsekos et Michael Moustakas. Springer Netherlands. https://doi.org/10.1007/978-94-011-5274-7_37.

Trigas, Panayiotis, Eleftherios Kalpoutzakis, et Theophanis Constantinidis. 2017. « Two New Allium (A. Sect. Cupanioscordum, Amaryllidaceae) Species from Greece ». Phytotaxa 297 (2): 179-88.

Trigas, Panayiotis, et Dimitris Tzanoudakis. 2000. « Allium Runemarkii (Liliaceae), a New Species from the Island of Ewia (W Aegean, Greece) ». Nordic Journal of Botany 20 (1): 89-92. https://doi.org/10.1111/j.1756-1051.2000.tb00737.x.

Trigas, Panayiotis, et Gregoris Iatrou. 2006. « The Local Endemic Flora of Evvia (W Aegean, Greece) ». Willdenowia 36 (1): 257-70. https://doi.org/10.3372/wi.36.36121.

Trigas, Panayiotis, et Pepy Bareka. 2020. « Allium Stamatiadae, a New Species of Sect. Codonoprasum (Amaryllidaceae) from Andros Island (Aegean Archipelago, Greece) ». Phytotaxa 443 (3): 3. https://doi.org/10.11646/phytotaxa.443.3.5.

Trigas, Panayiotis, Gregoris Iatrou, et Dimitris Tzanoudakis. 2010. « Allium Apergii Sp. Nov. (Alliaceae, A. Sect. Codonoprasum) from Evvia Island, Greece ». Journal of Biological Research-Thessaloniki 14: 225-29.

Tzanoudakis, D, et Z Kypriotakis. 1993. « Allium Platakisii, a New Species of the Greek Insular Flora ». Flora Mediterranea, 6.

Tzanoudakis, D. 1986. « Chromosome Studies in the Greek Flora. II. Karyotypes of Four Aegean Endemics of Allium sect. Codonoprasum (Liliaceae) ». Willdenowia 16 (1): 203-11.

Tzanoudakis, Dimitrios, Maria Panitsa, Panayiotis Trigas, et Gregoris Iatrou. 2006. « Floristic and Phytosociological Investigation of the Island Antikythera and Nearby Islets (SW Aegean, Greece) ». Willdenowia 36 (1): 285-301. https://doi.org/10.3372/wi.36.36123.

Tzanoudakis, Dimitris, et Canio G Vosa. 1986. « The Cytogeographieal Distribution Pattern of Allium (Alliaceae) in the

Greek Peninsula and Islands ». Plant Systematics and Evolution 159: 193-215.

Tzanoudakis, Dimitris, et Fania Kollmann. 1991. « Allium chalkii (Liliaceae), a New Species from the Eastern Aegean Island of Chalki (Greece) ». Israel Journal of Botany 40 (1): 61-64. https://doi.org/10.1080/0021213X.1991.10677177.

Tzanoudakis, Dimitris, et Kit Tan. 2000. « Allium Samothracicum Tzanoud., Strid & Kit Tan, a New Species from the North Aegean Area, Greece ». Portugaliae Acta Biol. 19: 355-60.

Tzanoudakis, Dimitris, et Panayiotis Trigas. 2015. « Allium Occultum, a New Species of A. Sect. Codonoprasum (Amaryllidaceae) from Skiros Island (W Aegean, Greece) ». Phytotaxa 202 (2): 135-42.

Tzanoudakis, Dimitris, et Zacharias Kypriotakis. 2008. « Allium brussalisii (Alliaceae), a new species from Greece ». Botanical Journal of the Linnean Society 158 (1): 140-46. https://doi.org/10.1111/j.1095-8339.2008.00853.x.

Tzanoudakis, Dimitris, Maria Tsakiri, et Thomas Raus. 2019. « What is Allium achaium Boiss. & Orph.? Disentangling the taxonomy of a Greek mountain species ». Willdenowia 49 (2): 231-39. https://doi.org/10.3372/wi.49.49211.

Tzanoudakis, Dimitris, Maria Tsakiri, et Thomas Raus. 2019. « What is Allium achaium Boiss. & Orph.? Disentangling the taxonomy of a Greek mountain species ». Willdenowia 49 (2): 231-39. https://doi.org/10.3372/wi.49.49211.

Tzanoudakis, Dimitris. 1983. « Karyotypes of Ten Taxa of Allium Section Scorodon from Greece ». Caryologia 36 (3): 259-84. https://doi.org/10.1080/00087114.1983.10797667.

Vojtěchová, Kateřina, Lucie Kobrlová, Miloslav Kitner, et al. 2023. « Allium Goumenissanum (Allium Sect. Codonoprasum), a New Species for Bulgaria and New Localities in Greece: Testing an Integrated Approach to Species Identification ». Mediterranean Botany Online first (novembre): 1-23. https://doi.org/10.5209/mbot.89106.

Καραβοκύρου, Ευδοξία. 1995. « Μελετη Του Φυτικου Γενους Allium Στην Ανατολικη Ελλαδα - Study of the Plant Genus Allium in Eastern Greece ». Πανεπιστήμιο Πατρών; University of Patras. http://oatd.org/oatd/record?record=handle%5C%3A10442%5C%2Fhedi%5C%2F3404.

Μαστοροπούλου, Σοφία Σ. 2019. « Προς ένα βιώσιμο πρότυπο τουριστικής ανάπτυξης στη Νήσο Νάξο-Προσέγγιση σε Μικρο-και Μακρο-επίπεδο - Towards a sustainable model of tourism development on Naxos Island-Approach to Micro-and Macro-level ». ΕΘΝΙΚΟ ΜΕΤΣΟΒΙΟ ΠΟΛΥΤΕΧΝΕΙΟ.

Τσακίρη, Μαρία. 2014. « Νήσος Χάλκη : καταγραφή της χλωριδικής ποικιλότητας, βιοπαρακολούθηση των σπάνιων και ενδημικών φυτών της και η συμβολή τους στην οικοτουριστική ανάπτυξη της περιοχής - Chalki : recording of floristic diversity, biomonitoring of rare and endemic plants, and its contribution to the ecotourism development of the island ». Thesis, University of Patras. http://nemertes.library.upatras.gr/jspui//handle/10889/9655.

Amaryllidaceae

Galanthus

Arslan, N., M. Koyuncu, et T. Ekim. 1997. « Commercial propagation of snowdrops (Galanthus elwesii Hook.) in different environments ». *Acta Horticulturae*, nº 430 (décembre): 743-46. https://doi.org/10.17660/ActaHortic.1997.430.118.

Şahin, Nevin Ferda. 1998. « Morphological, anatomical and physiological studies on Galanthus ikariae Baker and G. Rizehensis Stern (Amaryllidaceae) grown around NE Turkey ». *Pak. J. Bot.* 30 (1): 117-31.

Şahin, Nevin Ferda, Nazmiye Şakiyan, et Nur Münevver Pinar. 1997. « An Investigation on the Pollen Morphology of Galanthus Ikariae Baker and Galanthus Rizehensis Stern (Amaryllidaceae) ». *TURKISH JOURNAL OF BOTANY* 21 (5): 305-7.

Şener, Bilge, et İlkay Orhan. 2005. « Discovery of Drug Candidates from Some Turkish Plants and Conservation of Biodiversity ». *Pure and Applied Chemistry* 77 (1): 53-64. https://doi.org/10.1351/pac200577010053.

Tan, Kit, Burkhard Biel, et Sonja Siljak-Yakovlev. 2014. « Galanthus Samothracicus (Amaryllidaceae) from the Island of Samothraki, Northeastern Greece ». *Phytologia Balcanica* 20 (1): 65-70.

Tipirdamaz, Rukiye. 2003. « Rooting and Acclimatization of in Vitro Micropropagated Snowdrop (Galanthus Ikariae Baker.) Bulblets ». *AKDENİZ ÜNİVERSİTESİ ZİRAAT FAKÜLTESİ DERGİSİ* 16 (2): 121-26.

Üçüncü, Osman, Cemalettin Baltaci, Şeyda Merve Karataş, et al. 2019. « Galanthus ikariae Baker Bitkisinin Toprak Üstü Kısımlarının Uçucu Yağının Kimyasal Bileşimi ve Biyolojik Aktiviteleri ». *Gümüşhane Üniversitesi Fen Bilimleri Enstitüsü Dergisi* 9 (4): 674-80. https://doi.org/10.17714/gumusfenbil.525990.

Sternbergia

Gage, Ewan, Paul Wilkin, Mark W. Chase, et Julie A. Hawkins. 2011. « Phylogenetic systematics of Sternbergia (Amaryllidaceae) based on plastid and ITS sequence data ». *Botanical Journal of the Linnean Society* 166 (2): 149-62. https://doi.org/10.1111/j.1095-8339.2011.01138.x.

Kamari, Georgia, et Rea Artelari. 1990. « Karyosystematic Study of the Genus Sternbergia (Amaryllidaceae) in Greece. I. South Aegean Islands ». *Willdenowia* 19 (2): 367-88.

Apiaceae

Bunium

Stevanovic, V., Kit Tan, et G. Iatrou. 2003. « Distribution of the Endemic Balkan Flora on Serpentine I. - Obligate Serpentine Endemics ». *Plant Systematics and Evolution* 242 (1-4): 149-70. https://doi.org/10.1007/s00606-003-0044-8.

Bupleurum

Council Directive 92/43/EEC of 21 May 1992 on the conservation of natural habitats and of wild fauna and flora, 31992L0043 7 (1992).

Ecosistemaglobal. 2012. « Bupleurum kakiskalae ». *Flora en peligro de extincion en bosque mediterraneo*, janvier 26. https://ecosistemaglobal.wordpress.com/2012/01/26/bupleurum-kakiskalae/.

Fournaraki, C, I Remoundou, et C A Thanos. 2008. « Ex Situ Conservation of European Threatened Plants in Western Crete, Greece (CRETAPLANT Project, EU-LIFE) ». Poster. Agraria Universita Mediterranensium Nationum. http://cretaplant.biol.uoa.gr/posters/FournarakiPerthPoster.pdf.

Snogerup, Sven, et Britt Snogerup. 2003. « Local Endemism in European Annual Bupleurum (Umbelliferae) », 5.

Thanos, Costas A., C Fournaraki, Kyriacos Georghiou, Panayotis Dimopoulos, et Erwin Bergmeier. 2008. *The establishment, monitoring and management of a pilot network of micro-reserves in Western Crete for the conservation of European threatened plants (CRETAPLANT Project, EU-LIFE)*.

Turland, Nicholas J. 2008. « Anthemis Samariensis (Asteraceae, Anthemideae), a New Species from the Mountains of W Kriti (Greece) ». *Willdenowia* 38 (1): 61. https://doi.org/10.3372/wi.38.38103.

Ταμπούκου, Άννας. 2010. « Βοτανικοί Κήποι και ο ρόλος τους στο χώρο της Περιβαλλοντικής Εκπαίδευσης - Botanical Gardens and their role in the field of education to environment ». Postgraduate, ΓΕΩΠΟΝΙΚΟ ΠΑΝΕΠΙΣΤΗΜΙΟ ΑΘΗΝΩΝ. http://dspace.aua.gr/xmlui/bitstream/handle/10329/6294/Tampoukou_A.pdf?sequence=1.

Carum

Giannopoulou, Christinas. 2016. « ΜΕΛΕΤΗ ΑΥΤΟΦΥΩΝ ΚΑΙ ΕΝΔΗΜΙΚΩΝ ΦΥΤΩΝ ΤΟΥ ΤΑΥΓΕΤΟΥ - Study of natural and indigenous plants of Taygetos ». Kalamata.

Marhold, Karol, Jaromír Kučera, Alexander V. Agafonov, Tatiana V. Alexeeva, Sergei V. Asbaganov, Mikhail P. Danilov, Olga V. Dorogina, et al. 2019. « IAPT Chromosome Data 29 ». *TAXON* 68 (4): 880-83. https://doi.org/10.1002/tax.12130.

Zakharova, Ekaterina A, Eugene V Kljuykov, Galina V Degtjareva, Tahir H Samigullin, Uliana A Ukrainskaya, et Stephen R Downie. 2016. « A Taxonomic Study of the Genus Hellenocarum H.Wolff (Umbelliferae-Apioideae) Based on Morphology, Fruit Anatomy, and Molecular Data ». *Turk J Bot*, 20.

Conium

Evergetis, Epameinondas, Antonios Michaelakis, et Serkos A. Haroutounian. 2012. « Essential Oils of Umbelliferae (Apiaceae) Family Taxa as Emerging Potent Agents for Mosquito Control ». In *Integrated Pest Management and Pest Control - Current and Future Tactics*, par Sonia Soloneski, 613-38. https://doi.org/10.5772/1383.

Kamboukou, Christina-Anna. 2019. « ΜΕΛΕΤΗ ΤΗΣ ΑΝΤΙΟΞΕΙΔΩΤΙΚΗΣ ΔΡΑΣΗΣ ΤΟΥ ΕΚΧΥΛΙΣΜΑΤΟΣ ΤΟΥ ΦΥΤΟΥ Conium divaricatumΣΕ ΗΠΑΤΙΚΑ ΚΥΤΤΑΡΑ HepG2 - Assessment of the antioxidant activity of Conium divaricatum extract in HepG2 hepatic cells. » Larisa: 1 ΠΑΝΕΠΙΣΤΗΜΙΟ ΘΕΣΣΑΛΙΑΣΣΧΟΛΗ ΕΠΙΣΤΗΜΩΝ ΥΓΕΙΑΣΤΜΗΜΑ ΒΙΟΧΗΜΕΙΑΣ ΚΑΙ ΒΙΟΤΕΧΝΟΛΟΓΙ. https://ir.lib.uth.gr/xmlui/bitstream/handle/11615/50191/18880.pdf?sequence=1.

Marhold, Karol, Jaromír Kučera, Julio Rubén Daviña, et Ana Isabel Honfi. 2018. « IAPT Chromosome Data 28 ». *Taxon* 67 (6): 1235-45. https://doi.org/10.12705/676.39.

Διατριβή, Μεταπτυχιακή, et Ευτυχία Κοντοζήση. 2019. « Μελέτη της αντιοξειδωτικής δράσης τουεκχυλίσματος του φυτού Conium divaricatumσε ενδοθηλιακά κύτταρα - Study of antioxidant activity of Conium divaricatum extract on endothelial cells. » Larisa: ΠΑΝΕΠΙΣΤΗΜΙΟ ΘΕΣΣΑΛΙΑΣ ΣΧΟΛΗ ΕΠΙΣΤΗΜΩΝ ΥΓΕΙΑΣ ΤΜΗΜΑ ΒΙΟΧΗΜΕΙΑΣ ΚΑΙ ΒΙΟΤΕΧΝΟΛΟΓΙΑΣ.

Λαδας, Δημήτριος. 2018. « Προσδιορισμός της αντιοξειδωτικής δράσης σε εκχυλίσματα από Conium divaricatum, Ruta graveolens, και Artemisia arborescens - Assessment of antioxidant activity of extracts from Conium divaricatum, Ruta graveolens and Artemisia arborescens ». Larisa: ΠΑΝΕΠΙΣΤΗΜΙΟ ΘΕΣΣΑΛΙΑΣΣΧΟΛΗ ΕΠΙΣΤΗΜΩΝ ΥΓΕΙΑΣΤΜΗΜΑ ΒΙΟΧΗΜΕΙΑΣ ΚΑΙ ΒΙΟΤΕΧΝΟΛΟΓ. https://ir.lib.uth.gr/xmlui/bitstream/handle/11615/48245/17438.pdf?sequence=1.

Chaerophyllum

Ebadollahi, Asgar. 2013. « Plant Essential Oils from Apiaceae Family », juin, 24.

Evergetis, Epameinondas, Antonios Michaelakis, et Serkos A. Haroutounian. 2012. « Essential Oils of Umbelliferae (Apiaceae) Family Taxa as Emerging Potent Agents for Mosquito Control ». In *Integrated Pest Management and Pest Control - Current and Future Tactics*, par Sonia Soloneski, 613-38. https://doi.org/10.5772/1383.

Prokopiou, Lydia, Abealghani Halahlah, Spyros Grigorakis, Christini Fournaraki, Eugene Kokkalou, et Anastasia Karioti. 2021. « Threatened Cretan species Chaerophyllum creticum Boiss. & Heldr.: phenolic profile by HPLC-PDA-MS and in vitro antioxidant capacity ». *Natural Product Research* 0 (0): 1-6. https://doi.org/10.1080/14786419.2021.1889545.

Strid, Arne. 2020. « The Botanical Exploration of Greece ». *Plant Systematics and Evolution* 306 (2): 27. https://doi.org/10.1007/s00606-020-01637-z.

Dichoropetalum

Averyanov. 2016. « Xyloselinum Laoticum (Umbelliferae), a New Species from Laos, and Taxonomic Placement of the Genus in the Light of NrDNA ITS Sequence Analysis ». *Phytotaxa* 244 (3): 248. https://doi.org/10.11646/phytotaxa.244.3.2.

Eryngium

Fokialakis, N., E. Kalpoutzakis, B. L. Tekwani, et al. 2007. « Evaluation of the Antimalarial and Antileishmanial Activity of Plants from the Greek Island of Crete ». *Journal of Natural Medicines* 61 (1): 38-45. https://doi.org/10.1007/s11418-006-0013-y.

Fokialakis, Nikolas, Charles L. Cantrell, Stephen O. Duke, Alexios L. Skaltsounis, et David E. Wedge. 2006. « Antifungal Activity of Thiophenes from Echinops Ritro ». *Journal of Agricultural and Food Chemistry* 54 (5): 1651-55. https://doi.org/10.1021/jf052702j.

Goetz, P. 2006. « Traitement des troubles de la libido masculine - Treatments for male libido disorders ». *Phytothérapie* 4 (1): 9-14. https://doi.org/10.1007/s10298-006-0142-1.

Wörz, Arno. 2006. *Systematics and Distribution Patterns of the Balkan Species of Eryngium (Apiaceae, Saniculoideae)*. 10.

Ferulago

Demetzos, Costas, Dimitrios Perdetzoglou, Maria Gazouli, Kit Tan, et Costas Economakis. 2000. « Chemical Analysis and Antimicrobial Studies on Three Species of Ferulago from Greece ». *Planta Medica* 66 (6): 560-63. https://doi.org/10.1055/s-2000-8652.

Snogerup, Sven, Britt Snogerup, Elli Stamatiadou, Roland von Bothmer, et Mats Gustafsson. 2006. « Flora and Vegetation of Andros, Kikladhes, Greece ». *Ann. Musei Goulandris* 11: 85-270.

Stevanovic, V., Kit Tan, et G. Iatrou. 2003. « Distribution of the Endemic Balkan Flora on Serpentine I. - Obligate Serpentine Endemics ». *Plant Systematics and Evolution* 242 (1-4): 149-70. https://doi.org/10.1007/s00606-003-0044-8.

Geocaryum

Evergetis, Epameinondas, et Serkos A. Haroutounian. 2014. « Exploitation of Apiaceae Family Plants as Valuable Renewable Source of Essential Oils Containing Crops for the Production of Fine Chemicals ». *Industrial Crops and Products* 54 (mars): 70-77. https://doi.org/10.1016/j.indcrop.2014.01.009.

Médail, Frédéric. 2013. « The Unique Nature of Mediterranean Island Floras and the Future of Plant Conservation ».

Panousi, Panagiota. 2011. « The endemic flora of Mount Parnon ». Kalamata: Τεχνολογίας Γεωπονίας, Φυτικής Παραγωγής. http://nestor.teipel.gr/xmlui/bitstream/handle/123456789/17618/STEG_FP_00693_Medium.pdf?sequence=1.

Strid, Arne. 2020. « The Botanical Exploration of Greece ». *Plant Systematics and Evolution* 306 (2): 27. https://doi.org/10.1007/s00606-020-01637-z.

Trigas, Panayiotis, et Gregoris Iatrou. 2006. « The Local Endemic Flora of Evvia (W Aegean, Greece) ». Willdenowia 36 (1): 257-70. https://doi.org/10.3372/wi.36.36121.

Zogaris, Stamatis, et Basiliki Blami. 2011. Προτάσεις οικοτουριστικής ανάδειξης με σκοπό τη διατήρηση της παράκτιας βιοποικιλότητας της Θάσου. μοσίευτη έκθεση προς Δήμο Θάσου & Περιφέρεια Ανατολικής Μακεδονίας και Θράκης. - Ecotourism promotion proposals in order to preserve the coastal biodiversity of Thassos. Municipality of Thassos. https://www.researchgate.net/profile/Stamatis-Zogaris/publication/259292233_Ecotourism_proposals_for_the_promotion_of_coastal_biodiversity_conservation_on_Thassos_Greece/data/0046352aca7afa51a6000000/thassos-z-v-final2011.pdf.

Βεις, Δημήτριος. 2011. « Αρωμάτικα και φαρμακεύτικα φυτά Πελοποννήσου: Ρίγανη, Υπέρικο, Ταπάξακο - Aromatic and pharmaceutical plants of Peloponnese: Oregano, Hypericum, Taraxacum ». A.T.E.I. Kalamatas. http://nestor.teipel.gr/xmlui/bitstream/handle/123456789/13572/STEG_THEKA_00486_Medium.pdf?sequence=1.

Heptatera

Evergetis, Epameinondas, Sofia D. Koulocheri, et Serkos A. Haroutounian. 2015. « Exploitation of Apiaceae Family Plants as Valuable Renewable Source of Essential Oils Containing Crops for the Production of Fine Chemicals: Part II ». *Industrial Crops and Products* 64 (février): 59-67. https://doi.org/10.1016/j.indcrop.2014.10.069.

Krigas, Nikos, Marina Panagiotidou, et Eleni Maloupa. 2017. « Incorporating biogeographical principles in horticulture: design and creation of the ionian islands unique rock garden in Thessaloniki, Greece. » *Sibbaldia: The Journal of Botanic Garden Horticulture*, 2017, 15 édition.

Maloupa, E, N Krigas, et A Karydas. 2007. « The in Situ Plant Conservation Actions of the Balkan Botanic Garden of Kroussia in Greece », 5.

Mousavi, Sabereh, Valiollah Mozaffarian, Klaus Mummenhoff, Stephen R. Downie, et Shahin Zarre. 2020. « An updated lineage-based tribal classification of Apiaceae subfamily Apioideae with special focus on Iranian genera ». *Systematics and Biodiversity* 19 (1): 89-109. https://doi.org/10.1080/14772000.2020.1834002.

Horstrissea

Egli, Bernhard, Pedro Gerstberger, Werner Greuter, et Horst Risse. 1990. « Horstrissea dolinicola, a New Genus and Species of Umbels (Umbelliferae, Apiaceae) from Kriti (Greece) ». *Willdenowia* 19 (2): 389-99.

Fenu, Giuseppe, Gianluigi Bacchetta, S. Christodoulou Charalambos, et al. 2019. « An Early Evaluation of Translocation Actions for Endangered Plant Species on Mediterranean Islands ». *Plant Diversity*, Restoration of threatened plant species and their habitats, vol. 41 (2): 94-104. https://doi.org/10.1016/j.pld.2019.03.001.

Quintero Ruiz, Joab Raziel, et Laura Yanez Espinosa. 2017. « Los relieves karsticos, formaciones invaluables de la naturaleza - Karst landforms, invaluables natural formations ». *Universitarios Potosinos* 208 (février): 4-10.

Vargas, Pablo, Pedro Jiménez-Mejías, et Mario Fernández-Mazuecos. 2020. « 'Endangered Living Fossils' (ELFs): Long-Term Survivors through Periods of Dramatic Climate Change ». *Environmental and Experimental Botany*, The climatic challenge: learning from past survivors and present outliers, vol. 170 (février): 103892. https://doi.org/10.1016/j.envexpbot.2019.103892.

Johrenia

Constantinidis, Theophanis, Georgia Kamari, et Dimitrios Phitos. 1997. « A Cytological Study of 28 Phanerogams from the Mountains of SE Sterea Ellas, Greece ». *Willdenowia* 27 (1/2): 121-42.

Evergetis, Epameinondas, Sofia D. Koulocheri, et Serkos A. Haroutounian. 2015. « Exploitation of Apiaceae Family Plants as Valuable Renewable Source of Essential Oils Containing Crops for the Production of Fine Chemicals: Part II ». *Industrial Crops and Products* 64 (février): 59-67. https://doi.org/10.1016/j.indcrop.2014.10.069.

Laserpitium

Conti, Fabio, Fabrizio Bartolucci, Gianluigi Bacchetta, Riccardo Pennesi, Dmitar Lakušić, et Marjan Niketić. 2021. « A taxonomic revision of the Siler montanum group (Apiaceae) in Italy and the Balkan Peninsula ». *Willdenowia* 51 (3): 321-47. https://doi.org/10.3372/wi.51.51301.

Evergetis, Epameinondas, et Serkos A. Haroutounian. 2014. « Exploitation of Apiaceae Family Plants as Valuable Renewable Source of Essential Oils Containing Crops for the Production of Fine Chemicals ». *Industrial Crops and Products* 54 (mars): 70-77. https://doi.org/10.1016/j.indcrop.2014.01.009.

Evergetis, Epameinondas, Antonios Michaelakis, et Serkos A. Haroutounian. 2012. « Essential Oils of Umbelliferae (Apiaceae) Family Taxa as Emerging Potent Agents for Mosquito Control ». In *Integrated Pest Management and Pest Control - Current and Future Tactics*, par Sonia Soloneski, 613-38. https://doi.org/10.5772/1383.

Franzén, R., et L.-Å. Gustavsson. 1983. « Chromosome Numbers in Flowering Plants from the High Mountains of Sterea Ellas, Greece ». *Willdenowia* 13 (1): 101-6.

Reich, Dieter, Walter Gutermann, Katharina Bardy, et al. 2021. « The Type Specimens in Eugen von Halácsy´s Herbarium Graecum ». *Phytotaxa* 493 (1): 1-156. https://doi.org/10.11646/phytotaxa.493.1.1.

Spalik, Krzysztof, Aneta Wojewódzka, Theophanis Constantinidis, Stephen R Downie, Michał Gierek, et Łukasz Banasiak. 2019. « Laserocarpum, a New Genus of Apiaceae Endemic to Greece ». *Acta Soc Bot Pol*, 9.

Ligusticum

Hartvig, P. 1984. « Two New Species of Apiaceae from Greece ». *Willdenowia* 13 (2): 289-93.

Αναγνωστάκης, Σπυρίδων, Ανδρέας Γκανάτσιος, et Κωνσταντίνος Σπανός. 2011. *Το φυσικό περιβάλλον του Ολύμπου & Ολύμπος, αείφορος αναπτύξη - The natural environment of Olympus & Olympus, sustainable development*. Experimental seminar. Κέντρο Περιβαλλοντικής Εκπαίδευσης (Κ.Π.Ε.) Ελασσόνας. http://repository.edulll.gr/edulll/bitstream/10795/2096/2/2096_ekdosi_2011.pdf.

Peucedanum

Hadaček, Franz, and Rosabelle Samuel. 1994. 'Chromosome Counts and Chemotaxonomy in Peucedanum Sect. Peucedanum (Apiaceae, Apioideae) from the Balkan Peninsula'. Willdenowia 24 (1/2): 33–48.

Stevanovic, V., Kit Tan, and G. Iatrou. 2003. 'Distribution of the Endemic Balkan Flora on Serpentine I. - Obligate Serpentine Endemics'. Plant Systematics and Evolution 242 (1–4): 149–70. https://doi.org/10.1007/s00606-003-0044-8.

Pimpinella

Evergetis, Epameinondas, et Serkos A. Haroutounian. 2014. « Exploitation of Apiaceae Family Plants as Valuable Renewable Source of Essential Oils Containing Crops for the Production of Fine Chemicals ». *Industrial Crops and Products* 54 (mars): 70-77. https://doi.org/10.1016/j.indcrop.2014.01.009.

Evergetis, Epameinondas, Antonios Michaelakis, et Serkos A. Haroutounian. 2012. « Essential Oils of Umbelliferae (Apiaceae) Family Taxa as Emerging Potent Agents for Mosquito Control ». In *Integrated Pest Management and Pest Control - Current and Future Tactics*, par Sonia Soloneski, 613-38. https://doi.org/10.5772/1383.

Kougioumoutzis, K., A. Tiniakou, O. Georgiou, and T. Georgiadis. 2015. 'Contribution to the Flora and Biogeography of the Kiklades: Folegandros Island (Kiklades, Greece)'. Edinburgh Journal of Botany 72 (3): 391–412. https://doi.org/10.1017/S0960428615000128.

Skoula, M, C Dal Cin D'Agata, and A Sarpaki. 2009. 'Contribution to the Ethnobotany of Crete, Greece'. Bocconea 23: 479–87.

Yurtseva, O. V., and V. N. Tikhomirov. 1998. 'Morphological Diversity and Taxonomy of the Pimpinella Tragium VILL. Group (Umbelliferae – Apioideae) in the Mediterranean'. Feddes Repertorium 109 (7–8): 479–500. https://doi.org/10.1002/fedr.19981090703.

Scaligeria

Krigas, Nikos, Marina Panagiotidou, et Eleni Maloupa. 2017. « Incorporating biogeographical principles in horticulture: design and creation of the ionian islands unique rock garden in Thessaloniki, Greece. » *Sibbaldia: The Journal of Botanic Garden Horticulture*, 2017, 15 édition.

Siljak-Yakovlev, Sonja, Perla Farhat, Nicolas Valentin, Pepy Bareka, et Georgia Kamari. 2019. « New Estimates of Nuclear DNA Amount for 25 Taxa from Kefallinia Island », 23.

Seseli

Cattaneo, Cristina, and Mauro Grano. 2018. 'Contribution to the Flora of Tilos Island (Dodecanese Islands, Greece)'. *Parnassiana Archives* 6: 41–53.

Evergetis, Epameinondas. 2012. 'Εθνοβοτανική και φυτοχημεία των Umbelliferae (Apiaceae) της Ελλάδας. Μελέτη της σύστασης των αιθερίων ελαίων τους, της βιοδραστικότητας τους και των χημειοταξονομικών τους εφαρμογών - Ethnobotany and phytochemistry of Umbelliferae (Apiaceae) of Greece. Study of the composition of their essential oils, their bioactivity and their chemotaxonomic applications.' PhD in biology, Γεωπονικό Πανεπιστήμιο Αθηνώ. http://dspace.aua.gr/xmlui/bitstream/handle/10329/5717/Evergetis_E.pdf?sequence=3.

Evergetis, Epameinondas, and Serkos A. Haroutounian. 2014. 'Exploitation of Apiaceae Family Plants as Valuable Renewable Source of Essential Oils Containing Crops for the Production of Fine Chemicals'. *Industrial Crops and Products* 54 (March): 70–77. https://doi.org/10.1016/j.indcrop.2014.01.009.

Evergetis, Epameinondas, Antonios Michaelakis, and Serkos A. Haroutounian. 2012. 'Essential Oils of Umbelliferae (Apiaceae) Family Taxa as Emerging Potent Agents for Mosquito Control'. In *Integrated Pest Management and Pest Control - Current and Future Tactics*, by Sonia Soloneski. https://doi.org/10.5772/1383.

Hartvig, P. 1984. « Two New Species of Apiaceae from Greece ». *Willdenowia* 13 (2): 289-93.

Kokkoris, Ioannis, Georgios Dimitrellos, Konstantinos Kougioumoutzis, Ioannis Laliotis, Theodoros Georgiadis, and Argyro Tiniakou. 2014. 'The Native Flora of Mountain Panachaikon (Peloponnese, Greece): New Records and Diversity'. *Journal of Biological Research-Thessaloniki* 21 (1): 9. https://doi.org/10.1186/2241-5793-21-9.

Thamnosciadium

Evergetis, Epameinondas, Antonios Michaelakis, et Serkos A. Haroutounian. 2012. « Essential Oils of Umbelliferae (Apiaceae) Family Taxa as Emerging Potent Agents for Mosquito Control ». In *Integrated Pest Management and Pest Control - Current and Future Tactics*, par Sonia Soloneski, 613-38. https://doi.org/10.5772/1383.

Tzakou, Olga, Konstantinos Lempesis, et Anargyros Loukis. 2010. « Essential Oil Composition of the Endemic Species Thamnosciadium Junceum (Sm.) Hartvig ». *Journal of Essential Oil Research* 22 (3): 257-58. https://doi.org/10.1080/10412905.2010.9700319.

Torilis

Polymenakos, Kostas, Kit Tan, and Vasilis Pantavos. 2024. 'Torilis Samia (Apiaceae) and Ehrharta Erecta (Poaceae), Two New Species for Greece'. *Phytologia Balcanica* 30 (2): 197–202.

Trinia

Franzén, R., et L.-Å. Gustavsson. 1983. « Chromosome Numbers in Flowering Plants from the High Mountains of Sterea Ellas, Greece ». *Willdenowia* 13 (1): 101-6.

Löve, Áskell. 1981. 'Chromosome Number Reports LXXIII'. *Taxon* 30 (4): 829–61.

Apocynaceae

Vincetoxicum

Bourgou, Soumaya, Imtinen Ben Haj Jilani, Olfa Karous, et al. 2021. 'Medicinal-Cosmetic Potential of the Local Endemic Plants of Crete (Greece), Northern Morocco and Tunisia: Priorities for Conservation and Sustainable Exploitation of Neglected and Underutilized Phytogenetic Resources'. *Biology* 10 (12): 1344. https://doi.org/10.3390/biology10121344.

Liede-Schumann, Sigrid, Rizwana Khanum, Abdul Samad Mumtaz, Iulian Gherghel, and Amirhossein Pahlevani. 2016. 'Going West – A Subtropical Lineage (Vincetoxicum, Apocynaceae: Asclepiadoideae) Expanding into Europe'. *Molecular Phylogenetics and Evolution* 94 (January): 436–46. https://doi.org/10.1016/j.ympev.2015.09.021.

Araceae

Arum

Azab, Abdullatif. 2017. 'Arum: A Plant Genus with Great Medicinal Potential'. *European Chemical Bulletin* 6 (2): 59. https://doi.org/10.17628/ecb.2017.6.59-68.

Brandes, Dietmar. 2002. 'Some Remarks on the Flora of Walls and Ruins in Eastern Crete'. *Technical University of Braunschweig*, 18.

Fokialakis, Nikolas, Charles L. Cantrell, Stephen O. Duke, Alexios L. Skaltsounis, and David E. Wedge. 2006. 'Antifungal Activity of Thiophenes from Echinops Ritro'. *Journal of Agricultural and Food Chemistry* 54 (5): 1651–55. https://doi.org/10.1021/jf052702j.

Khabbach, Abdelmajid, Georgios Tsoktouridis, Ioannis Anestis, et al. 2021. 'Exploring the Potential of Neglected Local Endemic Plants of Three Mediterranean Regions in the Ornamental Sector'. Online poster. Colaplamed, 9no Congreso Latinoamericano de Plantas Medicinales. https://colaplamed2021.cedia.edu.ec/dmdocuments/Poster/39-Poster.pdf.

Kite, Geoffrey C., Wilbert L.A. Hetterscheid, Mervyn J. Lewis, et al. 1998. 'Inflorescence Odours and Pollinators of Arum and Amorphophallus (Araceae)'. In *Reproductive Biology*, edited by S.J. Owens and P.J. Rudall. Royal Botanic Gardens. https://florapix.nl/arisaema-l/secure/Literature/Kite%20et%20al.%201998%20-%20Inflorescence%20Odours%20&%20Pollinators%20of%20Arum%20and%20Amorphophallus.pdf.

Krigas, Nikos, Viktoria Menteli, and Despoina Vokou. 2014. 'The Electronic Trade in Greek Endemic Plants: Biodiversity, Commercial and Legal Aspects'. *Economic Botany* 68 (1): 85–95. https://doi.org/10.1007/s12231-014-9264-9.

Krigas, Nikos, Georgios Tsoktouridis, Ioannis Anestis, et al. 2021. 'Exploring the Potential of Neglected Local Endemic Plants of Three Mediterranean Regions in the Ornamental Sector: Value Chain Feasibility and Readiness Timescale for Their Sustainable Exploitation'. *Sustainability* 13 (5): 5. https://doi.org/10.3390/su13052539.

Menteli, Viktoria, Nikos Krigas, Manolis Avramakis, Nicholas Turland, and Despoina Vokou. 2019. 'Endemic Plants of Crete in Electronic Trade and Wildlife Tourism: Current Patterns and Implications for Conservation'. *Journal of Biological Research-Thessaloniki* 26 (1): 10. https://doi.org/10.1186/s40709-019-0104-z.

Rohloff, Jens, Ariaya Hymete, and Yinebeb Tariku. 2013. 'Plant-Derived Natural Products for the Treatment of Leishmaniasis'. In *Studies in Natural Products Chemistry*, edited by Atta-ur- Rahman, vol. 39. Elsevier.

Urru, Isabella, Johannes Stökl, Jeanine Linz, Tamara Krügel, Marcus C. Stensmyr, and Bill S. Hansson. 2010. 'Pollination Strategies in Cretan Arum Lilies'. *Biological Journal of the Linnean Society* 101 (4): 991–1001. https://doi.org/10.1111/j.1095-8312.2010.01537.x.

Biarum

Ayalp, Şükran. 2021. 'Türkiye'de Peyzaj Tasarım ve Uygulamalarında Araceae Cinslerinin Kullanım Olanaklarının Araştırılması - Investigation of the usage possibilities of Araceae genera in landscape design and applications in Turkey'. *Anadolu Orman Araştırmaları Dergisi* 7 (1): 59–94. https://doi.org/10.53516/ajfr.919864.

Brown, N. E. 1880. 'On Some New Aroideae; with Observations on Other Known Forms. -Part I.' *Journal of the Linnean Society of*

London, Botany 18 (109): 242–63. https://doi.org/10.1111/j.1095-8339.1880.tb00650.x.

Constantinidis, Theophanis. 2012. 'Hedysarum Grandiflorum Subsp. Bulgaricum (Leguminosae) and Biarum Ditschianum (Araceae), Two New Records for the Greek Flora'. *Phytologia Balcanica* 18 (2): 155–61.

Krigas, Nikos, Viktoria Menteli, and Despoina Vokou. 2014. 'The Electronic Trade in Greek Endemic Plants: Biodiversity, Commercial and Legal Aspects'. *Economic Botany* 68 (1): 85–95. https://doi.org/10.1007/s12231-014-9264-9.

Krigas, Nikos, Georgios Tsoktouridis, Ioannis Anestis, et al. 2021. 'Exploring the Potential of Neglected Local Endemic Plants of Three Mediterranean Regions in the Ornamental Sector: Value Chain Feasibility and Readiness Timescale for Their Sustainable Exploitation'. *Sustainability* 13 (5): 5. https://doi.org/10.3390/su13052539.

Menteli, Viktoria, Nikos Krigas, Manolis Avramakis, Nicholas Turland, and Despoina Vokou. 2019. 'Endemic Plants of Crete in Electronic Trade and Wildlife Tourism: Current Patterns and Implications for Conservation'. *Journal of Biological Research-Thessaloniki* 26 (1): 10. https://doi.org/10.1186/s40709-019-0104-z.

Panitsa, Maria, Panayiotis Trigas, Dimitrios Kontakos, Anna-Thalassini Valli, et Gregoris Iatrou. 2021. « Natural and cultural heritage interaction: aspects of plant diversity in three East Peloponnesian castles (Greece) and conservation evaluation ». *Plant Biosystems - An International Journal Dealing with all Aspects of Plant Biology* 0 (0): 1-15. https://doi.org/10.1080/11263504.2021.1889701.

Aristolochiaceae

Aristolochia

Brandes, Dietmar. 2002. 'Some Remarks on the Flora of Walls and Ruins in Eastern Crete'. Technical University of Braunschweig, 18.

Constantinidis, Theophanis, Georgia Kamari, et Dimitrios Phitos. 1997. « A Cytological Study of 28 Phanerogams from the Mountains of SE Sterea Ellas, Greece ». *Willdenowia* 27 (1/2): 121-42.

Delitheos, A., E. Tiligada, A. Yannitsaros, et I. Bazos. 1997. « Antiphage Activity in Extracts of Plants Growing in Greece ». *Phytomedicine* 4 (2): 117-24. https://doi.org/10.1016/S0944-7113(97)80055-4.

Fokialakis, Nikolas, Charles L. Cantrell, Stephen O. Duke, Alexios L. Skaltsounis, and David E. Wedge. 2006. 'Antifungal Activity of Thiophenes from Echinops Ritro'. Journal of Agricultural and Food Chemistry 54 (5): 1651–55. https://doi.org/10.1021/jf052702j.

Georgopoulou, C., N. Aligiannis, N. Fokialakis, and S. Mitaku. 2005. 'Acretoside, a New Sucrose Ester from Aristolochia Cretica'. Journal of Asian Natural Products Research 7 (6): 799–803. https://doi.org/10.1080/1028602042000191617.

Rupp, Thomas, Birgit Oelschlägel, Katharina Rabitsch, Hafez Mahfoud, Torsten Wenke, R. Henry L. Disney, Christoph Neinhuis, Stefan Wanke, et Stefan Dötterl. 2021. « Flowers of Deceptive Aristolochia Microstoma Are Pollinated by Phorid Flies and Emit Volatiles Known From Invertebrate Carrion ». *Frontiers in Ecology and Evolution* 9. https://doi.org/10.3389/fevo.2021.658441.

Asparagaceae

Prospero

Hamouche, Yasmina, Nabila Amirouche, Marie-Thérèse Misset, and Rachid Amirouche. 2010. 'Cytotaxonomy of Autumnal Flowering Species of Hyacinthaceae from Algeria'. Plant Systematics and Evolution 285 (3): 177–87. https://doi.org/10.1007/s00606-010-0275-4.

Menteli, Viktoria, Nikos Krigas, Manolis Avramakis, Nicholas Turland, and Despoina Vokou. 2019. 'Endemic Plants of Crete in Electronic Trade and Wildlife Tourism: Current Patterns and Implications for Conservation'. Journal of Biological Research-Thessaloniki 26 (1): 10. https://doi.org/10.1186/s40709-019-0104-z.

Speta, F. 2000. 'Beitrag zur Kenntnis der Gattung Prospero SALISB. (Hyacinthaceae) auf der griechischen Insel Kreta - Contribution to the knowledge of the genus Prospero Salisb. (Hyacinthaceae) on the Greek island of Crete'. Linzer Biol. Beitr. 32 (2): 1323–26.

Tzanoudakis, Dimitris, and Zaharias Kypriotakis. 1998. 'A New Polyploid Scilla (Liliaceae) from the Cretan Area (Greece)'. Folia Geobotanica 33 (1): 103–8. https://doi.org/10.1007/BF02914932.

Valdés, Benito. 2004. 'Some Validations in Liliaceae'. Willdenowia 34 (1): 63–64. https://doi.org/10.3372/wi.34.34104.

Asteraceae

Achillea

Baltisberger, Matthias, and Alex Widmer. 2016. 'Chromosome Numbers and Karyotypes within the Genus Achillea (Asteraceae : Anthemideae)'. Willdenowia 46 (1): 121–35. https://doi.org/10.3372/wi.46.46110.

Dębicz, R., et K. Wróblewska. 2010. « Evaluation of decorative value of six covering perennials. » Zeszyty Naukowe Uniwersytetu Przyrodniczego we Wrocławiu - Rolnictwo 97 (578): 179-88.

Diapoulis, Char. 1959. 'Conservation Measures for the Plants of the Greek Flora'. Revue d'Ecologie, Terre et Vie, Société Nationale de Protection de La Nature Sup: 189–91.

Franzén, Roy. 1986. 'Taxonomy of the Achillea Clavennae Group and the A. Ageratifolia Group (Asteraceae, Anthemideae) on the Balkan Peninsula'. Willdenowia 16 (1): 13–33.

Gilman, Edward F., et Terry Delvalle. 1999. « Achillea spp. - Fact sheet ». 11. Institute of Food and Agricultural Sciences, University of Florida. http://hort.ufl.edu/shrubs/ACHSPPA.PDF.

Grigoriadou, Katerina, Nikos Krigas, et Eleni Maloupa. 2011. « GIS-Facilitated in Vitro Propagation and Ex Situ Conservation of Achillea Occulta ». Plant Cell, Tissue and Organ Culture (PCTOC) 107 (3): 531-40. https://doi.org/10.1007/s11240-011-0004-x.

Hurrell, Julio Alberto. 2017. Plantas cultivadas de la Argentina: Asteráceas(=compuestas). Plantas cultivadas de la Argentina. Buenos Aires: Hemisferio Sur.

Krigas, Nikos, Viktoria Menteli, et Despoina Vokou. 2014. « The Electronic Trade in Greek Endemic Plants: Biodiversity, Commercial and Legal Aspects ». Economic Botany 68 (1): 85-95. https://doi.org/10.1007/s12231-014-9264-9.

Kundakovic, T., N. Fokialakis, N. Kovacevic, et I. Chinou. 2007. « Essential Oil Composition of Achillea Lingulata and A. Umbellata ». Flavour and Fragrance Journal 22 (3): 184-87. https://doi.org/10.1002/ffj.1778.

Maffei, M., M. Mucciarelli, et S. Scannerini. 1994. « Essential Oils from Achillea Species of Different Geographic Origin ». Biochemical Systematics and Ecology 22 (7): 679-87. https://doi.org/10.1016/0305-1978(94)90054-X.

Magiatis, Prokopios, Alexios-Leandros Skaltsounis, Ioanna Chinou, et Serkos A. Haroutounian. 2002. « Chemical Composition And In-Vitro Antimicrobial Activity Of The Essential Oils Of Three Greek Achillea Species ». Zeitschrift Für Naturforschung C 57 (3-4): 287-90. https://doi.org/10.1515/znc-2002-3-415.

Maloupa, E., D. Zervaki, K. Grigoriadou, and K. Papanastasi. 2004. 'The Development of a Native Plant Collection Nursery in the Kroussia Balkan Botanic Garden'. Scripta Botanica Belgica 29: 15–20.

Maloupa, E., K. Grigoriadou, D. Zervaki, and K. Papanastassi. 2005. 'Management of the Balkan Native Flora for Sustainable Floricultural Commercial Use'. Acta Horticulturae, no. 683 (June): 189–96. https://doi.org/10.17660/ActaHortic.2005.683.21.

Murray, Katie A. 2004. « The Marriage of Form and Function in Contemporary Kitchen Gardens ». University of British Columbia. https://doi.org/10.14288/1.0099767.

Pavlides, G. 1997. 'The Flora of Prespa National Park with Emphasis on Species of Conservation Interest'. In Lake Prespa, Northwestern Greece, edited by Alain J. Crivelli and George Catsadorakis. Springer Netherlands. https://doi.org/10.1007/978-94-011-5180-1_3.

Radulović, Niko S., Milan S. Dekić, Pavle J. Ranđelović, Nikola M. Stojanović, Aleksandra R. Zarubica, et Zorica Z. Stojanović-Radić. 2012. « Toxic Essential Oils: Anxiolytic, Antinociceptive and Antimicrobial Properties of the Yarrow Achillea Umbellata Sibth. et Sm. (Asteraceae) Volatiles ». Food and Chemical Toxicology 50 (6): 2016-26. https://doi.org/10.1016/j.fct.2012.03.047.

Radulovic, Niko, Bojan Zlatković, Radosav Palic, et Gordana Stojanovic. 2007. « Chemotaxonomic Significance of the Balkan Achillea Volatiles ». Natural Product Communications 2 (4): 1934578X0700200. https://doi.org/10.1177/1934578X0700200417.

Shah, Rahul M., Abhishek Patel, Mamta Shah, et Bela Peethambaran. 2015. « Anti-Acne Activity of Achillea Moonshine Petroleum Ether Extract ». Journal of Medicinal Plants Research 9 (27): 755-63. https://doi.org/10.5897/JMPR2015.5792.

Shah, Rahul M., Tejal Patel, Cristina Maria Tettamanzi, Jesse Rajan, Mamta Shah, et Bela Peethambaran. 2016. « Isolation of a Novel Piperidide from Achillea Moonshine Using Bioactivity Guided Fractionation for the Treatment of Acne ». Journal of Medicinal Plants Research 10 (30): 495-504. https://doi.org/10.5897/JMPR2016.6157.

Tzakou, Olga, et Anargyros Loukis. 2009. « Chemical composition of the essential oil of Achillea umbellata growing in Greece ». Natural Product Research 23 (3): 264-70. https://doi.org/10.1080/14786410801997166.

Valant-Vetschera, Karin M, et Eckhard Wollenweber. 2001. « Exudate Flavonoid Aglycones in the Alpine Species of Achillea Sect. Ptarmica: Chemosystematics of A. Moschata and Related Species (Compositae–Anthemideae) ». Biochemical Systematics and Ecology 29 (2): 149-59. https://doi.org/10.1016/S0305-1978(00)00033-8.

Valant-Vetschera, Karin M., and Eckhard Wollenweber. 1996. 'Comparative Analysis of Leaf Exudate Flavonoids in Achillea Subsect. Filipendulinae'. Biochemical Systematics and Ecology 24 (5): 435–46. https://doi.org/10.1016/0305-1978(96)00039-7.

Vladimirov, Vladimir, Feruzan Dane, Vlado Matevski, and Kit Tan. 2016. 'New Floristic Records in the Balkans: 29'. Phytologia Balcanica: International Journal of Balkan Flora and Vegetation. Phytologia Balcanica: International Journal of Balkan Flora and Vegetation 22 (1): 93–123.

Wollenweber, E., K. M. Valant-Vetschera, S. Ivancheva, et B. Kuzmanov. 1986. « Flavonoid Aglycones from the Leaf Surfaces of Some Achillea Species ». Phytochemistry 26 (1): 181-82. https://doi.org/10.1016/S0031-9422(00)81506-X.

Παππας, Ιωαννις Οδυσσεας. 2021. 'Συγκριση υπογειας και υπαιθριας χαραξης για την οδικη συνδεση Μελισσουργοι-Θεοδωριανα - Comparison of underground and outdoor layout for the road connection Melissourgoi - Theodoriana'. Bachelor Thesis, ΕΘΝΙΚΟ ΜΕΤΣΟΒΙΟ ΠΟΛΥΤΕΧΝΕΙΟ. https://dspace.lib.ntua.gr/xmlui/bitstream/handle/123456789/53526/Pappas_diplomatiki.pdf?sequence=1.

Anthemis

Bazos, Ioannis, Ioannis P. Kokkoris, and Panayotis Dimopoulos. 2021. 'Diversity of Halophytes and Salt Tolerant Plants at the Species-, Habitats- and High-Rank Syntaxa Level in Greece'. In Handbook of Halophytes, edited by Marius-Nicusor Grigore. Springer International Publishing. https://doi.org/10.1007/978-3-030-57635-6_26.

Bergmeier, Erwin, and Panayotis Dimopoulos. 2003. 'The Vegetation of Islets in the Aegean and the Relation between the Occurrence of Islet Specialists, Island Size, and Grazing'. Phytocoenologia 33 (2–3): 447–74. https://doi.org/10.1127/0340-269X/2003/0033-0447.

Biel, Burkhard, and Kit Tan. 2024. 'Recent Progress on the Flora of Milos (Kiklades, S Aegean, Greece)'. Phytologia Balcanica 30 (2): 189–92.

Cattaneo, Cristina, and Mauro Grano. 2021. Kasos: An Unexpected Island. Floristic and Ecological Analysis of Kasos Island (SE Aegean, Dodecanese, Greece), with Noteworthy Floristic Additions. 28.

Christodoulakis, Dimitrios, Theodoros Georgiadis, E. Economidou, Gregoris Iatrou, and Dimitrios Tzanoudakis. 1990. 'Flora und

Vegetation der Dionysaden-Inseln (Südägäis, Griechenland) - Flora and vegetation of the Dionysad Islands (South Aegean, Greece)'. Willdenowia 19: 425–43.

Dimitrellos, Georgios, and Dimitrios Christodoulakis. 1999. 'The Phytogeographical Distribution Patterns of the Flora of Mt Timfristos (N.W. Sterea Ellas, Greece)'. Flora Mediterranea 9: 215–29.

Franzén, Roy. 1986. 'Anthemis Cretica (Asteraceae) and Related Species in Greece'. Willdenowia 16 (1): 35–45.

Georgiou, Ourania, Maria Panitsa, et Dimitrios Tzanoudakis. 2006. « Anthemis scopulorum (Asteraceae), an "Islet Specialist" Endemic to the Aegean Islands (Greece) ». *Willdenowia* 36 (1): 339-49.

Goula, Katerina, and Theophanis Constantinidis. 2021. 'Taxonomic Diversity and Karyology of Anthemis Rigida (Anthemideae, Asteraceae) in the Aegean, Greece'. Phytotaxa 484 (1): 1. https://doi.org/10.11646/phytotaxa.484.1.7.

Goula, Katerina, and Theophanis Constantinidis. 2023. 'Anthemissect.Hiorthia (Asteraceae) on Kriti Island, Greece: High Ploidy Levels and a New Species'. PhytoKeys 229 (July): 113. https://doi.org/10.3897/phytokeys.229.102703.

Goula, Katerina, Konstantinos Touloumis, Panayotis Dimopoulos, and Theophanis Constantinidis. 2022. 'A Morphometric and Karyological Study of the Anthemis Macedonica Group (Asteraceae, Anthemideae) Reveals a New Species from Greece'. Plants 11 (21): 21. https://doi.org/10.3390/plants11213006.

Greuter, Werner, Regina Pleger, and Thomas Raus. 1983. 'The Vascular Flora of the Karpathos Island Group (Dodecanesos, Greece). A Preliminary Checklist'. Willdenowia 13 (1): 43–78.

Höner, D., and Werner Greuter. 1988. 'Plant Population Dynamics and Species Turnover on Small Islands near Karpathos (South Aegean, Greece)'. Vegetatio 77: 129–37.

Kougioumoutzis, Konstantinos, Argyro Tiniakou, Ourania Georgiou, and Theodoros Georgiadis. 2012. 'Contribution to the Flora of the South Aegean Volcanic Arc: Anafi Island (Kiklades, Greece)'. Willdenowia 42 (1): 127–41. https://doi.org/10.3372/wi.42.42115.

Krigas, Nikos, Georgios Tsoktouridis, Ioannis Anestis, et al. 2021. 'Exploring the Potential of Neglected Local Endemic Plants of Three Mediterranean Regions in the Ornamental Sector: Value Chain Feasibility and Readiness Timescale for Their Sustainable Exploitation'. *Sustainability* 13 (5): 5. https://doi.org/10.3390/su13052539.

Löve, Áskell. 1982. « IOPB Chromosome Number Reports LXXVII ». *Taxon* 31 (4): 761-77.

Maloupa, Eleni, Nikos Krigas, Katerina Grigoriadou, Diamanto Lazari, and Georgios Tsoktouridis. 2008. Conservation Strategies for Native Plant Species and Their Sustainable Exploitation: Case of the Balkan Botanic Garden of Kroussia, N. Greece. 21.

Pasta, S., A. Perez-Graber, L. Fazan, and B De Montmollin. 2017. The Top 50 Mediterranean Island Plants, Update 2017. IUCN/SSC/Mediterranean Plant Specialist Group. https://top50.iucn-mpsg.org/book.

Raab-Straube, Eckhard Von, and Thomas Raus. 2016. 'Euro Med-Checklist Notulae, 6'. Willdenowia 46 (3): 423–42. https://doi.org/10.3372/wi.46.46310.

Reeves, Roger D., Maria Aloupi, Emmanouil I. Daftsis, John A. Stratis, Petros Mastoras, and Panayiotis G. Dimitrakopoulos. 2022. 'Biogeochemical Aspects of the Serpentines of Rhodes (Greece) and Cyprus'. Plant and Soil 472 (1–2): 491–508. https://doi.org/10.1007/s11104-021-05265-5.

Saroglou, Vasiliki, Nikos Dorizas, Zacharias Kypriotakis, et Helen D. Skaltsa. 2006. « Analysis of the Essential Oil Composition of Eight Anthemis Species from Greece ». *Journal of Chromatography A* 1104 (1): 313-22. https://doi.org/10.1016/j.chroma.2005.11.087.

Stephanaki, Dimitri. 2009. 'Βοτανικοι πηποι στην Ευρωπη: το παραδειγμα της Ροδου - Botanical gardens in Europe: the example of Rhodes'. Master of Science, T.E.I. Kritis.

Strid, Arne. 2015. 'Reliquiae Runemarkianae. Chromosome Numbers of Angiosperms from the Aegean Islands'. Phytologia Balcanica 21 (3): 245–93.

Thanos, Costas A., C Fournaraki, Kyriacos Georghiou, Panayotis Dimopoulos, and Erwin Bergmeier. 2008. The Establishment, Monitoring and Management of a Pilot Network of Micro-Reserves in Western Crete for the Conservation of European Threatened Plants (CRETAPLANT Project, EU-LIFE).

Thanos, Costas A., Christini Fournaraki, Kyriacos Georghiou, and Panayotis Dimopoulos. 2013. 'PMRs in Western Crete'. In Plant Micro-Reserves: From Theory to Practice. Utopia Publishing.

Thanos, Costas A., Apostolis Kaltsis, and Katerina Koutsovoulou. 2013. 'PMRs as Field Laboratories for Scientific Research'. In Plant Micro-Reserves: From Theory to Practice. Utopia Publishing.

Turland, Nicholas J. 2008. 'Anthemis Samariensis (Asteraceae, Anthemideae), a New Species from the Mountains of W Kriti (Greece)'. Willdenowia 38 (1): 61. https://doi.org/10.3372/wi.38.38103.

Wan, Xia, and Li-Bing Zhang. 2022. 'Global New Taxa of Vascular Plants Published in 2021'. Biodiversity Science 30 (8): 22116.

Κοντού, Αναστασία. 2018. 'Απειλούμενα είδη της Ρόδου: σχεδιασμός εκπαιδευτικού υλικού για το νηπιαγωγείο - Endangered species of Rhodes: design of educational material for the kindergarten'. Postgraduate, Panepistimio Aigaiou. http://hellanicus.lib.aegean.gr/handle/11610/20700.

Bellis

Egli, B. 1984. 'Bellis Longifolia, Ein Kretischer Endemit - Bellis Longifolia, an Endemic from Crete'. *Botanica Helvetica* (Teufen) 94 (1): 61–65.

Fiz, Omar, Virginia Valcárcel, and Pablo Vargas. 2002. 'Phylogenetic Position of Mediterranean Astereae and Character Evolution of Daisies (Bellis, Asteraceae) Inferred from nrDNA ITS Sequences'. *Molecular Phylogenetics and Evolution* 25 (1): 157–71. https://doi.org/10.1016/S1055-7903(02)00228-2.

Stavropoulou, M. I., N. Aligiannis, E. Kalpoutzakis, et al. 2012. 'Screeninig for Phytotoxicity Crude Exctracts from the Flora of Crete and Phytochemical Investigation of Bellis Longifolia'. *Planta*

Medica 78 (11): PD147. https://doi.org/10.1055/s-0032-1320505.

Stavropoulou, Maria I., Apostolis Angelis, Nektarios Aligiannis, et al. 2017. 'Phytotoxic Triterpene Saponins from Bellis Longifolia, an Endemic Plant of Crete'. *Phytochemistry* 144 (December): 71–77. https://doi.org/10.1016/j.phytochem.2017.08.019.

Cardus

Tan, Kit, and Aris Zografidis. 2014. 'On the Identity of Carduus Euboicus (Asteraceae) from Mt Dirphys, Evvia, Greece'. *Phytotaxa* 161 (3): 194. https://doi.org/10.11646/phytotaxa.161.3.2.

Carlina

Apparao, M., A. Kjær, O. Olsen, E. Venkata Rao, K. W. Rasmussen, and H. Sørensen. 1981. 'Alliin in the Garlicky Taxon Adenocalymma Alliaceum (Bignoniaceaei)'. *Phytochemistry* 20 (4): 822–23. https://doi.org/10.1016/0031-9422(81)85185-0.

Badry, Mohamed O., Ahmed K. Osman, and Ahmed Elkordy. 2020. 'Pollen Diversity in the Genus Carlina L. (Subtribe Carlininae, Compositae) and Its Systematic Significance'. *Review of Palaeobotany and Palynology* 279 (August): 104243. https://doi.org/10.1016/j.revpalbo.2020.104243.

Barclay, Colville. 1986. 'Crete: Checklist of the Vascular Plants'. *Englera*, no. 6: I–138. https://doi.org/10.2307/3776733.

Bohlmann, F., A. Schuster, and H. Meusel. 1981. 'Carlina Oxide Derivative from Carlina Diae'. *Phytochemistry* 20 (4): 823–24.

Goulimis, C. 1958. *Report on Species of Plants Requiring Protection in Greece and Measures for Securing Their Protection*. Athens. http://documents.irevues.inist.fr/bitstream/handle/2042/59422/LATERREETLAVIE_1959_Sup_168.pdf?sequence=1.

Greuter, Werner, Regina Pleger, and Thomas Raus. 1983. 'The Vascular Flora of the Karpathos Island Group (Dodecanesos, Greece). A Preliminary Checklist'. *Willdenowia* 13 (1): 43–78.

Grigoriadou, Katerina, Virginia Sarropoulou, Nikos Krigas, Eleni Maloupa, and Georgios Tsoktouridis. 2020. 'GIS-Facilitated Effective Propagation Protocols of the Endangered Local Endemic of Crete Carlina Diae (Rech. f.) Meusel and A. Kástner (Asteraceae): Serving Ex Situ Conservation Needs and Its Future Sustainable Utilization as an Ornamental'. *Plants* 9 (11): 11. https://doi.org/10.3390/plants9111465.

Krigas, Nikos, Georgios Tsoktouridis, Ioannis Anestis, et al. 2021. 'Exploring the Potential of Neglected Local Endemic Plants of Three Mediterranean Regions in the Ornamental Sector: Value Chain Feasibility and Readiness Timescale for Their Sustainable Exploitation'. *Sustainability* 13 (5): 5. https://doi.org/10.3390/su13052539.

Meusel, Hermann, and Arndt Kästner. 2013. *Lebensgeschichte der Gold- und Silberdisteln Monographie der mediterran-mitteleuropäischen Compositen-Gattung Carlina: Band II: Artenvielfalt und Stammesgeschichte der Gattung - Life history of the gold and silver thistles Monograph of the Mediterranean-Central European composite genus Carlina: Volume II: Species diversity and phylogenetic history of the genus*. Springer-Verlag.

Montmollin, Bertrand de, and Grigoris A. Iatrou. 1995. 'Knowledge and conservation of the flora of Kriti Island - Connaissance et conservation de la flore de l'île de Crète'. *Ecologia Mediterranea* 21 (1): 173–84. https://doi.org/10.3406/ecmed.1995.1765.

Carthamus

DiTomaso, Joseph M. 2011. « Invasive Plant Threats and Prevention Approaches in the Asia-Pacific Region and United States », nº 978: 11.

Garnatje, Teresa, Sonia Garcia, Roser Vilatersana, et Joan Valles. 2006. « Genome Size Variation in the Genus Carthamus (Asteraceae, Cardueae): Systematic Implications and Additive Changes During Allopolyploidization ». *Annals of Botany* 97 (3): 461-67. https://doi.org/10.1093/aob/mcj050.

Rejmánek, Marcel. 2007. Review of *Review of Weeds of California and Other Western States. Two volumes*, par Joseph M. DiTomaso et Evelyn A. Healy. *Madroño* 54 (4): 361-63.

Centaurea

Arianoutsou, M, D Kazanis, and V Varela. 2007. 'Mapping the Post-Fire Resilience of Mediterranean Pine Forests: The Case of Sounion National Park, Greece'. CIHEAM Options Méditerranéennes, CIHEAM Options Méditerranéennes, vol. 75: 25–31.

Armağan, Metin, and Tuna Uysal. 2018. 'Centaurea Kirmacii (Asteraceae), a New Species from Southwestern Anatolia, Turkey'. Phytotaxa 362 (2): 233. https://doi.org/10.11646/phytotaxa.362.2.10.

Baliousis, Evagelos. 2018. 'The Flora of Mt Imittos (Sterea Ellas, Greece): Checklist, New Records, Analysis and Phytogeographical Aspects'. Flora Mediterranea 28: 99–110. https://doi.org/10.7320/FlMedit28.099.

Ben Jemia, Mariem, Carmen Formisano, Svetlana Bancheva, Maurizio Bruno, and Felice Senatore. 2012. 'Chemical Composition of the Essential Oils of Centaurea Formanekii and C. Orphanidea Ssp. Thessala, Growing Wild in Greece'. Natural Product Communications 7 (8): 1934578X1200700. https://doi.org/10.1177/1934578X1200700830.

Bergmeier, Erwin, and Arne Strid. 2019. 'Centaurea Devasiana – a New Species from Prespa, NW Greece'. Phytologia Balcanica 25 (3): 281–86.

Carlström, A. 1986. 'New Taxa and Notes from the SE Aegean Area and SW Turkey'. Willdenowia 16: 73–78.

Cattaneo, Cristina, and Mauro Grano. 2015. 'New Contribution on the Vascular Flora of the Aegean Island of Chalki (Archipelago of Rhodes, Aegean Sea)'. Biodiversity Journal 6 (4): 773–88.

Chiarucci, A., and T. Constantinidis. 2004. 'A Survey of the Ultramafic Flora and Vegetation of Mt. Gerania, Greece.' Ultramafic Rocks: Their Soils, Vegetation and Fauna. Proceedings of the Fourth International Conference on Serpentine Ecology, Cuba, 21-26 April, 2003 ., 161–69.

Constantinidis, Theophanis, and D. Vassiliades. 1996. 'Centaurea Musarum (Compositae): The Rediscovery of a Rare Endemic Species'. Botanika Chronika 12. https://www.researchgate.net/profile/Theophanis-Constantinidis/publication/281686758_Centaurea_musarum_Co

mpositae_the_rediscovery_of_a_rare_endemic_species/links/55f4a86808ae1d980394c05b/Centaurea-musarum-Compositae-the-rediscovery-of-a-rare-endemic-species.pdf.

Constantinidis, Theophanis, Georgia Kamari, and Dimitrios Phitos. 1997. 'A Cytological Study of 28 Phanerogams from the Mountains of SE Sterea Ellas, Greece'. Willdenowia 27 (1/2): 121–42.

Constantinidis, Theophanis. 1999. 'Dianthus Haematocalyx Subsp. Phitosianus (Caryophyllaceae), a New Serpentine Endemic from Greece'. Phyton (Horn, Austria) 39 (2): 277–91.

Council Directive 92/43/EEC of 21 May 1992 on the Conservation of Natural Habitats and of Wild Fauna and Flora, 31992L0043 7 (1992).

Cousturier, MM. Paul, et Michel Gandoger. 1916. « Herborisations en Crète (1913–1914) ». Bulletin de la Société Botanique de France 63 (1-4): 1-15. https://doi.org/10.1080/00378941.1916.10835941.

Dimopoulos, Panayotis, Karle V. Sýkora, Ladislav Mucina, and Theodoros Georgiadis. 1997. 'The High-Rank Syntaxa of the Rock-Cliff and Scree Vegetation of the Mainland Greece and Crete'. Folia Geobotanica et Phytotaxonomica 32 (3): 313–34. https://doi.org/10.1007/BF02804010.

Dural, Huseyin, Yavuz Bagci, Kuddisi Ertugrul, et al. 2003. 'Essential Oil Composition of Two Endemic Centaurea Species from Turkey, Centaurea Mucronifera and Centaurea Chrysantha, Collected in the Same Habitat'. Biochemical Systematics and Ecology 31 (12): 1417–25. https://doi.org/10.1016/S0305-1978(03)00128-5.

Fournaraki, Christini, and Panagiota Gotsiou. 2007. 'The Significance of the Flora from the Lefka Ori (White Mountains) in Crete, Greece and Activities Undertaken for Its Conservation'. ENSC News.

Gamal-Eldin, Elsayeda, and G. Wagenitz. 1984. 'Eine Neue Centaurea-Art Der Sektion Phalolepis (Compositae) Aus Nordgriechenland (Chalkidike) - A New Species of Centaurea of the Phalolepis Section (Compositae) from Northern Greece (Chalkida)'. Willdenowia 13 (2): 323–27.

Georgiadis, T., and D. Phitos. 1979. 'A new Centaurea from Mount Olympus (Greece) of the section Philepsis (Cass.) DC. Centaurea litochorea sp. nov'. Biologie et ecologie mediterraneenne 5 (1): 31–33.

Georgiadis, Theodoros, Georgios Dimitrellos, and Eugenia Routsi. 1996. 'Centaurea Messenicolasiana (Asteraceae), A New Species of C. Sect. Phalolepis (Cass.) DC. from Greece'. Willdenowia 25 (2): 561–69.

Georgiadis, Theodoros. 1979. 'Centaurea Musakii: A New Species from Thessalia (Greece)'. Bot. Not. 132 (3): 311–12.

Georgiadou, Evanthia, Helen Skaltsa, Diamanto Lazari, Begoña Garcia, and Catherine Harvala. 2000. 'A Novel Eudesmanolide from Centaurea Thessala Hausskn. Ssp. Drakiensis (Freyn & Sint.) Georg'. Natural Product Letters 14 (3): 167–73. https://doi.org/10.1080/10575630008041227.

Gerasimidis, Achilles, and Georgios Korakis. 2009. 'Contribution to the Study of the Flora of Mount Mitsikeli, NW Greece'. Flora Mediterranea, 24.

Goulimis, C. 1958. « Report on species of plants requiring protection in Greece and measures for securing their protection ». Athens. http://documents.irevues.inist.fr/bitstream/handle/2042/59422/LATERREETLAVIE_1959_Sup_168.pdf?sequence=1.

Goulimis, C. 1958. Report on Species of Plants Requiring Protection in Greece and Measures for Securing Their Protection. Athens. http://documents.irevues.inist.fr/bitstream/handle/2042/59422/LATERREETLAVIE_1959_Sup_168.pdf?sequence=1.

Gousiadou, C., and H. Skaltsa. 2003. 'Secondary Metabolites from Centaurea Orphanidea'. Biochemical Systematics and Ecology 31 (4): 389–96. https://doi.org/10.1016/S0305-1978(02)00162-X.

Greuter, Werner, and Eckhard Von Raab-Straube. 2007. 'Euro+Med Notulae, 3'. Willdenowia 37 (1): 139–89. https://doi.org/10.3372/wi.37.37107.

Greuter, Werner. 2003. 'The Euro Med Treatment of Cardueae (Compositae) — Generic Concepts and Required New Names'. Willdenowia 33 (1): 49–61. https://doi.org/10.3372/wi.33.33104.

Guinochet, M., and J. Foissac. 1962. 'Sur les Caryotypes de quelques espèces du genre Centaurea L. et leur signification taxonomique - On the karyotype of some species of the genus Centaurea L. and their taxonomic significance'. Bulletin de la Société Botanique de France 109 (sup2): 373–89. https://doi.org/10.1080/00378941.1962.10838114.

Hayek, August v. 1914. 'Neue Orientalische Pflanzenarten - New Oriental Plant Species'. Österreichische Botanische Zeitschrift 64 (8): 358–60.

Hilpold, A., N. Garcia-Jacas, R. Vilatersana, and A. Susanna. 2014. 'Notas Taxonómicas y Nomenclaturales En Centaurea: Propuesta de Clasificación, Descripción de Secciones y Subsecciones Nuevas, y Lista de Especies de Una Sección Centaurea Redefinida - Taxonomical and Nomenclatural Notes on Centaurea: A Proposal of Classification, a Description of New Sections and Subsections, and a Species List of the Redefined Section Centaurea'. Collectanea Botanica 33 (December): 001. https://doi.org/10.3989/collectbot.2013.v33.001.

Hronová-Šafářová, Lucie. 2013. « Plant species of the city park in Olomouc in 19th century ». In Acta Pruhoniciana. 103. Department of Garden Art and Landscape Design, Faculty for Horticulture, Mendel University in Brno, Zem. https://www.vukoz.cz/acta/dokumenty/acta_103/Acta-103_komplet-cz.pdf#page=30.

Iliadou, Eleni, Ioannis Bazos, Konstantinos Kougioumoutzis, et al. 2020. 'Taxonomic and Phylogenetic Diversity Patterns in the Northern Sporades Islets Complex (West Aegean, Greece)'. Plant Systematics and Evolution 306 (2): 28. https://doi.org/10.1007/s00606-020-01660-0.

Jacas, Núria Garcia, and Alfonso Susanna de la Serna. 1992. 'Karyological Notes onCentaurea Sect.Acrocentron (Asteraceae)'.

Plant Systematics and Evolution 179 (1–2): 1–18. https://doi.org/10.1007/BF00938015.

Kafyri, Andriani, Tasos Hovardas, and Konstantinos Poirazidis. 2012. 'Determinants of Visitor Pro-Environmental Intentions on Two Small Greek Islands: Is Ecotourism Possible at Coastal Protected Areas?' Environmental Management 50 (1): 64–76. https://doi.org/10.1007/s00267-012-9856-z.

Kalpoutzakis, Eleftherios, and Theophanis Constantinidis. 2004. 'A New Species of Centaurea (Sect. Phalolepis, Compositae: Cardueae) from Eastern Peloponnisos, Greece'. Botanical Journal of the Linnean Society 146 (3): 375–83. https://doi.org/10.1111/j.1095-8339.2004.00344.x.

Kalpoutzakis, Eleftherios, et Theophanis Constantinidis. 2004. « A new species of Centaurea (sect. Phalolepis, Compositae: Cardueae) from eastern Peloponnisos, Greece ». Botanical Journal of the Linnean Society 146 (3): 375-83. https://doi.org/10.1111/j.1095-8339.2004.00344.x.

Kamari, Georgia, Dimitrios Phitos, Britt Snogerup, and Sven Snogerup. 1988. 'Flora and Vegetation of Yioura, N Sporades, Greece'. Willdenowia 17 (1/2): 59–85.

Karetsos, George, Alexandra D. Solomou, Panayiotis Trigas, and Konstantinia Tsagari. 2018. 'The Vascular Flora of Mt. Oiti National Park and the Surrounding Area in Greece'. Journal of Forest Science 64 (No. 10): 435–54. https://doi.org/10.17221/65/2018-JFS.

Kougioumoutzis, Konstantinos, Panayiota Kotsakiozi, Efthalia Stathi, Panayiotis Trigas, and Aristeidis Parmakelis. 2021. 'Conservation Genetics of Four Critically Endangered Greek Endemic Plants: A Preliminary Assessment'. Diversity 13 (4): 4. https://doi.org/10.3390/d13040152.

Koukoulitsa, Ekaterini, Helen Skaltsa, Anastasia Karioti, Costas Demetzos, et Kostas Dimas. 2002. « Bioactive Sesquiterpene Lactones from Centaurea Species and their Cytotoxic/Cytostatic Activity Against Human Cell Lines in vitro ». Planta Medica 68 (7): 649-52. https://doi.org/10.1055/s-2002-32893.

Koukoulitsa, Ekaterini, Helen Skaltsa, Anastasia Karioti, Costas Demetzos, and Kostas Dimas. 2002. 'Bioactive Sesquiterpene Lactones from Centaurea Species and their Cytotoxic/Cytostatic Activity Against Human Cell Lines in vitro'. Planta Medica 68 (7): 649–52. https://doi.org/10.1055/s-2002-32893.

Krigas, N., V. Menteli, and D. Vokou. 2016. 'Analysis of the Ex Situ Conservation of the Greek Endemic Flora at National European and Global Scales and of Its Effectiveness in Meeting GSPC Target 8'. Plant Biosystems - An International Journal Dealing with All Aspects of Plant Biology 150 (3): 573–82. https://doi.org/10.1080/11263504.2014.988194.

Krigas, Nikos, Eleftherios Karapatzak, Marina Panagiotidou, et al. 2022. 'Prioritizing Plants around the Cross-Border Area of Greece and the Republic of North Macedonia: Integrated Conservation Actions and Sustainable Exploitation Potential'. Diversity 14 (7): 7. https://doi.org/10.3390/d14070570.

Krigas, Nikos, Georgios Tsoktouridis, Ioannis Anestis, et al. 2021. 'Exploring the Potential of Neglected Local Endemic Plants of Three Mediterranean Regions in the Ornamental Sector: Value Chain Feasibility and Readiness Timescale for Their Sustainable Exploitation'. *Sustainability* 13 (5): 5. https://doi.org/10.3390/su13052539.

Krigas, Nikos, Marina Panagiotidou, and Eleni Maloupa. 2017. 'Incorporating Biogeographical Principles in Horticulture: Design and Creation of the Ionian Islands Unique Rock Garden in Thessaloniki, Greece'. Sibbaldia: The Journal of Botanic Garden Horticulture 0 (15): 129–46.

Kyriakopoulos, Ch., Pepy Bareka, et Georgia Kamari. 2016. « Karyological Data of Some Endemic Taxa from Mt Taigetos, Greece ». Flora Mediterranea 26 (décembre). https://doi.org/10.7320/FlMedit26.224.

Lazari, Diamanto M., Helen D. Skaltsa, and Theophanis Constantinidis. 2000. 'Volatile Constituents of Centaurea Pelia DC., C. Thessala Hausskn. Subsp. Drakiensis (Freyn & Sint.) Georg. and C. Zuccariniana DC. from Greece'. Flavour and Fragrance Journal 15 (1): 7–11. https://doi.org/10.1002/(SICI)1099-1026(200001/02)15:1%253C7::AID-FFJ860%253E3.0.CO;2-3.

Lazari, Diamanto M., Helen D. Skaltsa, et Theophanis Constantinidis. 1999. « Volatile Constituents of Centaurea Raphanina Sm. Subsp. Mixta (DC.) Runemark and C. Spruneri Boiss. & Heldr. (Asteraceae), Growing Wild in Greece ». Flavour and Fragrance Journal 14 (6): 415-18. https://doi.org/10.1002/(SICI)1099-1026(199911/12)14:6<415::AID-FFJ857>3.0.CO;2-9.

Liveri, Eleni, Andrew A. Crowl, Evgeny Mavrodiev, Hasan Yıldırım, Georgia Kamari, and Nico Cellinese. 2020. 'Another Piece of the Puzzle, Another Brick in the Wall: The Inevitable Fate of Campanula Section Quinqueloculares (Campanulaceae: Campanuloideae)'. TAXON 69 (6): 1239–58. https://doi.org/10.1002/tax.12372.

López-Vinyallonga, Sara, Jordi López-Pujol, Theophanis Constantinidis, Alfonso Susanna, and Núria Garcia-Jacas. 2015. 'Mountains and Refuges: Genetic Structure and Evolutionary History in Closely Related, Endemic Centaurea in Continental Greece'. Molecular Phylogenetics and Evolution 92 (November): 243–54. https://doi.org/10.1016/j.ympev.2015.06.018.

Löve, Áskell. 1981. 'Chromosome Number Reports LXXIII'. Taxon 30 (4): 829–61.

Löve, Askell. 1983. 'IOPB Chromosome Number Reports LXXVIII'. Taxon 32 (1): 138–41.

Löve, Áskell. 1988. 'Chromosome Number Reports XCIX'. Taxon 37 (2): 396–99.

Löve, Áskell. 1988. « Chromosome Number Reports XCIX ». Taxon 37 (2): 396-99.

Maloupa, E., K. Grigoriadou, D. Zervaki, and K. Papanastassi. 2005. 'Management of the Balkan Native Flora for Sustainable Floricultural Commercial Use'. Acta Horticulturae, no. 683 (June): 189–96. https://doi.org/10.17660/ActaHortic.2005.683.21.

Maloupa, Eleni, Nikos Krigas, Katerina Grigoriadou, Diamanto Lazari, and Georgios Tsoktouridis. 2008. Conservation Strategies for Native Plant Species and Their Sustainable Exploitation: Case of the Balkan Botanic Garden of Kroussia, N. Greece. 21.

Meliadis, Ioannis, Panagiotis Platis, Apostolos Ainalis, and Miltiadis Meliadis. 2010. 'Monitoring and Analysis of Natural Vegetation in a Special Protected Area of Mountain Antichasia—Meteora, Central Greece'. Environmental Monitoring and Assessment 163 (1–4): 455–65. https://doi.org/10.1007/s10661-009-0849-1.

Mikropoulou, Eleni V., Konstantina Vougogiannopoulou, Eleftherios Kalpoutzakis, Aimilia D. Sklirou, Zoi Skaperda, Joëlle Houriet, Jean-Luc Wolfender, et al. 2018. « Phytochemical Composition of the Decoctions of Greek Edible Greens (Chórta) and Evaluation of Antioxidant and Cytotoxic Properties ». Molecules 23 (7): 1541. https://doi.org/10.3390/molecules23071541.

Novaković, Jelica, Nemanja Rajčević, Sretco Milanovici, Petar D. Marin, and Pedja Janaćković. 2016. 'Essential Oil Composition of Centaurea Atropurpurea and Centaurea Orientalis Inflorescences from the Central Balkans – Ecological Significance and Taxonomic Implications'. Chemistry & Biodiversity 13 (9): 1221–29. https://doi.org/10.1002/cbdv.201600029.

Nowak, Gerard, Bohdan Drożdż, and Theodoros Georgiadis. 1984. 'Sesquiterpene Lactones. XXIX. Cnicin in Species of the Subgenus Acrolophus (Cass.) Dobrocz'. Acta Societatis Botanicorum Poloniae 53 (2): 2. https://doi.org/10.5586/asbp.1984.018.

Oreizi, Elaheh, Kazem Negaresh, and Mohammad Reza Rahiminejad. 2017. 'A New Name in Centaurea (Asteraceae, Cardueae) from Turkey'. Candollea 72 (2): 319–22. https://doi.org/10.15553/c2017v722a7.

Panagouleas, Constantinos, Helen Skaltsa, Diamanto Lazari, Alexios-Leandros Skaltsounis, et Marina Sokovic. 2003. « Antifungal Activity of Secondary Metabolites of Centaurea raphanina ssp. mixta, Growing Wild in Greece ». Pharmaceutical Biology 41 (4): 266-70. https://doi.org/10.1076/phbi.41.4.266.15664.

Panitsa, Maria, and Eleni Iliadou. 2011. 'Flora and Phytogeography of the Ionian Islands (Greece)'. Illes i Plantes: Conservacio i Coneixement de La Flora a Les Illes de La Mediterrania, April, 21. https://d1wqtxts1xzle7.cloudfront.net/39457673/Flora_and_Phytogeography_of_the_Ionian_I20151027-31421-11jlqgp-with-cover-page-v2.pdf?Expires=1660147409&Signature=VzX0tI6WvdEsHL7fl9Jan7hmhpm45jWTgnFMn2~I7-00egQPHPH6q9xDYBK4Bk36dxFKAYQiO7AHr7c7f8ftGcYHeZqfG13NRPpXmlVetK1aauoIVJb-V2aqanmbMrAcKBuq7oagjgu2fOUVstRpfPFMOvDy5~JwelK8fFOxC9YpSvfO43rqiXcjC6n8A6xNqoy~MPnX1418gs5JbZ73BTZOAMgmmqzs1gjd8OubDTIkV6W77y0Yn584BdGCqilMj9QDekOthA9UVth3Dqn6MqOpK-BPszXLAAyR~vDBJLCSbBlxsiR3rwXEyRcBFDE9fQWzJFWXehCssigQfEIJuA__&Key-Pair-Id=APKAJLOHF5GGSLRBV4ZA.

Panitsa, Maria, Eleni Iliadou, Ioannis Kokkoris, et al. 2020. 'Distribution Patterns of Ruderal Plant Diversity in Greece'. Biodiversity and Conservation 29 (3): 869–91. https://doi.org/10.1007/s10531-019-01915-4.

Panitsa, Maria, Nikolia Iliopoulou, and Emmanouil Petrakis. 2021. 'Citizen Science, Plant Species, and Communities' Diversity and Conservation on a Mediterranean Biosphere Reserve'. Sustainability 13 (17): 17. https://doi.org/10.3390/su13179925.

Pavlides, G. 1997. 'The Flora of Prespa National Park with Emphasis on Species of Conservation Interest'. In Lake Prespa, Northwestern Greece, edited by Alain J. Crivelli and George Catsadorakis. Springer Netherlands. https://doi.org/10.1007/978-94-011-5180-1_3.

Petropoulos, Spyridon A., Ângela Fernandes, Maria Ines Dias, Carla Pereira, Ricardo Calhelha, Francesco Di Gioia, Nikolaos Tzortzakis, et al. 2020. « Wild and Cultivated Centaurea Raphanina Subsp. Mixta: A Valuable Source of Bioactive Compounds ». Antioxidants 9 (4): 314. https://doi.org/10.3390/antiox9040314.

Petropoulos, Spyridon A., Ângela Fernandes, Maria Inês Dias, Carla Pereira, Ricardo C. Calhelha, Marija Ivanov, Marina D. Sokovic, Isabel C. F. R. Ferreira, et Lillian Barros. 2020. « The Effect of Nitrogen Fertigation and Harvesting Time on Plant Growth and Chemical Composition of Centaurea Raphanina Subsp. Mixta (DC.) Runemark ». Molecules 25 (14): 3175. https://doi.org/10.3390/molecules25143175.

Petropoulos, Spyridon, Ângela Fernandes, Maria Ines Dias, Carla Pereira, Ricardo C. Calhelha, Antonios Chrysargyris, Nikolaos Tzortzakis, et al. 2020. « Chemical Composition and Plant Growth of Centaurea Raphanina Subsp. Mixta Plants Cultivated under Saline Conditions ». Molecules 25 (9): 2204. https://doi.org/10.3390/molecules25092204.

Phitos, D. 1963. 'Eine Neue Art Der Gattung Centaurea Aus Der Ägäis - A New Species of the Genus Centaurea from the Aegean'. Annalen Des Naturhistorischen Museums in Wien 67: 165–67.

Phitos, Dimitrios, and Theophanis Constantinidis. 1993. 'A New Species of Centaurea Sect. Phalolepis from Greece'. Flora Mediterranea 3: 273–75.

Pop, Ioan. 1975. 'Studium über die Taxonomie, Chorologie und Zönologie der Art Centaurea atropurpurea W. et K. - Studies on the taxonomy, chorology and zoenology of the species Centaurea atropurpurea W. et K.' Phytocoenologia 2 (1–2): 49–53. https://doi.org/10.1127/phyto/2/1975/49.

Reeves, Roger D., A.J.M. Baker, and A. Kelepertsis. 1997. 'The Distribution and Biogeochemistry of Some Serpentine Plants of Greece'. In Ecologie Des Milieux Sur Roches Ultramafiques et Sur Sols Métallifères - The Ecology of Ultramafic and Metalliferous Areas, vol. 2. Documents Scientifiques et Techniques 3. ORSTOM - Centre de Nouméa. https://horizon.documentation.ird.fr/exl-doc/pleins_textes/pleins_textes_7/carton01/010011243.pdf#page=205.

Sarropoulou, Virginia, and Eleni Maloupa. 2019. 'Vegetative Propagation of Three Prioritized Greek Endemics with Potential Commercial Interest: Erysimum Naxense Snogerup, Erysimum Krendlii Polatschek and Centaurea Paxorum Phitos & Georgiadis'. GSC Biological and Pharmaceutical Sciences 6 (1): 1. https://doi.org/10.30574/gscbps.2019.6.1.0002.

Schuler, Andreas. 2007. 'Contribution to the Flora of Northern and Central Greece'. Willdenowia 37 (1): 229–41. https://doi.org/10.3372/wi.37.37113.

Shuka, Lulëzim, Sadik Malo, and Kit Tan. 2011. 'New Chorological Data and Floristic Notes for Albania'. Botanica Serbica 35 (2): 157–62.

Skaltsa, Helen, Diamante Lazari, Evanthia Georgiadou, Sotiris Kakavas, and Theophanis Constantinidis. 1999. 'Sesquiterpene Lactones from Centaurea Species: C. Thessala Subsp. Drakiensis and C. Attica Subsp. Attica'. Planta Medica 65 (4): 393–393. https://doi.org/10.1055/s-2006-960801.

Skaltsa, Helen, Diamanto Lazari, Begoña Garcia, José R. Pedro, Marina Sokovic, et Theophanis Constantinidis. 2000. « Sesquiterpene Lactones from Centaurea Achaia, a Greek Endemic Species: Antifungal Activity ». Zeitschrift Für Naturforschung C 55 (7-8): 534-39. https://doi.org/10.1515/znc-2000-7-810.

Skaltsa, Helen, Diamanto Lazari, Constantinos Panagouleas, Evanthia Georgiadou, Begoña Garcia, and Marina Sokovic. 2000. 'Sesquiterpene Lactones from Centaurea Thessala and Centaurea Attica. Antifungal Activity'. Phytochemistry 55 (8): 903–8. https://doi.org/10.1016/S0031-9422(00)00254-5.

Spanou, Sofia, Eirini Aplada, Argyro Tiniakou, and Theodoros Georgiadis. 2010. Contribution to the Study of the Flora of Attiki (Greece), New Records from the Flora of the Wider Athens International Airport Area. 17.

Spiliotis, Panagiotis. 2015. 'Are European Threatened Species Adequately Preserved in Ex Situ Institutions? Limitations of the IUCN Red List as a Guideline for Ex Situ Plant Conservation and a Proposal for an Integrated Strategy in Assessing the Conservation Importance and Value of a Species in Ex Situ Plant Collections.' Master of Science, University of Edinburgh.

Stamatis, George, Panayiotis Kyriazopoulos, Stamatina Golegou, Aris Basayiannis, Spyros Skaltsas, and Helen Skaltsa. 2003. 'In Vitro Anti-Helicobacter Pylori Activity of Greek Herbal Medicines'. Journal of Ethnopharmacology 88 (2): 175–79. https://doi.org/10.1016/S0378-8741(03)00217-4.

Stephanaki, Dimitri. 2009. 'Βοτανικοι πηποι στην Ευρωπη: το παραδειγμα της Ροδου - Botanical gardens in Europe: the example of Rhodes'. Master of Science, T.E.I. Kritis.

Stevanovic, V., Kit Tan, and G. Iatrou. 2003. 'Distribution of the Endemic Balkan Flora on Serpentine I. - Obligate Serpentine Endemics'. Plant Systematics and Evolution 242 (1–4): 149–70. https://doi.org/10.1007/s00606-003-0044-8.

Strid, Arne, and Kit Tan. 2009. 'A New Species of Centaurea (Asteraceae) from the Island of Samothraki (NE Greece)'. Phytologia Balcanica 15 (2): 185–89.

Strid, Arne. 2015. 'Reliquiae Runemarkianae. Chromosome Numbers of Angiosperms from the Aegean Islands'. Phytologia Balcanica 21 (3): 245–93.

Strid, Arne. 2020. 'The Botanical Exploration of Greece'. Plant Systematics and Evolution 306 (2): 27. https://doi.org/10.1007/s00606-020-01637-z.

Tan, Kit, and Giannis Kofinas. 2022. 'Notes on Centaurea Vandasii and C. Wettsteinii (Asteraceae) , Two Rare and Little-Known Species in the Balkan Peninsula'. Phytologia Balcanica 28 (1): 61–67. https://doi.org/10.7546/PhB.28.2022.5.

Tan, Kit, Andreas Schuler, and Arne Strid. 2007. 'A Pink-Flowered Variant of Centaurea Prespana (Asteraceae) in NW Greece'. Phytologia Balcanica 13 (3): 351–52.

Thanos, Costas A. 2014. 'In Situ and Ex Situ Plant Conservation in Greece in the Framework of the Global Strategy for Plant Conservation'. Bullettin of the Gioenia Academy of Natural Sciences of Catania 47 (377/SFE): 377/SFE.

Thanos, Costas A. 2014. « In Situ and Ex Situ Plant Conservation in Greece in the Framework of the Global Strategy for Plant Conservation ». Bullettin of the Gioenia Academy of Natural Sciences of Catania 47 (377/SFE): SFE25-30.

Trigas, P., Th. Constantinidis, and T. Touloumenidou. 2008. 'A New Hexaploid Species of Centaurea Section Acrolophus (Asteraceae) from Evvia Island, Greece'. Botanical Journal of the Linnean Society 158 (4): 762–74. https://doi.org/10.1111/j.1095-8339.2008.00870.x.

Trigas, Panayiotis, and Gregoris Iatrou. 2006. 'The Local Endemic Flora of Evvia (W Aegean, Greece)'. Willdenowia 36 (1): 257–70. https://doi.org/10.3372/wi.36.36121.

Tsakiri, Maria, Konstantinos Kougioumoutzis, and Gregoris Iatrou. 2016. 'Contribution to the Vascular Flora of Chalki Island (East Aegean, Greece) and Biomonitoring of a Local Endemic Taxon'. Willdenowia 46 (1): 175–90. https://doi.org/10.3372/wi.46.46114.

Turland, Nicholas J., and Lance Chilton. 2000. 'A Revision of Centaurea Argentea (Compositae, Cardueae), an Endemic Species of Kriti and Kithira (Greece)'. Botanika Chronika 13: 71–79.

Turland, Nicholas, Dimitrios Phitos, Georgia Kamari, and Pepy Bareka. 2004. 'Weeds of the Traditional Agriculture of Crete'. Willdenowia 34 (2): 381–406. https://doi.org/10.3372/wi.34.34206.

Turland, Nicholas. 1994. 'Another Daphne from Crete'. Curtis's Botanical Magazine 11 (3): 117–28. https://doi.org/10.1111/j.1467-8748.1994.tb00424.x.

Uysal, Tuna, Kuddisi Ertugrul, Alfonso Susanna, and Núria Garcia-Jacas. 2009. 'New Chromosome Counts in the Genus Centaurea (Asteraceae) from Turkey'. Botanical Journal of the Linnean Society 159 (2): 280–86. https://doi.org/10.1111/j.1095-8339.2008.00939.x.

Vladimirov, Vladimir, Mehmet Aybeke, and Kit Tan. 2022. 'New Floristic Records in the Balkans: 48'. Phytologia Balcanica 28 (2): 249–79. https://doi.org/10.7546/PhB.28.2.2022.9.

Vonica, Ghizela, and Maria Cantor. 2012. 'Centaurea Species Attractive Transylvanian (Romania) Endemic and Rare Plants with High Potential as Flowering Garden Plant'. Bulletin UASVM Horticulture 69 (1): 354–62.

Wagenitz, G, and E Gamal-Eldin. 1985. 'Zur Kenntnis der griechischen Centaurea-Arten der Sektion Acrocentron - About the Greek Centaurea species of the Acrocentron section'. Bot. Jahrb. Syst. 107 (14): 95–127.

Wagenitz, Gerhard, ERWIN Bergmeier, THOMAS Gregor, LENZ Meierott, Lulezim Shuka, and Kit Tan. 2018. 'A Synopsis of the Centaurea Soskae and Triniifolia Group (Centaurea Sect.

Acrolophus) in the Prespa Area and Northern Pindos.' Phytotaxa 348 (2): 77–89.

Wagenitz, Gerhard. 1971. 'Centaurea Pseudocadmea, Eine Neue Art Der Sektion Phalolepis Aus Griechenland'. Annalen Des Naturhistorischen Museums in Wien 75: 243–47.

Wagenitz, Gerhard. 1971. « Centaurea pseudocadmea, eine neue Art der Sektion Phalolepis aus Griechenland ». Annalen des Naturhistorischen Museums in Wien 75: 243-47.

Zeghichi, Sabrina, Stamatina Kallithraka, Artemis P. Simopoulos, and Zacharias Kypriotakis. 2003. 'Nutritional Composition of Selected Wild Plants in the Diet of Crete'. In Plants in Human Health and Nutrition Policy, edited by Artemis P. Simopoulos and C. Gopalan, vol. 91. Karger Medical and Scientific Publishers.

Zervaki, D., K. Papanastasi, and E. Maloupa. 2009. 'A NEW THEORY – MODEL STRATEGY FOR NEW FLOWER CROPS DEVELOPMENT'. Acta Horticulturae, no. 813 (March): 147–54. https://doi.org/10.17660/ActaHortic.2009.813.19.

Zervaki, D., K. Papanastasi, et E. Maloupa. 2009. « A NEW THEORY – MODEL STRATEGY FOR NEW FLOWER CROPS DEVELOPMENT ». Acta Horticulturae, no 813 (mars): 147-54. https://doi.org/10.17660/ActaHortic.2009.813.19.

Zografidis, Aris, Svetlana Bancheva, and Kit Tan. 2014. 'A New Yellow-Flowered Species of Centaurea (Asteraceae) from Mt Imittos, Attica, Greece'. Phytologia Balcanica 20 (1): 57–63.

Βηλαρά, Βασιλική. 2011. 'Υδρογεωλογική και περιβαλλοντική μελέτη της λεκάνης των Τρικάλων - Hydrogeological and environmental study of the surroundings of Trikala'. Thesis, Patras University. http://nemertes.library.upatras.gr/jspui//handle/10889/5302.

Παππας, Ιωαννις Οδυσσεας. 2021. 'Συγκριση υπογειας και υπαιθριας χαραξης για την οδικη συνδεση Μελισσουργοι-Θεοδωριανα - Comparison of underground and outdoor layout for the road connection Melissourgoi - Theodoriana'. Bachelor Thesis, ΕΘΝΙΚΟ ΜΕΤΣΟΒΙΟ ΠΟΛΥΤΕΧΝΕΙΟ. https://dspace.lib.ntua.gr/xmlui/bitstream/handle/123456789/53526/Pappas_diplomatiki.pdf?sequence=1.

Τζανελλη, Γεωργια. 2006. 'Μελέτη Της Χλωρίδας Ενός Βιολογικού, Ενός Συμβατικού Και Ενός Εγκαταλελειμμένου Ελαιώνα - Study of the Flora of an Organic, a Conventional and an Abandoned Olive Grove'. A.T.E.I. Kritis. https://apothesis.lib.hmu.gr/bitstream/handle/20.500.12688/821/2006Tzanelli.pdf?sequence=1&isAllowed=y.

Τσακίρη, Μαρία. 2014. 'Νήσος Χάλκη : καταγραφή της χλωριδικής ποικιλότητας, βιοπαρακολούθηση των σπάνιων και ενδημικών φυτών της και η συμβολή τους στην οικοτουριστική ανάπτυξη της περιοχής - Chalki : recording of floristic diversity, biomonitoring of rare and endemic plants, and its contribution to the ecotourism development of the island'. Thesis, University of Patras. http://nemertes.library.upatras.gr/jspui//handle/10889/9655.

Cirsium

Larnaudie, Stephanie, and Pierre Labrude. 1999. 'Marcel Petitmengin (1881-1908), pharmacien botaniste : sa vie, son œuvre - Marcel Petitmengin (1881-1908), pharmacist and botanist: his life, his work'. *Revue d'Histoire de la Pharmacie* 87 (323): 335–42. https://doi.org/10.3406/pharm.1999.4972.

Strid, Arne. 2020. 'The Botanical Exploration of Greece'. *Plant Systematics and Evolution* 306 (2): 27. https://doi.org/10.1007/s00606-020-01637-z.

Strid, Arne, and Kit Tan. 2017. 'Recent Progress In Plant Taxonomy And Floristic Studies In Greece'. *Botanica Serbica* 41 (2): 123–52. https://doi.org/10.5281/ZENODO.1026649.

Vogiatzakis, I. N., G. H. Griffiths, and A. M. Mannion. 2003. 'Environmental Factors and Vegetation Composition, Lefka Ori Massif, Crete, S. Aegean'. *Global Ecology and Biogeography* 12 (2): 131–46. https://doi.org/10.1046/j.1466-822X.2003.00021.x.

Vogiatzakis, Ioannis N., and Geoffrey H. Griffiths. 2001. 'Vegetation-Environment Relationships in Lefka Ori (Crete, Greece) : Ordination Results from Montane-Mediterranean and Oro-Mediterranean Communities'. *Ecologia Mediterranea* 27 (1): 1–13. https://doi.org/10.3406/ecmed.2001.1903.

Chondrilla

Iatrou, Gregoris, Panayiotis Trigas, et Nicolaos Pettas. 2007. « The Vascular Flora of Akrokorinthos Castle and Its Surrounding Area (NE Peloponnese, Greece) », 12.

Sytwala, Sonja, André Domsalla, et Matthias F. Melzig. 2015. « Investigation of Plant Latices of Asteraceae and Campanulaceae Regarding Proteolytic Activity ». *Plant Physiology and Biochemistry* 97 (décembre): 117-23. https://doi.org/10.1016/j.plaphy.2015.09.017.

Crepis

Anagnostopoulos, Anastasios. 1997. Karyotype Variation in Crepis Fraasii and C. Reuteriana (Asteraceae) in Greece. 6.

Authier, Pierre. 1997. 'La flore de la région des monts Timfi (Epire, nord-ouest de la Grèce): un premier bilan - The flora of the region of Mounts Timfi (Epirus, North-West Greece)'. Bocconea 5: 477–85.

Babcock, E. B., et Lillian Hollingshead. 1930. Chromosomes and Phylogeny in Crepis,. Berkeley, Calif.,: University of California press,. https://doi.org/10.5962/bhl.title.61469.

Barda, Christina, Ana Ciric, Marina Soković, Michael Tsoukalas, et Helen Skaltsa. 2018. « Phytochemical Investigation of Crepis Incana Sm. (Asteraceae) Endemic to Southern Greece ». Biochemical Systematics and Ecology 80 (octobre): 59-62. https://doi.org/10.1016/j.bse.2018.06.009.

Bergmeier, Erwin, Thomas Blockeel, Niels Böhling, et al. 2011. 'An Inventory of the Vascular Plants and Bryophytes of Gavdopoula Island (S Aegean, Greece) and Its Phytogeographical Significance'. Willdenowia 41 (1): 179–90. https://doi.org/10.3372/wi.41.41121.

Böhling, Niels, Werner Greuter, and Thomas Raus. 1998. 'Trifolium Phitosianum (Leguminosae), a New Annual Clover Species from Crete'. Bot. Chron. 13.

Constantinidis, Theophanidis, Eleftheria-Perdiko Bareka, and Georgia Kamari. 2002. 'Karyotaxonomy of Greek Serpentine Angiosperms'. Botanical Journal of the Linnean Society 139 (1): 109–24. https://doi.org/10.1046/j.1095-8339.2002.00044.x.

Constantinidis, Theophanis, Alex Korakis, and George Fakas. 2014. ‘Not a Cretan Area Endemic Any Longer: The Discovery of Crepis Tybakiensis (Asteraceae: Cichorieae) in Samos (East Aegean Islands, Greece)’. Parnassiana Archives 2: 65–70.

Georgiadis, Lazaros. 2004. Voras-Kaimakchalan (Greece) Presentation of the Natural Characteristics and the Protection Status. http://catsg.org/balkanlynx/04_land-use/4_3_protected-area/protected-areas/Pdfs/Georgiadis_2001_Voras_Greece.pdf.

Kamari, G. 1992. ‘Karyosystematic Studies on Three Crepis Species (Asteraceae) Endemic to Greece’. Plant Systematics and Evolution 182 (1): 1–19. https://doi.org/10.1007/BF00941411.

Kamari, G. 1992. « Karyosystematic Studies on Three Crepis Species (Asteraceae) Endemic to Greece ». Plant Systematics and Evolution 182 (1): 1-19. https://doi.org/10.1007/BF00941411.

Kamari, Georgia, and Arne Strid. 1989. ‘Crepis Arcuata (Asteraceae), a New Species from N Greece’. Willdenowia 19 (1): 79–82.

Kamari, Georgia, and Per Hartvig. 1988. ‘Crepis Merxmuelleri (Asteraceae), a New Species from NW Greece’. Willdenowia 18: 63–66.

Kamari, Georgia, and Ursula Matthas. 1986. ‘Cytotaxonomical Contributions on the Flora of Crete. III’. Willdenowia 15 (2): 515–20.

Karetsos, George, Alexandra D. Solomou, Panayiotis Trigas, and Konstantinia Tsagari. 2018. ‘The Vascular Flora of Mt. Oiti National Park and the Surrounding Area in Greece’. Journal of Forest Science 64 (No. 10): 435–54. https://doi.org/10.17221/65/2018-JFS.

Karydas, Antony, and Georgia Kamari. 2019. ‘Monitoring Six Local Endemic Taxa of the Mt Athos and Assessment According to the IUCN Red List Categories and Criteria’. Botanika Chronika 22: 195–208.

Kazakis, George, Dany Ghosn, Ilektra Remoundou, Panagiotis Nyktas, Michael A. Talias, and Ioannis N. Vogiatzakis. 2021. ‘Altitudinal Vascular Plant Richness and Climate Change in the Alpine Zone of the Lefka Ori, Crete’. Diversity 13 (1): 1. https://doi.org/10.3390/d13010022.

Kougioumoutzis, Konstantinos, Ioannis P. Kokkoris, Maria Panitsa, Arne Strid, and Panayotis Dimopoulos. 2021. ‘Extinction Risk Assessment of the Greek Endemic Flora’. Biology 10 (3): 3. https://doi.org/10.3390/biology10030195.

Krigas, Nikos, Georgios Tsoktouridis, Ioannis Anestis, et al. 2021. ‘Exploring the Potential of Neglected Local Endemic Plants of Three Mediterranean Regions in the Ornamental Sector: Value Chain Feasibility and Readiness Timescale for Their Sustainable Exploitation’. *Sustainability* 13 (5): 5. https://doi.org/10.3390/su13052539.

Krigas, Nikos, Viktoria Menteli, et Despoina Vokou. 2014. « The Electronic Trade in Greek Endemic Plants: Biodiversity, Commercial and Legal Aspects ». Economic Botany 68 (1): 85-95. https://doi.org/10.1007/s12231-014-9264-9.

Löve, Askell. 1983. ‘IOPB Chromosome Number Reports LXXVIII’. Taxon 32 (1): 138–41.

Mahmutaj, Ermelinda, Petrit Hoda, and Lulëzim Shuka. 2014. ‘Rare Plants and Their Conservation Status in Tomorri National Park’. Journal of Endocytobiosis and Cell Research 25: 27–32.

Stevanovic, V., Kit Tan, and G. Iatrou. 2003. ‘Distribution of the Endemic Balkan Flora on Serpentine I. - Obligate Serpentine Endemics’. Plant Systematics and Evolution 242 (1–4): 149–70. https://doi.org/10.1007/s00606-003-0044-8.

Vogiatzakis, I. N., G. H. Griffiths, and A. M. Mannion. 2003. ‘Environmental Factors and Vegetation Composition, Lefka Ori Massif, Crete, S. Aegean’. Global Ecology and Biogeography 12 (2): 131–46. https://doi.org/10.1046/j.1466-822X.2003.00021.x.

Echinops

Özdeniz, Ebru. 2017. « Syntaxonomical Synopsis of the Steppe Vegetation of Turkey ». *Ankara Üniversitesi Çevrebilimleri Dergisi* 5 (2): 29-49. https://doi.org/10.1501/Csaum_0000000079.

Papadopoulou, Panagiota, Maria Couladis, et Olga Tzakou. 2006. « Essential Oil Composition of two Greek Echinops species: E. graecus Miller and E. ritro L. » *Journal of Essential Oil Research* 18 (3): 242-43. https://doi.org/10.1080/10412905.2006.9699076.

Sánchez-Jiménez, Ismael, Oriane Hidalgo, Miguel Ángel Canela, Sonja Siljak-Yakovlev, Marija Edita Šolić, Joan Vallès, et Teresa Garnatje. 2012. « Genome Size and Chromosome Number in Echinops (Asteraceae, Cardueae) in the Aegean and Balkan Regions: Technical Aspects of Nuclear DNA Amount Assessment and Genome Evolution in a Phylogenetic Frame ». *Plant Systematics and Evolution* 298 (6): 1085-99. https://doi.org/10.1007/s00606-012-0618-4.

Filago

Bergmeier, Erwin. 2010. ‘Filago Wagenitziana (Asteraceae, Gnaphalieae), a New Species from Western Crete, Greece’. *Willdenowia* 40 (2): 183–88.

Kougioumoutzis, Konstantinos, Argyro Tiniakou, Ourania Georgiou, and Theodoros Georgiadis. 2012. ‘Contribution to the Flora of the South Aegean Volcanic Arc: Anafi Island (Kiklades, Greece)’. *Willdenowia* 42 (1): 127–41. https://doi.org/10.3372/wi.42.42115.

Wagenitz, Gerhard, and H. Runemark. 1970. ‘Die Gattung Filago L. s. l. (Compositae-Inuleae) in Der Ägäis - The Genus Filago L. s. l. (Compositae-Inuleae) in the Aegaean Area’. *Willdenowia* 6 (1): 115–38.

Χατζησεβαστός, Βησσαρίων. 2019. ‘Η αυτοφυής χλωρίδα της νήσου Κω και η χρήση της από τους κατοίκους. - The native flora of Kos and species used by the people of Kos’. Bachelor Thesis, Technological Educational Institute of Crete, Department of Agriculture. https://apothesis.lib.hmu.gr/handle/20.500.12688/9254.

Helichrysum

Chinou, Ioanna B., Christos Bougatsos, and Dimitrios Perdetzoglou. 2004. ‘Chemical Composition and Antimicrobial Activities of Helichrysum Amorginum Cultivated in Greece’. *Journal of Essential Oil Research* 16 (3): 243–45. https://doi.org/10.1080/10412905.2004.9698711.

Chinou, Ioanna B., Vassilios Roussis, Demitrios Perdetzoglou, Olga Tzakou, and Anargiros Loukis. 1997. 'Chemical and Antibacterial Studies of Two Helichrysum Species of Greek Origin'. *Planta Medica* 63 (2): 181–83. https://doi.org/10.1055/s-2006-957641.

Goulimis, C. 1958. *Report on Species of Plants Requiring Protection in Greece and Measures for Securing Their Protection*. Athens. http://documents.irevues.inist.fr/bitstream/handle/2042/59422/LATERREETLAVIE_1959_Sup_168.pdf?sequence=1.

Karydas, Antony, and Georgia Kamari. 2019. 'Monitoring Six Local Endemic Taxa of the Mt Athos and Assessment According to the IUCN Red List Categories and Criteria'. *Botanika Chronika* 22: 195–208.

Libiad, Mohamed, Abdelmajid Khabbach, Mohamed El Haissoufi, et al. 2021. 'Agro-Alimentary Potential of the Neglected and Underutilized Local Endemic Plants of Crete (Greece), Rif-Mediterranean Coast of Morocco and Tunisia: Perspectives and Challenges'. *Plants* 10 (9): 9. https://doi.org/10.3390/plants10091770.

Löve, Áskell. 1986. « Chromosome Number Reports XCIII ». *Taxon* 35 (4): 897-903.

Maloupa, E, N Krigas, and A Karydas. 2007. *The in Situ Plant Conservation Actions of the Balkan Botanic Garden of Kroussia in Greece*. 5.

Maloupa, Eleni, Nikos Krigas, Katerina Grigoriadou, Diamanto Lazari, and Georgios Tsoktouridis. 2008. *Conservation Strategies for Native Plant Species and Their Sustainable Exploitation: Case of the Balkan Botanic Garden of Kroussia, N. Greece*. 21.

Roussis, Vassilios, Maria Tsoukatou, Panos V. Petrakis, Ioanna Chinou, Melpomeni Skoula, and Jeffrey B. Harborne. 2000. 'Volatile Constituents of Four Helichrysum Species Growing in Greece'. *Biochemical Systematics and Ecology* 28 (2): 163–75. https://doi.org/10.1016/S0305-1978(99)00046-0.

Strid, Arne. 2020. 'The Botanical Exploration of Greece'. *Plant Systematics and Evolution* 306 (2): 27. https://doi.org/10.1007/s00606-020-01637-z.

Tan, Kit. 2007. 'The Endemic Flora of Greece'. *Building a Sustainable Future: The Role of Botanic Gardens.*, April 16, 5.

Ανέστης, Ιωάννης Ανδρέα. 2020. 'Πολύπλευρη αξιολόγηση, οικολογικά προφίλ και δράσεις εκτός τόπου διατήρησης τοπικών ενδημικών φυτών της Κρήτης - Multifaceted assessment, ecological profiles and off-site conservation actions of local endemic plants of Crete'. Master of Science, Αριστοτέλειο Πανεπιστήμιο Θεσσαλονίκης.

Αργυρίου, Σοφία. 2020. *Επίδραση θερμοκρασίας και φωτός στη φυτρωτική ικανότητα σπόρων Helichrysum amorginum Boiss & Orph.* June 16. http://dspace.aua.gr/xmlui/handle/10329/7077.

Hieracium

Authier, Pierre. 1997. 'La flore de la région des monts Timfi (Epire, nord-ouest de la Grèce): un premier bilan - The flora of the region of Mounts Timfi (Epirus, North-West Greece)'. *Bocconea* 5: 477–85.

Bergmeier, Erwin, Fanourios-Nikolaos Sakellarakis, Arne Strid, and Constant Swinkels. 2020. *New Additions to the Flora of Prespa, Greece*. 28.

Gottschlich, G. 2023. 'Four New Hieracium Taxa (Compositae) from the Balkans (North Macedonia, Montenegro) and Greece and One New Hieracium Record for Europe'. *Annalen Des Naturhistorischen Museums in Wien. Serie B Für Botanik Und Zoologie* 125: 143–54.

Gottschlich, G., G. Domina, and E. Di Gristina. 2017. 'Hieracium Umbrosum Subsp. Abietinum (Asteraceae), a Further Example of Amphi-Adriatic Disjunction'. *Plant Biosystems - An International Journal Dealing with All Aspects of Plant Biology* 151 (5): 792–94. https://doi.org/10.1080/11263504.2017.1341439.

Gottschlich, Günter, Detlev Drenckhahn, et Lenz Meierott. 2006. « Hieracium greuteri (Compositae), a Local Endemic of N Peloponnisos (Greece) ». *Willdenowia* 36 (1): 351-56.

Gottschlich, Günter, and Franz G Dunkel. 2019. 'New Taxa of Hieracium and Pilosella (Asteraceae) from Northern Greece II.' *STAPFIA* 111: 5–32.

Gottschlich, Günter, Konstantina Melikoki, Eleni Eleftheriadou, and Konstantinos Theodoropoulos. 2013. 'Three New Hieracium Taxa (Asteraceae, Cichorieae) from Mt Cholomon, Chalkidiki, Central Makedonia, Greece'. *Willdenowia* 43 (1): 59–64.

Kanellopoulos, Nikolaos. 2018. 'Περιβαλλοντικη Αποκατασταση Ανενεργου Λατομειου στη θεση τζουμα του δημου κεντρικων τζοθμερκων - Environmental restoration of an inactive quarry at the Juma site of the Municipality of Central Jothmerka'. Bachelor Thesis, Ethniko Metsobio Polytechneio. https://dspace.lib.ntua.gr/xmlui/bitstream/handle/123456789/48466/%CE%94%CE%99%CE%A0%CE%9B%CE%A9%CE%9C%CE%91%CE%A4%CE%99%CE%9A%CE%97.pdf?sequence=1.

Musiał, Krystyna, Vladimir Vladmirov, and Zbigniew Szeląg. 2020. 'Chromosome Numbers in Hieracium (Asteraceae) from Central and Southeastern Europe VI'. *Acta Biologica Cracoviensia s. Botanica* 62 (2): 43–50. https://doi.org/10.24425/ABCSB.2020.131672.

Szelag, Z., and V. Vladimirov. 2019. 'The Species Intermediate between Hieracium Petrovae and H. Olympicum (Asteraceae): A Treatment of H. Kritschimanum and Description of a New Species from Greece.' *Phytotaxa* 402 (2): 107–13.

Tashev, Alexander, and Evgenia Pancheva. 2011. 'The Melliferous Plants of the Bulgarian Flora - Conservation Importance'. *Forestry Ideas* 17 (2): 10.

Vrakota, Basiliki. 2017. 'Η τοπικη ιστορια ως εργαλειο αναδειξης πολιτισμικης κληρονομιας. Το παραδειγμα των Ζαγοροχωριων της περιφερειας Ιωαννινων. Το Γρεβενιτι. - The local history as a tool of cultural heritage. The example of Zagorochoria, Ioannina. Greveniti.' Bachelor Thesis, Panepistimio Diktikis Makedonias. http://dspace.uowm.gr/xmlui/bitstream/handle/123456789/579/Vrakota%20Vasiliki.pdf?sequence=1&isAllowed=y.

Hymenonema

Liveri, Eleni, Salvatore Tomasello, Christian Hammerschmid, Georgia Kamari, et Christoph Oberprieler. 2018. « Differentiation of the Endemic Greek Genus Hymenonema and Its Relatives of Subtribe Scolyminae (Compositae, Cichorieae) Based on a Multilocus Species Tree Reconstruction ». *Plant Systematics and Evolution* 304 (10): 1255-67. https://doi.org/10.1007/s00606-018-1545-9.

Liveri, Eleni, Salvatore Tomasello, Christoph Oberprieler, et Georgia Kamari. 2016. « Cytological and Phylogenetic Study of the Greek Endemic Genus Hymenonema Cass. (Cichorieae, Compositae) ». https://doi.org/10.13140/RG.2.2.25437.82401.

Petropoulos, Spyridon A., Ângela Fernandes, Nikolaos Tzortzakis, et al. 2019. 'Bioactive Compounds Content and Antimicrobial Activities of Wild Edible Asteraceae Species of the Mediterranean Flora under Commercial Cultivation Conditions'. *Food Research International* 119 (May): 859–68. https://doi.org/10.1016/j.foodres.2018.10.069.

Salonikioti, A., S. Petropoulos, V. Antoniadis, E. Levizou, and A. Alexopoulos. 2015. 'Wild Edible Species with Phytoremediation Properties'. *Procedia Environmental Sciences* 29: 98–99. https://doi.org/10.1016/j.proenv.2015.07.180.

Hypochaeris

Bacchetta, Gianluigi, Salvatore Brullo, and Maria Carmen Terrasi. 2003. 'A New Species of Hypochaeris L. (Asteraceae, Cichorieae) from Sardinia'. *Willdenowia* 33 (1): 71–78. https://doi.org/10.3372/wi.33.33107.

Inula

Ahmed, Ahmed A., Abou El-Hamd H. Mohamed, Olga Tzakou, Alexandra Petropoulou, Mohamed E. Hassan, Mohamed A. El-Maghraby, et Klaus-Peter Zeller. 2003. « Terpenes from Inula Verbascifolia ». *Phytochemistry*, Reports on Structure Elucidation, 62 (8): 1191-94. https://doi.org/10.1016/S0031-9422(02)00478-8.

De Montmollin, B. 1984. « Etude cytotaxonomique de la flore de la Crète. II: Nombres chromosomiques ». *Etude cytotaxonomique de la flore de la Crète. II: Nombres chromosomiques* 94 (2): 261-67.

Harvala, Evagelia, Nektarios Aligiannis, Alexios-Leandros Skaltsounis, Haris Pratsinis, George Lambrinidis, Catherine Harvala, et Ioanna Chinou. 2002. « Cytotoxic Germacranolides from Inula verbascifolia subsp. methanea ». *Journal of Natural Products* 65 (7): 1045-48. https://doi.org/10.1021/np010569t.

Iatrou, Gregoris, Panayiotis Trigas, et Nicolaos Pettas. 2007. « The Vascular Flora of Akrokorinthos Castle and Its Surrounding Area (NE Peloponnese, Greece) », 12.

Krigas, Nikos, Georgios Tsoktouridis, Ioannis Anestis, et al. 2021. 'Exploring the Potential of Neglected Local Endemic Plants of Three Mediterranean Regions in the Ornamental Sector: Value Chain Feasibility and Readiness Timescale for Their Sustainable Exploitation'. *Sustainability* 13 (5): 5. https://doi.org/10.3390/su13052539.

Maleš, Željan, Kroata Hazler Pilepić, Lina Petrović, et Iva Bagarić. 2010. « Quantitative Analysis of Phenolic Compounds of Inula Candida (L.) Cass. » *Periodicum Biologorum* 112 (3): 307-10.

Michalakea, Eleftheria, Konstantia Graikou, Nektarios Aligiannis, George Panoutsopoulos, Eleftherios Kalpoutzakis, Christos Roussakis, et Ioanna Chinou. 2019. « Isolation and Structure Elucidation of Secondary Metabolites of Two Greek Endemic Inula Species. Biological Activities ». *Phytochemistry Letters* 31 (juin): 155-60. https://doi.org/10.1016/j.phytol.2019.04.004.

Trigas, Panayiotis, and Gregoris Iatrou. 2006. 'The Local Endemic Flora of Evvia (W Aegean, Greece)'. *Willdenowia* 36 (1): 257–70. https://doi.org/10.3372/wi.36.36121.

Tzakou, O., A. Petropoulou, C. Harvala, et Th. Constantinidis. 2001. « Volatile Compounds of Two Members of Inula Verbascifolia Group: I. Verbascifolia (Willd.) Hausskn. Ssp. Parnassica (Boiss. Et Heldr.) Tutin and I. Verbascifolia Ssp. Methanea (Hausskn.) Tutin ». *Journal of Essential Oil Research* 13 (5): 364-66. https://doi.org/10.1080/10412905.2001.9712235.

Jacobaea

Cattaneo, Cristina, and Mauro Grano. 2021. Kasos: An Unexpected Island. Floristic and Ecological Analysis of Kasos Island (SE Aegean, Dodecanese, Greece), with Noteworthy Floristic Additions. 28.

Doumas, Panayiotis, Katerina Goula, and Theophanis Constantinidis. 2022. 'Thirty-Two New and Noteworthy Floristic Records from North-Eastern Greece'. Biodiversity Data Journal 10 (April): e81817. https://doi.org/10.3897/BDJ.10.e81817.

Sutorỳ, Karel. 2021. 'Taxonomic and Nomenclatural Notes on Jacobaea Othonnae (Asteraceae-Senecioneae-Senecioninae).' Phytotaxa 510 (1): 61–68.

Lactuca

Maxted, Nigel, and Shelagh Kell. 2012. 'Greece: Cradle of European Plant Genetic Diversity and Hotspot for Conservation Action'. Lesson in Agrobiodiversity. University of Birmingham, UK.

Wani, Mohammad Saleem, Younas Rasheed Tantray, Vijay Kumar Singhal, and R. C. Gupta. 2020. 'Chapter 1: Lactuca L.: World Distribution and Importance'. In *Lactuca: Cultivation and Uses*, edited by Jan Krüger. Plant Science Research and Practices. Nova Science Publishers.

Lamyropsis

Greuter, Werner. 1979. 'Quisquiliae Floristicae Graecae, 4. Lamyropsis Carpini (Compositae), a New Species from NW.Greece'. *Willdenowia* 9 (1): 57–66.

Kamari, Georgia, Pepy Bareka, Theophanis Constantinidis, and Dimitrios Phitos. 2003. 'Karyosystematic Studies of Plant Taxa from the East Mediterranean Region (Grece, Cyprus, Syria)'. *Phytologia Balcanica* 9 (3): 487–502.

Leontodon

Iatrou, Gregoris, Panayiotis Trigas, et Nicolaos Pettas. 2007. « The Vascular Flora of Akrokorinthos Castle and Its Surrounding Area (NE Peloponnese, Greece) », 12.

Krigas, Nikos, Marina Panagiotidou, et Eleni Maloupa. 2017. « Incorporating biogeographical principles in horticulture: design and creation of the ionian islands unique rock garden in Thessaloniki, Greece. » *Sibbaldia: The Journal of Botanic Garden Horticulture*, 2017, 15 édition.

Onopordum

Aytaç, Zeki, and Hayri Duman. 2013. 'A New Species and 2 New Records from Turkey'. *Turkish Journal of Botany* 37 (6): 1055–60. https://doi.org/10.3906/bot-1209-24.

Phitosia

Enke, Neela, et Birgit Gemeinholzer. 2008. « Babcock Revisited: New Insights into Generic Delimitation and Character Evolution in Crepis L. (Compositae: Cichorieae) from ITS and MatK Sequence Data ». *TAXON* 57 (3): 756-68. https://doi.org/10.1002/tax.573008.

Kamari, G, C Kyriakopoulos, et G Kofinas. 2010. « New Finding of Phitosia Crocifolia (Compositae) in E Peloponnisos », 4.

Vargas, Pablo, Pedro Jiménez-Mejías, et Mario Fernández-Mazuecos. 2020. « 'Endangered Living Fossils' (ELFs): Long-Term Survivors through Periods of Dramatic Climate Change ». *Environmental and Experimental Botany*, The climatic challenge: learning from past survivors and present outliers, 170 (février): 103892. https://doi.org/10.1016/j.envexpbot.2019.103892.

Zhang, Jian-Wen, Ze-Long Nie, Jun Wen, et Hang Sun. 2011. « Molecular Phylogeny and Biogeography of Three Closely Related Genera, Soroseris, Stebbinsia, and Syncalathium (Asteraceae, Cichorieae), Endemic to the Tibetan Plateau, SW China ». *TAXON* 60 (1): 15-26. https://doi.org/10.1002/tax.601003.

Pilosella

Gottschlich, Günter, and Franz G Dunkel. 2019. 'New Taxa of Hieracium and Pilosella (Asteraceae) from Northern Greece II.' *STAPFIA* 111: 5–32.

Vladimirov, Vladimir, Kamil Çoşkunçelebi, and Kit Tan. 2015. 'A New Diploid Species of Pilosella (Asteraceae) from Turkey'. *TURKISH JOURNAL OF BOTANY* 39: 70–75. https://doi.org/10.3906/bot-1401-92.

Rhaponticoides

Çinbilgel, İlker, Özkan Eren, and Hayri Duman. 2014. 'Rhaponticoides Gokceoglui (Asteraceae), a Striking New Species from Turkey'. PHYTOTAXA 170 (2). https://doi.org/10.11646/phytotaxa.170.2.5.

Scorzonera

Constantinidis, Theophanis, Georgia Kamari, et Dimitrios Phitos. 1997. « A Cytological Study of 28 Phanerogams from the Mountains of SE Sterea Ellas, Greece ». *Willdenowia* 27 (1/2): 121-42.

Gustafsson, M., and Sven Snogerup. 1972. 'Scorzonera Scyria, a New Chasmophytic Species from Greece'. *Bot. Not.* 125 (4): 323–28.

Paraschos, Sotiris, Prokopios Magiatis, Eleftherios Kalpoutzakis, Catherine Harvala, et Alexios-Leandros Skaltsounis. 2001. « Three New Dihydroisocoumarins from the Greek Endemic Species Scorzonera cretica ». *Journal of Natural Products* 64 (12): 1585-87. https://doi.org/10.1021/np0103665.

Saeed, A. 2006. « Synthesis of 6-O-methyl ether of Scorzocreticin and Scorzocreticoside I, metabolites from Sorzonera cretica ». *Journal of Asian Natural Products Research* 8 (5): 417-23. https://doi.org/10.1080/10286020500172632.

Zaika, Maxim A., Norbert Kilian, Katy Jones, et al. 2020. 'Scorzonera Sensu Lato (Asteraceae, Cichorieae) – Taxonomic Reassessment in the Light of New Molecular Phylogenetic and Carpological Analyses'. *PhytoKeys* 137 (January): 1–85. https://doi.org/10.3897/phytokeys.137.46544.

Senecio

Calvo, Joel, Inés Álvarez, Carlos Aedo, and Pieter B. Pelser. 2013. 'A Phylogenetic Analysis and New Delimitation of Senecio Sect. Crociseris (Compositae: Senecioneae), with Evidence of Intergeneric Hybridization'. *TAXON* 62 (1): 127–40. https://doi.org/10.1002/tax.621011.

Staehelina

Antonidaki-Giatromanolaki, Anna. 2006. 'Development of Native Species of Crete in the Urban Landscape: An Investigation of Systems and Plant Reproduction, Adaptability and Fitness of Wild Species in Commercial Horticulture'. Phd, University of Greenwich. https://gala.gre.ac.uk/id/eprint/8086/.

Kotsos, Maria P., Nektarios Aligiannis, Vasillios Myrianthopoulos, Sofia Mitaku, and Leandros Skaltsounis. 2008. 'Sesquiterpene Lactones from Staehelina Fruticosa'. *Journal of Natural Products* 71 (5): 847–51. https://doi.org/10.1021/np070252e.

Marhold, Karol, and Jaromír Kučera. 2016. 'IAPT/IOPB Chromosome Data 21'. *Taxon* 65 (3): 673–76.

Tripleurospermum

Colak, Nesrin, Huseyin Inceer, Jiri Gruz, et al. 2017. 'Antioxidant Capacity of Phenolics in Some Representatives of the Tribe Anthemideae (Asteraceae) from Turkey'. *International Journal of Pharmaceutical Sciences and Research* 8 (8): 3265–77.

Inceer, Huseyin. 2021. 'Lectotypification of the Name Chamaemelum Heterolepis, the Basionym of Tripleurospermum Heterolepis and Taxonomic Notes on T. Tempskyanum (Asteraceae)'. *Botanica Serbica* 45 (2): 347–52.

Inceer, Huseyin, and Sema Hayirlioglu-Ayaz. 2010. 'Chromosome Numbers in Tripleurospermum Sch. Bip. (Asteraceae) and Closely Related Genera: Relationships between Ploidy Level and Stomatal Length'. *Plant Systematics and Evolution* 285 (3): 149–57. https://doi.org/10.1007/s00606-009-0266-5.

Boraginaceae

Alkanna

Assimopoulou, A. N., I. Karapanagiotis, A. Vasiliou, S. Kokkini, et V. P. Papageorgiou. 2006. « Analysis of Alkannin Derivatives from Alkanna Species by High-Performance Liquid Chromatography/Photodiode Array/Mass Spectrometry ». *Biomedical Chromatography* 20 (12): 1359-74. https://doi.org/10.1002/bmc.705.

Assimopoulou, A. N., J. Tappeiner, M. Ganzera, A. Vasiliou, H. Stuppner, and V. P. Papageorgiou. 2011. 'Determination of Alkannin/Shikonin Derivatives in Endemic Greek Alkanna Species'. *Planta Medica* 77 (12): PL93. https://doi.org/10.1055/s-0031-1282742.

Bazos, Ioannis, Ioannis P. Kokkoris, et Panayotis Dimopoulos. 2021. « Diversity of Halophytes and Salt Tolerant Plants at the Species-, Habitats- and High-Rank Syntaxa Level in Greece ». In

Handbook of Halophytes, édité par Marius-Nicusor Grigore, 787-820. Cham: Springer International Publishing. https://doi.org/10.1007/978-3-030-57635-6_26.

Iatrou, Gregoris, Panayiotis Trigas, et Nicolaos Pettas. 2007. « The Vascular Flora of Akrokorinthos Castle and Its Surrounding Area (NE Peloponnese, Greece) », 12.

Rechinger, Von K H. 1965. 'Zur Kenntnis der europäischen Arten der Gattung Alkanna - About the European species of the genus Alkanna'. *Ann. Naturhistor. Mus. Wien* 68 (November): 191–220.

Saito, Maria Lucia. 1984. « Farmacognosia de Cordia ecalyculata Vell. (Boraginaceae) ». Sao Paulo: Universidad de Sao Paulo, Faculdade de Ciencias Farmaceuticas. https://www.teses.usp.br/teses/disponiveis/9/9138/tde-09102015-174213/publico/Maria_Lucia_Saito_Mestrado.pdf.

Semerdjieva, Ivanka, Galya Petrova, Elina Yankova-Tsvetkova, Tsvetelina Doncheva, Nadezhda Kostova, Rozalia Nikolova, et Valtcho D. Zheljazkov. 2020. « Genetic Diversity, Reproductive Capacity and Alkaloids Content in Three Endemic Alkanna Species ». *PLOS ONE* 15 (6): e0233516. https://doi.org/10.1371/journal.pone.0233516.

Tappeiner, Jasmin, Alexandra Vasiliou, Markus Ganzera, Dimitrios Fessas, Hermann Stuppner, Vassilios P. Papageorgiou, et Andreana N. Assimopoulou. 2014. « Quantitative Determination of Alkannins and Shikonins in Endemic Mediterranean Alkanna Species ». *Biomedical Chromatography* 28 (7): 923-33. https://doi.org/10.1002/bmc.3096.

Tufa, T., H. Damianakos, K. Graikou, et I. Chinou. 2016. « A Phytochemical Study of Two Endemic Greek Boraginaceae Plants ». In *Planta Medica*, 82:P328. Georg Thieme Verlag KG. https://doi.org/10.1055/s-0036-1596457.

Tufa, T., H. Damianakos, G. Zengin, K. Graikou, et I. Chinou. 2019. « Antioxidant and Enzyme Inhibitory Activities of Disodium Rabdosiin Isolated from Alkanna Sfikasiana Tan, Vold and Strid ». *South African Journal of Botany*, Special Issue on Enzyme Inhibitors of natural origin., 120 (janvier): 157-62. https://doi.org/10.1016/j.sajb.2018.04.005.

Tufa, Teisa, Harilaos Damianakos, Konstantia Graikou, et Ioanna Chinou. 2017. « Comparative Study of Naphthoquinone Contents of Selected Greek Endemic Boraginaceae Plants - Antimicrobial Activities ». *Natural Product Communications* 12 (2): 1934578X1701200. https://doi.org/10.1177/1934578X1701200209.

Wagner, Wolfgang. 2017. « Field Records of Larvae of Euchalcia Siderifera (Eversmann, 1856) and Euchalcia Chlorocharis (Dufay, 1961) (Lepidoptera, Noctuidae, Plusiinae) in Greece », 4.

Anchusa

Bigazzi, Massimo, and Federico Selvi. 2000. 'Anchusa Samothracica (Boraginaceae), a New Species from the Island of Samothraki, Greece'. *Nordic Journal of Botany* 20 (2): 141–48. https://doi.org/10.1111/j.1756-1051.2000.tb01557.x.

Krigas, Nikos, Georgios Tsoktouridis, Ioannis Anestis, et al. 2021. 'Exploring the Potential of Neglected Local Endemic Plants of Three Mediterranean Regions in the Ornamental Sector: Value Chain Feasibility and Readiness Timescale for Their Sustainable Exploitation'. *Sustainability* 13 (5): 5. https://doi.org/10.3390/su13052539.

Menteli, Viktoria, Nikos Krigas, Manolis Avramakis, Nicholas Turland, and Despoina Vokou. 2019. 'Endemic Plants of Crete in Electronic Trade and Wildlife Tourism: Current Patterns and Implications for Conservation'. *Journal of Biological Research-Thessaloniki* 26 (1): 10. https://doi.org/10.1186/s40709-019-0104-z.

Selvi, Federico, and Massimo Bigazzi. 2003. 'Revision of Genus Anchusa (Boraginaceae-Boragineae) in Greece'. *Botanical Journal of the Linnean Society* 142 (4): 431–54. https://doi.org/10.1046/j.1095-8339.2003.00206.x.

Anchusella

Maloupa, Eleni, Nikos Krigas, Katerina Grigoriadou, Diamanto Lazari, et Georgios Tsoktouridis. 2008. « Conservation Strategies for Native Plant Species and Their Sustainable Exploitation: Case of the Balkan Botanic Garden of Kroussia, N. Greece », 21.

Nepi, Massimo, Federico Selvi, et Ettore Pacini. 2010. « Variation in nectar–sugar profile of Anchusa and allied genera (Boraginaceae) ». *Botanical Journal of the Linnean Society* 162 (4): 616-27. https://doi.org/10.1111/j.1095-8339.2010.01036.x

Lithodora

Papafotiou, M. 2010. « IN VITRO PROPAGATION OF TEMPERATE ZONE WOODY PLANTS WITH POTENTIAL ORNAMENTAL USE ». *Acta Horticulturae*, nº 885 (décembre): 255-62. https://doi.org/10.17660/ActaHortic.2010.885.34.

Papafotiou, M., et A. Kalantzis. 2009. « STUDIES ON IN VITRO PROPAGATION OF LITHODORA ZAHNII ». *Acta Horticulturae*, nº 813 (mars): 465-70. https://doi.org/10.17660/ActaHortic.2009.813.61.

Myosotis

Greuter, Werner. 1981. 'Med-Checklist Notulae, 3'. *Willdenowia* 11 (1): 23–43.

Omphalodes

Strid, Arne, et Kit Tan. 2005. « A New Species of Omphalodes (Boraginaceae) from Southeast Peloponnese, Greece », 4.

Onosma

Damianakos, H., G. Sotiroudis, et I. Chinou. 2010. « Chemical Constituents from the Aerial Parts of Onosma Erecta (Boraginaceae) ». Planta Medica 76 (12): P180. https://doi.org/10.1055/s-0030-1264478.

Damianakos, Harilaos, Malgorzata Jeziorek, Agnieszka Pietrosiuk, et Ioanna Chinou. 2014. « The Chemical Profile of Pyrrolizidine Alkaloids from Selected Greek Endemic Boraginaceae Plants Determined by Gas Chromatography/Mass Spectrometry ». Journal of AOAC INTERNATIONAL 97 (5): 1244-49. https://doi.org/10.5740/jaoacint.SGEDamianakos.

Damianakos, Harilaos, Georgios Sotiroudis, et Ioanna Chinou. 2013. « Pyrrolizidine Alkaloids from Onosma erecta ». Journal of Natural Products 76 (10): 1829-35. https://doi.org/10.1021/np300785g.

Kretsi, Ourania, Nektarios Aligiannis, Alexios Leandros Skaltsounis, et Ioanna B. Chinou. 2003. « Pyrrolizidine Alkaloids from Onosma Leptantha ». Helvetica Chimica Acta 86 (9): 3136-40. https://doi.org/10.1002/hlca.200390254.

Kundakovic, T., N. Fokialakis, S. Dobric, H. Pratsinis, D. Kletsas, N. Kovacevic, et I. Chinou. 2006. « Evaluation of the Anti-Inflammatory and Cytotoxic Activities of Naphthazarine Derivatives from Onosma Leptantha ». Phytomedicine 13 (4): 290-94. https://doi.org/10.1016/j.phymed.2004.10.009.

Löve, Askell. 1983. « IOPB Chromosome Number Reports LXXVIII ». Taxon 32 (1): 138-41.

Panitsa, Maria, Panayiotis Trigas, Dimitrios Kontakos, Anna-Thalassini Valli, et Gregoris Iatrou. 2021. « Natural and cultural heritage interaction: aspects of plant diversity in three East Peloponnesian castles (Greece) and conservation evaluation ». Plant Biosystems - An International Journal Dealing with all Aspects of Plant Biology 0 (0): 1-15. https://doi.org/10.1080/11263504.2021.1889701.

Teppner, H. 2010. « Flower Visitation of Bombus Haematurus KRIECHBAUMER 1870 (Hymenoptera, Apidae) in Graz, Styria », juillet, 6.

Tufa, Teisa, Harilaos Damianakos, Konstantia Graikou, et Ioanna Chinou. 2017. « Comparative Study of Naphthoquinone Contents of Selected Greek Endemic Boraginaceae Plants - Antimicrobial Activities ». Natural Product Communications 12 (2): 1934578X1701200. https://doi.org/10.1177/1934578X1701200209.

Authier, P. 2000. 'An annotated catalogue of the flora of the mount Timfi area (National park of Vikos-Aoos and surroundings - Epirus - North west Greece). 4. Boraginaceae.' Candollea 55 (1): 153–78.

Damianakos, Harilaos, Malgorzata Jeziorek, Agnieszka Pietrosiuk, and Ioanna Chinou. 2014. 'The Chemical Profile of Pyrrolizidine Alkaloids from Selected Greek Endemic Boraginaceae Plants Determined by Gas Chromatography/Mass Spectrometry'. Journal of AOAC INTERNATIONAL 97 (5): 1244–49. https://doi.org/10.5740/jaoacint.SGEDamianakos.

Delipetrou, Pinelopi, and Erotokritos Kalogeropoulos. 2011. Μελέτη του πληθυσμού του απειλούμενο είδους Onosma stridii στο Όρος Καλλίδρομο - Study of the population of the threatened species Onosma stridii on Mt Kallidromo. Ελληνικη Εταιρεια Προστασιας της Φυσης. http://rgdoi.net/10.13140/2.1.1091.6163.

Orfanou, Ioanna Maria, Harilaos Damianakos, Ioannis Bazos, Konstantia Graikou, and Ioanna Chinou. 2016. 'Pyrrolizidine Alkaloids from Onosma Kaheirei Teppner (Boraginaceae)'. Rec. Nat. Prod. 10 (2): 331–227.

Teppner, H. 1988a. 'Onosma kaheirei spec. nova und O. erectum (Boraginaceae) aus Griechenland'. Phyton 28 (1): 115–31.

Teppner, H. 1988b. 'Onosma stridii spec. nova (Boraginaceae) aus Griechenland'. Phyton 28 (2): 271–75.

Tufa, Teisa, Harilaos Damianakos, Konstantia Graikou, and Ioanna Chinou. 2017. 'Comparative Study of Naphthoquinone Contents of Selected Greek Endemic Boraginaceae Plants - Antimicrobial Activities'. Natural Product Communications 12 (2): 1934578X1701200. https://doi.org/10.1177/1934578X1701200209.

Δαράκου, Αικατερίνη. 2021. 'Οι επιπτώσεις των προστατευόμενων περιοχών από την οπτική των τοπικών κοινοτήτων: η περίπτωση του εθνικού πάρκου Δάσους Δαδιάς - Λευκίμης - Σουφλίου - The impacts of protected areas from the perspective of local communities: the case of Dadia - Lefkimi - Soufliou National Park'. Master of Science, Democritus University of Thrace. https://repo.lib.duth.gr/jspui/handle/123456789/13870.

Rindera

Ganos, Christos, Nektarios Aligiannis, Ioanna Chinou, Nikolaos Naziris, Maria Chountoulesi, Tomasz Mroczek, et Konstantia Graikou. 2020. « Rindera Graeca (Boraginaceae) Phytochemical Profile and Biological Activities ». *Molecules* 25 (16): 3625. https://doi.org/10.3390/molecules25163625.

Graikou, Konstantia, Harilaos Damianakos, Christos Ganos, Katarzyna Sykłowska-Baranek, Małgorzata Jeziorek, Agnieszka Pietrosiuk, Christos Roussakis, et Ioanna Chinou. 2021. « Chemical Profile and Screening of Bioactive Metabolites of Rindera Graeca (A. DC.) Bois. & Heldr. (Boraginaceae) In Vitro Cultures ». *Plants* 10 (5): 834. https://doi.org/10.3390/plants10050834.

Sykłowska-Baranek, K., A. Pietrosiuk, K. Graikou, H. Damianakos, M. Jeziorek, et I. Chinou. 2013. « Phenolic Compounds from in Vitro Cultures of Rindera Graeca Boiss. & Feldr. » *Planta Medica* 79 (13): PI27. https://doi.org/10.1055/s-0033-1352117.

Symphytum

Grau, J. 1971. « Cytologische Untersuchungen an Boraginaceae II - Cytological examinations on Boraginaceae II ». *Mitt. Bot. München* 9 (août): 177-94.

Stephanaki, Dimitri. 2009. 'Βοτανικοι πηποι στην Ευρωπη: το παραδειγμα της Ροδου - Botanical gardens in Europe: the example of Rhodes'. Master of Science, T.E.I. Kritis.

Brassicaceae

Aethionema

Karydas, Antony, and Georgia Kamari. 2019. 'Monitoring Six Local Endemic Taxa of the Mt Athos and Assessment According to the IUCN Red List Categories and Criteria'. *Botanika Chronika* 22: 195–208.

Kougioumoutzis, Konstantinos, Panayiota Kotsakiozi, Efthalia Stathi, Panayiotis Trigas, and Aristeidis Parmakelis. 2021. 'Conservation Genetics of Four Critically Endangered Greek Endemic Plants: A Preliminary Assessment'. *Diversity* 13 (4): 4. https://doi.org/10.3390/d13040152.

Pasta, S., A. Perez-Graber, L. Fazan, and B De Montmollin. 2017. *The Top 50 Mediterranean Island Plants, Update 2017*. IUCN/SSC/Mediterranean Plant Specialist Group. https://top50.iucn-mpsg.org/book.

Alyssum

Adamidis, G. C., M. Aloupi, E. Kazakou, and P. G. Dimitrakopoulos. 2014. 'Intra-Specific Variation in Ni Tolerance, Accumulation and Translocation Patterns in the Ni-Hyperaccumulator Alyssum Lesbiacum'. *Chemosphere* 95: 496–502.

Adamidis, George C., Panayiotis G. Dimitrakopoulos, Apostolos Manolis, and Aristotelis C. Papageorgiou. 2014. 'Genetic Diversity and Population Structure of the Serpentine Endemic Ni Hyperaccumulator Alyssum Lesbiacum'. *Plant Systematics and Evolution* 300 (9): 2051–60.

Baker, A. J. M., S. P. McGrath, C. M. D. Sidoli, and R. D. Reeves. 1994. 'The Possibility of in Situ Heavy Metal Decontamination of Polluted Soils Using Crops of Metal-Accumulating Plants'. *Resources, Conservation and Recycling*, Environmental biotechnology in waste treatment and recycling, vol. 11 (1): 41–49. https://doi.org/10.1016/0921-3449(94)90077-9.

Baklanov, I. A., I. V. Seregin, and V. B. Ivanov. 2009. 'Histochemical Analysis of Nickel Distribution in the Hyperaccumulator and Excluder in the Genus Alyssum L.' *Doklady Biological Sciences* 429 (1): 548.

Bianchi, Elisabetta, Aida Bani, Ilaria Colzi, Cristina Gonnelli, and Federico Selvi. 2022. 'Exploring Ni-Accumulation in Serpentinophytic Taxa of Brassicaceae from Albania and Greece'. *Plant Biosystems - An International Journal Dealing with All Aspects of Plant Biology* 0 (0): 1–11. https://doi.org/10.1080/11263504.2022.2098870.

Brooks, R. R., C. C. Radford, and Richard Ellis Ford Matthews. 1978. 'Nickel Accumulation by European Species of the Genus Alyssum'. *Proceedings of the Royal Society of London. Series B. Biological Sciences* 200 (1139): 217–24. https://doi.org/10.1098/rspb.1978.0016.

Cecchi, Lorenzo, Roberto Gabbrielli, Miluscia Arnetoli, Cristina Gonnelli, Agim Hasko, and Federico Selvi. 2010. 'Evolutionary Lineages of Nickel Hyperaccumulation and Systematics in European Alysseae (Brassicaceae): Evidence from nrDNA Sequence Data'. *Annals of Botany* 106 (5): 751–67. https://doi.org/10.1093/aob/mcq162.

Cetlová, Veronika, Judita Zozomová-Lihová, Andrea Melichárková, Lenka Mártonfiová, and Stanislav Španiel. 2021. 'Multiple Drivers of High Species Diversity and Endemism Among Alyssum Annuals in the Mediterranean: The Evolutionary Significance of the Aegean Hotspot'. *Frontiers in Plant Science* 12 (April): 627909. https://doi.org/10.3389/fpls.2021.627909.

Dudley, T. R. 1964. 'Studies in Alyssum: Near Eastern Representatives and Their Allies, I'. *Journal of the Arnold Arboretum.* 45 (1): 57–100. https://doi.org/10.5962/p.185679.

Dudley, T. R., and D. Christodoulakis. 1989. 'Alyssum Samium: A New Endemic from Samos'. *Notes from the Royal Botanic Garden, Edinburgh* 45 (3): 433–38.

Homer, F. A., R. S. Morrison, R. R. Brooks, J. Clemens, and R. D. Reeves. 1991. 'Comparative Studies of Nickel, Cobalt, and Copper Uptake by Some Nickel Hyperaccumulators of the Genus Alyssum'. *Plant and Soil* 138 (2): 195–205. https://doi.org/10.1007/BF00012246.

Ingle, R. A., M. D. Fricker, and J. A. C. Smith. 2008. 'Evidence for Nickel/Proton Antiport Activity at the Tonoplast of the Hyperaccumulator Plant Alyssum Lesbiacum'. *Plant Biology* 10 (6): 746–53.

Ingle, Robert A., J. Andrew C. Smith, and Lee J. Sweetlove. 2005. 'Responses to Nickel in the Proteome of the Hyperaccumulator Plant Alyssum Lesbiacum'. *Biometals* 18 (6): 627–41.

Karetsos, George, Alexandra D. Solomou, Panayiotis Trigas, and Konstantinia Tsagari. 2018. 'The Vascular Flora of Mt. Oiti National Park and the Surrounding Area in Greece'. *Journal of Forest Science* 64 (No. 10): 435–54. https://doi.org/10.17221/65/2018-JFS.

Kazakis, G., D. Ghosn, I. N. Vogiatzakis, and V. P. Papanastasis. 2007. 'Vascular Plant Diversity and Climate Change in the Alpine Zone of the Lefka Ori, Crete'. *Biodiversity and Conservation* 16 (6): 1603–15. https://doi.org/10.1007/s10531-006-9021-1.

Kazakou, Elena, G. C. Adamidis, Panayiotis G. Dimitrakopoulos, Alan J. M. Baker, Roger D. Reeves, and Malinda Godino. 2008. *Alyssum Lesbiacum: A New Ni-Hyperaccumulator from Lesbos*. http://ptaba.gr/dyn/banner/051716134706_b.pdf.

Kazakou, Elena, George C. Adamidis, Alan J. M. Baker, Roger D. Reeves, Malinda Godino, and Panayiotis G. Dimitrakopoulos. 2010. 'Species Adaptation in Serpentine Soils in Lesbos Island (Greece): Metal Hyperaccumulation and Tolerance'. *Plant and Soil* 332 (1): 369–85. https://doi.org/10.1007/s11104-010-0302-9.

Kerkeb, Loubna, and Ute Krämer. 2003. 'The Role of Free Histidine in Xylem Loading of Nickel inAlyssum Lesbiacum and Brassica Juncea'. *Plant Physiology* 131 (2): 716–24. https://doi.org/10.1104/pp102.010686.

Krämer, U., G. W. Grime, J. A. C. Smith, C. R. Hawes, and A. J. M. Baker. 1997. 'Micro-PIXE as a Technique for Studying Nickel Localization in Leaves of the Hyperaccumulator Plant Alyssum Lesbiacum'. *Nuclear Instruments and Methods in Physics Research Section B: Beam Interactions with Materials and Atoms* 130 (1–4): 346–50.

Küpper, Hendrik, Enzo Lombi, Fang-Jie Zhao, Gerlinde Wieshammer, and Steve P. McGrath. 2001. 'Cellular Compartmentation of Nickel in the Hyperaccumulators Alyssum Lesbiacum, Alyssum Bertolonii and Thlaspi Goesingense'. *Journal of Experimental Botany* 52 (365): 2291–300. https://doi.org/10.1093/jexbot/52.365.2291.

McGrath, S. P., C. M. D. Sidoli, A. J. M. Baker, and R. D. Reeves. 1993. 'The Potential for the Use of Metal-Accumulating Plants for the in Situ Decontamination of Metal-Polluted Soils'. In *Integrated Soil and Sediment Research: A Basis for Proper Protection*, edited by Herman J. P. Eijsackers and Timo Hamers. Soil & Environment. Springer Netherlands. https://doi.org/10.1007/978-94-011-2008-1_145.

Morrison, R. S., R. R. Brooks, and R. D. Reeves. 1980. 'Nickel Uptake by Alyssum Species'. *Plant Science Letters* 17 (4): 451–57. https://doi.org/10.1016/0304-4211(80)90132-7.

Nedelkoska, T. V., and P. M. Doran. 2000. 'Characteristics of Heavy Metal Uptake by Plant Species with Potential for Phytoremediation and Phytomining'. *Minerals Engineering* 13 (5): 549–61. https://doi.org/10.1016/S0892-6875(00)00035-2.

Orcan, Nermin, and Riza Binzet. 2006. 'A New Record for the Flora of Turkey: Alyssum Idaeum Boiss. & Heldr.' *Pak. J. Bot.* 38 (4): 931–33.

Psaras, G. 2000. ‘Relative Abundance of Nickel in the Leaf Epidermis of Eight Hyperaccumulators: Evidence That the Metal Is Excluded from Both Guard Cells and Trichomes’. *Annals of Botany* 86 (1): 73–78. https://doi.org/10.1006/anbo.2000.1161.

Reich, Dieter, Walter Gutermann, Katharina Bardy, et al. 2021. ‘The Type Specimens in Eugen von Halácsy´s Herbarium Graecum’. *Phytotaxa* 493 (1): 1–156. https://doi.org/10.11646/phytotaxa.493.1.1.

Singer, Andrew C., Thomas Bell, Chloe A. Heywood, J. A. C. Smith, and Ian P. Thompson. 2007. ‘Phytoremediation of Mixed-Contaminated Soil Using the Hyperaccumulator Plant Alyssum Lesbiacum: Evidence of Histidine as a Measure of Phytoextractable Nickel’. *Environmental Pollution* 147 (1): 74–82.

Smart, K. E., M. R. Kilburn, C. J. Salter, J. A. C. Smith, and C. R. M. Grovenor. 2007. ‘NanoSIMS and EPMA Analysis of Nickel Localisation in Leaves of the Hyperaccumulator Plant Alyssum Lesbiacum’. *International Journal of Mass Spectrometry* 260 (2–3): 107–14.

Španiel, Stanislav, Karol Marhold, and Judita Zozomová-Lihová. 2017. ‘The Polyploid Alyssum Montanum-A. Repens Complex in the Balkans: A Hotspot of Species and Genetic Diversity’. *Plant Systematics and Evolution* 303 (10): 1443–65. https://doi.org/10.1007/s00606-017-1470-3.

Trigas, Panayiotis, and Gregoris Iatrou. 2006. ‘The Local Endemic Flora of Evvia (W Aegean, Greece)’. *Willdenowia* 36 (1): 257–70. https://doi.org/10.3372/wi.36.36121.

Turland, Nicholas J. 2008. ‘Anthemis Samariensis (Asteraceae, Anthemideae), a New Species from the Mountains of W Kriti (Greece)’. *Willdenowia* 38 (1): 61. https://doi.org/10.3372/wi.38.38103.

Αμπατζίδης, Ράλλης, and Χρήστος Μπακιρτζής. 2018. ‘Η αμυντική λειτουργία του νικελίου στον υπερσυσσωρευτή Alyssum lesbiacum - The defensive function of nickel in the hyperaccumulator Alyssum lesbiacum’. Bachelor Thesis, University of Aigaio. http://hellanicus.lib.aegean.gr/handle/11610/19353.

Μάστορας, Πέτρος, and Μαρία Ιωάννα Παπαδάκη. 2016. ‘Συγκέντρωση και μάζα Ni στα διαφορετικά στάδια ανάπτυξης του ενδημικού είδους Alyssum lesbiacum της Λέσβου - Concentration and mass of Ni in the different growth stages of the endemic species Alyssum lesbiacum of Lesbos’. Bachelor Thesis, University of Aigaio. http://hellanicus.lib.aegean.gr/handle/11610/18373.

Σκοπιανός, Δημήτριος. 2018. ‘Συγκέντρωση Ni στα ενδημικά φυτικά είδη της Λέσβου, Alyssum lesbiacum και Alyssum xiphocarpum, μετά την ανάπτυξή τους σε καλλιέργειες διαφορετικής ποικιλότητας υπό διαφορετικούς τύπους υποστρώματος - Ni concentration in the endemic plant species of Lesvos, Alyssum lesbiacum and Alyssum xiphocarpum, after their growth in crops of different diversity under different substrate types’. Bachelor Thesis, University of Aigaio. http://hellanicus.lib.aegean.gr/handle/11610/23171.

Χριστοφάκη, Μαρία. 2008. ‘Διερεύνηση της καταλληλότητας των φυτικών ειδών Nicotiana Glauca και Alyssum Baldaccii για βιοεξυγίανση ρυπασμένων εδάφων με βαρέα μέταλλα. - Investigation of the suitability of the plant species Nicotiana Glauca and Alyssum Baldaccii for bioremediation of soils contaminated with heavy metals.’ Bachelor Thesis, TEI of Crete. https://apothesis.lib.hmu.gr/handle/20.500.12688/819.

Aubrieta

Karydas, Antony, and Georgia Kamari. 2019. ‘Monitoring Six Local Endemic Taxa of the Mt Athos and Assessment According to the IUCN Red List Categories and Criteria’. *Botanika Chronika* 22: 195–208.

Koch, Marcus A., Robert Karl, and Dmitry A. German. 2017. ‘Underexplored Biodiversity of Eastern Mediterranean Biota: Systematics and Evolutionary History of the Genus Aubrieta (Brassicaceae)’. *Annals of Botany* 119 (1): 39–57. https://doi.org/10.1093/aob/mcw204.

Maloupa, E., K. Grigoriadou, D. Zervaki, and K. Papanastassi. 2005. ‘Management of the Balkan Native Flora for Sustainable Floricultural Commercial Use’. *Acta Horticulturae*, no. 683 (June): 189–96. https://doi.org/10.17660/ActaHortic.2005.683.21.

Maloupa, Eleni, Nikos Krigas, Katerina Grigoriadou, Diamanto Lazari, and Georgios Tsoktouridis. 2008. *Conservation Strategies for Native Plant Species and Their Sustainable Exploitation: Case of the Balkan Botanic Garden of Kroussia, N. Greece*. 21.

Muhammed, Jotyar Jassim. 2017. ‘Systematic and Genomic Studies in the Genus Aubrieta (Brassicaceae)’. PhD Thesis, University of Leicester.

Psaras, G. 2000. ‘Relative Abundance of Nickel in the Leaf Epidermis of Eight Hyperaccumulators: Evidence That the Metal Is Excluded from Both Guard Cells and Trichomes’. *Annals of Botany* 86 (1): 73–78. https://doi.org/10.1006/anbo.2000.1161.

Τζακαλος, Φιλοχρηστος. 2011. ‘Μελετη εθνικων δρυμον Ελλαδας: χλωριδα, πανιαδα και καθεστως διαχειρισης - Study of national parks of Greece: flora, fauna and management regime’. Bachelor Thesis, T.E.I. Kavalas. http://digilib.teiemt.gr/jspui/bitstream/123456789/3852/1/TSAKALOS.pdf.

Bornmuellera

Bianchi, Elisabetta, Aida Bani, Ilaria Colzi, Cristina Gonnelli, and Federico Selvi. 2022. ‘Exploring Ni-Accumulation in Serpentinophytic Taxa of Brassicaceae from Albania and Greece’. *Plant Biosystems - An International Journal Dealing with All Aspects of Plant Biology* 0 (0): 1–11. https://doi.org/10.1080/11263504.2022.2098870.

Chardot, Vanessa, Stamatia Tina Massoura, Guillaume Echevarria, Roger D. Reeves, and Jean-Louis Morel. 2005. ‘Phytoextraction Potential of the Nickel Hyperaccumulators Leptoplax Emarginata and Bornmuellera Tymphaea’. *International Journal of Phytoremediation* 7 (4): 323–35. https://doi.org/10.1080/16226510500327186.

Durand, A., S. Piutti, M. Rue, J. L. Morel, G. Echevarria, and E. Benizri. 2016. ‘Improving Nickel Phytoextraction by Co-Cropping Hyperaccumulator Plants Inoculated by Plant Growth Promoting Rhizobacteria’. *Plant and Soil* 399 (1): 179–92. https://doi.org/10.1007/s11104-015-2691-2.

Morrison, Richard Stephen. 1980. ‘Aspects of the Accumulation of Cobalt, Copper and Nickel by Plants : A Thesis Presented in Partial

Fulfilment of the Requirements for the Degree of Doctor of Philosophy in Chemistry, Massey University'. Thesis, Massey University. https://mro.massey.ac.nz/handle/10179/3606.

Peer, Wendy Ann, Mehrzad Mahmoudian, John L. Freeman, et al. 2006. 'Assessment of Plants from the Brassicaceae Family as Genetic Models for the Study of Nickel and Zinc Hyperaccumulation'. *New Phytologist* 172 (2): 248–60. https://doi.org/10.1111/j.1469-8137.2006.01820.x.

Rue, Marie, Jessica Vallance, Guillaume Echevarria, et al. 2015. 'Phytoextraction of Nickel and Rhizosphere Microbial Communities under Mono- or Multispecies Hyperaccumulator Plant Cover in a Serpentine Soil'. *Australian Journal of Botany* 63 (2): 92–102. https://doi.org/10.1071/BT14249.

Tognacchini, Alice, Theresa Rosenkranz, Antony van der Ent, Gaylord Erwan Machinet, Guillaume Echevarria, and Markus Puschenreiter. 2020. 'Nickel Phytomining from Industrial Wastes: Growing Nickel Hyperaccumulator Plants on Galvanic Sludges'. *Journal of Environmental Management* 254 (January): 109798. https://doi.org/10.1016/j.jenvman.2019.109798.

Brassica

Andersson, Meike S., and M. Carmen de Vicente. 2010. *Gene Flow Between Crops and Their Wild Relatives*. JHU Press.

Aziz, Muhammad Abdul, Amir Hasan Khan, Muhammad Adnan, and Izatullah Izatullah. 2017. 'Traditional Uses of Medicinal Plants Reported by the Indigenous Communities and Local Herbal Practitioners of Bajaur Agency, Federally Administrated Tribal Areas, Pakistan'. *Journal of Ethnopharmacology* 198 (February): 268–81. https://doi.org/10.1016/j.jep.2017.01.024.

Biswas, Sayani, Ambarish Mukherjee, and Jiban K. Pal. 2017. 'Flowering Seasons of Some Common Ethnomedicinal Plants of Bankura District, West Bengal (India)'. *Indian J. Sci. Res.* 15 (1): 54–60.

Iatrou, Gregoris, Panayiotis Trigas, et Nicolaos Pettas. 2007. « The Vascular Flora of Akrokorinthos Castle and Its Surrounding Area (NE Peloponnese, Greece) », 12.

Katsakiori, Maria. 2015. *Κείμενα έκθεσης του Κέντρου Πληροφόρησης` Επισκεπτών Εθνικού Δρυμού Ολύμπου στην αγγλική γλώσσα - Exhibition texts of the Olympus National Forest Visitor Information Center in English*. Φορεας Διαχειρισης Εθνικου Δρυμου Ολυμπου. http://repository.biodiversity-info.gr/bitstream/11340/1933/1/1680.pdf.

Lannér, C., T. Bryngelsson, et M. Gustafsson. 1997. « Relationships of Wild Brassica Species with Chromosome Number 2n = 18, Based on RFLP Studies ». *Genome* 40 (3): 302-8. https://doi.org/10.1139/g97-042.

Sikorska-Zimny, Kalina, et Luciano Beneduce. 2020. « The glucosinolates and their bioactive derivatives in Brassica: a review on classification, biosynthesis and content in plant tissues, fate during and after processing, effect on the human organism and interaction with the gut microbiota ». *Critical Reviews in Food Science and Nutrition* 0 (0): 1-28. https://doi.org/10.1080/10408398.2020.1780193.

Cardamine

Perný, Marián, Andreas Tribsch, and Mincho E. Anchev. 2004. 'Infraspecific Differentiation in the Balkan diploidCardamine Acris (Brassicaceae): Molecular and Morphological Evidence'. *Folia Geobotanica* 39 (4): 405–29. https://doi.org/10.1007/BF02803211.

Šlenker, Marek, Marián Perný, Judita Zozomová-Lihová, and Karol Marhold. 2021. 'Taxonomic Position and Circumscription of Cardamine Barbaraeoides (Brassicaceae), a Systematically Challenging Taxon from the Balkan Peninsula'. *Phytotaxa* 502 (2): 2. https://doi.org/10.11646/phytotaxa.502.2.1.

Tan, Kit, Konstantinos Giannopoulos, and Gert Vold. 2013. 'Cardamine Calliphaea Sp. Nov. (Brassicaceae) from Southwestern Greece'. *Nordic Journal of Botany* 31 (3): 282–85. https://doi.org/10.1111/j.1756-1051.2012.00045.x.

Draba

Krigas, Nikos, Viktoria Menteli, and Despoina Vokou. 2014. 'The Electronic Trade in Greek Endemic Plants: Biodiversity, Commercial and Legal Aspects'. *Economic Botany* 68 (1): 85–95. https://doi.org/10.1007/s12231-014-9264-9.

Krigas, Nikos, Georgios Tsoktouridis, Ioannis Anestis, et al. 2021. 'Exploring the Potential of Neglected Local Endemic Plants of Three Mediterranean Regions in the Ornamental Sector: Value Chain Feasibility and Readiness Timescale for Their Sustainable Exploitation'. *Sustainability* 13 (5): 5. https://doi.org/10.3390/su13052539.

Löve, Áskell. 1982. 'IOPB Chromosome Number Reports LXXV'. *Taxon* 31 (2): 342–68.

Erysimum

Al-Gendy, A. A., O. D. El-gindi, Al. S. Hafez, et A. M. Ateya. 2010. « Glucosinolates, Volatile Constituents and Biological Activities of Erysimum Corinthium Boiss. (Brassicaceae) ». *Food Chemistry* 118 (3): 519-24. https://doi.org/10.1016/j.foodchem.2009.05.009.

Bergmeier, E., M. Ristow, S. Meyer, and M. Panitsa. 2021. 'Phytodiversity of Limnos (North Aegean, Greece)—an Update and Evaluation'. *Flora Mediterranea* 31: 233–46. https://doi.org/10.7320/FlMedit31.233.

Boissier, Edmond. 1849. *Diagnoses plantarum orientalium novarum*. Apud B. Herrmann.

Çalişkan, Gültekin. 2020. « A Study of Herbs Used in Food in Rize Province within the Context of Gastronomy Tourisme ». *International Journal of Social Humanities Sciences Research (JSHSR)* 7 (51): 573-83. https://doi.org/10.26450/jshsr.1789.

Cullaj, A, A Hasko, I McBow, et F Kongoli. 2004. « Investigation of the Potential of Several Plants for Phytoremediation of Nickel Contaminated Soils and for Nickel Phytoextraction », 8.

Delforge, Pierre. 1997. 'Les orchidées de l'île d'Amorgos (Cyclades, Grèce)'. *Natural. belges* 78 (Orchid. 10): 103–52.

Delitheos, A., E. Tiligada, A. Yannitsaros, et I. Bazos. 1997. « Antiphage Activity in Extracts of Plants Growing in Greece ». *Phytomedicine* 4 (2): 117-24. https://doi.org/10.1016/S0944-7113(97)80055-4.

Franzén, R., et L.-Å. Gustavsson. 1983. « Chromosome Numbers in Flowering Plants from the High Mountains of Sterea Ellas, Greece ». *Willdenowia* 13 (1): 101-6.

Gkika, Paraskevi I., Nikos Krigas, George Menexes, Ilias G. Eleftherohorinos, and Eleni Maloupa. 2013. 'Effect of Temperature and Light on Seed Germination of Erysimum Naxense and Erysimum Krendlii'. *Central European Journal of Biology* 8 (12): 1194–203. https://doi.org/10.2478/s11535-013-0229-2.

Greuter, Werner, and Thomas Raus. 1983. 'Med-Checklist Notulae, 7'. *Willdenowia* 13: 79–99.

Heldreich, Theodor von. 1882. *Flore de l'ile de Cephalonie: ou, Catalogue des plantes qui croissent naturellement et se cultivent le plus fréquemment dans cette ile*. Bridel.

Iatrou, Gregoris, Panayiotis Trigas, et Nicolaos Pettas. 2007. « The Vascular Flora of Akrokorinthos Castle and Its Surrounding Area (NE Peloponnese, Greece) », 12.

Kamari, Georgia, Dimitrios Phitos, Britt Snogerup, and Sven Snogerup. 1988. 'Flora and Vegetation of Yioura, N Sporades, Greece'. *Willdenowia* 17 (1/2): 59–85.

Kougioumoutzis, K., A. Tiniakou, O. Georgiou, and T. Georgiadis. 2015. 'Contribution to the Flora and Biogeography of the Kiklades: Folegandros Island (Kiklades, Greece)'. *Edinburgh Journal of Botany* 72 (3): 391–412. https://doi.org/10.1017/S0960428615000128.

Krigas, Nikos, Viktoria Menteli, and Despoina Vokou. 2014. 'The Electronic Trade in Greek Endemic Plants: Biodiversity, Commercial and Legal Aspects'. *Economic Botany* 68 (1): 85–95. https://doi.org/10.1007/s12231-014-9264-9.

Moazzeni, Hamid, Shahin Zarre, Bernard E. Pfeil, et al. 2014. 'Phylogenetic Perspectives on Diversification and Character Evolution in the Species-Rich Genus Erysimum (Erysimeae; Brassicaceae) Based on a Densely Sampled ITS Approach'. *Botanical Journal of the Linnean Society* 175 (4): 497–522. https://doi.org/10.1111/boj.12184.

Polatschek, Adolf. 1982. 'Erysimum Canum Und E. Hayekii (Brassicaceae) / Erysimum Canum and E. Hayekii (Brassicaceae)'. *Plant Systematics and Evolution* 140 (4): 321–23.

Sarropoulou, Virginia, and Eleni Maloupa. 2019. 'Vegetative Propagation of Three Prioritized Greek Endemics with Potential Commercial Interest: Erysimum Naxense Snogerup, Erysimum Krendlii Polatschek and Centaurea Paxorum Phitos & Georgiadis'. *GSC Biological and Pharmaceutical Sciences* 6 (1): 1. https://doi.org/10.30574/gscbps.2019.6.1.0002.

Strid, Arne. 1981. 'New Species of Cephalaria (Dipsacaceae) and Stipa (Gramineae) from the Greek Mountains'. *Willdenowia* 11 (2): 301–5.

Thomas, Konstantinos, Ricos Thanopoulos, Helmut Knüpffer, and Penelope J. Bebeli. 2012. 'Plant Genetic Resources of Lemnos (Greece), an Isolated Island in the Northern Aegean Sea, with Emphasis on Landraces'. *Genetic Resources and Crop Evolution* 59 (7): 1417–40. https://doi.org/10.1007/s10722-011-9770-x.

Hesperis

Parolly, Gerald, and Kit Tan. 2006. 'A New Species of Hesperis (Brassicaceae) from SW Anatolia, Turkey'. *Willdenowia* 36 (2): 851–56. https://doi.org/10.3372/wi.36.36215.

Iberis

Delforge, Pierre. 1997. 'Les orchidées de l'île d'Amorgos (Cyclades, Grèce)'. *Natural. belges* 78 (Orchid. 10): 103–52.

Isatis

Papanicolaou, Kostas. 1983. 'Isatis Vermia Sp.Nov. from North Central Greece Materials for the Mountain Flora of Greece, 14'. *Nordic Journal of Botany* 2 (6): 553–56. https://doi.org/10.1111/j.1756-1051.1983.tb01048.x.

Leptoplax

Bartoli, François, David Coinchelin, Christophe Robin, and Guillaume Echevarria. 2012. 'Impact of Active Transport and Transpiration on Nickel and Cadmium Accumulation in the Leaves of the Ni-Hyperaccumulator Leptoplax Emarginata: A Biophysical Approach'. *Plant and Soil* 350 (1): 99–115. https://doi.org/10.1007/s11104-011-0885-9.

Bartoli, François, Mathilde Royer, David Coinchelin, et al. 2018. 'Multiscale and Age-Dependent Leaf Nickel in the Ni-Hyperaccumulator Leptoplax Emarginata'. *Ecological Research* 33 (4): 723–36. https://doi.org/10.1007/s11284-018-1594-0.

Chardot, Vanessa, Stamatia Tina Massoura, Guillaume Echevarria, Roger D. Reeves, and Jean-Louis Morel. 2005. 'Phytoextraction Potential of the Nickel Hyperaccumulators Leptoplax Emarginata and Bornmuellera Tymphaea'. *International Journal of Phytoremediation* 7 (4): 323–35. https://doi.org/10.1080/16226510500327186.

Chardot-Jacques, Vanessa, Christophe Calvaruso, Bruno Simon, Marie-Pierre Turpault, Guillaume Echevarria, and Jean-Louis Morel. 2013. 'Chrysotile Dissolution in the Rhizosphere of the Nickel Hyperaccumulator Leptoplax Emarginata'. *Environmental Science & Technology* 47 (6): 2612–20. https://doi.org/10.1021/es301229m.

Coinchelin, D., D. Stemmelen, and F. Bartoli. 2014. 'A Simple Model for Estimating Ni Availability and Leaf Ni Accumulation for the Ni-Hyperaccumulator Leptoplax Emarginata'. *Plant and Soil* 374 (1): 131–47. https://doi.org/10.1007/s11104-013-1873-z.

Coinchelin, David. 2011. 'Mécanismes et modélisation de l'accumulation foliaire du nickel par l'hyperaccumulateur Leptoplax emarginata - Mecanism and modelization of the leaf accumulation of Nickel by hyperaccumulator Leptoplax emarginata'. PhD Thesis, Institut National Polytechnique de Lorraine - INPL. https://tel.archives-ouvertes.fr/tel-01502547.

Constantinidis, Theophanidis, Eleftheria-Perdiko Bareka, and Georgia Kamari. 2002. 'Karyotaxonomy of Greek Serpentine Angiosperms'. *Botanical Journal of the Linnean Society* 139 (1): 109–24. https://doi.org/10.1046/j.1095-8339.2002.00044.x.

Hazotte, Claire, Baptiste Laubie, Stéphanie Pacault, Olivier Dufaud, and Marie-Odile Simonnot. 2020. 'Evaluation of the Performance of Nickel Hyperaccumulator Plants as Combustion Fuel'. *Biomass and Bioenergy* 140 (September): 105671. https://doi.org/10.1016/j.biombioe.2020.105671.

Hazotte, Claire, Baptiste Laubie, Ramez Saad, et al. 2017. ‘Caractérisation de plantes hyperaccumulatrices pour la production d’énergie et de sels de nickel - Characterization of hyperaccumulating plants for the production of energie and Nickel salts’. 110 (July): Papier #3.10. https://hal.univ-lorraine.fr/hal-02354414.

Lucisine, Pierre, Guillaume Echevarria, Thibault Sterckeman, Jessica Vallance, Patrice Rey, and Emile Benizri. 2014. ‘Effect of Hyperaccumulating Plant Cover Composition and Rhizosphere-Associated Bacteria on the Efficiency of Nickel Extraction from Soil’. *Applied Soil Ecology* 81 (September): 30–36. https://doi.org/10.1016/j.apsoil.2014.04.011.

Montargès-Pelletier, Emmanuelle, Vanessa Chardot, Guillaume Echevarria, Laurent J. Michot, Allan Bauer, and Jean-Louis Morel. 2008. ‘Identification of Nickel Chelators in Three Hyperaccumulating Plants: An X-Ray Spectroscopic Study’. *Phytochemistry* 69 (8): 1695–709. https://doi.org/10.1016/j.phytochem.2008.02.009.

Pardo, Tania, Beatriz Rodríguez-Garrido, Ramez F. Saad, et al. 2018. ‘Assessing the Agromining Potential of Mediterranean Nickel-Hyperaccumulating Plant Species at Field-Scale in Ultramafic Soils under Humid-Temperate Climate’. *Science of The Total Environment* 630 (July): 275–86. https://doi.org/10.1016/j.scitotenv.2018.02.229.

Psaras, G. K., Th. Constantinidis, B. Cotsopoulos, and Y. Manetas. 2000. ‘Relative Abundance of Nickel in the Leaf Epidermis of Eight Hyperaccumulators: Evidence That the Metal Is Excluded from Both Guard Cells and Trichomes’. *Annals of Botany* 86 (1): 73–78. https://doi.org/10.1006/anbo.2000.1161.

Redjala, Tanegmart, Thibault Sterckeman, Samia Skiker, and Guillaume Echevarria. 2010. ‘Contribution of Apoplast and Symplast to Short Term Nickel Uptake by Maize and Leptoplax Emarginata Roots’. *Environmental and Experimental Botany* 68 (1): 99–106. https://doi.org/10.1016/j.envexpbot.2009.10.010.

Zhang, Xin, Vivian Houzelot, Aida Bani, Jean Louis Morel, Guillaume Echevarria, and Marie-Odile Simonnot. 2014. ‘Selection and Combustion of Ni-Hyperaccumulators for the Phytomining Process’. *International Journal of Phytoremediation* 16 (10): 1058–72. https://doi.org/10.1080/15226514.2013.810585.

Malcolmia

Al-Shehbaz, Ihsan A., Dmitry A. German, Klaus Mummenhoff, and Hamid Moazzeni. 2014. ‘Systematics, Tribal Placements, and Synopses of the Malcolmia S.L. Segregates (Brassicaceae)’. *Harvard Papers in Botany* 19 (1): 53–71. https://doi.org/10.3100/hpib.v19iss1.2014.n4.

Delitheos, A., E. Tiligada, A. Yannitsaros, et I. Bazos. 1997. « Antiphage Activity in Extracts of Plants Growing in Greece ». *Phytomedicine* 4 (2): 117-24. https://doi.org/10.1016/S0944-7113(97)80055-4.

Iliadou, Eleni, Ioannis Bazos, Konstantinos Kougioumoutzis, et al. 2020. ‘Taxonomic and Phylogenetic Diversity Patterns in the Northern Sporades Islets Complex (West Aegean, Greece)’. *Plant Systematics and Evolution* 306 (2): 28. https://doi.org/10.1007/s00606-020-01660-0.

Panitsa, Maria, Britt Snogerup, Sven Snogerup, and Dimitrios Tzanoudakis. 2003. ‘Floristic Investigation of Lemnos Island (NE Aegean Area, Greece)’. *Willdenowia* 33 (1): 79–105. https://doi.org/10.3372/wi.33.33108.

Noccaeae

Broadley, Martin R., Philip J. White, John P. Hammond, Ivan Zelko, et Alexander Lux. 2007. « Zinc in Plants ». *New Phytologist* 173 (4): 677-702. https://doi.org/10.1111/j.1469-8137.2007.01996.x.

Meyer, Friedrich Karl. 1986. ‘Eine Neue Noccaea-Art (Cruciferae) von Kreta - A New Species of Noccaea (Cruciferae) from Crete’. *Willdenowia* 15 (2): 389–91.

Reeves, R. D., and R. R. Brooks. 1983. ‘European Species of Thlaspi L. (Cruciferae) as Indicators of Nickel and Zinc’. *Journal of Geochemical Exploration* 18 (3): 275–83. https://doi.org/10.1016/0375-6742(83)90073-0.

Odontarrhena

Cecchi, Lorenzo, Stanislav Španiel, Elisabetta Bianchi, Andrea Coppi, Cristina Gonnelli, and Federico Selvi. 2020. ‘Odontarrhena Stridii (Brassicaceae), a New Nickel-Hyperaccumulating Species from Mainland Greece’. *Plant Systematics and Evolution* 306 (4): 69. https://doi.org/10.1007/s00606-020-01687-3.

Hopewell, Tyler, Federico Selvi, Hans-Jürgen Ensikat, and Maximilian Weigend. 2021. ‘Trichome Biomineralization and Soil Chemistry in Brassicaceae from Mediterranean Ultramafic and Calcareous Soils’. *Plants* 10 (2): 377.

Jakovljevic, Ksenija, Aida Bani, Dolja Pavlova, et al. 2022. ‘Hyperaccumulator Plant Discoveries in the Balkans: Accumulation, Distribution, and Practical Applications’. *Botanica Serbica* 46 (2): 161–78. https://doi.org/10.2298/BOTSERB2202161J.

Raparia

Mandáková, Terezie, Vasantika Singh, Ute Krämer, and Martin A. Lysak. 2015. ‘Genome Structure of the Heavy Metal Hyperaccumulator Noccaea Caerulescens and Its Stability on Metalliferous and Nonmetalliferous Soils’. *Plant Physiology* 169 (1): 674–89. https://doi.org/10.1104/pp.15.00619.

Ricotia

Mandáková, Terezie, Xinyi Guo, Barış Özüdoğru, Klaus Mummenhoff, and Martin A. Lysak. 2018. ‘Hybridization-Facilitated Genome Merger and Repeated Chromosome Fusion after 8 Million Years’. *The Plant Journal* 96 (4): 748–60. https://doi.org/10.1111/tpj.14065.

Rorippa

Delforge, Pierre. 1997. ‘Les orchidées de l’île d’Amorgos (Cyclades, Grèce)’. *Natural. belges* 78 (Orchid. 10): 103–52.

Campanulaceae

Asyneuma

Cellinese, Nicoletta, Stephen A. Smith, Erika J. Edwards, et al. 2009. 'Historical Biogeography of the Endemic Campanulaceae of Crete'. *Journal of Biogeography* 36 (7): 1253–69. https://doi.org/10.1111/j.1365-2699.2008.02077.x.

Spiliotis, Panagiotis. 2015. 'Are European Threatened Species Adequately Preserved in Ex Situ Institutions? Limitations of the IUCN Red List as a Guideline for Ex Situ Plant Conservation and a Proposal for an Integrated Strategy in Assessing the Conservation Importance and Value of a Species in Ex Situ Plant Collections.' Master of Science, University of Edinburgh.

Campanula / Halacsyella

Bergmeier, E., M. Ristow, S. Meyer, and M. Panitsa. 2021. 'Phytodiversity of Limnos (North Aegean, Greece)—an Update and Evaluation'. *Flora Mediterranea* 31: 233–46. https://doi.org/10.7320/FlMedit31.233.

Biel, Burkhard, and Kit Tan. 2010. 'A New Species of Campanula (Campanulaceae) from the Island of Samothraki, NE Greece'. *Phytologia Balcanica* 16 (3): 351–53.

Carlström, Annette. 1986. 'A Revision of the Campanula Drabifolia Complex (Campanulaceae)'. *Willdenowia* 15 (2): 375–87.

Cattaneo, Cristina, and Mauro Grano. 2019. 'Checklist Updating and Analysis of the Flora of Symi Island and of the Nearby Island of Seskli (Dodecanese, Greece)'. *Bocconea* 28: 425–63.

Cattaneo, Cristina, and Mauro Grano. 2021. *Kasos: An Unexpected Island. Floristic and Ecological Analysis of Kasos Island (SE Aegean, Dodecanese, Greece), with Noteworthy Floristic Additions*. 28.

Cellinese, Nicoletta, Stephen A. Smith, Erika J. Edwards, et al. 2009. 'Historical Biogeography of the Endemic Campanulaceae of Crete'. *Journal of Biogeography* 36 (7): 1253–69. https://doi.org/10.1111/j.1365-2699.2008.02077.x.

Christodoulakis, D, R Artelari, Th Georgiadis, and D Tzanoudakis. 2001. 'New Records to the Flora of Fourni (E. Aegean Islands, Greece)'. *Bocconea* 13: 491–94.

Christodoulakis, Dimitrios. 1996. 'The Phytogeographical Distribution Patterns of the Flora of Ikaria (E Aegean, Greece) within the E Mediterranean'. *Flora* 191 (4): 393–99. https://doi.org/10.1016/S0367-2530(17)30748-X.

Constantinidis, Theophanis. 2013. 'The Flora of the Kastellorizo Island Group (East Aegean Islands, Greece): New Records and Comments'. *Flora Mediterranea* 23 (December): 69–86. https://doi.org/10.7320/FlMedit23.069.

Contandriopoulos, Mlle J. 1964. « Contribution à l'étude caryologique des Campanulacées de Grèce ». *Bulletin de la Société Botanique de France* 111 (5-6): 222-35. https://doi.org/10.1080/00378941.1964.10838197.

Dehnen-Schmutz, Katharina, Julia Touza, Charles Perrings, et Mark Williamson. 2007. « The Horticultural Trade and Ornamental Plant Invasions in Britain ». *Conservation Biology* 21 (1): 224-31. https://doi.org/10.1111/j.1523-1739.2006.00538.x.

Diapoulis, Char. 1959. 'Conservation Measures for the Plants of the Greek Flora'. *Revue d'Ecologie, Terre et Vie, Société Nationale de Protection de La Nature* Sup: 189–91.

Dietrich, Volker Jörg. 2018. 'Geography, Nature and Habitation of Nisyros Volcano'. In *Nisyros Volcano: The Kos - Yali - Nisyros Volcanic Field*, edited by Volker Jörg Dietrich and Evangelos Lagios. Active Volcanoes of the World. Springer International Publishing. https://doi.org/10.1007/978-3-319-55460-0_1.

Fokialakis, Nikolas, Charles L. Cantrell, Stephen O. Duke, Alexios L. Skaltsounis, and David E. Wedge. 2006. 'Antifungal Activity of Thiophenes from Echinops Ritro'. *Journal of Agricultural and Food Chemistry* 54 (5): 1651–55. https://doi.org/10.1021/jf052702j.

Goulimis, C. 1958. *Report on Species of Plants Requiring Protection in Greece and Measures for Securing Their Protection*. Athens. http://documents.irevues.inist.fr/bitstream/handle/2042/59422/LATERREETLAVIE_1959_Sup_168.pdf?sequence=1.

Grigoriadou, K., N. Krigas, and E. Maloupa. 2014. 'GIS-Facilitated Ex Situ Conservation of the Rare Greek Endemic Campanula Incurva Aucher: Seed Germination Requirements and Effect of Growth Regulators on in Vitro Proliferation and Rooting'. *Plant Biosystems - An International Journal Dealing with All Aspects of Plant Biology* 148 (6): 1169–77. https://doi.org/10.1080/11263504.2014.980355.

Hartvig, Per. 1998. 'Campanula Pangea, a New Species of C. Sect. Involucratae from Mt Pangeon, NE Greece'. *Willdenowia* 28 (1/2): 65–68.

Iatrou, Gregoris, Panayiotis Trigas, et Nicolaos Pettas. 2007. « The Vascular Flora of Akrokorinthos Castle and Its Surrounding Area (NE Peloponnese, Greece) », 12.

Iliadou, Eleni, Ioannis Bazos, Konstantinos Kougioumoutzis, et al. 2020. 'Taxonomic and Phylogenetic Diversity Patterns in the Northern Sporades Islets Complex (West Aegean, Greece)'. *Plant Systematics and Evolution* 306 (2): 28. https://doi.org/10.1007/s00606-020-01660-0.

Jensen, Gert, Rune Harboe Nielsen, and Arne Skytt Andersen. 2005. 'Campanula (Campanulaceae) in Nature and in Pots©'. *Combined Proceedings International Plant Propagators' Society* 55: 310–18.

Kalpoutzakis, Eleftherios, Theophanis Constantinidis, and Panayiotis Trigas. 2019. *Chorological Additions for Some Noteworthy Taxa of the Greek Flora*. 18.

Kamari, Georgia. 2013. 'The Present and Future of the Flora of Greece and Its Conservation Assessment'. *Flora Mediterranea* 23 (December): 183–94. https://doi.org/10.7320/FlMedit23.183.

Karras, George, Ioannis Tsirogiannis, and Evaggelos Fillis. 2013. 'A Case Study of an in Situ Botanical Garden in Zagori, Greece'. *Journal of Agricultural Science and Technology* 3 (B): 823–31.

Khoshbakht, Korous, and Karl Hammer. 2007. 'Threatened and Rare Ornamental Plants'. *Journal of Agriculture and Rural Development in the Tropics and Subtropics (JARTS)* 108 (1): 1.

Klimienė, Asta, Rimanta Vainorienė, and Roberta Dubosaitė. 2011. 'Rare Plants in Ex Situ Collection in Botanic Garden of Siauliai University'. *Acta Biol. Univ. Daugavp.* 11 (2): 106–12.

Kovos, Dimitrios, and Karagiannis Georgios. 2018. 'Field Study on the "National Marine Park" of Alonissos, Greece'. *Journal of Tourism Research* 19 (June): 243–53.

https://www.researchgate.net/profile/Celio-Marques-2/publication/359095716_The_Internet_as_Place_Branding_strategy_in_tourist_destinations_management_a_proposal_for_practical_application/links/6227d6e83c53d31ba4b2e03a/The-Internet-as-Place-Branding-strategy-in-tourist-destinations-management-a-proposal-for-practical-application.pdf#page=243.

Krigas, Nikos, Viktoria Menteli, and Despoina Vokou. 2014. 'The Electronic Trade in Greek Endemic Plants: Biodiversity, Commercial and Legal Aspects'. *Economic Botany* 68 (1): 85–95. https://doi.org/10.1007/s12231-014-9264-9.

Krigas, Nikos, Marina Panagiotidou, and Eleni Maloupa. 2017. 'Incorporating Biogeographical Principles in Horticulture: Design and Creation of the Ionian Islands Unique Rock Garden in Thessaloniki, Greece'. *Sibbaldia: The Journal of Botanic Garden Horticulture* 0 (15): 129–46.

Kull, Ulrich, et Stergos Diamantoglou. 1987. « The Flora, Vegetation and Monuments of Classical Greece ». https://doi.org/10.18419/opus-2403.

Kyriakopoulos, Ch., Pepy Bareka, et Georgia Kamari. 2016. « Karyological Data of Some Endemic Taxa from Mt Taigetos, Greece ». *Flora Mediterranea* 26 (décembre). https://doi.org/10.7320/FlMedit26.224.

Liveri, Eleni, Pepy Bareka, and Georgia Kamari. 2020. 'Karyosystematic Study of Some Taxa from Campanula Section Quinqueloculares (Campanulaceae). I.' *Flora Mediterranea* 30. https://doi.org/10.7320/FlMedit30.440.

Maloupa, Eleni, Nikos Krigas, Katerina Grigoriadou, Diamanto Lazari, and Georgios Tsoktouridis. 2008. *Conservation Strategies for Native Plant Species and Their Sustainable Exploitation: Case of the Balkan Botanic Garden of Kroussia, N. Greece*. 21.

Menteli, Viktoria, Nikos Krigas, Manolis Avramakis, Nicholas Turland, and Despoina Vokou. 2019. 'Endemic Plants of Crete in Electronic Trade and Wildlife Tourism: Current Patterns and Implications for Conservation'. *Journal of Biological Research-Thessaloniki* 26 (1): 10. https://doi.org/10.1186/s40709-019-0104-z.

Müller, Andreas. 2015. « Palaearctic Chelostoma Bees of the Subgenus Gyrodromella (Megachilidae, Osmiini): Biology, Taxonomy and Key to Species ». *Zootaxa* 3936 (3): 408. https://doi.org/10.11646/zootaxa.3936.3.6.

Özdöl, Tuğkan, Özal Güner, Abdurrahman Sefali, Ekrem AkçiÇek, Tuncay DiRmenci, and Hasan Yildirim. 2022. 'Three New Records for the Flora of Turkey: Campanula Lyrata Subsp. Icarica (Campanulaceae), Erysimum Aureum (Brassicaceae) and Stachys Benthamiana (Lamiaceae)'. *Phytotaxa* 531 (2): 147–50. https://doi.org/10.11646/phytotaxa.531.2.8.

Peñuela, Adriana Guerrero. 2009. 'Estudio etnobotanico de las huertas familiares y su papel en la seguridad alimentaria y la sostenebilidad economica de los campesinos del municipio de Cogua (Cundinamarca) - Ethnobotanical study of family gardens and their role in food security and economic sustainability of peasants in the municipality of Cogua (Cundinamarca)'. Bachelor Thesis, Pontificia Universidad Javeriana.

Phitos, Dimitrios. 2021. 'Reinstating Campanula Nisyria as a Distinct Species of Sect. Quinqueloculares (Campanulaceae)'. *Flora Mediterranea* 31: 101–7. https://doi.org/10.7320/FlMedit31.101.

Psaroudaki, Antonia, Petros Dimitropoulakis, Theophanis Constantinidis, Andreas Katsiotis, and George N. Skaracis. 2012. 'Ten Indigenous Edible Plants: Contemporary Use in Eastern Crete, Greece'. *Culture, Agriculture, Food and Environment* 34 (2): 172–77. https://doi.org/10.1111/j.2153-9561.2012.01076.x.

Quezel, P. 1964. 'Végétation des Hautes Montagnes de la Grèce Méridionale - High moutains vegetation in Meridional Greece'. *Vegetatio Acta Geobotanica* 12 (5–6): 289–385. https://doi.org/10.1007/BF03026056.

Raab-Straube, Eckhard Von. 2014. 'Euro Med-Checklist Notulae, 3'. *Willdenowia* 44 (2): 287–99. https://doi.org/10.3372/wi.44.44211.

Sarropoulou, Virginia, Nikos Krigas, Georgios Tsoktouridis, Eleni Maloupa, and Katerina Grigoriadou. 2022. 'Seed Germination Trials and Ex Situ Conservation of Local Prioritized Endemic Plants of Crete (Greece) with Commercial Interest'. *Seeds* 1 (4): 4. https://doi.org/10.3390/seeds1040024.

Siljak-Yakovlev, Sonja, Perla Farhat, Nicolas Valentin, Pepy Bareka, and Georgia Kamari. 2019. *New Estimates of Nuclear DNA Amount for 25 Taxa from Kefallinia Island*. 23.

Skoula, M, C Dal Cin D'Agata, and A Sarpaki. 2009. 'Contribution to the Ethnobotany of Crete, Greece'. *Bocconea* 23: 479–87.

Strid, Arne. 2015. 'Reliquiae Runemarkianae. Chromosome Numbers of Angiosperms from the Aegean Islands'. *Phytologia Balcanica* 21 (3): 245–93.

Tan, Kit, and Burkhard Biel. 2011. 'Nomenclatural Note'. *Phytologia Balcanica* 17 (2): 265.

Trigas, Panayiotis, and Gregoris Iatrou. 2006. 'The Local Endemic Flora of Evvia (W Aegean, Greece)'. *Willdenowia* 36 (1): 257–70. https://doi.org/10.3372/wi.36.36121.

Turland, Nicholas J. 2006. « Lectotypification of Campanula saxatilis, Phyteuma pinnatum and Verbascum arcturus, Linnaean Names of Three Taxa Endemic to Crete ». *Willdenowia* 36 (1): 303-9.

Γιαννούλης, Δημήτρης. 2008. 'Χρήση ενδημικών φυτών στην Κρήτη - Use of the native plants in Crete'. Bachelor Thesis, TEI of Crete. https://apothesis.lib.hmu.gr/handle/20.500.12688/956.

Λαγογιάννης, Γεώργιος Ιωακείμ. 2019. 'Φυτοχημική μελέτη του αυτοφυούς εδώδιμου είδους της ανατολικής Κρήτης Campanula pelviformis (Campanulaceae) - Phytochemical study of the edible species of eastern Crete Campanula pelviformis (Campanulaceae)'. Aristotle University Of Thessaloniki (AUTH); Αριστοτέλειο Πανεπιστήμιο Θεσσαλονίκης (ΑΠΘ).

Μαξούτογλου, Μαργαρίτα Ι. 2014. 'Τουριστική ανάπτυξη απομακρυσμένων νησιωτικών περιοχών: μελέτη περίπτωσης νήσου Νισύρου - Tourism development of remote island areas: a case study of the island of Nisyros'. Master Thesis, University of Peiraios. https://dione.lib.unipi.gr/xmlui/handle/unipi/6190.

Φασές, Παναγιώτης. 2009. 'Διερεύνηση, ομαδοποίηση, σύνθεση και εφαρμογή στη διαχείριση της μέχρι τώρα ερευνητικής δραστηριότητας στο Εθνικό Θαλάσσιο Πάρκο της Αλοννήσου,

Βόρειες Σποράδες - Investigation, grouping, synthesis and application in the management of research activity so far in the National Marine Park of Alonissos, Northern Sporades'. Bachelor Thesis, University of Thessaly. https://ir.lib.uth.gr/xmlui/bitstream/handle/11615/1425/P0001425.pdf?sequence=1&isAllowed=y.

Φραντζεσκάκη, Αντωνία. 2006. 'Επίδραση του paclobutrazol, της φωτοπεριόδου και της έντασης του φωτισμού, την ανάπτυξη και άνθιση της δενδρώδους αγγελικής (Pittosporum undulatum) και της καμπανούλας (Campanula pelviformis).' Τεχνολογικό Εκπαιδευτικό Ίδρυμα. https://apothesis.lib.hmu.gr/bitstream/handle/20.500.12688/568/2006Frantzeskaki.pdf?sequence=1.

Ψαρουδάκη, Αντωνία. 2015. *Καταγραφή, βοτανική ταυτοποίηση, γενετική ποικιλότητα και ιδιότητες αυτοφυών εδώδιμων φυτών της Κρήτης. Συμμετοχή τους στο σύγχρονο διατροφικό πρότυπο - Recording, botanical identification, genetic diversity and properties of native edible plants of Crete. Their participation in the modern dietary model*. September 25. http://gaia.aua.gr/xmlui/handle/10329/6132.

Legousia

Strid, Arne. 2015. 'Reliquiae Runemarkianae. Chromosome Numbers of Angiosperms from the Aegean Islands'. *Phytologia Balcanica* 21 (3): 245–93.

Tan, Kit, Burkhard Biel, and Giorgos Sfikas. 2015. 'Legousia Snogerupii (Campanulaceae), a New Species from Southeastern Kiklades, Greece'. *Phytotaxa* 201 (1): 1. https://doi.org/10.11646/phytotaxa.201.1.4.

Petromarula

Fokialakis, Nikolas, Charles L. Cantrell, Stephen O. Duke, Alexios L. Skaltsounis, and David E. Wedge. 2006. 'Antifungal Activity of Thiophenes from Echinops Ritro'. *Journal of Agricultural and Food Chemistry* 54 (5): 1651–55. https://doi.org/10.1021/jf052702j.

Heywood, Vernon Hilton. 1999. 'Chapter 3: Contributions to Rural Households'. In *Use and Potential of Wild Plants in Farm Households*. FAO Farm Systems Management Series 15. Food & Agriculture Organization of the United Nations.

Krigas, Nikos, Viktoria Menteli, and Despoina Vokou. 2014. 'The Electronic Trade in Greek Endemic Plants: Biodiversity, Commercial and Legal Aspects'. *Economic Botany* 68 (1): 85–95. https://doi.org/10.1007/s12231-014-9264-9.

Libiad, Mohamed, Abdelmajid Khabbach, Mohamed El Haissoufi, et al. 2021. 'Agro-Alimentary Potential of the Neglected and Underutilized Local Endemic Plants of Crete (Greece), Rif-Mediterranean Coast of Morocco and Tunisia: Perspectives and Challenges'. *Plants* 10 (9): 9. https://doi.org/10.3390/plants10091770.

Menteli, Viktoria, Nikos Krigas, Manolis Avramakis, Nicholas Turland, and Despoina Vokou. 2019. 'Endemic Plants of Crete in Electronic Trade and Wildlife Tourism: Current Patterns and Implications for Conservation'. *Journal of Biological Research-Thessaloniki* 26 (1): 10. https://doi.org/10.1186/s40709-019-0104-z.

Morales Gómez, Patricia. 2011. 'Vegetales silvestres de uso alimentario: determinación de compuestos bioactivos y valoración de la capacidad antioxidante - Forest plants of food use: determination of bioactive compounds and evaluation of their antioxidant activity'. PhD in biology, Universidad Complutense Madrid. https://eprints.ucm.es/id/eprint/14444/.

Nikolakaki, Sophie. 2005. *Landscapes for Learning – Rediscovering the Mediterranean Landscapes – & the Case of University of Crete's Woodland in Rethymnon.* 6.

Pieroni, Andrea, Naji Sulaiman, and Renata Sõukand. 2022. 'Chorta (Wild Greens) in Central Crete: The Bio-Cultural Heritage of a Hidden and Resilient Ingredient of the Mediterranean Diet'. *Biology* 11 (5): 5. https://doi.org/10.3390/biology11050673.

Rackham, Oliver, and Jennifer Moody. 1996. *The Making of the Cretan Landscape*. Manchester University Press.

Sarropoulou, Virginia, Nikos Krigas, Georgios Tsoktouridis, Eleni Maloupa, and Katerina Grigoriadou. 2022. 'Seed Germination Trials and Ex Situ Conservation of Local Prioritized Endemic Plants of Crete (Greece) with Commercial Interest'. *Seeds* 1 (4): 4. https://doi.org/10.3390/seeds1040024.

Skoula, M, C Dal Cin D'Agata, and A Sarpaki. 2009. 'Contribution to the Ethnobotany of Crete, Greece'. *Bocconea* 23: 479–87.

Vasilopoulou, Effie, and Antonia Trichopoulou. 2011. 'Green Pies: The Flavonoid Rich Greek Snack'. *Food Chemistry* 126 (3): 855–58. https://doi.org/10.1016/j.foodchem.2010.11.051.

Μαρκακη, Ελενη. 2006. 'Εκτός τόπου" διατήρηση (Ex-situ) των ενδημικών φυτών της Κρήτης - "Ex-situ" conservation of the endemic plants of Crete.' Bachelor Thesis, Ανώτατο Τεχνολογικό Εκπαιδευτικό Ίδρυμα Κρήτης.

Solenopsis

Christodoulou, Charalambos S, Ralf Hand, and Kontantinos Iosif. 2020. 'Solenopsis Annua Comb. Nov., a New Taxon for Cyprus'. *Cypricola* 17 (September): 1–8.

Menteli, Viktoria, Nikos Krigas, Manolis Avramakis, Nicholas Turland, and Despoina Vokou. 2019. 'Endemic Plants of Crete in Electronic Trade and Wildlife Tourism: Current Patterns and Implications for Conservation'. *Journal of Biological Research-Thessaloniki* 26 (1): 10. https://doi.org/10.1186/s40709-019-0104-z.

Caryophyllaceae

Agrostemma

Tsogkas, Pantelis. 2021. 'Σχέδιο Δράσης του Δήμου Φαρσάλων για το μετριασμό της κλιματικής αλλαγής και την προσαρμογή σε αυτή - Action Plan of the Municipality of Farsala for mitigating climate change and adapting to it'. Master of Science, Ethniko Metsobio Polytechneio.

Arenaria

Bazos, Ioannis, Ioannis P. Kokkoris, et Panayotis Dimopoulos. 2021. « Diversity of Halophytes and Salt Tolerant Plants at the Species-, Habitats- and High-Rank Syntaxa Level in Greece ». In

Handbook of Halophytes, édité par Marius-Nicusor Grigore, 787-820. Cham: Springer International Publishing. https://doi.org/10.1007/978-3-030-57635-6_26.

Brofas, G., Panayiotis Trigas, G. Mantakas, G. Karetsos, Costas A. Thanos, Kyriacos Georghiou, et Chr. Mermiris. 2007. « Rehabilitation of disturbed areas by mining activities in high floristic diversity areas: the case of Mt Giona ». In . Milos island, Greece. http://publicationslist.org/data/kgeorghi/ref-41/%CE%9419.%20Floristic%20diversity%20Giona.pdf.

Carlström, Annette. 1986. 'A Revision of Arenaria Sect. Orientales Ser. Orientales Incl. Ser. Deflexae (Caryophyllaceae) in the Aegean and SW Turkey'. *Willdenowia* 15 (2): 359–74.

Christodoulakis, Dimitrios. 1996. 'The Phytogeographical Distribution Patterns of the Flora of Ikaria (E Aegean, Greece) within the E Mediterranean'. *Flora* 191 (4): 393–99. https://doi.org/10.1016/S0367-2530(17)30748-X.

Kamari, Georgia, Dimitrios Phitos, Britt Snogerup, and Sven Snogerup. 1988. 'Flora and Vegetation of Yioura, N Sporades, Greece'. *Willdenowia* 17 (1/2): 59–85.

Κωστέλης, Απόστολος. 2018. 'Οι Ανωδασικές Φυτοκοινότητες Του Όρους Παγγαίου - The Upland Plant Communities of Mount Pangaeus'. Master Thesis, Δημοκρίτειο Πανεπιστήμιο Θράκης. https://repo.lib.duth.gr/jspui/handle/123456789/13781.

Bolanthus

Phitos, D, N Turland, and E Bergmeier. 2011. 'A New Subspecies of Bolanthus Creutzburgii (Caryophyllaceae) from Coastal SW Kriti (Greece)'. *Flora Mediterranea*.

Trigas, Panayiotis, Gregoris Iatrou, and Giorgos Karetsos. 2007. 'Species Diversity, Endemism and Conservation of the Family Caryophyllaceae in Greece'. *Biodiversity and Conservation* 16 (2): 357–76. https://doi.org/10.1007/s10531-005-3013-4.

Zografidis, Aris, Ori Fragman-Sapir, Arne Strid, and Panayotis Dimopoulos. 2020. 'Notes on the Generic Name Graecobolanthus (Caryophylleae, Caryophyllaceae)'. *TAXON* 69 (5): 992–97. https://doi.org/10.1002/tax.12292.

Cerastium

Couladis, Maria, et Olga Tzakou. 2000. « Volatile Constituents of Cerastium candidissimum Corr. from Greece ». *Journal of Essential Oil Research* 12 (6): 691-92. https://doi.org/10.1080/10412905.2000.9712192.

Franzén, R., and L.-Å. Gustavsson. 1983. 'Chromosome Numbers in Flowering Plants from the High Mountains of Sterea Ellas, Greece'. *Willdenowia* 13 (1): 101–6.

Greuter, Werner, Niels Böhling, and Ralf Jahn. 2002. 'The Cerastium Scaposum Group (Caryophyllaceae): Three Annual Taxa Endemic to Crete (Greece), Two of Them New'. *Willdenowia* 32 (1): 45–54. https://doi.org/10.3372/wi.32.32103.

Iatrou, Gregoris, Panayiotis Trigas, et Nicolaos Pettas. 2007. « The Vascular Flora of Akrokorinthos Castle and Its Surrounding Area (NE Peloponnese, Greece) », 12.

Krigas, Nikos, Viktoria Menteli, et Despoina Vokou. 2014. « The Electronic Trade in Greek Endemic Plants: Biodiversity, Commercial and Legal Aspects ». *Economic Botany* 68 (1): 85-95. https://doi.org/10.1007/s12231-014-9264-9.

Krigas, Nikos, Marina Panagiotidou, et Eleni Maloupa. 2017. « Incorporating biogeographical principles in horticulture: design and creation of the ionian islands unique rock garden in Thessaloniki, Greece. » *Sibbaldia: The Journal of Botanic Garden Horticulture*, 2017, 15 édition.

Lazari, Diamanto M., Helen D. Skaltsa, et Theophanis Constantinidis. 2000. « Volatile Constituents of Cerastium Candidissimum, a Greek Endemic Species ». *Flavour and Fragrance Journal* 15 (3): 174-76. https://doi.org/10.1002/1099-1026(200005/06)15:3<174::AID-FFJ886>3.0.CO;2-Y.

Niketić, Marjan, Sanja Z Đurović, Gordana Tomović, Peter Schönswetter, and Božo Frajman. 2022. 'Diversification within Ploidy-Variable Balkan Endemic Cerastium Decalvans (Caryophyllaceae) Reconstructed Based on Genetic, Morphological and Ecological Evidence'. *Botanical Journal of the Linnean Society* 199 (2): 578–608. https://doi.org/10.1093/botlinnean/boab037.

Stamatis, George, Panayiotis Kyriazopoulos, Stamatina Golegou, Aris Basayiannis, Spyros Skaltsas, et Helen Skaltsa. 2003. « In Vitro Anti-Helicobacter Pylori Activity of Greek Herbal Medicines ». *Journal of Ethnopharmacology* 88 (2): 175-79. https://doi.org/10.1016/S0378-8741(03)00217-4.

Strid, Arne. 2020. 'The Botanical Exploration of Greece'. *Plant Systematics and Evolution* 306 (2): 27. https://doi.org/10.1007/s00606-020-01637-z.

Trigas, Panayiotis, Gregoris Iatrou, and Giorgos Karetsos. 2007. 'Species Diversity, Endemism and Conservation of the Family Caryophyllaceae in Greece'. *Biodiversity and Conservation* 16 (2): 357–76. https://doi.org/10.1007/s10531-005-3013-4.

Zagka, Th. D. 2011. 'Το φυσικο περιβαλλον του Ολυμπου - The natural environment of Olympus'. 15–27. http://repository.edulll.gr/edulll/bitstream/10795/2096/2/2096_ekdosi_2011.pdf.

Dianthus / Gypsophila

Browicz, Kazmierz. 1997. 'Woody Flora of Melos and Kimolos (Cyclades, Greece)'. Arboretum Kornickie Rocznik 42: 45–63.

Cattaneo, Cristina, and Mauro Grano. 2016. Contribution to the Knowledge of Vascular Flora on Astypalea Island (Dodecanese, Greece).

Cattaneo, Cristina, and Mauro Grano. 2019. 'Checklist Updating and Analysis of the Flora of Symi Island and of the Nearby Island of Seskli (Dodecanese, Greece)'. Bocconea 28: 425–63.

Cattaneo, Cristina, and Mauro Grano. 2021. Kasos: An Unexpected Island. Floristic and Ecological Analysis of Kasos Island (SE Aegean, Dodecanese, Greece), with Noteworthy Floristic Additions. 28.

Constantinidis, Theophanis. 1999. 'Dianthus Haematocalyx Subsp. Phitosianus (Caryophyllaceae), a New Serpentine Endemic from Greece'. Phyton (Horn, Austria) 39 (2): 277–91.

Constantinidis, Theophanis. 2004. 'The Floristic Diversity of Serpentine in Greece 1. An Inventory of the Aliki Area (Sterea Ellas, Central Greece)'. Phyton (Horn, Austria) 44 (1): 45–67.

Davis, Peter H. 1938. 'The Flowers of Lasithi'. The Annual of the British School at Athens 38 (November): 146–48. https://doi.org/10.1017/S0068245400012107.

Drossos, Elisseos. 1992. 'A Floristic Study of Mitrikou Lake and the Lagoons of Nomos Rodhopi in W Thrace (N Greece)'. Willdenowia 22: 97–117.

Greuter, Werner, Ursula Matthäs, and Horst Risse. 1984. 'Additions to the Flora of Crete, 1973-1983: I'. Willdenowia 14: 27–36.

Franzén, R., et L.-Å. Gustavsson. 1983. « Chromosome Numbers in Flowering Plants from the High Mountains of Sterea Ellas, Greece ». *Willdenowia* 13 (1): 101-6.

Kougioumoutzis, K., A. Tiniakou, O. Georgiou, and T. Georgiadis. 2014. 'Contribution to the Flora of the South Aegean Volcanic Arc: Kimolos Island (Kiklades, Greece)'. Edinburgh Journal of Botany 71 (2): 135–60. https://doi.org/10.1017/S0960428614000055.

Kougioumoutzis, Konstantinos, Argyro Tiniakou, Ourania Georgiou, and Theodoros Georgiadis. 2012. 'Contribution to the Flora of the South Aegean Volcanic Arc: Anafi Island (Kiklades, Greece)'. Willdenowia 42 (1): 127–41. https://doi.org/10.3372/wi.42.42115.

Krigas, N., A. Vergou, and E. Maloupa. 2007. 'What's New in a Deciduous Oak Forest? The Path of Biodiversity in the Balkan Botanic Garden of Kroussia, N Greece'. Paper presented at 3rd Global Botanic Gardens Congress, Wuhan, China. https://www.bgci.org/files/Wuhan/PaperEd/Maloupa,%20Eleni%20-%20Greece.pdf.

Krigas, Nikos, Marina Panagiotidou, et Eleni Maloupa. 2017. « Incorporating biogeographical principles in horticulture: design and creation of the ionian islands unique rock garden in Thessaloniki, Greece. » *Sibbaldia: The Journal of Botanic Garden Horticulture*, 2017, 15 édition.

Krigas, Nikos, Georgios Tsoktouridis, Ioannis Anestis, et al. 2021. 'Exploring the Potential of Neglected Local Endemic Plants of Three Mediterranean Regions in the Ornamental Sector: Value Chain Feasibility and Readiness Timescale for Their Sustainable Exploitation'. Sustainability 13 (5): 5. https://doi.org/10.3390/su13052539.

Löve, Áskell. 1978. 'IOPB Chromosome Number Reports LXI'. TAXON 27 (4): 375–92. https://doi.org/10.1002/j.1996-8175.1978.tb04261.x.

Maloupa, E., D. Zervaki, K. Grigoriadou, and K. Papanastasi. 2004. 'The Development of a Native Plant Collection Nursery in the Kroussia Balkan Botanic Garden'. Scripta Botanica Belgica 29: 15–20.

Nektarios, Panayiotis A., Ioannis Amountzias, Iro Kokkinou, et Nikolaos Ntoulas. 2011. « Green Roof Substrate Type and Depth Affect the Growth of the Native Species Dianthus Fruticosus Under Reduced Irrigation Regimens ». *HortScience* 46 (8): 1208-16. https://doi.org/10.21273/HORTSCI.46.8.1208.

Rechinger, K. H. 1971. 'Potentilla goulandrii, Dianthus stamatiadae, Onosma psammophilum, drei neue Arten der griechischen Flora - Potentilla goulandrii, Dianthus stamatiadae, Onosma psammophilum, three new species from the flora of Greece'. Botaniska notiser, 75–81.

Sarropoulou, Virginia, Nikos Krigas, Georgios Tsoktouridis, Eleni Maloupa, and Katerina Grigoriadou. 2022. 'Seed Germination Trials and Ex Situ Conservation of Local Prioritized Endemic Plants of Crete (Greece) with Commercial Interest'. Seeds 1 (4): 4. https://doi.org/10.3390/seeds1040024.

Sarropoulou, Virginia, and Eleni Maloupa. 2022. 'Micropropagation and Ex Situ Conservation of Three Rare and Endemic Ornamental Dianthus Taxa (Caryophyllaceae)'. Botanica Serbica 46 (1): 49–60.

Siljak-Yakovlev, Sonja, Perla Farhat, Nicolas Valentin, Pepy Bareka, et Georgia Kamari. 2019. « New Estimates of Nuclear DNA Amount for 25 Taxa from Kefallinia Island », 23.

Strid, Arne, and Kit Tan. 1998. 'Flora Hellenica, Vol. 1'. TAXON 47: 219–21.

Trakolis, Dimitrios, Panagiotis Platis, and Ioannis Meliadis. 2000. 'Biodiversity and Conservation Actions on Mount Voras, Greece'. Environmental Management 26 (2): 145–51. https://doi.org/10.1007/s002670010077.

Trigas, Panayiotis, Gregoris Iatrou, and Giorgos Karetsos. 2007. 'Species Diversity, Endemism and Conservation of the Family Caryophyllaceae in Greece'. Biodiversity and Conservation 16 (2): 357–76. https://doi.org/10.1007/s10531-005-3013-4.

Tsakiri, Maria, Konstantinos Kougioumoutzis, and Gregoris Iatrou. 2016. 'Contribution to the Vascular Flora of Chalki Island (East Aegean, Greece) and Biomonitoring of a Local Endemic Taxon'. Willdenowia 46 (1): 175–90. https://doi.org/10.3372/wi.46.46114.

Turland, Nicholas J. 1992. 'Studies on the Cretan Flora 2. The Dianthus Juniperinus Complex (Caryophyllaceae)'. Bulletin of the British Museum (Natural History, Botany) 22 (2): 165–69.

Tzonev, Rossen, Dolja Pavlova, Daniel Sánchez-Mata, and Vicenta de la Fuente. 2013. 'Contribution to the Knowledge of Bulgarian Serpentine Grasslands and Their Relationships with Balkan Serpentine Syntaxa'. Plant Biosystems - An International Journal Dealing with All Aspects of Plant Biology 147 (4): 955–69. https://doi.org/10.1080/11263504.2013.788573.

Vogiatzakis, I. N., G. H. Griffiths, and A. M. Mannion. 2003. 'Environmental Factors and Vegetation Composition, Lefka Ori Massif, Crete, S. Aegean'. Global Ecology and Biogeography 12 (2): 131–46. https://doi.org/10.1046/j.1466-822X.2003.00021.x.

Voutzourakis, Nikolaos. 2019. 'Effect of Climatic and Agronomic Factors on Animal Health, Milk Yield and Quality Parameters in Sfakiano Dairy Sheep Production Systems in Crete'. Thesis, Newcastle University. http://theses.ncl.ac.uk/jspui/handle/10443/4728.

Γιαννούλης, Δημήτρης. 2008. 'Χρήση ενδημικών φυτών στην Κρήτη - Use of the native plants in Crete'. Bachelor Thesis, TEI of Crete. https://apothesis.lib.hmu.gr/handle/20.500.12688/956.

Κονταξη, Χρυση. 2016. 'Η διαχειριση των υδατικων πορων στον Θεσσαλικο καμπο - The management of water resources in the Thessalian plain'. Bachelor Thesis, Panepistimio Thessalias. https://ir.lib.uth.gr/xmlui/bitstream/handle/11615/47750/14249.pdf?sequence=1.

Μαρκάκη, Ελένη. 2008. '"Εκτός τόπου" διατήρηση (Ex-situ) των ενδημικών φυτών της Κρήτης. - "Off the ground" conservation (ex-situ) of native plant of Crete'. Bachelor Thesis, TEI of Crete. https://apothesis.lib.hmu.gr/handle/20.500.12688/556.

Μεραμβελιωτάκη, Χρυσή, and Αρβανίτης Στέφανος. 2009. Κηποτεχνική μελέτη διαμόρφωσης & ανάπλασης του πάρκου Αϊ-Γιάννη Αρχανών Ν. Ηρακλείου - Horticultural design and renovation of the park of Aï-Yiannis Archanos, Heraklion Prefecture. A.T.E.I. Kritis.

Μπαζάνης, Απόστολος-Εμμανουήλ. 2022. 'Διερεύνηση της οικοφυσιολογίας του σπόρου και του μικροπολλαπλασιασμού του Dianthus cruentus - Investigating the seed ecophysiology and micropropagation of Dianthus cruentus'. Master of Science, Agricultural University of Athens. http://dspace.aua.gr/xmlui/handle/10329/7559.

Herniaria

Stephanaki, Dimitri. 2009. 'Βοτανικοι πηποι στην Ευρωπη: το παραδειγμα της Ροδου - Botanical gardens in Europe: the example of Rhodes'. Master of Science, T.E.I. Kritis.

Trigas, Panayiotis, Gregoris Iatrou, and Giorgos Karetsos. 2007. 'Species Diversity, Endemism and Conservation of the Family Caryophyllaceae in Greece'. *Biodiversity and Conservation* 16 (2): 357–76. https://doi.org/10.1007/s10531-005-3013-4.

Minuartia

Conti, Fabio. 2003. 'Minuartia Graminifolia (Caryophyllaceae), a South-East European Species'. *Botanical Journal of the Linnean Society* 143 (4): 419–32. https://doi.org/10.1111/j.1095-8339.2003.00232.x.

Dillenberger, Markus S., et Joachim W. Kadereit. 2014. « Maximum Polyphyly: Multiple Origins and Delimitation with Plesiomorphic Characters Require a New Circumscription of Minuartia (Caryophyllaceae) ». *TAXON* 63 (1): 64-88. https://doi.org/10.12705/631.5.

Franzén, R., et L.-Å. Gustavsson. 1983. « Chromosome Numbers in Flowering Plants from the High Mountains of Sterea Ellas, Greece ». *Willdenowia* 13 (1): 101-6.

Kamari, Georgia. 1995. 'Minuartia Greuteriana (Caryophyllaceae), A New Species from NE Greece'. *Willdenowia* 25 (1): 99–104.

Konstantinou, Maria, and Ioannis Tsiripidis. 2015. 'Heavy Metal Uptake by Species from Metalliferous Sites in Northern Greece'. *Phytomining Strategic Metals and Other Elements from Naturally Mineralized Soils and Mineral Wastes* 4: 5.

Nunvářová Kabátová, Klára, Filip Kolář, Vlasta Jarolímová, Karol Krak, and Jindřich Chrtek. 2019. 'Does Geography, Evolutionary History or Ecology Drive Ploidy and Genome Size Variation in the Minuartia Verna Group (Caryophyllaceae) across Europe?' *Plant Systematics and Evolution* 305 (10): 1019–40. https://doi.org/10.1007/s00606-019-01621-2.

Paronychia

Médail, Frédéric. 2013. 'The Unique Nature of Mediterranean Island Floras and the Future of Plant Conservation'. Islands and Plants: Preservation and Understanding of Flora on Mediterranean Islands, Recerca, vol. 20: 414. http://lifereneix.cime.es/WebEditor/Pagines/file/Libro%20II%20JORNADES%20BOT%C3%80NICA%20pdf%20baixa.pdf#page=310.

Tan, Kit, and Arne Strid. 2008. 'Paronychia Manfrediana (Caryophyllaceae), a New Species from Northeast Greece'. Phytologia Balcanica 14 (1): 41–44.

Trigas, Panayiotis, Gregoris Iatrou, and Giorgos Karetsos. 2007. 'Species Diversity, Endemism and Conservation of the Family Caryophyllaceae in Greece'. Biodiversity and Conservation 16 (2): 357–76. https://doi.org/10.1007/s10531-005-3013-4.

Petrorhagia

Iatrou, Gregory. 1985. « Petrorhagia Grandiflora Sp. Nov. (Caryophyllaceae) from Greece ». *Nordic Journal of Botany* 5 (5): 441-45. https://doi.org/10.1111/j.1756-1051.1985.tb01673.x.

Trigas, Panayiotis, Konstantinos Kougioumoutzis, Aikaterini Ermidou, and Eleftherios Kalpoutzakis. 2018. 'Multivariate Morphometric Analysis of Petrorhagia Subsect. Saxifragae (Caryophyllaceae) in Greece, with a New Species from SE Peloponnisos: P. Laconica'. *Willdenowia* 48 (1): 137–46. https://doi.org/10.3372/wi.48.48110.

Siljak-Yakovlev, Sonja, Perla Farhat, Nicolas Valentin, Pepy Bareka, et Georgia Kamari. 2019. « New Estimates of Nuclear DNA Amount for 25 Taxa from Kefallinia Island », 23.

Saponaria

Bazos, Ioannis, Ioannis P. Kokkoris, et Panayotis Dimopoulos. 2021. « Diversity of Halophytes and Salt Tolerant Plants at the Species-, Habitats- and High-Rank Syntaxa Level in Greece ». In *Handbook of Halophytes*, édité par Marius-Nicusor Grigore, 787-820. Cham: Springer International Publishing. https://doi.org/10.1007/978-3-030-57635-6_26.

Kougioumoutzis, Konstantinos, Panayiota Kotsakiozi, Efthalia Stathi, Panayiotis Trigas, et Aristeidis Parmakelis. 2021. « Conservation Genetics of Four Critically Endangered Greek Endemic Plants: A Preliminary Assessment ». *Diversity* 13 (4): 152. https://doi.org/10.3390/d13040152.

Samaropoulou, S., P. Bareka, R. Artelari, and G. Kamari. 2013. 'Karyological Studies on Some Endemic and Rare Species of Kefalonia, Ionian Islands, Greece'. *Flora Mediterranea* 23 (December). https://doi.org/10.7320/FlMedit23.215.

Silene

Aplada, E., Th Georgiadis, A. Tiniakou, and M. Theocharopoulos. 2007. 'Phytogeography and Ecological Evaluation of the Flora and Vegetation of Mt Parnitha (Attica, Greece)'. Edinburgh Journal of Botany 64 (2): 185–207. https://doi.org/10.1017/S096042860700087X.

Aydin, Zeynep, Thomas Marcussen, Alaattin Selcuk Ertekin, and Bengt Oxelman. 2014. 'Marginal Likelihood Estimate Comparisons to Obtain Optimal Species Delimitations in Silene Sect.

Cryptoneurae (Caryophyllaceae)'. PLOS ONE 9 (9): e106990. https://doi.org/10.1371/journal.pone.0106990.

Bazos, Ioannis, Ioannis P. Kokkoris, and Panayotis Dimopoulos. 2021. 'Diversity of Halophytes and Salt Tolerant Plants at the Species-, Habitats- and High-Rank Syntaxa Level in Greece'. In Handbook of Halophytes, edited by Marius-Nicusor Grigore. Springer International Publishing. https://doi.org/10.1007/978-3-030-57635-6_26.

Bazos, Ioannis, Ioannis P. Kokkoris, et Panayotis Dimopoulos. 2021. « Diversity of Halophytes and Salt Tolerant Plants at the Species-, Habitats- and High-Rank Syntaxa Level in Greece ». In Handbook of Halophytes, édité par Marius-Nicusor Grigore, 787-820. Cham: Springer International Publishing. https://doi.org/10.1007/978-3-030-57635-6_26.

Carlström, A. 1986. 'New Taxa and Notes from the SE Aegean Area and SW Turkey'. Willdenowia 16: 73–78.

Constantinidis, Theophanidis, Eleftheria-Perdiko Bareka, and Georgia Kamari. 2002. 'Karyotaxonomy of Greek Serpentine Angiosperms'. Botanical Journal of the Linnean Society 139 (1): 109–24. https://doi.org/10.1046/j.1095-8339.2002.00044.x.

Du Pasquier, Pierre-Emmanuel, Daniel Jeanmonod, and Yamama Naciri. 2017. 'Morphological Convergence in the Recently Diversified Silene Gigantea Complex (Caryophyllaceae) in the Balkan Peninsula and South-Western Turkey, with the Description of a New Subspecies'. Botanical Journal of the Linnean Society 183 (3): 474–93. https://doi.org/10.1093/botlinnean/bow016.

Đurović, Sanja, Ksenija Jakovljević, Uroš Buzurović, Marjan Niketić, Nevena Mihailović, and Gordana Tomović. 2016. 'Differences in Trace Element Profiles of Three Subspecies of Silene Parnassica (Caryophyllaceae) Growing on Ophiolitic Substrate'. Australian Journal of Botany 64 (3): 235. https://doi.org/10.1071/BT15166.

Goulimis, C. 1958. Report on Species of Plants Requiring Protection in Greece and Measures for Securing Their Protection. Athens. http://documents.irevues.inist.fr/bitstream/handle/2042/59422/LATERREETLAVIE_1959_Sup_168.pdf?sequence=1.

Greuter, Werner. 1995. 'Studies in Greek Caryophylloideae: Agrostemma, Silene, and Vaccaria'. Willdenowia 25: 105–42.

Hatzilazarou, Stefanos, Ioannis Anestis, Elias Pipinis, et al. 2023. 'GIS-Facilitated Seed Germination of Six Local Endemic Plants of Crete (Greece) and Multifaceted Evaluation in Three Economic Sectors'. Journal of Biological Research - Thessaloniki 30 (0): 0. https://doi.org/10.26262/jbrt.v30i0.9019.

Heller, Joseph. 1976. 'The Biogeography of Enid Landsnails on the Aegean Islands'. Journal of Biogeography 3 (3): 281–92. https://doi.org/10.2307/3038018.

Iatrou, Gregoris, Panayiotis Trigas, et Nicolaos Pettas. 2007. « The Vascular Flora of Akrokorinthos Castle and Its Surrounding Area (NE Peloponnese, Greece) », 12.

Karydas, Antony, and Georgia Kamari. 2019. 'Monitoring Six Local Endemic Taxa of the Mt Athos and Assessment According to the IUCN Red List Categories and Criteria'. Botanika Chronika 22: 195–208.

Krigas, Nikos, Kimon Papadimitriou, and Antonios D. Mazaris. 2012. 'GIS and Ex Situ Plant Conservation'. In Application of Geographic Information Systems, edited by Bhuiyan Monwar Alam. BoD – Books on Demand.

Kyriakopoulos, Ch., Pepy Bareka, et Georgia Kamari. 2016. « Karyological Data of Some Endemic Taxa from Mt Taigetos, Greece ». Flora Mediterranea 26 (décembre). https://doi.org/10.7320/FlMedit26.224.

Löve, Áskell. 1970. « IOPB Chromosome Number Reports. XXVI ». Taxon 19 (2): 264-69.

Maloupa, Eleni, Nikos Krigas, Katerina Grigoriadou, Diamanto Lazari, and Georgios Tsoktouridis. 2008. Conservation Strategies for Native Plant Species and Their Sustainable Exploitation: Case of the Balkan Botanic Garden of Kroussia, N. Greece. 21.

Melzheimer, V. 1978. 'Bemerkungen zur Cytologie einiger Arten der GattungSilene (Caryophyllaceae) aus Zentralgriechenland und Kreta'. Plant Systematics and Evolution 130 (3–4): 203–7. https://doi.org/10.1007/BF00982804.

Melzheimer, V. 1983. « Silene conglomeratica (Caryophyllaceae), eine neue Art aus S-Griechenland (Peloponnes) ». Willdenowia 13 (1): 123-27.

Oxelman, Bengt. 1995. « A Revision of the Silene sedoides-Group (Caryophyllaceae) ». Willdenowia 25 (1): 143-69.

Panitsa, Maria, and Eleni Iliadou. 2011. 'Flora and Phytogeography of the Ionian Islands (Greece)'. Illes i Plantes: Conservacio i Coneixement de La Flora a Les Illes de La Mediterrania, April, 21. https://d1wqtxts1xzle7.cloudfront.net/39457673/Flora_and_Phytogeography_of_the_Ionian_I20151027-31421-11jlqgp-with-cover-page-v2.pdf?Expires=1660147409&Signature=VzX0tI6WvdEsHL7fl9Jan7hmhpm45jWTgnFMn2~I7-00egQPHPH6q9xDYBK4Bk36dxFKAYQiO7AHr7c7f8ftGcYHeZqfG13NRPpXmlVetK1aauoIVJb-V2aqanmbMrAcKBuq7oagjgu2fOUVstRpfPFMOvDy5~JwelK8fFOxC9YpSvfO43rqiXcjC6n8A6xNqoy~MPnX1418gs5JbZ73BTZOAMgmmqzs1gjd8OubDTIkV6W77y0Yn584BdGCqilMj9QDekOthA9UVth3Dqn6MqOpK-BPszXLAAyR~vDBJLCSbBlxsiR3rwXEyRcBFDE9fQWzJFWXehCssigQfEIJuA__&Key-Pair-Id=APKAJLOHF5GGSLRBV4ZA.

Panitsa, Maria, Panayiotis Trigas, Dimitrios Kontakos, Anna-Thalassini Valli, et Gregoris Iatrou. 2021. « Natural and cultural heritage interaction: aspects of plant diversity in three East Peloponnesian castles (Greece) and conservation evaluation ». Plant Biosystems - An International Journal Dealing with all Aspects of Plant Biology 0 (0): 1-15. https://doi.org/10.1080/11263504.2021.1889701.

Popp, Magnus, and Bengt Oxelman. 2001. 'Inferring the History of the Polyploid Silene Aegaea (Caryophyllaceae) Using Plastid and Homoeologous Nuclear DNA Sequences'. Molecular Phylogenetics and Evolution 20 (3): 474–81. https://doi.org/10.1006/mpev.2001.0977.

Reeves, Roger D., Maria Aloupi, Emmanouil I. Daftsis, John A. Stratis, Petros Mastoras, and Panayiotis G. Dimitrakopoulos. 2022. 'Biogeochemical Aspects of the Serpentines of Rhodes (Greece) and Cyprus'. Plant and Soil 472 (1–2): 491–508. https://doi.org/10.1007/s11104-021-05265-5.

Sarropoulou, Virginia, and Eleni Maloupa. 2019. 'Micropropagation and Ex Situ Conservation of Silene Fabaria (L.) Sm. in Sibth. & Sm. Subsp. Domokina Greuter (Caryophyllaceae); an Important Endemic Plant in Greece with Medicinal and Ornamental Value'. JOURNAL OF ADVANCES IN BIOTECHNOLOGY 8 (February): 1044–57. https://doi.org/10.24297/jbt.v8i0.8062.

Sarropoulou, Virginia, Nikos Krigas, Katerina Grigoriadou, George Tsoktouridis, and Eleni Maloupa. 2018. 'Vegetative Propagation of the Range-Restricted Greek Endemic Silene Fabaria (L.) Sm. in Sibth. & Sm. Subsp. Domokina Greuter (Caryophyllaceae)'. International Journal of Botany Studies 3 (5): 37–40.

Siljak-Yakovlev, Sonja, Perla Farhat, Nicolas Valentin, Pepy Bareka, and Georgia Kamari. 2019. New Estimates of Nuclear DNA Amount for 25 Taxa from Kefallinia Island. 23.

Stevanovic, V., Kit Tan, and G. Iatrou. 2003. 'Distribution of the Endemic Balkan Flora on Serpentine I. - Obligate Serpentine Endemics'. Plant Systematics and Evolution 242 (1–4): 149–70. https://doi.org/10.1007/s00606-003-0044-8.

Strid, Arne. 1986. Moutain Flora of Greece. Vol. 1. Cambridge University Press.

Thanos, Costas A. 2014. 'In Situ and Ex Situ Plant Conservation in Greece in the Framework of the Global Strategy for Plant Conservation'. Bullettin of the Gioenia Academy of Natural Sciences of Catania 47 (377/SFE): 377/SFE.

Trigas, Panayiotis, and Gregoris Iatrou. 2006. 'The Local Endemic Flora of Evvia (W Aegean, Greece)'. Willdenowia 36 (1): 257–70. https://doi.org/10.3372/wi.36.36121.

Trigas, Panayiotis, Eleftherios Kalpoutzakis, Epaminondas Kalogiannis, et al. 2021. 'Noteworthy New Floristic Records from Greece'. Botanica Serbica 45 (2): 321–31. https://doi.org/10.2298/BOTSERB2102321T.

Trigas, Panayiotis, Gregoris Iatrou, and Giorgos Karetsos. 2007. 'Species Diversity, Endemism and Conservation of the Family Caryophyllaceae in Greece'. Biodiversity and Conservation 16 (2): 357–76. https://doi.org/10.1007/s10531-005-3013-4.

Trigas, Panayiotis, Ioannis Kokkoris, and Konstantinos Kougioumoutzis. 2017. 'The Rediscovery of Silene Guicciardii (Caryophyllaceae) on Mt. Parnassos (Greece) after 160 Years: Taxonomic Re-Evaluation and Conservation'. Phytotaxa 331 (2): 281. https://doi.org/10.11646/phytotaxa.331.2.12.

Vogiatzakis, I. N., G. H. Griffiths, and A. M. Mannion. 2003. 'Environmental Factors and Vegetation Composition, Lefka Ori Massif, Crete, S. Aegean'. Global Ecology and Biogeography 12 (2): 131–46. https://doi.org/10.1046/j.1466-822X.2003.00021.x.

Zervaki, D., K. Papanastasi, and E. Maloupa. 2009. 'A New Theory - Model Strategy for New Flower Crops Development'. Acta Horticulturae, no. 813 (March): 147–54. https://doi.org/10.17660/ActaHortic.2009.813.19.

Αναγνωστοπούλου, Μ., Μ. Σεφερλής, Β. Τσιαούση, Ε. Μάντζιου, and Σ Σπύρου. 2015. Κείμενα Εκθεμάτων Του Κέντρου Πληροφόρησης Επισκεπτών Εθνικού Δρυμού Ολύμπου-Μουσείο Ολύμπου Στην Αγγλική Γλώσσα. Natural History Museum Goulandri. http://repository.biodiversity-info.gr/bitstream/11340/1933/1/1680.pdf.

Σαμαροπούλου, Σοφία. 2014. 'Κυτταρολογική μελέτη ενδημικών και σπάνιων φυτών στην Κεφαλονιά'. Master Thesis, University of Patras. http://hdl.handle.net/10889/8163.

Telephium

Vogiatzakis, I. N., G. H. Griffiths, and A. M. Mannion. 2003. 'Environmental Factors and Vegetation Composition, Lefka Ori Massif, Crete, S. Aegean'. *Global Ecology and Biogeography* 12 (2): 131–46. https://doi.org/10.1046/j.1466-822X.2003.00021.x.

Chenopodiaceae

Atriplex

Raus, Thomas, Elpida Karadimou, and Panayotis Dimopoulos. 2019. 'Taxonomic and Functional Plant Diversity of the Santorini-Christiana Island Group (Aegean Sea, Greece)'. *Willdenowia* 49 (3): 363–81.

Beta

Franzén, R., et L.-Å. Gustavsson. 1983. « Chromosome Numbers in Flowering Plants from the High Mountains of Sterea Ellas, Greece ». *Willdenowia* 13 (1): 101-6.

Maxted, Nigel, M. Ehsan Dulloo, Brian V. Ford-Lloyd, Lothar Frese, José Iriondo, et Pinheiro de Carvalho A.A. 2011. *Agrobiodiversity Conservation Securing the Diversity of Crop Wild Relatives and Landraces*. CABI.

Smookler, M. M. 1971. « Properties of Inhibitors of Plant Virus Infection Occurring in the Leaves of Species in the Chenopodiales ». *Annals of Applied Biology* 69 (2): 157-68. https://doi.org/10.1111/j.1744-7348.1971.tb04668.x.

Caroxylon

Bazos, Ioannis, Ioannis P. Kokkoris, et Panayotis Dimopoulos. 2021. « Diversity of Halophytes and Salt Tolerant Plants at the Species-, Habitats- and High-Rank Syntaxa Level in Greece ». In *Handbook of Halophytes*, édité par Marius-Nicusor Grigore, 787-820. Cham: Springer International Publishing. https://doi.org/10.1007/978-3-030-57635-6_26.

Sukhorukov, Alexander P., Alina V. Fedorova, Maria Kushunina, and Evgeny V. Mavrodiev. 2022. 'Akhania, a New Genus for Salsola Daghestanica, Caroxylon Canescens and C. Carpathum (Salsoloideae, Chenopodiaceae, Amaranthaceae)'. *PhytoKeys* 211: 45–61.

Cistaceae

Halimium

Demetzos, Costas, Dimitrios Perdetzoglou, et Kit Tan. 2001. « Chemical Analysis and Antimicrobial Activity of Halimium Voldii ». *Zeitschrift Für Naturforschung C* 56 (11-12): 979-82. https://doi.org/10.1515/znc-2001-11-1212.

Helianthemum

Delitheos, A., E. Tiligada, A. Yannitsaros, et I. Bazos. 1997. « Antiphage Activity in Extracts of Plants Growing in Greece ». *Phytomedicine* 4 (2): 117-24. https://doi.org/10.1016/S0944-7113(97)80055-4.

Colchicaceae

Colchicum

Alexiou, Sotiris. 2013. 'The Genus Colchicum L. (Colchicaceae) in Greece'. *Parnassia Archives* 1: 59–73.

Giannopoulos, Konstantinos, and Kit Tan. 2021. 'Contributions to the Bulb Flora of Ilias (NW Peloponnese, Greece): Asphodelaceae, Colchicaceae, Fumariaceae and Geraniaceae'. *Phytologia Balcanica* 27 (2): 231–37.

Goula, Katerina, and Giorgos Konsoulas. 2016. 'Colchicum Chimonanthum (Colchicaceae): Confirmation of Old Reports and Discovery of New Populations'. *Parnassiana Archives* 4: 23–26.

Goulimis, C. 1958. *Report on Species of Plants Requiring Protection in Greece and Measures for Securing Their Protection*. Athens. http://documents.irevues.inist.fr/bitstream/handle/2042/59422/LATERREETLAVIE_1959_Sup_168.pdf?sequence=1.

Krigas, Nikos, Viktoria Menteli, et Despoina Vokou. 2014. « The Electronic Trade in Greek Endemic Plants: Biodiversity, Commercial and Legal Aspects ». *Economic Botany* 68 (1): 85-95. https://doi.org/10.1007/s12231-014-9264-9.

Marhold, Karol. 2009. « IAPT/IOPB Chromosome Data 7 ». *Taxon* 58 (1): 181-83.

Menteli, Viktoria, Nikos Krigas, Manolis Avramakis, Nicholas Turland, and Despoina Vokou. 2019. 'Endemic Plants of Crete in Electronic Trade and Wildlife Tourism: Current Patterns and Implications for Conservation'. *Journal of Biological Research-Thessaloniki* 26 (1): 10. https://doi.org/10.1186/s40709-019-0104-z.

Negbi, Moshe. 1989. 'Theophrastus on Geophytes'. *Botanical Journal of the Linnean Society* 100 (1): 15–43. https://doi.org/10.1111/j.1095-8339.1989.tb01708.x.

Persson, Karin. 1988. « New Species of Colchicum (Colchicaceae) from the Greek Mountains ». *Willdenowia* 18 (1): 29-46.

Persson, Karin. 1999. 'New and Revised Species ofColchicum (Colchicaceae) from the Balkan Peninsula'. *Plant Systematics and Evolution* 217 (1–2): 55–80. https://doi.org/10.1007/BF00984922.

Petaloudi, Lydia-Maria, Petros Ganatsas, and Marianthi Tsakaldimi. 2022. 'Exploring Biodiversity and Disturbances in the of Peri-Urban Forests of Thessaloniki, Greece'. *Sustainability* 14 (14): 14. https://doi.org/10.3390/su14148497.

Polymenakos, Kostas, and Kit Tan. 2020. *Floristic Notes for the Plateau of Dervenochoria and Mts Parnitha, Pastra and Kitheronas (Nomos Viotias, Sterea Ellas, Central Greece)*.

Poulakou-Rebelakou, E., C. Tsiamis, G. Panteleakos, and S.G. Marketos. 2010. 'Pharmacology in Byzantine Dental Practice'. *Asklepios, International Annual for History and Philosophy of Medicine* 1: 24–27.

Tsiftsis, Spyros, Thomas Giannakis, Sampson Panajiotidis, Eleni Eleftheriadou, and Konstantinos Theodoropoulos. 2021. 'Colchicum Tulakii (Colchicaceae), a New Species from Central Macedonia, Northeastern Greece'. *Nordic Journal of Botany* 39 (6). https://doi.org/10.1111/njb.03207.

Convolvulaceae

Convolvulus

De Montmollin, Bertrand, and Wendy Strahm. 2005. *The Top 50 Mediterranean Island Plants: Wild Plants at the Brink of Extinction, and What Is Needed to Save Them*. IUCN.

Kougioumoutzis, Konstantinos, Panayiota Kotsakiozi, Efthalia Stathi, Panayiotis Trigas, and Aristeidis Parmakelis. 2021. 'Conservation Genetics of Four Critically Endangered Greek Endemic Plants: A Preliminary Assessment'. *Diversity* 13 (4): 4. https://doi.org/10.3390/d13040152.

Krigas, Nikos, Georgios Tsoktouridis, Ioannis Anestis, et al. 2021. 'Exploring the Potential of Neglected Local Endemic Plants of Three Mediterranean Regions in the Ornamental Sector: Value Chain Feasibility and Readiness Timescale for Their Sustainable Exploitation'. *Sustainability* 13 (5): 5. https://doi.org/10.3390/su13052539.

Turland, Nicholas. 1992. 'Floristic Notes from Crete'. *Botanical Journal of the Linnean Society* 108 (4): 345–57. https://doi.org/10.1111/j.1095-8339.1992.tb00250.x.

Cuscuta

Krigas, Nikos, Georgios Tsoktouridis, Ioannis Anestis, et al. 2021. 'Exploring the Potential of Neglected Local Endemic Plants of Three Mediterranean Regions in the Ornamental Sector: Value Chain Feasibility and Readiness Timescale for Their Sustainable Exploitation'. *Sustainability* 13 (5): 5. https://doi.org/10.3390/su13052539.

Crassulaceae

Prometheum

Hart, Henk 't, Roeland D. H. J. van Ham, Jan F. Stevens, Elizabeth T. Elema, Herman van der Klis, and Theo W. J. Gadella. 1999. 'Biosystematic, Molecular and Phytochemical Evidence for the Multiple Origin of Sympetaly in Eurasian Sedoideae (Crassulaceae)'. *Biochemical Systematics and Ecology* 27 (4): 407–26. https://doi.org/10.1016/S0305-1978(98)00098-2.

Sedum

Agricultural University of Athens. 2019. Life Andros Park, 'Conservation of Priority Species and Habitats of Andros Island Protected Area Integrating Socioeconomic Considerations'. Action A.1 - Final Report on the Plant Communities and Their Seasonal and Spatial Variation of the Target Habitat Including the Results of the Base Study. Agricultural University of Athens. http://www.life-androspark.gr/en/wp-content/uploads/2023/01/A1_AUA-Final-Report-on-Vegetation.pdf.

Hart, H. 't. 1984. 'Sedum Apoleipon, a New Species of the Sedum Acre Group (Crassulaceae) from Central Greece'. Willdenowia 13 (2): 309–19.

Hart, H. 't. 1985. « Chromosome Numbers in Sedum (Crassulaceae) from Greece ». *Willdenowia* 15 (1): 115-35.

Iatrou, Gregoris, Panayiotis Trigas, et Nicolaos Pettas. 2007. « The Vascular Flora of Akrokorinthos Castle and Its Surrounding Area (NE Peloponnese, Greece) », 12.

Krigas, Nikos, Viktoria Menteli, and Despoina Vokou. 2014. 'The Electronic Trade in Greek Endemic Plants: Biodiversity, Commercial and Legal Aspects'. Economic Botany 68 (1): 85–95. https://doi.org/10.1007/s12231-014-9264-9.

Snogerup, Sven, Britt Snogerup, Elli Stamatiadou, Roland von Bothmer, and Mats Gustafsson. 2006. 'Flora and Vegetation of Andros, Kikladhes, Greece'. Ann. Musei Goulandris 11: 85–270.

Trigas, Panayiotis, Eleftherios Kalpoutzakis, Epaminondas Kalogiannis, et al. 2021. 'Noteworthy New Floristic Records from Greece'. Botanica Serbica 45 (2): 321–31. https://doi.org/10.2298/BOTSERB2102321T.

Umbilicus

Carlström, Annette. 1984. 'New Species of Alyssum, Consolida, Origanum and Umbilicus from the SE Aegean Sea'. *Willdenowia* 14 (1): 15–26.

Cattaneo, Cristina, and Mauro Grano. 2015. 'New Contribution on the Vascular Flora of the Aegean Island of Chalki (Archipelago of Rhodes, Aegean Sea)'. *Biodiversity Journal* 6 (4): 773–88.

Tsakiri, Maria, Konstantinos Kougioumoutzis, and Gregoris Iatrou. 2016. 'Contribution to the Vascular Flora of Chalki Island (East Aegean, Greece) and Biomonitoring of a Local Endemic Taxon'. *Willdenowia* 46 (1): 175–90. https://doi.org/10.3372/wi.46.46114.

Cyperaceae

Carex

Bergmeier, Erwin, and Stefan Abrahamczyk. 2007. 'Ecology and Distributon of the Aegean Wetland Endemics Carex Cretica and Lathyrus Neurolobus'. *Nova Hedwigia* 131: 207–19.

Escudero, Marcial, Virginia Valcárcel, Pablo Vargas, and Modesto Luceño. 2008. 'Evolution in Carex L. Sect. Spirostachyae (Cyperaceae): A Molecular and Cytogenetic Approach'. *Organisms Diversity & Evolution* 7 (4): 271–91. https://doi.org/10.1016/j.ode.2006.08.006.

Gradstein, S. R., and J. H. Kern. 1968. 'A New Carex from Crete'. *Acta Botanica Neerlandica* 17 (4): 242–47.

Greuter, Werner, Ursula Matthäs, and Horst Risse. 1985. 'Additions to the Flora of Crete, 1973-1983 (1984) - III'. *Willdenowia* 15 (1): 23–60.

Dipsacaceae

Cephalaria

Cattaneo, Cristina, and Mauro Grano. 2015. 'Considerationi preliminari sull'aspetto vegetale delle isole di Chalki e Alimia (Arcipelago di Rodi, Egeo Se)'. *Ann. Mus. civ. Rovereto* 30: 369–99.

Constantinidis, Theophanis, Georgia Kamari, et Dimitrios Phitos. 1997. « A Cytological Study of 28 Phanerogams from the Mountains of SE Sterea Ellas, Greece ». *Willdenowia* 27 (1/2): 121-42.

Constantinidis, Theophanidis, Eleftheria-Perdiko Bareka, and Georgia Kamari. 2002. 'Karyotaxonomy of Greek Serpentine Angiosperms'. *Botanical Journal of the Linnean Society* 139 (1): 109–24. https://doi.org/10.1046/j.1095-8339.2002.00044.x.

Dimitrellos, Georgios, and Dimitrios Christodoulakis. 1999. 'The Phytogeographical Distribution Patterns of the Flora of Mt Timfristos (N.W. Sterea Ellas, Greece)'. *Flora Mediterranea* 9: 215–29.

Strid, Arne. 1981. 'New Species of Cephalaria (Dipsacaceae) and Stipa (Gramineae) from the Greek Mountains'. *Willdenowia* 11 (2): 301–5.

Lomeliosa

Bergmeier, Erwin. 2011. 'New Floristic Records, Confirmations and Other Phytogeographical Notes from Crete (Greece)'. *Willdenowia* 41 (1): 167–77.

Cattaneo, Cristina, and Mauro Grano. 2018. 'Contribution to the Flora of Tilos Island (Dodecanese Islands, Greece)'. *Parnassiana Archives* 6: 41–53.

Grigoriadou, Katerina, Nikos Krigas, Virginia Sarropoulou, Eleni Maloupa, and Georgios Tsoktouridis. 2021. 'Propagation and Ex-Situ Conservation of Lomelosia Minoana Subsp. Minoana and Scutellaria Hirta - Two Ornamental and Medicinal Cretan Endemics (Greece)'. *Notulae Botanicae Horti Agrobotanici Cluj-Napoca* 49 (1): 1. https://doi.org/10.15835/nbha49112168.

Grigoriadou, K., N. Krigas, V. Sarropoulou, K. Papanastasi, G. Tsoktouridis, et E. Maloupa. 2019. « In Vitro Propagation of Medicinal and Aromatic Plants: The Case of Selected Greek Species with Conservation Priority ». *In Vitro Cellular & Developmental Biology - Plant* 55 (6): 635-46. https://doi.org/10.1007/s11627-019-10014-6.

Hatzilazarou, Stefanos, Stefanos Kostas, Elias Pipinis, et al. 2023. 'GIS-Facilitated Seed Germination, Fertilization Effects on Growth, Nutrient and Phenol Contents and Antioxidant Potential in Three Local Endemic Plants of Crete (Greece) with Economic Interest: Implications for Conservation and Sustainable Exploitation'. *Horticulturae* 9 (3): 3. https://doi.org/10.3390/horticulturae9030335.

Krigas, Nikos, Georgios Tsoktouridis, Ioannis Anestis, et al. 2021. ‘Exploring the Potential of Neglected Local Endemic Plants of Three Mediterranean Regions in the Ornamental Sector: Value Chain Feasibility and Readiness Timescale for Their Sustainable Exploitation’. *Sustainability* 13 (5): 5. https://doi.org/10.3390/su13052539.

Sarropoulou, Virginia, Nikos Krigas, Georgios Tsoktouridis, Eleni Maloupa, and Katerina Grigoriadou. 2022. ‘Seed Germination Trials and Ex Situ Conservation of Local Prioritized Endemic Plants of Crete (Greece) with Commercial Interest’. *Seeds* 1 (4): 4. https://doi.org/10.3390/seeds1040024.

Turland, Nicholas. 1992. ‘Floristic Notes from Crete’. *Botanical Journal of the Linnean Society* 108 (4): 345–57. https://doi.org/10.1111/j.1095-8339.1992.tb00250.x.

Λυσίκατος, Χαράλαμπος. 2021. « Διερεύνηση της επίδρασης του είδους υποστρώματος και της συχνότητας άρδευσης στην ανάπτυξη των ξηροφυτικών ειδών Globularia alypum και Lomelosia hymettia σε συνθήκες φυτοδώματος - Investigation of the effect of the substrate type and the frequency of irrigation on the growth of the dry plant species Globularia alypum and Lomelosia hymettia under phytonutrient conditions », mars. http://dspace.aua.gr/xmlui/handle/10329/7253.

Pterocephalus

Graikou, Konstantia, Nektarios Aligiannis, et Ioanna B. Chinou. 2006. « Chemical Constituents from Pterocephalus Perennis Subsp. Perennis (Dipsacaceae) ». *Biochemical Systematics and Ecology* 34 (5): 438-41. https://doi.org/10.1016/j.bse.2005.12.008.

Graikou, Konstantia, Nektarios Aligiannis, Ioanna B. Chinou, et Catherine Harvala. 2002. « Cantleyoside-Dimethyl-Acetal and Other Iridoid Glucosides from Pterocephalus Perennis – Antimicrobial Activities ». *Zeitschrift Für Naturforschung C* 57 (1-2): 95-99. https://doi.org/10.1515/znc-2002-1-217.

Gülcemal, Derya, Milena Masullo, Ozgen Alankuş-Çalışkan, Tamer Karayıldırım, Serdar G. Şenol, Sonia Piacente, et Erdal Bedir. 2010. « Monoterpenoid Glucoindole Alkaloids and Iridoids from Pterocephalus Pinardii ». *Magnetic Resonance in Chemistry* 48 (3): 239-43. https://doi.org/10.1002/mrc.2559.

Euphorbiaceae

Euphorbia

Andrić, Tihana. 2017. ‘Sakupljanje Novog Sadnog Materijala u Botaničkom Vrtu u Chania, Kreta’. Master Thesis, Josip Juraj Strossmayer University of Osijek. Faculty of agriculture …. https://repozitorij.unios.hr/en/islandora/object/pfos%3A1345.

Krigas, Nikos, Georgios Tsoktouridis, Ioannis Anestis, et al. 2021. ‘Exploring the Potential of Neglected Local Endemic Plants of Three Mediterranean Regions in the Ornamental Sector: Value Chain Feasibility and Readiness Timescale for Their Sustainable Exploitation’. *Sustainability* 13 (5): 5. https://doi.org/10.3390/su13052539.

Nieto Feliner, Gonzalo, Nico Cellinese, Andrew A. Crowl, and Božo Frajman. 2023. ‘Editorial: Understanding Plant Diversity and Evolution in the Mediterranean Basin’. *Frontiers in Plant Science* 14. https://www.frontiersin.org/articles/10.3389/fpls.2023.1152340.

Spanos, Ioannis, Panagiotis Platis, Ioannis Meliadis, and Alexandros Tsiontis. 2008. ‘A Review on the Ecology and Management of the Samaria Gorge, a Greek Biosphere Reserve’. *Journal of Geography and Regional Planning* 1 (2): 19–33.

Strid, Arne, Bengt Bentzer, Roland Von Bothmer, Lennart Engstrand, and Mats Gustafsson. 1989. ‘Euphorbia Sultan-Hassei (Euphorbiaceae), a New Species from SW Crete’. *Willdenowia* 19 (1): 63–67.

Fabaceae

Anthyllis

Grigoriadou, K., N. Krigas, V. Sarropoulou, K. Papanastasi, G. Tsoktouridis, and E. Maloupa. 2019. ‘In Vitro Propagation of Medicinal and Aromatic Plants: The Case of Selected Greek Species with Conservation Priority’. *In Vitro Cellular & Developmental Biology - Plant* 55 (6): 635–46. https://doi.org/10.1007/s11627-019-10014-6.

Kalpoutzakis, Eleftherios, Theophanis Constantinidis, and Panayiotis Trigas. 2019. *Chorological Additions for Some Noteworthy Taxa of the Greek Flora*. 18.

Papafotiou, M., A.N. Martini, and G. Vlachou. 2017. ‘In Vitro Propagation as a Tool to Enhance the Use of Native Ornamentals in Archaeological Sites of Greece’. *Acta Horticulturae*, no. 1155 (March): 301–8. https://doi.org/10.17660/ActaHortic.2017.1155.43.

Saxena, Akansha, Wen-Lu Bi, Mukund R. Shukla, Syd Cannings, Bruce Bennett, and Praveen K. Saxena. 2021. ‘Micropropagation and Cryopreservation of Yukon Draba (Draba Yukonensis), a Special Concern Plant Species Endemic to Yukon Territory, Canada’. *Plants* 10 (10): 10. https://doi.org/10.3390/plants10102093.

Astragalus

Ghaffari, Muhammad Abuzar, Bashir Ahmad Chaudhry, Muhammad Uzair, Muhammad Imran, and Khuram Ashfaq. 2021. ‘Total Phenolic and Flavonoid Contents, Cytotoxic, Immunomodulatory and Anti-Inflammatory Potential of Whole Plant of Astragalus Creticus (Fabaceae)’. *Tropical Journal of Pharmaceutical Research* 20 (10): 10. https://doi.org/10.4314/tjpr.v20i10.14.

Ghaffari, Muhammad Abuzar, Bashir Ahmad Chaudhry, Muhammad Uzair, Muhammad Imran, Muhammad Haneef, and Khuram Ashfaq. 2021. ‘Biological and Phytochemical Investigations of Crude Extracts of Astragalus Creticus’. *Pakistan Journal of Pharmaceutical Sciences* 34 (1): 403–9.

Krigas, N., V. Menteli, and D. Vokou. 2016. ‘Analysis of the Ex Situ Conservation of the Greek Endemic Flora at National European and Global Scales and of Its Effectiveness in Meeting GSPC Target 8’. *Plant Biosystems - An International Journal Dealing with All Aspects of Plant Biology* 150 (3): 573–82. https://doi.org/10.1080/11263504.2014.988194.

Krigas, Nikos, Marina Panagiotidou, and Eleni Maloupa. 2017. ‘Incorporating Biogeographical Principles in Horticulture: Design

and Creation of the Ionian Islands Unique Rock Garden in Thessaloniki, Greece'. *Sibbaldia: The Journal of Botanic Garden Horticulture* 0 (15): 129–46.

Krigas, Nikos, Georgios Tsoktouridis, Ioannis Anestis, et al. 2021. 'Exploring the Potential of Neglected Local Endemic Plants of Three Mediterranean Regions in the Ornamental Sector: Value Chain Feasibility and Readiness Timescale for Their Sustainable Exploitation'. *Sustainability* 13 (5): 5. https://doi.org/10.3390/su13052539.

Lafranchis, Tristan. 2019. « Notes on the biology of some butterflies in Greece (Lepidoptera: Papilionoidea) ». *Entomologist's Gazette* 70 (2): 113-134(22).Löve, Áskell. 1971. 'IOPB Chromosome Number Reports XXXIV'. *TAXON* 20 (5–6): 785–97. https://doi.org/10.1002/j.1996-8175.1971.tb03208.x.

Löve, Áskell. 1977. « IOPB Chromosome Number Reports LVII ». *Taxon* 26 (4): 443-52.

Maloupa, E, N Krigas, and A Karydas. 2007. *The in Situ Plant Conservation Actions of the Balkan Botanic Garden of Kroussia in Greece*. 5.

Myrtsi, Eleni D., Epameinondas Evergetis, Sofia D. Koulocheri, and Serkos A. Haroutounian. 2023. 'Bioactivity of Wild and Cultivated Legumes: Phytochemical Content and Antioxidant Properties'. *Antioxidants* 12 (4): 4. https://doi.org/10.3390/antiox12040852.

Persson, J., et A. Strid. 1982. « A New Species of Astragalus (Fabaceae) from Mt Taygetos ». *Willdenowia* 12 (2): 207-9.

Podlech, D. 2008. 'The Genus Astragalus L. (Fabaceae) in Europe with Exclusion of the Former Soviet Union'. *Feddes Repertorium* 119 (5–6): 310–87. https://doi.org/10.1002/fedr.200811171.

Sabir, Muhammad, Edita Baltrėnaitė-Gedienė, Allah Ditta, et al. 2022. 'Bioaccumulation of Heavy Metals in a Soil–Plant System from an Open Dumpsite and the Associated Health Risks through Multiple Routes'. *Sustainability* 14 (20): 20. https://doi.org/10.3390/su142013223.

Strid, Arne. 2007. 'Lost and Found in the Greek Flora'. 16–20.

Ullah, Manzoor, Muhammad Usman Khan, Adeel Mahmood, et al. 2013. 'An Ethnobotanical Survey of Indigenous Medicinal Plants in Wana District South Waziristan Agency, Pakistan'. *Journal of Ethnopharmacology* 150 (3): 918–24. https://doi.org/10.1016/j.jep.2013.09.032.

Žikić, Vladimir, Saša S Stanković, Boženka Hric, Mircea-Dan Mitroiu, Martin Schwartz, et Hans-Peter Tschorsnig. 2015. « First Records of Parasitoids of the Orders Hymenoptera (Ichneumonidae and Pteromalidae) and Diptera (Tachinidae) from Heterogynis Sondereggeri de Freina, 2012 (Lepidoptera: Heterogynidae) », 5.

Cicer

Stathi, Efthalia, Konstantinos Kougioumoutzis, Eleni M. Abraham, Panayiotis Trigas, Ioannis Ganopoulos, Evangelia V. Avramidou, et Eleni Tani. 2020. « Population genetic variability and distribution of the endangered Greek endemic Cicer graecum under climate change scenarios | AoB PLANTS | Oxford Academic ». *AoB Plants* 12 (2). https://doi.org/10.1093/aobpla/plaa007.

Ebenus

Duan, Lei, Jun Wen, Xue Yang, Pei-Liang Liu, Emine Arslan, Kuddisi Ertuğrul, et Zhao-Yang Chang. 2015. « Phylogeny of Hedysarum and Tribe Hedysareae (Leguminosae: Papilionoideae) Inferred from Sequence Data of ITS, MatK, TrnL-F and PsbA-TrnH ». *TAXON* 64 (1): 49-64. https://doi.org/10.12705/641.26.

Kounadi, S., N. Aligiannis, I. Pongratz, P. Lelovas, D. Ismini, and A. L. Skaltsounis. 2011. 'Estrogenic Activity of the Methanolic Extract of Ebenus Cretica L'. Planta Medica 77 (12): PM168. https://doi.org/10.1055/s-0031-1282926.

Krigas, Nikos, Georgios Tsoktouridis, Ioannis Anestis, et al. 2021. 'Exploring the Potential of Neglected Local Endemic Plants of Three Mediterranean Regions in the Ornamental Sector: Value Chain Feasibility and Readiness Timescale for Their Sustainable Exploitation'. Sustainability 13 (5): 5. https://doi.org/10.3390/su13052539.

Mitrocotsa, Dimitra, Alexios-Leandros Skaltsounis, Sofia Mitaku, Catherine Harvala, et Francois Tillequin. 1999. « Flavonoid and Terpene Glycosides from European Ebenus Species ». *Biochemical Systematics and Ecology* 27 (3): 305-7. https://doi.org/10.1016/S0305-1978(98)90077-1.

Nektarios, P.A., E. Nydrioti, T. Kapsali, and N. Ntoulas. 2016. 'Substrate Type, Depth and Irrigation Regime Effects on Ebenus Cretica Growth in Extensive Green Roof'. Acta Horticulturae, no. 1108 (February): 297–302. https://doi.org/10.17660/ActaHortic.2016.1108.39.

Obón, Concepción, Diego Rivera, Elena Fonollá, Francisco Alcaraz, et Latifa Attieh. 2021. « A Comparison Study on Traditional Mixtures of Herbal Teas Used in Eastern Mediterranean Area ». *Frontiers in Pharmacology* 12 (avril): 632692. https://doi.org/10.3389/fphar.2021.632692.

Syros, T., G. Kofidis, A. S. Economou, and A. M. Bosabalidis. 2006. 'Leaf Structural Dynamics Associated with Adaptation of Two Ebenus Cretica Ecotypes'. Biologia Plantarum 50 (2): 245–50. https://doi.org/10.1007/s10535-006-0014-4.

Syros, Thomas, Traianos Yupsanis, Helias Zafiriadis, and Athanasios Economou. 2004. 'Activity and Isoforms of Peroxidases, Lignin and Anatomy, during Adventitious Rooting in Cuttings of Ebenus Cretica L.' Journal of Plant Physiology 161 (1): 69–77. https://doi.org/10.1078/0176-1617-00938.

Vlahos, J. C., and M. Dragassaki. 1995. 'Propagation of Cretan Silver Bush (Ebenus Cretica L.), A Potential New Flower Crop'. HortScience. HortScience 30 (4): 851G – 851. https://doi.org/10.21273/HORTSCI.30.4.851G.

Genista

Fokialakis, N., N. Aligiannis, X. Alexi, M. N. Alexis, H. Pratsinis, E. Kalpoutzakis, et S. Miatkou. 2012. « Phytoestrogens from Genista Halacsyi (Leguminosae) ». *Planta Medica* 78 (11): PI361. https://doi.org/10.1055/s-0032-1321048.

Fokialakis, N., N. Aligiannis, X. Alexi, M. N. Alexis, H. Pratsinis, E. Kalpoutzakis, et A. L. Skaltsounis. 2008. « Isolation of Bioactive Compounds from Genista Halacsyi (Leguminosae) and Evaluation of Their Estrogenic Activity ». *Planta Medica* 74 (9): PB165. https://doi.org/10.1055/s-0028-1084509.

Fokialakis, Nikolas, Xanthippi Alexi, Nektarios Aligiannis, Athina Boulaka, Aggeliki K. Meligova, George Lambrinidis, Eleftherios Kalpoutzakis, et al. 2019. « Biological Evaluation of Isoflavonoids from Genista Halacsyi Using Estrogen-Target Cells: Activities of Glucosides Compared to Aglycones ». *PLOS ONE* 14 (1): e0210247. https://doi.org/10.1371/journal.pone.0210247.

Musarella, Carmelo Maria, Salvatore Brullo, and Gianpietro Giusso Del Galdo. 2020. 'Contribution to the Orophilous Cushion-like Vegetation of Central-Southern and Insular Greece'. *Plants* 9 (12): 1678.

Tiziana, Cusma Velari, Laura Feoli Chiapella, et Vera Kosovel. 2009. « Karyomorphology and Systematics of the Eastern Taxa of Genista Sect. Spartioides and G. Pulchella (Genisteae-Fabaceae). » *Caryologia* 62 (2): 102-13. https://doi.org/10.1080/00087114.2004.10589675.

Lathyrus

Bergmeier, Erwin, and Stefan Abrahamczyk. 2007. 'Ecology and Distributon of the Aegean Wetland Endemics Carex Cretica and Lathyrus Neurolobus'. Nova Hedwigia 131: 207–19.

Kenicer, Gregory, and Sylvia Norton. 2008. '631. LATHYRUS NEUROLOBUS: Leguminosae'. Curtis's Botanical Magazine 25 (4): 310–16.

Vaz Patto, M. C., and D. Rubiales. 2014. 'Lathyrus Diversity: Available Resources with Relevance to Crop Improvement – L. Sativus and L. Cicera as Case Studies'. Annals of Botany 113 (6): 895–908. https://doi.org/10.1093/aob/mcu024.

Medicago

Eriksson, Jonna S., Filipe de Sousa, Yann J. K. Bertrand, Alexandre Antonelli, Bengt Oxelman, and Bernard E. Pfeil. 2018. 'Allele Phasing Is Critical to Revealing a Shared Allopolyploid Origin of Medicago Arborea and M. Strasseri (Fabaceae)'. *BMC Evolutionary Biology* 18 (1): 9. https://doi.org/10.1186/s12862-018-1127-z.

González-Andrés, Fernando, and José-Luis Ceresuela. 1998. 'Chemical Composition of Some Iberian Mediterranean Leguminous Shrubs Potentially Useful for Forage in Seasonally Dry Areas'. *New Zealand Journal of Agricultural Research* 41 (2): 139–47. https://doi.org/10.1080/00288233.1998.9513297.

Greuter, W., U. Matthäs, and H. Risse. 1982. 'Notes on Cardaegean Plants. 3. Medicago Strasseri, a New Leguminous Shrub from Kriti'. *Willdenowia* 12 (2): 201–6.

Lefi, E, J Gulías, M Ribas-Carbo, and H Medrano. 2004. 'Soil Water Deficit Effects on Photosynthesis, Water Use Efficiency and Growth of Three Mediterranean Shrubs: Medicago Arborea, Medicago Citrina and Medicago Strasseri'. *Cahier Options Méditerranéennes*, Réhabilitation des pâturages et des parcours en milieux méditerranéens, vol. 62: 65–68.

Perez-Garcia, Felix, Jose L. Ceresuela, Aldo E. Gonzalez, and Itziar Aguinagalde. 1992. 'Flavonoids in Seed Coats of Medicago Arborea and M. Strasseri (Leguminosae): Ecophysiological Aspects'. *Journal of Basic Microbiology* 32 (4): 241–48. https://doi.org/10.1002/jobm.3620320406.

Melilotus

Abou-El-Enain, Maged Mahmoud. 2014. « Reassessment of the Taxonomic Relationships between Closely Related Taxa of Papilionoideae ». *Pure and Applied Biology* 3 (1): 32-54. https://doi.org/10.19045/bspab.2014.31005.

Delitheos, A., E. Tiligada, A. Yannitsaros, et I. Bazos. 1997. « Antiphage Activity in Extracts of Plants Growing in Greece ». *Phytomedicine* 4 (2): 117-24. https://doi.org/10.1016/S0944-7113(97)80055-4.

Ρίτσος, Βασίλειος. 2015. « Σε αναζήτηση συμβολής του υποδοχέα οιστρογόνων στη νευροπροστατευτική δράση ενώσεων φυτικής προέλευσης - Research on estrogen receptor contribution to the neuroprotective activity of compounds of plant origin ». Larisa: ΠΑΝΕΠΙΣΤΗΜΙΟ ΘΕΣΣΑΛΙΑΣ ΤΜΗΜΑ ΒΙΟΧΗΜΕΙΑΣ & ΒΙΟΤΕΧΝΟΛΟΓΙΑΣ ΣΧΟΛΗ ΕΠΙΣΤΗΜΩΝ ΥΓΕΙΑΣ. https://ir.lib.uth.gr/xmlui/bitstream/handle/11615/46749/14107.pdf?sequence=1.

Onobrychis

Dontas, I., M. Halabalaki, P. Moutsatsou, S. Mitakou, Z. Papoutsi, L. Khaldi, A. Galanos, et G. P. Lyritis. 2006. « Protective Effect of Plant Extract from Onobrychis Ebenoides on Ovariectomy-Induced Bone Loss in Rats ». *Maturitas* 53 (2): 234-42. https://doi.org/10.1016/j.maturitas.2005.05.007.

Greuter, Werner. 1987. 'Onobrychis Aliacmonia (Leguminosae) — the Unusual Story of a Rediscovery'. *Plant Systematics and Evolution* 155 (1): 215–17. https://doi.org/10.1007/BF00936300.

Greuter, Werner, and Thomas Raus. 1999. 'Med-Checklist Notulae, 18'. *Willdenowia* 29 (1/2): 51–67. https://doi.org/10.3372/wi.29.2905.

Halabalaki, M., I. T. Roumeliotis, E. Giannopoulou, X. Alexi, L. Meijer, M. N. Alexis, A. L. Skaltsounis, et D. S. Garbis. 2008. « Pharmacoproteomic and Toxicoproteomic Study of the Natural Product Ebenfuran III in DU-145 Prostate Cancer Cells Using ITRAQ with 2D LC and Tandem Mass Spectrometry ». *Planta Medica* 74 (9): SL40. https://doi.org/10.1055/s-0028-1083920.

Halabalaki, Maria, Xanthippi Alexi, Necktarios Aligiannis, Michael N. Alexis, et Alexios-Leandros Skaltsounis. 2008. « Ebenfurans IV–VIII from Onobrychis ebenoides: Evidence that C-Prenylation is the Key Determinant of the Cytotoxicity of 3-Formyl-2-arylbenzofurans ». *Journal of Natural Products* 71 (11): 1934-37. https://doi.org/10.1021/np800134h.

Halabalaki, Maria, Xanthippi Alexi, Nektarios Aligiannis, George Lambrinidis, Harris Pratsinis, Ida Florentin, Sofia Mitakou, Emmanuel Mikros, Alexios-Leandros Skaltsounis, et Michael N. Alexis. 2006. « Estrogenic Activity of Isoflavonoids from Onobrychis Ebenoides ». *Planta Medica* 72 (6): 488-93. https://doi.org/10.1055/s-2005-916261.

Halabalaki, Maria, Nektarios Aligiannis, Zoi Papoutsi, Sofia Mitakou, Paraskevi Moutsatsou, Constantine Sekeris, et Alexios-Leandros Skaltsounis. 2000. « Three New Arylobenzofurans from Onobrychis ebenoides and Evaluation of Their Binding Affinity for the Estrogen Receptor ». *Journal of Natural Products* 63 (12): 1672-74. https://doi.org/10.1021/np000071b.

Katsanou, Efrosini S., Maria Halabalaki, Nektarios Aligiannis, Sofia Mitakou, Alexios-Leandros Skaltsounis, Xanthippi Alexi, Harris

Pratsinis, et Michael N. Alexis. 2007. « Cytotoxic Effects of 2-Arylbenzofuran Phytoestrogens on Human Cancer Cells: Modulation by Adrenal and Gonadal Steroids ». *The Journal of Steroid Biochemistry and Molecular Biology*, 17th International Symposium of the Journal of Steroid Biochemistry & Molecular Biology 'Recent Advances in Steroid Biochemistry and Molecular Biology' (Seefeld, Tyrol, Austria, 31 May – 03 June 2006)., 104 (3): 228-36. https://doi.org/10.1016/j.jsbmb.2007.03.028.

Lafranchis, Tristan, et Antoine Lafranchis. 2012. « Five Blues on a Flower: Interactions between Polyommatinae Butterflies (Lepidoptera, Lycaenidae), Ants and Parasitoids in the Northern Peloponnese (Greece) », 7.

Papoutsi, Z., E. Kassi, M. Halabalaki, S. Mitakou, et P. Moutsatsou. 2007. « Evaluation of Estrogenic/Antiestrogenic Activity of Onobrychis Ebenoides Extract – Interaction with Estrogen Receptor Subtypes ERα and ERβ ». *Toxicology in Vitro* 21 (3): 364-70. https://doi.org/10.1016/j.tiv.2006.09.009.

Tan, Kit. 2007. 'The Endemic Flora of Greece'. *Building a Sustainable Future: The Role of Botanic Gardens.*, April 16, 5.

Tan, Kit, and G. Iatrou. 1996. 'Onobrychis Aliacmonia Rech.f. – a New Slant on an Old Story'. *Annalen Des Naturhistorischen Museums in Wien. Serie B Für Botanik Und Zoologie* 98: 305–9.

Vladimirov, Compiled Vladimir, Feruzan Dane, and Kit Tan. 2016. 'New Floristic Records in the Balkans: 30'. *Phytologia Balcanica* 22 (2): 259–92.

Σαράτση, Αικατερίνη Αναστασίου. 2016. 'In vitro μελέτη ανθελμινθικής δράσης φυτικών ειδών, έναντι στα γαστρεντερικά παράσιτα των προβάτων'. In *Aristotle University of Thessaloniki Institutional Repository - IKEE*, GRI-2017-18615. Αριστοτέλειο Πανεπιστήμιο Θεσσαλονίκης. https://doi.org/10.26262/heal.auth.ir.287809.

Ononis

Bergmeier, Erwin, Thomas Blockeel, Niels Böhling, et al. 2011. 'An Inventory of the Vascular Plants and Bryophytes of Gavdopoula Island (S Aegean, Greece) and Its Phytogeographical Significance'. *Willdenowia* 41 (1): 179–90. https://doi.org/10.3372/wi.41.41121.

Turland, Nicholas, Dimitrios Phitos, Georgia Kamari, et Pepy Bareka. 2004. « Weeds of the traditional agriculture of Crete ». *Willdenowia* 34 (2): 381-406. https://doi.org/10.3372/wi.34.34206.

Securigera

Bourgou, Soumaya, Imtinen Ben Haj Jilani, Olfa Karous, et al. 2021. 'Medicinal-Cosmetic Potential of the Local Endemic Plants of Crete (Greece), Northern Morocco and Tunisia: Priorities for Conservation and Sustainable Exploitation of Neglected and Underutilized Phytogenetic Resources'. *Biology* 10 (12): 1344. https://doi.org/10.3390/biology10121344.

Trifolium

Böhling, Niels, Werner Greuter, and Thomas Raus. 1998. 'Trifolium Phitosianum (Leguminosae), a New Annual Clover Species from Crete'. *Bot. Chron.* 13.

Greuter, Werner, Regina Pleger, and Thomas Raus. 1983. 'The Vascular Flora of the Karpathos Island Group (Dodecanesos, Greece). A Preliminary Checklist'. *Willdenowia* 13 (1): 43–78.

Lassen, P. 1996. 'Trifolium Andricum (Fabaceae), a New Species from Greece'. *Annalen Des Naturhistorischen Museums in Wien. Serie B Für Botanik Und Zoologie* 98: 301–3.

Löve, Áskell. 1981. « Chromosome Number Reports LXXIII ». *Taxon* 30 (4): 829-61.

Scoppola, A., J. López Tirado, F. Manzano Gutiérrez, and S. Magrini. 2018. 'The Genus *Trifolium* (Fabaceae) in South Europe: A Critical Review on Species Richness and Distribution'. *Nordic Journal of Botany* 36 (1_2): njb-01723. https://doi.org/10.1111/njb.01723.

Trigonella

Bazos, Ioannis, Ioannis P. Kokkoris, et Panayotis Dimopoulos. 2021. « Diversity of Halophytes and Salt Tolerant Plants at the Species-, Habitats- and High-Rank Syntaxa Level in Greece ». In *Handbook of Halophytes*, édité par Marius-Nicusor Grigore, 787-820. Cham: Springer International Publishing. https://doi.org/10.1007/978-3-030-57635-6_26.

Vicia

Constantinidis, Theophanis. 2013. 'The Flora of the Kastellorizo Island Group (East Aegean Islands, Greece): New Records and Comments'. *Flora Mediterranea* 23 (December): 69–86. https://doi.org/10.7320/FlMedit23.069.

Delitheos, A., E. Tiligada, A. Yannitsaros, and I. Bazos. 1997. 'Antiphage Activity in Extracts of Plants Growing in Greece'. *Phytomedicine* 4 (2): 117–24. https://doi.org/10.1016/S0944-7113(97)80055-4.

Fagaceae

Quercus

Grigoriadou, K., N. Krigas, V. Sarropoulou, K. Papanastasi, G. Tsoktouridis, and E. Maloupa. 2019. 'In Vitro Propagation of Medicinal and Aromatic Plants: The Case of Selected Greek Species with Conservation Priority'. *In Vitro Cellular & Developmental Biology - Plant* 55 (6): 635–46. https://doi.org/10.1007/s11627-019-10014-6.

Simeone, Marco Cosimo, Simone Cardoni, Roberta Piredda, et al. 2018. 'Comparative Systematics and Phylogeography of Quercus Section Cerris in Western Eurasia: Inferences from Plastid and Nuclear DNA Variation'. *PeerJ* 6 (October): e5793. https://doi.org/10.7717/peerj.5793.

Δόγανος, Δημήτριος. 2010. 'Η κατάσταση στη φύση και η δημιουργία μόνιμων επιφανειών παρακολούθησης του ενδημικού φυτού Quercus trojana Webb subsp. euboica (Papaioannou) K. I. Chr.' Master Thesis, University of Patras. https://hdl.handle.net/10889/4513.

Fumariaceae

Corydalis

Lidén, Magnus. 1996. « New taxa of tuberous Corydalis (Fumariaceae) ». *Willdenowia* 26 (1/2): 23-35. https://doi.org/10.3372/wi.26.2602.

Snogerup, Sven, Britt Snogerup, Elli Stamatiadou, Roland von Bothmer, and Mats Gustafsson. 2006. 'Flora and Vegetation of Andros, Kikladhes, Greece'. *Ann. Musei Goulandris* 11: 85–270.

Geraniaceae

Erodium

Krigas, Nikos, Viktoria Menteli, et Despoina Vokou. 2014. « The Electronic Trade in Greek Endemic Plants: Biodiversity, Commercial and Legal Aspects ». *Economic Botany* 68 (1): 85-95. https://doi.org/10.1007/s12231-014-9264-9.

Maloupa, Eleni, Nikos Krigas, Katerina Grigoriadou, Diamanto Lazari, and Georgios Tsoktouridis. 2008. *Conservation Strategies for Native Plant Species and Their Sustainable Exploitation: Case of the Balkan Botanic Garden of Kroussia, N. Greece*. 21.

Park, Jong Cheol. 2017. « 파리식물원 982종 약용식물의 조사 연구 - Research Study on 982 Medicinal Plants in Paris Plant Garden ». *Korean Herb. Med. Inf.* http://herba.kr/khmi/j/05(1)/KHMI-05(1)-01.pdf.

Vladimirov, Compiled Vladimir, Feruzan Dane, and Kit Tan. 2016. 'New Floristic Records in the Balkans: 30'. *Phytologia Balcanica* 22 (2): 259–92.

Geranium

Tan, Kit, Sonja Siljak-Yakovlev, et Gert Vold. 2011. « Geranium Kikianum Sp. Nov. (Geraniaceae) from the Southern Peloponnese, Greece ». *Nordic Journal of Botany* 29 (1): 1-5. https://doi.org/10.1111/j.1756-1051.2010.01028.x.

Zeljković, Sanja Ćavar, Kit Tan, Sonja Siljak-Yakovlev, et Milka Maksimović. 2017. « Essential Oil Profile, Phenolic Content and Antioxidant Activity of *Geranium Kikianum* ». *Natural Product Communications* 12 (2): 1934578X1701200. https://doi.org/10.1177/1934578X1701200234.

Gesneriaceae

Jancaea

Djilianov, Dimitar L, Daniela P Moyankova, and Petko V Mladenov. 2016. *The Mediterranean: A Cradle of the Resurrection Plants in Europe*.

Legardón, Ane, and José Ignacio García-Plazaola. 2023. 'Gesneriads, a Source of Resurrection and Double-Tolerant Species: Proposal of New Desiccation- and Freezing-Tolerant Plants and Their Physiological Adaptations'. *Biology* 12 (1): 1. https://doi.org/10.3390/biology12010107.

Stevanovié, Branka, and Olivera Glisié. 1997. 'Eco-Anatomical Differences between Balkan Endemo-Relict Species of Gesneriaceae'. *Bocconea* 5: 661–66.

Τσιτίνης, Γεώργιος Νικολάου. 2014. 'Jancaea heldreichii: Οικολογία και χωρολογία ενός υπολειμματικού, ενδημικού είδους του Ολύμπου'. In *Aristotle University of Thessaloniki Institutional Repository - IKEE*, GRI-2015-13932. Αριστοτέλειο Πανεπιστήμιο Θεσσαλονίκης. https://doi.org/10.26262/heal.auth.ir.135990.

Globulariaceae

Globularia

Bernhardt, Peter. 2008. *Chapter 4. The Triumph of Zeus. Gods and Goddesses in the Garden*. Rutgers University Press. https://www.degruyter.com/document/doi/10.36019/9780813544724-007/html.

Thanos, Costas A. 2014. « In Situ and Ex Situ Plant Conservation in Greece in the Framework of the Global Strategy for Plant Conservation ». *Bullettin of the Gioenia Academy of Natural Sciences of Catania* 47 (377/SFE): SFE25-30.

Heliotropiaceae

Heliotropium

Sarika, Maria, Alexandros Papanikolaou, Artemios Yannitsaros, Theodoros Chitos, and Maria Panitsa. 2019. 'Temporal Turnover of the Flora of Lake Islands: The Island of Lake Pamvotis (Epirus, Greece)'. *Biodiversity Data Journal* 7 (August): e37023. https://doi.org/10.3897/BDJ.7.e37023.

Hyacinthaceae

Bellevalia

Bareka, P., N.J. Turland, and G. Kamari. 2015. 'Bellevalia Juliana (Asparagaceae), a New Hexaploid Species from E Kriti (Greece)'. Plant Biosystems - An International Journal Dealing with All Aspects of Plant Biology 149 (4): 703–9. https://doi.org/10.1080/11263504.2015.1057258.

Bareka, Pepy, Dimitrios Phitos, and Georgia Kamari. 2008. 'A Karyosystematic Study of the Genus Bellevalia Lapeyr. (Hyacinthaceae) in Greece'. Botanical Journal of the Linnean Society 157 (4): 723–39. https://doi.org/10.1111/j.1095-8339.2008.00817.x.

Fenu, Giuseppe, Gianluigi Bacchetta, S. Christodoulou Charalambos, et al. 2019. 'An Early Evaluation of Translocation Actions for Endangered Plant Species on Mediterranean Islands'. Plant Diversity, Restoration of threatened plant species and their habitats, vol. 41 (2): 94–104. https://doi.org/10.1016/j.pld.2019.03.001.

Krigas, Nikos, Viktoria Menteli, and Despoina Vokou. 2014. 'The Electronic Trade in Greek Endemic Plants: Biodiversity, Commercial and Legal Aspects'. Economic Botany 68 (1): 85–95. https://doi.org/10.1007/s12231-014-9264-9.

Krigas, Nikos, Georgios Tsoktouridis, Ioannis Anestis, et al. 2021. 'Exploring the Potential of Neglected Local Endemic Plants of Three Mediterranean Regions in the Ornamental Sector: Value Chain

Feasibility and Readiness Timescale for Their Sustainable Exploitation'. Sustainability 13 (5): 5. https://doi.org/10.3390/su13052539.

Muscari

Bergmeier, Erwin, Thomas Blockeel, Niels Böhling, et al. 2011. 'An Inventory of the Vascular Plants and Bryophytes of Gavdopoula Island (S Aegean, Greece) and Its Phytogeographical Significance'. *Willdenowia* 41 (1): 179–90. https://doi.org/10.3372/wi.41.41121.

Doussi, Maria A., and Costas A. Thanos. 2002. 'Ecophysiology of Seed Germination in Mediterranean Geophytes. 1. Muscari Spp'. *Seed Science Research* 12 (3): 193–201. https://doi.org/10.1079/SSR2002111.

Giannopoulos, Konstantinos, and Kit Tan. 2021. *Contributions to the Bulb Flora of Ilias (NW Peloponnese, Greece): Hyacinthaceae*.

Hatzilazarou, Stefanos, Ioannis Anestis, Elias Pipinis, et al. 2023. 'GIS-Facilitated Seed Germination of Six Local Endemic Plants of Crete (Greece) and Multifaceted Evaluation in Three Economic Sectors'. *Journal of Biological Research - Thessaloniki* 30 (0): 0. https://doi.org/10.26262/jbrt.v30i0.9019.

Kougioumoutzis, K., A. Tiniakou, O. Georgiou, and T. Georgiadis. 2014. 'Contribution to the Flora of the South Aegean Volcanic Arc: Kimolos Island (Kiklades, Greece)'. *Edinburgh Journal of Botany* 71 (2): 135–60. https://doi.org/10.1017/S0960428614000055.

Kougioumoutzis, K., A. Tiniakou, O. Georgiou, and T. Georgiadis. 2015. 'Contribution to the Flora and Biogeography of the Kiklades: Folegandros Island (Kiklades, Greece)'. *Edinburgh Journal of Botany* 72 (3): 391–412. https://doi.org/10.1017/S0960428615000128.

Karlén, T. 1984. « Muscari pulchellum (Liliaceae) and Associated Taxa in Greece and W Turkey ». *Willdenowia* 14 (1): 89-118.

Krigas, Nikos, Georgios Tsoktouridis, Ioannis Anestis, et al. 2021. 'Exploring the Potential of Neglected Local Endemic Plants of Three Mediterranean Regions in the Ornamental Sector: Value Chain Feasibility and Readiness Timescale for Their Sustainable Exploitation'. *Sustainability* 13 (5): 5. https://doi.org/10.3390/su13052539.

Maloupa, Eleni, Nikos Krigas, Katerina Grigoriadou, Diamanto Lazari, and Georgios Tsoktouridis. 2008. *Conservation Strategies for Native Plant Species and Their Sustainable Exploitation: Case of the Balkan Botanic Garden of Kroussia, N. Greece*. 21.

Oran, S A S. 2015. « Selected Wild Aromatic Plants in Jordan ». *International Journal of Medicinal Plants*, 15.

Ornithogalum

Greuter, Werner, and Thomas Raus. 2012. 'Med-Checklist Notulae, 31'. *Willdenowia* 42 (2): 287–95.

Löve, Áskell. 1984. « Chromosome Number Reports LXXXV ». *Taxon* 33 (4): 756-60.

Rivera, Diego, Concepción Obón, Michael Heinrich, Cristina Inocencio, Alonso Verde, and José Fajardo. 2006. 'Gathered Mediterranean Food Plants – Ethnobotanical Investigations and Historical Development'. In *Local Mediterranean Food Plants and Nutraceuticals, Forum Nutrition*, edited by M. Heinrich, W.E. Müller, and C. Galli, vol. 59. S. Karger AG. https://doi.org/10.1159/000095207.

Scilla

Agricultural University of Athens. 2019. Life Andros Park, 'Conservation of Priority Species and Habitats of Andros Island Protected Area Integrating Socioeconomic Considerations'. Action A.1 - Final Report on the Plant Communities and Their Seasonal and Spatial Variation of the Target Habitat Including the Results of the Base Study. Agricultural University of Athens. http://www.life-androspark.gr/en/wp-content/uploads/2023/01/A1_AUA-Final-Report-on-Vegetation.pdf.

Greilhuber, Johann, Barbara Deumling, and Franz Speta. 1981. 'Evolutionary Aspects of Chromosome Banding, Heterochromatin, Satellite DNA, and Genome Size in Scilla (Liliaceae)'. Berichte Der Deutschen Botanischen Gesellschaft 94 (1): 249–66. https://doi.org/10.1111/j.1438-8677.1981.tb03401.x.

Pfosser, Martin, et Franz Speta. 1999. « Phylogenetics of Hyacinthaceae Based on Plastid DNA Sequences ». Annals of the Missouri Botanical Garden 86 (4): 852. https://doi.org/10.2307/2666172.

Speta, F. 1998. 'Die Scilla-Arten (Hyacinthaceae) der griechischen Inseln Kreta und Karpathos'. Linzer Biol. Beitr. 30 (1): 431–37.

Trigas, Panayiotis, Eleftherios Kalpoutzakis, Epaminondas Kalogiannis, et al. 2021. 'Noteworthy New Floristic Records from Greece'. Botanica Serbica 45 (2): 321–31. https://doi.org/10.2298/BOTSERB2102321T.

Hypericaceae

Hypericum

Agricultural University of Athens. 2019. Life Andros Park, 'Conservation of Priority Species and Habitats of Andros Island Protected Area Integrating Socioeconomic Considerations'. Action A.1 - Final Report on the Plant Communities and Their Seasonal and Spatial Variation of the Target Habitat Including the Results of the Base Study. Agricultural University of Athens. http://www.life-androspark.gr/en/wp-content/uploads/2023/01/A1_AUA-Final-Report-on-Vegetation.pdf.

Athanasas, Konstantinos, Prokopios Magiatis, Nikolas Fokialakis, Alexios-Leandros Skaltsounis, Harris Pratsinis, and Dimitris Kletsas. 2004. 'Hyperjovinols A and B: Two New Phloroglucinol Derivatives from Hypericum Jovis with Antioxidant Activity in Cell Cultures'. Journal of Natural Products 67 (6): 973–77. https://doi.org/10.1021/np034051w.

Couladis, M., I. B. Chinou, O. Tzakou, et P. V. Petrakis. 2003. « Composition and Antimicrobial Activity of the Essential Oil of Hypericum Rumeliacum Subsp. Apollinis (Boiss. & Heldr.) ». *Phytotherapy Research* 17 (2): 152-54. https://doi.org/10.1002/ptr.1093.

Crockett, Sara L., Betul Demirci, K. Husnu Can Başer, and Ikhlas A. Khan. 2007. 'Analysis of the Volatile Constituents of Five African and Mediterranean Hypericum L. (Clusiaceae, Hypericoideae)

Species'. Journal of Essential Oil Research 19 (4): 302–6. https://doi.org/10.1080/10412905.2007.9699287.

Daskalaki, Artemis, Maria-Eleni Grafakou, Christina Barda, Zacharias Kypriotakis, Jörg Heilmann, and Helen Skaltsa. 2021. 'Secondary Metabolites from Hypericum Trichocaulon Boiss. & Heldr., Growing Wild in the Island of Crete'. Biochemical Systematics and Ecology 97 (August): 104294. https://doi.org/10.1016/j.bse.2021.104294.

Dirmenci, Tuncay, and Norman K. B. Robson. 2019. 'Two New Taxa of Hypericum (Hypericaceae) from Turkey'. Kew Bulletin 74 (4): 66. https://doi.org/10.1007/s12225-019-9856-8.

Fournaraki, C, I Remoundou, and C A Thanos. 2008. 'Ex Situ Conservation of European Threatened Plants in Western Crete, Greece (CRETAPLANT Project, EU-LIFE)'. Poster. Agraria Universita Mediterranensium Nationum. http://cretaplant.biol.uoa.gr/posters/FournarakiPerthPoster.pdf.

Galati, E. M., G. Contartese, N. Miceli, M. F. Taviano, V. Sdrafkakis, M. Couladis, O. Tzakou, et F. Lanuzza. 2008. « Antiinflammatory and Antioxidant Activity of Hypericum Rumeliacum Boiss. Subsp. Apollinis (Boiss. & Heldr.) Robson & Strid Methanol Extract ». *Phytotherapy Research* 22 (6): 766-71. https://doi.org/10.1002/ptr.2360.

Grafakou, M. E., A. Diamanti, E. Simirioti, et al. 2019. 'Preliminary results: essential oils from Hypericum spp. growing wild in Greece and their wound healing effects'. Planta Medica 85 (18): P-365. https://doi.org/10.1055/s-0039-3400074.

Grafakou, Maria-Eleni, Christina Barda, Diandra Pintać, Marija Lesjak, Jörg Heilmann, and Helen Skaltsa. 2021. 'Prenylated Acylphloroglucinols from Hypericum Jovis with Anti-Inflammatory Potential'. Planta Medica 87 (14): 1184–91. https://doi.org/10.1055/a-1556-9721.

Grafakou, Maria-Eleni, Aggeliki Diamanti, Eleftheria Antaloudaki, et al. 2020. 'Chemical Composition and Antimicrobial Activity of the Essential Oils of Three Closely Related Hypericum Species Growing Wild on the Island of Crete, Greece'. Applied Sciences 10 (8): 8. https://doi.org/10.3390/app10082823.

Hazler Pilepić, Kroata, Anita Čorić, and Marina Radovčić. 2016. 'Odre\djivanje Sadržaja Ukupnih Hipericina u Odabranim Vrstama Roda Hypericum L.' Farmaceutski Glasnik 72 (6): 377–84.

Howard, Caroline, Eleni Socratous, Sarah Williams, et al. 2012. 'PlantID – DNA-Based Identification of Multiple Medicinal Plants in Complex Mixtures'. Chinese Medicine 7 (1): 18. https://doi.org/10.1186/1749-8546-7-18.

Kakouri, Eleni, Dimitra Daferera, Panayiotis Trigas, et al. 2023. 'Comparative Study of the Antibacterial Activity, Total Phenolic and Total Flavonoid Content of Nine Hypericum Species Grown in Greece'. Applied Sciences 13 (5): 5. https://doi.org/10.3390/app13053305.

Kakouri, Eleni, Panayiotis Trigas, Dimitra Daferera, Efstathia Skotti, Petros A. Tarantilis, and Charalabos Kanakis. 2023. 'Chemical Characterization and Antioxidant Activity of Nine Hypericum Species from Greece'. Antioxidants 12 (4): 4. https://doi.org/10.3390/antiox12040899.

Kolliopoulos, Alexandros. 2019. « Micropropagation of Hypericum taygeteum ». Kalamata.

Krigas, Nikos, Georgios Tsoktouridis, Ioannis Anestis, et al. 2021. 'Exploring the Potential of Neglected Local Endemic Plants of Three Mediterranean Regions in the Ornamental Sector: Value Chain Feasibility and Readiness Timescale for Their Sustainable Exploitation'. *Sustainability* 13 (5): 5. https://doi.org/10.3390/su13052539.

Madunić, Josip, Maja Matulić, Maja Friščić, and Kroata Hazler Pilepić. 2016. 'Evaluation of the Cytotoxic Activity of Hypericum Spp. on Human Glioblastoma A1235 and Breast Cancer MDA MB-231 Cells'. Journal of Environmental Science and Health, Part A 51 (13): 1157–63. https://doi.org/10.1080/10934529.2016.1206385.

Mathioudaki, A., M. Löhr, S. Schmidt, G. Jürgenliemk, H. Skaltsa, and J. Heilmann. 2013. 'Isolation & Structure Elucidation of Acylphloroglucinols from Hypericum Amblycalyx Coustur. & Gand.' Planta Medica 79 (13): PI72. https://doi.org/10.1055/s-0033-1352161.

Mathioudaki, Angeliki, Ariola Berzesta, Zacharias Kypriotakis, Helen Skaltsa, and Jörg Heilmann. 2018. 'Phenolic Metabolites from Hypericum Kelleri Bald., an Endemic Species of Crete (Greece)'. Phytochemistry 146 (February): 1–7. https://doi.org/10.1016/j.phytochem.2017.11.009.

Nürk, Nicolai M., and Sara L. Crockett. 2011. 'Morphological and Phytochemical Diversity among Hypericum Species of the Mediterranean Basin'. Medicinal and Aromatic Plant Science and Biotechnology 5 (Special Issue 1): 14–28.

Tan, Kit, Gregoris Iatroú, Gert Vold, et Arne Strid. 2010. « Hypericum Boehlingraabei (Hypericaceae), a New Species from the Northern Peloponnese (Greece) », 6.

Trigas, Panayiotis. 2018. 'A New Hypericum (Sect. Drosocarpium, Hypericaceae) from the Cyclades Islands (Greece)'. Nordic Journal of Botany 36 (11): e02205. https://doi.org/10.1111/njb.02205.

Vladimirov, Vladimir, Mehmet Aybeke, and Kit Tan. 2018. 'New Floristic Records in the Balkans: 35*'. Phytologia Balcanica: International Journal of Balkan Flora and Vegetation. Phytologia Balcanica: International Journal of Balkan Flora and Vegetation 24 (1): 155–74.

Winkelmann, Karin, Metin San, Zacharias Kypriotakis, Helen Skaltsa, Blazenka Bosilij, and Jörg Heilmann. 2003. 'Antibacterial and Cytotoxic Activity of Prenylated Bicyclic Acylphloroglucinol Derivatives from Hypericum Amblycalyx'. Zeitschrift Für Naturforschung C 58 (7–8): 527–32. https://doi.org/10.1515/znc-2003-7-814.

Κώστογλου, Δήμητρα. 2021. « Συγκριτική μελέτη διαφόρων ειδών Hypericum ως προς τη χημική τους σύσταση, το ολικό φαινολικό περιεχόμενο, την αντιοξειδωτική ικανότητα και την αντιμικροβιακή τους δράση - Comparative study of different species of Hypericum in terms of their chemical composition, total phenolic content, antioxidant capacity and antimicrobial activity », janvier. http://dspace.aua.gr/xmlui/handle/10329/7208.

Τσιλιάκου, Τζιόρτζιου, and Δήμητρα Βασιλείου. 2023. 'Μελέτη Αιθερίων Ελαίων του Γένους Hypericum (Hypericaceae)'. In Aristotle University of Thessaloniki Institutional Repository -

IKEE, GRI-2023-40547. Αριστοτέλειο Πανεπιστήμιο Θεσσαλονίκης. https://ikee.lib.auth.gr/record/350495.

Iridaceae

Crocus

Archibald, Jim, et Jenny Archibald. 1998. « The Need to Know - News from Jim and Jenny Archibald, Newsletter and seed list », septembre 1998. http://files.srgc.net/archibald/seedlists/JJA_seeds/JJA_seeds_1998.2_September.pdf.

Castillo, Raquel, José-Antonio Fernández, et Lourdes Gómez-Gómez. 2005. « Implications of Carotenoid Biosynthetic Genes in Apocarotenoid Formation during the Stigma Development of Crocus sativus and Its Closer Relatives ». *Plant Physiology* 139 (2): 674-89. https://doi.org/10.1104/pp.105.067827.

Chryssanthi, Dimitra G., Fotini N. Lamari, Gregoris Iatrou, Adamantia Pylara, Nikos K. Karamanos, et Paul Cordopatis. 2007. « Inhibition of Breast Cancer Cell Proliferation by Style Constituents of Different Crocus Species ». *Anticancer Research* 27 (1A): 357-62.

Diretto, Gianfranco, Oussama Ahrazem, Ángela Rubio-Moraga, et al. 2019. 'UGT709G1: A Novel Uridine Diphosphate Glycosyltransferase Involved in the Biosynthesis of Picrocrocin, the Precursor of Safranal in Saffron (*Crocus Sativus*)'. *New Phytologist* 224 (2): 725–40. https://doi.org/10.1111/nph.16079.

Ferrence, Susan C., et Gordon Bendersky. 2004. « Therapy with Saffron and the Goddess at Thera ». *Perspectives in Biology and Medicine* 47 (2): 199-226. https://doi.org/10.1353/pbm.2004.0026.

Harborne, Jeffrey B., et Christine A. Williams. 1984. « 6-Hydroxyflavones and Other Flavonoids of Crocus ». *Zeitschrift Für Naturforschung C* 39 (1-2): 18-23. https://doi.org/10.1515/znc-1984-1-204.

Harpke, Dörte, Shuchun Meng, Twan Rutten, Helmut Kerndorff, and Frank R. Blattner. 2013. 'Phylogeny of Crocus (Iridaceae) Based on One Chloroplast and Two Nuclear Loci: Ancient Hybridization and Chromosome Number Evolution'. *Molecular Phylogenetics and Evolution* 66 (3): 617–27. https://doi.org/10.1016/j.ympev.2012.10.007.

Harpke, Dörte, Lorenzo Peruzzi, Helmut Kerndorff, et al. 2014. 'Phylogeny, Geographic Distribution, and New Taxonomic Circumscription of the Crocus Reticulatus Species Group (Iridaceae)'. *TURKISH JOURNAL OF BOTANY* 38: 1182–98. https://doi.org/10.3906/bot-1405-60.

Jacobsen, N., et M. Ørgaard. 2004. « Crocus cartwrightianus on the Attica peninsula ». *Acta Horticulturae*, nº 650 (mai): 65-69. https://doi.org/10.17660/ActaHortic.2004.650.6.

Karamplianis, Theophanis, and Theophanis Constantinidis. 2019. 'The Taxa of Crocus Ser. Flavi (Iridaceae) in Greece: A Taxonomic and Karyomorphometric Study.' *Botanika Chronika* 22: 171–94.

Kazemi-Shahandashti, Seyyedeh-Sanam, Ludwig Mann, Abdullah El-nagish, et al. 2022. 'Ancient Artworks and Crocus Genetics Both Support Saffron's Origin in Early Greece'. *Frontiers in Plant Science* 13. https://www.frontiersin.org/articles/10.3389/fpls.2022.834416.

Koulakiotis, Ns, E Gikas, Dg Chryssanthi, Fn Lamari, P Cordopatis, et A Tsarbopoulos. 2008. « A New HPLC Method for the Analysis of Crocus Sativus Styles ». *Planta Medica* 74 (09): s-0028-1084639. https://doi.org/10.1055/s-0028-1084639.

Krigas, Nikos, Viktoria Menteli, et Despoina Vokou. 2014. « The Electronic Trade in Greek Endemic Plants: Biodiversity, Commercial and Legal Aspects ». *Economic Botany* 68 (1): 85-95. https://doi.org/10.1007/s12231-014-9264-9.

Lamari, Fotini N., Vassilis Papasotiropoulos, Dimitris Tsiris, Stavros E. Bariamis, Konstantinos Sotirakis, Efthimia Pitsi, Amalia P. Vogiatzoglou, et Gregoris Iatrou. 2018. « Phytochemical and Genetic Characterization of Styles of Wild Crocus Species from the Island of Crete, Greece and Comparison to Those of Cultivated C. Sativus ». *Fitoterapia* 130 (octobre): 225-33. https://doi.org/10.1016/j.fitote.2018.09.003.

Lawrence, Elizabeth. 2001. *A Southern Garden*. UNC Press Books.

Mathew, B., et C. A. Brighton. 1977. « Crocus tournefortei and Its Allies (Iridaceæ) ». *Kew Bulletin* 31 (4): 775-84. https://doi.org/10.2307/4109550.

Nemati, Zahra, Dörte Harpke, Almila Gemicioglu, Helmut Kerndorff, et Frank R. Blattner. 2019. « Saffron (Crocus Sativus) Is an Autotriploid That Evolved in Attica (Greece) from Wild Crocus Cartwrightianus ». *Molecular Phylogenetics and Evolution* 136 (juillet): 14-20. https://doi.org/10.1016/j.ympev.2019.03.022.

Pylara, Adamantias. 2007. « Συμβολή στην κυτταρολογία, μορφομετρία και χημική ανάλυση των στύλων ενδημικών ειδών του γένους Crocus L. από την Πελοπόννησο - Contribution to the cytology, morphometry and chemical analysis of the endemic species of the genus Crocus L. from Peloponnese ». Patra: 1 ΠΑΝΕΠΙΣΤΗΜΙΟ ΠΑΤΡΩΝ ΣΧΟΛΗ ΘΕΤΙΚΩΝ ΕΠΙΣΤΗΜΩΝ ΤΜΗΜΑ ΒΙΟΛΟΓΙΑΣ ΤΟΜΕΑΣ ΒΙΟΛΟΓΙΑΣ ΦΥΤΩΝ.

Ralli, P., et C. Dordas. 2012. « In Situ Conservation of Crocus Cartwrightianus in Cyclades and Crete ». In *Agrobiodiversity Conservation: Securing the Diversity of Crop Wild Relatives and Landraces*, par Nigel Maxted. CABI.

Richards, John. 2018. 'Notes on Some Autumn-Flowering Crocus from North-Western Greece'. *Crocus Group Newsletter* 49.

Wan, Xia, and Li-Bing Zhang. 2022. 'Global New Taxa of Vascular Plants Published in 2021'. *Biodiversity Science* 30 (8): 22116.

Iris

Kalpoutzakis, Eleftherios, Theodoros Chatzimitakos, Vassilis Athanasiadis, et al. 2023. 'Determination of the Total Phenolics Content and Antioxidant Activity of Extracts from Parts of Plants from the Greek Island of Crete'. *Plants* 12 (5): 5. https://doi.org/10.3390/plants12051092.

Makropoulou, M., C. Karakasi, N. Aligiannis, et al. 2015. 'Greek Iris Species as Sources of Agents Potentially Effective in Bone Metabolism'. *Planta Medica* 81 (16): PM_60. https://doi.org/10.1055/s-0035-1565437.

Mermygkas, Dionysios, Kit Tan, et Artemios Yannitsaros. 2010. « A New Species of Iris (Iridaceae) from the Northern Peloponnese (Greece) ». *Phytologia Balcanica*, 4.

Lamiaceae

Acinos

GBIF. n.d. 'GBIF Acinos Nanus'. Accessed 12 April 2019. https://www.gbif.org/species/3894955.

Turland, Nicholas, Dimitrios Phitos, Georgia Kamari, and Pepy Bareka. 2004. 'Weeds of the Traditional Agriculture of Crete'. Willdenowia 34 (2): 381–406. https://doi.org/10.3372/wi.34.34206.

Calamintha

Bourgou, Soumaya, Imtinen Ben Haj Jilani, Olfa Karous, et al. 2021. 'Medicinal-Cosmetic Potential of the Local Endemic Plants of Crete (Greece), Northern Morocco and Tunisia: Priorities for Conservation and Sustainable Exploitation of Neglected and Underutilized Phytogenetic Resources'. *Biology* 10 (12): 1344. https://doi.org/10.3390/biology10121344.

Greuter, Werner, and Thomas Raus. 2012. 'Med-Checklist Notulae, 31'. *Willdenowia* 42 (2): 287–95. https://doi.org/10.3372/wi.42.42215.

Grigoriadou, K., N. Krigas, V. Sarropoulou, K. Papanastasi, G. Tsoktouridis, and E. Maloupa. 2019. 'In Vitro Propagation of Medicinal and Aromatic Plants: The Case of Selected Greek Species with Conservation Priority'. *In Vitro Cellular & Developmental Biology - Plant* 55 (6): 635–46. https://doi.org/10.1007/s11627-019-10014-6.

Karousou, R., S. Kokkini, J-M. Bessière, and D. Vokou. 1996. 'Calamintha Cretica (Lamiaceae), a Cretan Endemic: Distribution and Essential Oil Composition'. *Nordic Journal of Botany* 16 (3): 247–52. https://doi.org/10.1111/j.1756-1051.1996.tb00224.x.

Krigas, Nikos, Georgios Tsoktouridis, Ioannis Anestis, et al. 2021. 'Exploring the Potential of Neglected Local Endemic Plants of Three Mediterranean Regions in the Ornamental Sector: Value Chain Feasibility and Readiness Timescale for Their Sustainable Exploitation'. *Sustainability* 13 (5): 5. https://doi.org/10.3390/su13052539.

Libiad, Mohamed, Abdelmajid Khabbach, Mohamed El Haissoufi, et al. 2021. 'Agro-Alimentary Potential of the Neglected and Underutilized Local Endemic Plants of Crete (Greece), Rif-Mediterranean Coast of Morocco and Tunisia: Perspectives and Challenges'. *Plants* 10 (9): 9. https://doi.org/10.3390/plants10091770.

Menteli, Viktoria, Nikos Krigas, Manolis Avramakis, Nicholas Turland, and Despoina Vokou. 2019. 'Endemic Plants of Crete in Electronic Trade and Wildlife Tourism: Current Patterns and Implications for Conservation'. *Journal of Biological Research-Thessaloniki* 26 (1): 10. https://doi.org/10.1186/s40709-019-0104-z.

Papafotiou, M., K.F. Bertsouklis, A.N. Martini, et al. 2017. 'Evaluation of the Establishment of Native Mediterranean Plant Species Suggested for Landscape Enhancement in Archaeological Sites of Greece'. *Acta Horticulturae*, no. 1189 (December): 177–80. https://doi.org/10.17660/ActaHortic.2017.1189.35.

Papafotiou, M., A.N. Martini, and G. Vlachou. 2017. 'In Vitro Propagation as a Tool to Enhance the Use of Native Ornamentals in Archaeological Sites of Greece'. *Acta Horticulturae*, no. 1155 (March): 301–8. https://doi.org/10.17660/ActaHortic.2017.1155.43.

Vlachou, Georgia, Maria Maria Papafotiou, and Konstantinos Bertsouklis. 2020. 'Seed Germination, Micropropagation from Adult and Juvenile Origin Explants and Address of Hyperhydricity of the Cretan Endemic Herb Calamintha Cretica'. *Notulae Botanicae Horti Agrobotanici Cluj-Napoca* 48 (3): 3. https://doi.org/10.15835/nbha48311926.

Δρόσος, Παναγιώτης. 2019. *Η Χρήση Των Αιθέριων Ελαίων Στη Διατροφή Των Ορνίθων Κρεοπαραγωγικού Τύπου / THE USE OF ESSENTIAL OILS IN THE FEED OF MEAT-BREEDING CHICKENS*. December 3. https://apothetirio.lib.uoi.gr/xmlui/handle/123456789/10590.

Clinopodium

Kyriakopoulos Ch., Bareka P., Kamari G. 2016. « Karyological Data of Some Endemic Taxa from Mt Taigetos, Greece ». *Flora Mediterranea* 26 (décembre). https://doi.org/10.7320/FlMedit26.224.

Lamium

Ghalkhani, Atieh, Shirin Moradkhani, Meysam Soleimani, et Dara Dastan. 2021. « Functional Components, Antibacterial, Antioxidant, and Cytotoxic Activities of Lamium Garganicum L. Ssp. Pictum as a Novel Natural Agents from Lamiaceae Family ». *Food Bioscience* 43 (octobre): 101265. https://doi.org/10.1016/j.fbio.2021.101265.

Marrubium

Karioti, Anastasia, Jörg Heilmann, et Helen Skaltsa. 2005. « Labdane diterpenes from Marrubium velutinum and Marrubium cylleneum ». *Phytochemistry* 66 (9): 1060-66. https://doi.org/10.1016/j.phytochem.2005.02.029.

Karioti, Anastasia, Anastasia Protopappa, Nikolaos Megoulas, et Helen Skaltsa. 2007. « Identification of tyrosinase inhibitors from Marrubium velutinum and Marrubium cylleneum ». *Bioorganic & Medicinal Chemistry* 15 (7): 2708-14. https://doi.org/10.1016/j.bmc.2007.01.035.

Karioti, Anastasia, Helen Skaltsa, Jörg Heilmann, et Otto Sticher. 2003. « Acylated flavonoid and phenylethanoid glycosides from Marrubium velutinum ». *Phytochemistry*, Special Issue in memory of Professor Jeffrey B. Harborne, 64 (2): 655-60. https://doi.org/10.1016/S0031-9422(03)00242-5.

Karioti, Anastasia, Margarita Skopeliti, Ourania Tsitsilonis, Jörg Heilmann, et Helen Skaltsa. 2007. « Cytotoxicity and immunomodulating characteristics of labdane diterpenes from Marrubium cylleneum and Marrubium velutinum ». *Phytochemistry* 68 (11): 1587-94. https://doi.org/10.1016/j.phytochem.2007.03.027.

Kozyra, Malgorzata, Agnieszka Korga, Marta Ostrowska, Ewelina Humeniuk, Grzegorz Adamczuk, Renata Gieroba, Anna Makuch-

Kocka, et Jaroslaw Dudka. 2020. « Cytotoxic Activity of Methanolic Fractions of Different Marrubium Spp. against Melanoma Cells Is Independent of Antioxidant Activity and Total Phenolic Content ». *FEBS Open Bio* 10 (1): 86-95. https://doi.org/10.1002/2211-5463.12755.

Lazari, Diamanto M., Helen D. Skaltsa, et Theophanis Constantinidis. 1999. « Essential Oils of Marrubium Velutinum Sm. and Marrubium Peregrinum L., Growing Wild in Greece ». *Flavour and Fragrance Journal* 14 (5): 290-92. https://doi.org/10.1002/(SICI)1099-1026(199909/10)14:5<290::AID-FFJ828>3.0.CO;2-4.

Michelis, Fotios, Ekaterini Tiligada, Helen Skaltsa, Diamanto Lazari, Alexios-Leandros Skaltsounis, et Andreas Delitheos. 2002. « Effects of the Flavonoid Pilloin Isolated from Marrubium Cylleneum on Mitogen-Induced Lymphocyte Transformation ». *Pharmaceutical Biology* 40 (4): 245-48. https://doi.org/10.1076/phbi.40.4.245.8472.

Micromeria

Bräuchler, Christian, Olof Ryding, and Günther Heubl. 2008. 'The Genus Micromeria (Lamiaceae), a Synoptical Update'. *Willdenowia* 38 (2): 363–410. https://doi.org/10.3372/wi.38.38202.

Greuter, Werner, and Thomas Raus. 2012. 'Med-Checklist Notulae, 31'. *Willdenowia* 42 (2): 287–95. https://doi.org/10.3372/wi.42.42215.

Krigas, Nikos, Georgios Tsoktouridis, Ioannis Anestis, et al. 2021. 'Exploring the Potential of Neglected Local Endemic Plants of Three Mediterranean Regions in the Ornamental Sector: Value Chain Feasibility and Readiness Timescale for Their Sustainable Exploitation'. *Sustainability* 13 (5): 5. https://doi.org/10.3390/su13052539.

Libiad, Mohamed, Abdelmajid Khabbach, Mohamed El Haissoufi, et al. 2021. 'Agro-Alimentary Potential of the Neglected and Underutilized Local Endemic Plants of Crete (Greece), Rif-Mediterranean Coast of Morocco and Tunisia: Perspectives and Challenges'. *Plants* 10 (9): 9. https://doi.org/10.3390/plants10091770.

Panistsa, Maria, and Eleni Iliadou. 2013. 'Flora and Phytogeography of the Ionian Islands (Greece)'. https://www.researchgate.net/profile/Marco_Simeone/publication/216634523_Endemic_taxa_of_Asperula_L_sect_Cynanchicae_DC_Boiss_on_the_Mediterranean_Islands/links/54e36bb40cf2b2314f5d1b1e/Endemic-taxa-of-Asperula-L-sect-Cynanchicae-DC-Boiss-on-the-Mediterranean-Islands.pdf#page=244.

Siljak-Yakovlev, Sonja, Kit Tan, Gregory Tsounis, and Lambros Tsounis. 2011. *Genome Size and Chromosome Number of Micromeria Acropolitana (Lamiaceae), a Steno-Endemic from Greece*. 4.

Tan, Kit, Burkhard Biel, and Jerzy Zieliński. 2020. 'Micromeria Zarkosii (Lamiaceae), an Unusual New Species from Naxos (Central Kiklades, Greece)'. *Nordic Journal of Botany* 38 (11). https://doi.org/10.1111/njb.02975.

Tan, Kit, Gregory Tsounis, and Lambros Tsounis. 2010. *Micromeria Acropolitana (Lamiaceae) Rediscovered in Athens (Greece)*. 6.

Tan, Kit, and Jerzy Zieliński. 2023. 'Micromeria Acropolitana (Lamiaceae) – Epilogue'. *Phytologia Balcanica* 29 (1). https://doi.org/10.7546/PhB.29.1.2023.6.

Tomás-Barberán, Franclsco A., Syed Z. Husain, and Maria I. Gil. 1988. 'The Distribution of Methylated Flavones in the Lamiaceae'. *Biochemical Systematics and Ecology* 16 (1): 43–46. https://doi.org/10.1016/0305-1978(88)90115-9.

Valli, Anna-Thalassini, Christos Chondrogiannis, George Grammatikopoulos, Gregoris Iatrou, and Panayiotis Trigas. 2021. 'Conservation of Micromeria Browiczii (Lamiaceae), Endemic to Zakynthos Island (Ionian Islands, Greece)'. *Plants* 10 (4): 4. https://doi.org/10.3390/plants10040778.

Βαλλή, Άννα-Θαλασσινή. 2013. 'Καταγραφή της χλωρίδας του Εθνικού Θαλάσσιου Πάρκου Ζακύνθου και βιο-παρακολούθηση των αποκλειστικά ενδημικών φυτικών taxa της Ζακύνθου'. Thesis. http://nemertes.lis.upatras.gr/jspui//handle/10889/7845.

Πιτιακούδη, Σωτήριου. 2022. 'Μελέτη των Διατροφικών Συνηθειών και της Χρήσης των Εδώδιμων Φαρμακευτικών Φυτών των Κατοίκων του Δήμου Σουφλίου'. Lesson. Program of Postgraduate studies, Primary Health Care, Social Medicine and Pharmaceutical Care, Hellenic Republic, Democritus University of Thrace, Medical School. https://repo.lib.duth.gr/jspui/bitstream/123456789/15225/1/PitiakoudisS_2022.pdf.

Nepeta

Aničić, Neda, Uroš Gašić, Feng Lu, Ana Ćirić, Marija Ivanov, Bojan Jevtić, Milena Dimitrijević, et al. 2021. « Antimicrobial and Immunomodulating Activities of Two Endemic Nepeta Species and Their Major Iridoids Isolated from Natural Sources ». *Pharmaceuticals* 14 (5): 414. https://doi.org/10.3390/ph14050414.

Aničić, Neda, Dragana Matekalo, Marijana Skorić, Jasmina Nestorović Živković, Luka Petrović, Milan Dragićević, Slavica Dmitrović, et Danijela Mišić. 2020. « Alterations in Nepetalactone Metabolism during Polyethylene Glycol (PEG)-Induced Dehydration Stress in Two Nepeta Species ». *Phytochemistry* 174 (juin): 112340. https://doi.org/10.1016/j.phytochem.2020.112340.

Baden, C. 1984. « Chromosome Numbers in the Nepeta sibthorpii Group (Lamiaceae) ». *Willdenowia* 13 (2): 337-40.

Baranauskienė, Renata, Vilma Bendžiuvienė, Ona Ragažinskienė, and Petras Rimantas Venskutonis. 2019. 'Essential Oil Composition of Five Nepeta Species Cultivated in Lithuania and Evaluation of Their Bioactivities, Toxicity and Antioxidant Potential of Hydrodistillation Residues'. *Food and Chemical Toxicology* 129 (July): 269–80. https://doi.org/10.1016/j.fct.2019.04.039.

Costa, R. M. S., P. Pavone, R. A. Carbonaro, et S. Pulvirenti. 2020. « An anonymous pre-Linnaean herbarium among the "treasures" of the "Civica and A. Ursino Recupero" joint library of Catania (Italy) ». *Plant Biosystems - An International Journal Dealing with all Aspects of Plant Biology* 154 (6): 910-23. https://doi.org/10.1080/11263504.2020.1722273.

Darras, A. I., I. Spiliopoulos, E. Kartsonas, P. Assimomitis, et S. Karras. 2020. « Antioxidant Profile, Propagation and Cultivation of

Nepeta Camphorata, the Endemic Species of Mt Taygetos (Greece) ». *South African Journal of Botany* 131 (juillet): 391-97. https://doi.org/10.1016/j.sajb.2020.03.009.

Dienaitė, Lijana, Milda Pukalskienė, Ana A. Matias, Carolina V. Pereira, Audrius Pukalskas, and Petras Rimantas Venskutonis. 2018. 'Valorization of Six Nepeta Species by Assessing the Antioxidant Potential, Phytochemical Composition and Bioactivity of Their Extracts in Cell Cultures'. *Journal of Functional Foods* 45 (June): 512–22. https://doi.org/10.1016/j.jff.2018.04.004.

Hanlidou, Effie, and Stella Kokkini. 1997. 'On the Flora of the Vikos-Aoos National Park (NW Greece)'. *Willdenowia* 27 (1/2): 81–100. https://doi.org/10.3372/wi.27.2708.

Kalpoutzakis, Eleftherios, Nektarios Aligiannis, Andreas Mentis, Sofia Mitaku, et Catherine Charvala. 2001. « Composition of the Essential Oil of Two Nepeta Species and in vitro Evaluation of their Activity against Helicobacter pylori ». *Planta Medica* 67 (9): 880-83. https://doi.org/10.1055/s-2001-18851.

Kalpoutzakis, Eleftherios, Theophanis Constantinidis, et Panayiotis Trigas. 2019. « Chorological Additions for Some Noteworthy Taxa of the Greek Flora », 18.

Kovtun-Vodyanitska, S. 2019. « ІНТРОДУКЦІЯ НЕТРАДИЦІЙНИХ ЕФІРООЛІЙНИХ РОСЛИН У НАЦІОНАЛЬНОМУ БОТАНІЧНОМУ САДУ ІМЕНІ М.М. ГРИШКА НАН УКРАЇНИ: ПРОБЛЕМНІ ВИДИ ». *Вісник Львівського університету. Серія біологічна* 0 (80): 52-58. https://doi.org/10.30970/vlubs.2019.80.06.

Krigas, Nikos, Georgios Tsoktouridis, Ioannis Anestis, et al. 2021. 'Exploring the Potential of Neglected Local Endemic Plants of Three Mediterranean Regions in the Ornamental Sector: Value Chain Feasibility and Readiness Timescale for Their Sustainable Exploitation'. *Sustainability* 13 (5): 5. https://doi.org/10.3390/su13052539.

Kyriakopoulos, Ch., Kamari, G., Kofinas, I. & Phitos, D. 2018. « Potentilla Greuteriana (Rosaceae), a New Species from Mt. Taigetos (S Peloponnisos, Greece) ». *Flora Mediterranea* 28 (décembre). https://doi.org/10.7320/FlMedit28.351.

Libiad, Mohamed, Abdelmajid Khabbach, Mohamed El Haissoufi, et al. 2021. 'Agro-Alimentary Potential of the Neglected and Underutilized Local Endemic Plants of Crete (Greece), Rif-Mediterranean Coast of Morocco and Tunisia: Perspectives and Challenges'. *Plants* 10 (9): 9. https://doi.org/10.3390/plants10091770.

Proestos, Charalampos, Konstantina Lytoudi, Olga Konstantina Mavromelanidou, Panagiotis Zoumpoulakis, and Vassileia J. Sinanoglou. 2013. 'Antioxidant Capacity of Selected Plant Extracts and Their Essential Oils'. *Antioxidants* 2 (1): 1. https://doi.org/10.3390/antiox2010011.

Skaltsa, Helen D., Diamanto M. Lazari, Anargyros E. Loukis, et Theophanis Constantinidis. 2000. « Essential Oil Analysis of Nepeta Argolica Bory & Chaub. Subsp. Argolica (Lamiaceae) Growing Wild in Greece ». *Flavour and Fragrance Journal* 15 (2): 96-99. https://doi.org/10.1002/(SICI)1099-1026(200003/04)15:2<96::AID-FFJ873>3.0.CO;2-F.

Thanos, Costas A., Christini Fournaraki, Kyriacos Georghiou, and Panayotis Dimopoulos. 2013. 'PMRs in Western Crete'. https://www.researchgate.net/profile/Costas_Thanos/publication/258119110_PMRs_in_Western_Crete/links/02e7e5270d8a300f04000000.pdf.

Thanos, Costas A., Apostolis Kaltsis, and Katerina Koutsovoulou. 2013. 'PMRs-as-Field-Laboratories-for-Scientific-Research'. https://www.researchgate.net/profile/Costas_Thanos/publication/258119615_PMRs_as_Field_Laboratories_for_Scientific_Research/links/0c9605270dbe66b968000000/PMRs-as-Field-Laboratories-for-Scientific-Research.pdf.

Tzakou, O., C. Harvala, E. M. Galati, et R. Sanogo. 2000. « Essential oil composition of Nepeta argolica Bory et Chaub. subsp. argolica ». *Flavour and Fragrance Journal* 15 (2): 115-18. https://doi.org/10.1002/(SICI)1099-1026(200003/04)15:2<115::AID-FFJ877>3.0.CO;2-9.

Origanum

Aćimović, Milica, Jovana Stanković, Mirjana Cvetković, et al. 2020. 'Chemical Composition of Origanum Dictamnus and Origanum Vulgare Ssp. Hirtum from Greece'. Book of Proceedings - GEA (Geo Eco-Eco Agro) International Conference 1: 244–49.

Aligiannis, N., E. Kalpoutzakis, Sofia Mitaku, and Ioanna B. Chinou. 2001. 'Composition and Antimicrobial Activity of the Essential Oils of Two Origanum Species'. Journal of Agricultural and Food Chemistry 49 (9): 4168–70. https://doi.org/10.1021/jf001494m.

Aligiannis, N., E. Kalpoutzakis, Sofia Mitaku, et Ioanna B. Chinou. 2001. « Composition and Antimicrobial Activity of the Essential Oils of Two Origanum Species ». Journal of Agricultural and Food Chemistry 49 (9): 4168-70. https://doi.org/10.1021/jf001494m.

Amaya Olivas, Nubia, Cindy Villalba Bejarano, Guillermo Ayala Soto, et al. 2020. 'Bioactive Compounds and Antioxidant Activity of Essential Oils of Origanum Dictamnus from Mexico'. AIMS Agriculture and Food 5 (3): 387–94. https://doi.org/10.3934/agrfood.2020.3.387.

Carlström, Annette. 1984. 'New Species of Alyssum, Consolida, Origanum and Umbilicus from the SE Aegean Sea'. Willdenowia 14 (1): 15–26. JSTOR.

Chinou, Ioanna, Christos Liolios, Dimitri Moreau, and Christos Roussakis. 2007. 'Cytotoxic Activity of Origanum Dictamnus'. Fitoterapia 78 (5): 342–44. https://doi.org/10.1016/j.fitote.2007.02.005.

Chishti, Shayista, Zahoor A Kaloo, and Phalestine Sultan. 2013. Medicinal Importance of Genus Origanum: A Review. 8.

Chorianopoulos, Nikos, Eleftherios Kalpoutzakis, Nektarios Aligiannis, Sofia Mitaku, George-John Nychas, and Serkos A. Haroutounian. 2004. 'Essential Oils of Satureja , Origanum , and Thymus Species: Chemical Composition and Antibacterial Activities Against Foodborne Pathogens'. Journal of Agricultural and Food Chemistry 52 (26): 8261–67. https://doi.org/10.1021/jf049113i.

Couladis, Maria, Olga Tzakou, Evmorfia Verykokidou, and Catherine Harvala. 2003. 'Screening of Some Greek Aromatic Plants for Antioxidant Activity'. Phytotherapy Research 17 (2): 194–95. https://doi.org/10.1002/ptr.1261.

Dadalioğlu, Itir, et Gulsun Akdemir Evrendilek. 2004. « Chemical Compositions and Antibacterial Effects of Essential Oils of Turkish

Oregano (Origanum Minutiflorum), Bay Laurel (Laurus Nobilis), Spanish Lavender (Lavandula Stoechas L.), and Fennel (Foeniculum Vulgare) on Common Foodborne Pathogens ». Journal of Agricultural and Food Chemistry 52 (26): 8255-60. https://doi.org/10.1021/jf049033e.

Deepa, N., K. Madhivadhani, M. Hari, N. Prakash, S. Lohith, and C. Prabakaran. 2021. 'Comparison of Anti-Inflammatory Activity of Various Species of Lamiaceae'. International Journal of Pharmacometrics and Integrated Biosciences 6 (1): 1. https://doi.org/10.26452/ijpib.v6i1.1405.

Demetzos, Costas, Dimitrios K. Perdetzoglou, and Kit Tan. 2001. 'Composition and Antimicrobial Studies of the Oils of Origanum Calcaratum Juss. and O. Scabrum Boiss. et Heldr. from Greece'. Journal of Essential Oil Research 13 (6): 460–62. https://doi.org/10.1080/10412905.2001.9699729.

Demetzos, Costas, Dimitrios K. Perdetzoglou, et Kit Tan. 2001. « Composition and Antimicrobial Studies of the Oils of Origanum Calcaratum Juss. and O. Scabrum Boiss. et Heldr. from Greece ». Journal of Essential Oil Research 13 (6): 460-62. https://doi.org/10.1080/10412905.2001.9699729.

Economakis, Costas, Costas Demetzos, Thalia Anastassaki, et al. 1999. 'Volatile Constituents of Bracts and Leaves of Wild and Cultivated Origanum Dictamnus'. Planta Medica 65 (2): 189–91. https://doi.org/10.1055/s-2006-960466.

Fanourakis, Dimitrios, Konstantinos Paschalidis, Georgios Tsaniklidis, et al. 2022. 'Pilot Cultivation of the Local Endemic Cretan Marjoram Origanum Microphyllum (Benth.) Vogel (Lamiaceae): Effect of Fertilizers on Growth and Herbal Quality Features'. Agronomy 12 (1): 1. https://doi.org/10.3390/agronomy12010094.

Figuérédo, Gilles, Patrick Cabassu, Jean-Claude Chalchat, and Bernard Pasquier. 2006. 'Studies of Mediterranean Oregano Populations. VIII—Chemical Composition of Essential Oils of Oreganos of Various Origins'. Flavour and Fragrance Journal 21 (1): 134–39. https://doi.org/10.1002/ffj.1543.

Fitsiou, Eleni, and Aglaia Pappa. 2019. 'Anticancer Activity of Essential Oils and Other Extracts from Aromatic Plants Grown in Greece'. Antioxidants 8 (8): 8. https://doi.org/10.3390/antiox8080290.

Ganos, Christos G., Olga Gortzi, Efrossini B. Chinou, Gioacchino Calapai, and Ioanna B. Chinou. 2022. 'Antimicrobial Properties of Selected Native Greek Aromatic Plants: An Ethnopharmacological Overview'. In Promising Antimicrobials from Natural Products, edited by Mahendra Rai and Ivan Kosalec. Springer International Publishing. https://doi.org/10.1007/978-3-030-83504-0_6.

Giri, Ashok, Swapnil Mundhe, and Mileend Shimpi. 2019. 'Herbal Antidotes for the Management of Snake Bite.' World Journal of Pharmacy and Pharmaceutical Sciences 9 (1): 10.

Gotsiou, P, G Naxakis, and M Skoula. 2002. 'Diversity in the Composition of Monoterpenoids of Origanum Microphyllum (Labiatae)'. Biochemical Systematics and Ecology 30 (9): 865–79. https://doi.org/10.1016/S0305-1978(02)00025-X.

Govindarajan, Marimuthu, Shine Kadaikunnan, Naiyf S. Alharbi, et Giovanni Benelli. 2016. « Acute Toxicity and Repellent Activity of the Origanum Scabrum Boiss. & Heldr. (Lamiaceae) Essential Oil against Four Mosquito Vectors of Public Health Importance and Its Biosafety on Non-Target Aquatic Organisms ». Environmental Science and Pollution Research 23 (22): 23228-38. https://doi.org/10.1007/s11356-016-7568-2.

Greuter, Werner, and Thomas Raus. 1986. 'Med-Checklist Notulae, 13'. Willdenowia 16 (1): 103–16. JSTOR.

Grigoriadou, K., N. Krigas, V. Sarropoulou, K. Papanastasi, G. Tsoktouridis, and E. Maloupa. 2019. 'In Vitro Propagation of Medicinal and Aromatic Plants: The Case of Selected Greek Species with Conservation Priority'. In Vitro Cellular & Developmental Biology - Plant 55 (6): 635–46. https://doi.org/10.1007/s11627-019-10014-6.

Harvala, C., P. Menounos, and N. Argyriadou. 1987. 'Essential Oil from Origanum Dictamnus'. Planta Medica 53 (01): 107–9. https://doi.org/10.1055/s-2006-962640.

Ietswaar, J H. 1972. « Delimitation of Origanum Scabrum Boiss. et Heldr. (Labiatae) by Means of Morphological Criteria », 9.

Ietswaart, J.H. 1980. 'A Taxonomic Revision of the Genus Origanum (Labiatae)'. http://citeseerx.ist.psu.edu/viewdoc/download?doi=10.1.1.880.8396&rep=rep1&type=pdf.

Ietswaart, J.H. 1980. « A taxonomic revision of the genus origanum (Labiatae) ». 1980. http://citeseerx.ist.psu.edu/viewdoc/download?doi=10.1.1.880.8396&rep=rep1&type=pdf.

Ifantis, T. M., S. Solujić, et H. Skaltsa. 2012. « Secondary Metabolites from the Aerial Parts of Origanum Scabrum Boiss. & Heldr. » Biochemical Systematics and Ecology 44: 289-94.

Kogiannou, Dimitra A. A., Nick Kalogeropoulos, Panagiotis Kefalas, Moschos G. Polissiou, and Andriana C. Kaliora. 2013. 'Herbal Infusions; Their Phenolic Profile, Antioxidant and Anti-Inflammatory Effects in HT29 and PC3 Cells'. Food and Chemical Toxicology, Mechanisms involved in oxidative stress regulation, vol. 61 (November): 152–59. https://doi.org/10.1016/j.fct.2013.05.027.

Köngül Şafak, Esra, Gökçe Şeker Karatoprak, Tuncay Dirmenci, Hayri Duman, and Nurgün Küçükboyacı. 2022. 'Cytotoxic Effects of Some Nepeta Species against Breast Cancer Cell Lines and Their Associated Phytochemical Properties'. Plants 11 (11): 11. https://doi.org/10.3390/plants11111427.

Kontaxakis, Emmanouil, Emmanouela Filippidi, Andriana Stavropoulou, Dimitra Daferera, Petros A. Tarantilis, and Dimitris Lydakis. 2020. 'Evaluation of Eight Essential Oils for Postharvest Control of Aspergillus Carbonarius in Grapes'. Journal of Food Protection 83 (9): 1632–40. https://doi.org/10.4315/JFP-19-582.

Kouremenos, Anna. 2020. Origanum Dictamnus (Dittany of Crete): Testaments, Uses, and Trade of a Sacred Plant in Antiquity. 26.

Kouri, Georgia, Dimitrios Tsimogiannis, Haido Bardouki, and Vassiliki Oreopoulou. 2007. 'Extraction and Analysis of Antioxidant Components from Origanum Dictamnus'. Innovative Food Science & Emerging Technologies 8 (2): 155–62. https://doi.org/10.1016/j.ifset.2006.09.003.

Krigas, Nikos, Diamanto Lazari, Eleni Maloupa, and Maria Stikoudi. 2015. 'Introducing Dittany of Crete (Origanum Dictamnus L.) to Gastronomy: A New Culinary Concept for a Traditionally Used Medicinal Plant'. International Journal of Gastronomy and Food Science 2 (2): 112–18. https://doi.org/10.1016/j.ijgfs.2015.02.001.

Krigas, Nikos, Georgios Tsoktouridis, Ioannis Anestis, et al. 2021. 'Exploring the Potential of Neglected Local Endemic Plants of Three Mediterranean Regions in the Ornamental Sector: Value Chain Feasibility and Readiness Timescale for Their Sustainable Exploitation'. *Sustainability* 13 (5): 5. https://doi.org/10.3390/su13052539.

Letsiou, Sophia, Maria Trapali, Despina Vougiouklaki, Aliki Tsakni, Dionysis Antonopoulos, and Dimitra Houhoula. 2023. 'Antioxidant Profile of Origanum Dictamnus L. Exhibits Antiaging Properties against UVA Irradiation'. Cosmetics 10 (5): 5. https://doi.org/10.3390/cosmetics10050124.

Libiad, Mohamed, Abdelmajid Khabbach, Mohamed El Haissoufi, et al. 2021. 'Agro-Alimentary Potential of the Neglected and Underutilized Local Endemic Plants of Crete (Greece), Rif-Mediterranean Coast of Morocco and Tunisia: Perspectives and Challenges'. Plants 10 (9): 9. https://doi.org/10.3390/plants10091770.

Liolios, C. C., O. Gortzi, S. Lalas, J. Tsaknis, and I. Chinou. 2009. 'Liposomal Incorporation of Carvacrol and Thymol Isolated from the Essential Oil of Origanum Dictamnus L. and in Vitro Antimicrobial Activity'. Food Chemistry 112 (1): 77–83. https://doi.org/10.1016/j.foodchem.2008.05.060.

Lionis, Christos, Elena Petelos, Manolis Linardakis, et al. 2023. 'A Mixture of Essential Oils from Three Cretan Aromatic Plants Inhibits SARS-CoV-2 Proliferation: A Proof-of-Concept Intervention Study in Ambulatory Patients'. Diseases 11 (3): 3. https://doi.org/10.3390/diseases11030105.

Menteli, Viktoria, Nikos Krigas, Manolis Avramakis, Nicholas Turland, and Despoina Vokou. 2019. 'Endemic Plants of Crete in Electronic Trade and Wildlife Tourism: Current Patterns and Implications for Conservation'. Journal of Biological Research-Thessaloniki 26 (1): 10. https://doi.org/10.1186/s40709-019-0104-z.

Mitropoulou, Gregoria, Eleni Fitsiou, Elisavet Stavropoulou, et al. 2015. 'Composition, Antimicrobial, Antioxidant, and Antiproliferative Activity of Origanum Dictamnus (Dittany) Essential Oil'. Microbial Ecology in Health and Disease 26 (1): 26543. https://doi.org/10.3402/mehd.v26.26543.

Møller, Jens K. S, Helle Lindberg Madsen, Tuula Aaltonen, and Leif H Skibsted. 1999. 'Dittany (Origanum Dictamnus) as a Source of Water-Extractable Antioxidants'. Food Chemistry 64 (2): 215–19. https://doi.org/10.1016/S0308-8146(98)00143-5.

Nikolova, Milena, Anatoli Dzhurmanski, and Strahil Berkov. 2021. 'Exudate Compounds of <Em>Origanum </Em>Species [†]'; Proceedings of 1st International Electronic Conference on Biological Diversity, Ecology and Evolution, March 12, 9408. https://doi.org/10.3390/BDEE2021-09408.

Novak, Johannes, Christina Bitsch, Jan Langbehn, et al. 2000. 'Ratios of Cis- and Trans-Sabinene Hydrate in Origanum Majorana L. and Origanum Microphyllum (Bentham) Vogel'. Biochemical Systematics and Ecology 28 (7): 697–704. https://doi.org/10.1016/S0305-1978(99)00098-8.

Paloukopoulou, Charikleia, Christina Tsadila, Sofia Govari, Athina Soulioti, Dimitris Mossialos, and Anastasia Karioti. 2023. 'Extensive Analysis of the Cultivated Medicinal Herbal Drug Origanum Dictamnus L. and Antimicrobial Activity of Its Constituents'. Phytochemistry 208 (April): 113591. https://doi.org/10.1016/j.phytochem.2023.113591.

Paloukopoulou, Charikleia, Sofia Govari, Athina Soulioti, et al. 2021. 'Phenols from Origanum Dictamnus L. and Thymus Vulgaris L. and Their Activity against Malassezia Globosa Carbonic Anhydrase'. Natural Product Research 0 (0): 1–7. https://doi.org/10.1080/14786419.2021.1880406.

Papaioannou, Charikleia, Konstantina Zeliou, Panayiotis Trigas, and Vasileios Papasotiropoulos. 2020. 'High Resolution Melting (HRM) Genotyping in the Genus Origanum: Molecular Identification and Discrimination for Authentication Purposes'. Biochemical Genetics 58 (5): 725–37. https://doi.org/10.1007/s10528-020-09970-1.

Papaioannou, Charikleia, Konstantina Zeliou, Panayiotis Trigas, et Vasileios Papasotiropoulos. 2020. « High Resolution Melting (HRM) Genotyping in the Genus Origanum: Molecular Identification and Discrimination for Authentication Purposes ». Biochemical Genetics 58 (5): 725-37. https://doi.org/10.1007/s10528-020-09970-1.

Pirintsos, S. A., M. Bariotakis, M. Kampa, G. Sourvinos, C. Lionis, and E. Castanas. 2020. 'The Therapeutic Potential of the Essential Oil of Thymbra Capitata (L.) Cav., Origanum Dictamnus L. and Salvia Fruticosa Mill. And a Case of Plant-Based Pharmaceutical Development'. Frontiers in Pharmacology 11. https://doi.org/10.3389/fphar.2020.522213.

Racanicci, Aline M. C., Bente Danielsen, José Fernando M. Menten, Marisa A. B. Regitano-d'Arce, and Leif H. Skibsted. 2004. 'Antioxidant Effect of Dittany (Origanum Dictamnus) in Pre-Cooked Chicken Meat Balls during Chill-Storage in Comparison to Rosemary (Rosmarinus Officinalis)'. European Food Research and Technology 218 (6): 521–24. https://doi.org/10.1007/s00217-004-0907-4.

Sarropoulou, Virginia, Charikleia Paloukopoulou, Anastasia Karioti, Eleni Maloupa, and Katerina Grigoriadou. 2023. 'Rosmarinic Acid Production from Origanum Dictamnus L. Root Liquid Cultures In Vitro'. Plants 12 (2): 2. https://doi.org/10.3390/plants12020299.

Shooraj, Mahdi, Fatemeh Ramezan Yazdi, and Seif Ali Mahdavi. 2022. 'A Review of the Effects of Herbal Medicines on Leishmaniasis'. Tabari Biomedical Student Research Journal, ahead of print, June 15. https://doi.org/10.18502/tbsrj.v4i2.9665.

Siakavella, Ioanna K., Fotini Lamari, Dimitrios Papoulis, et al. 2020. 'Effect of Plant Extracts on the Characteristics of Silver Nanoparticles for Topical Application'. Pharmaceutics 12 (12): 12. https://doi.org/10.3390/pharmaceutics12121244.

Sivropoulou, Afroditi, Eleni Papanikolaou, Constantina Nikolaou, Stella Kokkini, Thomas Lanaras, and Minas Arsenakis. 1996. 'Antimicrobial and Cytotoxic Activities of Origanum Essential Oils'.

Journal of Agricultural and Food Chemistry 44 (5): 1202–5. https://doi.org/10.1021/jf950540t.

Skoula, Melpomene, Renée J. Grayer, Geoffrey C. Kite, and Nigel C. Veitch. 2008. 'Exudate Flavones and Flavanones in Origanum Species and Their Interspecific Variation'. Biochemical Systematics and Ecology 36 (8): 646–54. https://doi.org/10.1016/j.bse.2008.05.003.

Skoula, Melpomeni, Panagiota Gotsiou, George Naxakis, and Christopher B. Johnson. 1999. 'A Chemosystematic Investigation on the Mono- and Sesquiterpenoids in the Genus Origanum (Labiatae)'. Phytochemistry 52 (4): 649–57. https://doi.org/10.1016/S0031-9422(99)00268-X.

Stavropoulou, Andriana, Kostas Loulakakis, Naresh Magan, and Nikos Tzortzakis. 2014. 'Origanum Dictamnus Oil Vapour Suppresses the Development of Grey Mould in Eggplant Fruit In Vitro'. Research article. BioMed Research International. https://doi.org/10.1155/2014/562679.

Tomás-Barberán, Franclsco A., Syed Z. Husain, and Maria I. Gil. 1988. 'The Distribution of Methylated Flavones in the Lamiaceae'. Biochemical Systematics and Ecology 16 (1): 43–46. https://doi.org/10.1016/0305-1978(88)90115-9.

Tsakni, Aliki, Archontoula Chatzilazarou, Efstathia Tsakali, Andreas G. Tsantes, Jan Van Impe, and Dimitra Houhoula. 2023. 'Identification of Bioactive Compounds in Plant Extracts of Greek Flora and Their Antimicrobial and Antioxidant Activity'. Separations 10 (7): 7. https://doi.org/10.3390/separations10070373.

Tseliou, Melpomeni, Stergios A. Pirintsos, Christos Lionis, Elias Castanas, and George Sourvinos. 2019. 'Antiviral Effect of an Essential Oil Combination Derived from Three Aromatic Plants (Coridothymus Capitatus (L.) Rchb. f., Origanum Dictamnus L. and Salvia Fruticosa Mill.) against Viruses Causing Infections of the Upper Respiratory Tract'. Journal of Herbal Medicine 17–18 (September): 100288. https://doi.org/10.1016/j.hermed.2019.100288.

Tsioutsiou, Efthymia Eleni, Antigoni Cheilari, and Nektarios Aligiannis. 2022. 'Discovery of Bioactive Natural Products with Skin Beneficial Properties through the Exploitation of Ethnobotanical Studies Conducted in the Balkans'. Macedonian Pharmaceutical Bulletin 68 (04): 159–60. https://doi.org/10.33320/maced.pharm.bull.2022.68.04.072.

Γούτη, Μαρία. 2021. Λειτουργικά τρόφιμα, βιοδραστικές ουσίες, φαρμακευτική και αντιοξειδωτική δράση του origanum dictamnus - Functional foods, bioactive compounds, pharmaceutical and antioxidant activity of origanum dictamnus. March 3. https://doi.org/10.26265/polynoe-328.

Τασούλα, Λαμπρινή. 2020. 'Διερεύνηση της ανάπτυξης, καθώς και ανατομικο-φυσιολογικών παραμέτρων ξηροφυτικών αρωματικών - φαρμακευτικών ειδών της μεσογειακής χλωρίδας, υπό την επίδραση υδατικής καταπόνησης και διαφορετικών ειδών υποστρώματος, σε αστικό φυτοδώμα - Investigation of growth, as well as anatomic-physiological parameters of aromatic xerophytes - medicinal species of the mediterranean flora, under water stress and different substrate types, on an urban green roof'. Διδακτορική Διατριβή, Γεωπονικό Πανεπιστήμιο Αθηνών. Σχολή Επιστημών των Φυτών. Τμήμα Επιστήμης Φυτικής Παραγωγής. Τομέας Κηπευτικών Καλλιεργειών Ανθοκομίας και Αρχιτεκτονικής Τοπίου. Εργαστήριο Ανθοκομίας και Αρχιτεκτονικής Τοπίου. http://hdl.handle.net/10442/hedi/46974.

Кустова, О. К., and А. З. Глухов. 2019. 'Интродукция Малораспространенных Ароматических Растений В Донецком Ботаническом Саду - Introduction of uncommon aromatic plants in the Donetsk Botanical Garden'. Промышленная Ботаника 19 (3). https://www.elibrary.ru/item.asp?id=41209476.

Phlomis

Aligiannis, N., E. Kalpoutzakis, I. Kyriakopoulou, S. Mitaku, et I. B. Chinou. 2004. « Essential Oils OfPhlomis Species Growing in Greece: Chemical Composition and Antimicrobial Activity ». *Flavour and Fragrance Journal* 19 (4): 320-24. https://doi.org/10.1002/ffj.1305.

Basta, Andriani, Olga Tzakou, et Maria Couladis. 2006. « The Essential Oil Composition OfPhlomis Cretica C. Presl ». *Flavour and Fragrance Journal* 21 (5): 795-97. https://doi.org/10.1002/ffj.1717.

Couladis, Maria, Andromachi Tanimanidis, Olga Tzakou, Ioanna B. Chinou, and Catherine Harvala. 2000. 'Essential Oil of Phlomis Lanata Growing in Greece: Chemical Composition and Antimicrobial Activity'. Planta Medica 66 (7): 670–72. https://doi.org/10.1055/s-2000-8631.

Couladis, Maria, Olga Tzakou, Evmorfia Verykokidou, and Catherine Harvala. 2003. 'Screening of Some Greek Aromatic Plants for Antioxidant Activity'. Phytotherapy Research 17 (2): 194–95. https://doi.org/10.1002/ptr.1261.

Georgescu, Luciana, Michalis K. Stefanakis, Stella Kokkini, Haralambos E. Katerinopoulos, and Stergios A. Pirintsos. 2016. 'Chemical and Genetic Characterization of Phlomis Species and Wild Hybrids in Crete'. Phytochemistry 122 (February): 91–102. https://doi.org/10.1016/j.phytochem.2015.11.007.

Kalpoutzakis, Eleftherios, Theodoros Chatzimitakos, Vassilis Athanasiadis, et al. 2023. 'Determination of the Total Phenolics Content and Antioxidant Activity of Extracts from Parts of Plants from the Greek Island of Crete'. Plants 12 (5): 5. https://doi.org/10.3390/plants12051092.

Karali, Debora, Luciana Georgescu, Stergios Pirintsos, and Irene Athanassakis. 2016. 'T Cell Regulation by Phlomis Lanata Protein Extracts in Mice'. Pharmaceutical Biology 54 (2): 207–14. https://doi.org/10.3109/13880209.2015.1027780.

Kontonasaki, Efpraxia, and Ευπραξία Κοντονασάκη. 2019. Αυτοφυή και καλλιεργούμενα φυτικά είδη της περιοχής της Βιάννου και οι χρήσεις τους από τους κατοίκους της. May 23. https://apothesis.lib.hmu.gr/handle/20.500.12688/9120.

Menteli, Viktoria, Nikos Krigas, Manolis Avramakis, Nicholas Turland, and Despoina Vokou. 2019. 'Endemic Plants of Crete in Electronic Trade and Wildlife Tourism: Current Patterns and Implications for Conservation'. Journal of Biological Research-Thessaloniki 26 (1): 10. https://doi.org/10.1186/s40709-019-0104-z.

Ryding, Olof. 2008. 'Pericarp Structure and Phylogeny of the Phlomis Group (Lamiaceae ...: Ingenta Connect'.

https://www.ingentaconnect.com/content/schweiz/bj/2008/00000127/00000003/art00005.

СУХОРУКОВ, А.П., and М.В. НИЛОВА. 2008. 'Карпологические особенности кустарниковых представителей рода Phlomis L. s. str. (Lamioideae, Labiatae)'. Бюллетень Московского Общества Испытателей Природы. Отдел Биологический 113 (4): 60–68.

Prunella

Egli, Bernhard, Pedro Gerstberger, Werner Greuter, and Horst Risse. 1990. 'Horstrissea Dolinicola, a New Genus and Species of Umbels (Umbelliferae, Apiaceae) from Kriti (Greece)'. *Willdenowia* 19 (2): 389–99. JSTOR.

Lyrintzis, G., and V. Papanastasis. 1995. 'Human Activities and Their Impact on Land Degradation - Psilorites Mountain in Crete: A Historical Perspective'. *Land Degradation and Development* 6 (2): 79–93. https://doi.org/10.1002/ldr.3400060203.

Spanos, Ioannis, Panagiotis Platis, Ioannis Meliadis, and Alexandros Tsiontis. 2008. *A Review on the Ecology and Management of the Samaria Gorge, a Greek Biosphere Reserve*. 16.

Salvia

Evangelia, Samara, Cook Catherine-Margaret, Lanaras Thomas, and Kokkini Stella. 2014. *ΤΑ ΑΙΘΕΡΙΑ ΕΛΑΙΑ ΤΟΥ ΓΕΝΟΥΣ SALVIA: ΥΠΑΡΧΟΥΣΑ ΓΝΩΣΗ ΑΠΟ ΕΛΛΑΔΙΚΑ ΟΙΚΟΣΥΣΤΗΜΑΤΑ*. https://doi.org/10.13140/2.1.4806.9127.

Fraskou, P., E.-M. Tomou, E. Dariotis, N. Krigas, and H. Skaltsa. 2022. 'Composition of the Essential Oils of Ten Salvia Taxa from Greece'. *Planta Medica* 88 (15): P-244. https://doi.org/10.1055/s-0042-1759218.

Nikolova, Milena, and Ina Aneva. 2017. 'European Species of Genus Salvia: Distribution, Chemodiversity and Biological Activity'. In *Salvia Biotechnology*, edited by Vasil Georgiev and Atanas Pavlov. Springer International Publishing. https://doi.org/10.1007/978-3-319-73900-7_1.

Petrakou, Kassiani, Gregoris Iatrou, and Fotini N. Lamari. 2020. 'Ethnopharmacological Survey of Medicinal Plants Traded in Herbal Markets in the Peloponnisos, Greece'. *Journal of Herbal Medicine* 19 (February): 100305. https://doi.org/10.1016/j.hermed.2019.100305.

Tomou, Ekaterina-Michaela, Panagiota Fraskou, Konstantina Dimakopoulou, Eleftherios Dariotis, Nikos Krigas, and Helen Skaltsa. 2024. 'Chemometric Analysis Evidencing the Variability in the Composition of Essential Oils in 10 Salvia Species from Different Taxonomic Sections or Phylogenetic Clades'. *Molecules* 29 (7): 7. https://doi.org/10.3390/molecules29071547.

Satureja

Askun, Tulin, Gulendam Tumen, Fatih Satil, and Didem Karaarslan. 2012. 'Active Constituents of Some Satureja L. Species and Their Biological Activities'. *African Journal of Microbiology Research* 6 (22). https://doi.org/10.5897/AJMR11.012.

Azaz, Dilek, Fatih Demirci, Fatih Satıl, Mine Kürkçüoğlu, Kemal Hüsnü, and Can Başerb. 2002. 'Antimicrobial Activity of Some Satureja Essential Oils'. *Zeitschrift Für Naturforschung C* 57 (9–10): 817–21. https://doi.org/10.1515/znc-2002-9-1011.

Chasapis, M., D. A. Samaras, K. Theodoropoulos, and E. Eleftheriadou. 2020. 'The Vascular Flora of Mt Tzena (Northern Greece)'. *Flora Mediterranea* 30. https://doi.org/10.7320/FlMedit30.055.

Chorianopoulos, Nikos, Epameinontas Evergetis, Athanasios Mallouchos, Eleftherios Kalpoutzakis, George-John Nychas, et Serkos A. Haroutounian. 2006. « Characterization of the Essential Oil Volatiles of *Satureja Thymbra* and *Satureja Parnassica* : Influence of Harvesting Time and Antimicrobial Activity ». *Journal of Agricultural and Food Chemistry* 54 (8): 3139-45. https://doi.org/10.1021/jf053183n.

Dardioti, A., C. M. Cook, S. Kokkini, and T. Lanaras. 1997. 'Composition of Satureja Horvatii Subsp. Macrophylla Oil Isolated by Hydrodistillation and Micro-Simultaneous Distillation/Extraction'. *Journal of Essential Oil Research* 9 (6): 663–66. https://doi.org/10.1080/10412905.1997.9700807.

Dardioti, Antonia, Effie Hanlidou, Tom Lanaras, and Stella Kokkini. 2010. 'The Essential Oils of the Greek Endemic Satureja Horvatii Ssp. Macrophylla in Relation to Bioclimate'. *Chemistry & Biodiversity* 7 (8): 1968–77. https://doi.org/10.1002/cbdv.200900181.

GBIF. n.d. 'Satureja Montana Subsp. Macedonica (Formánek) Baden'. Accessed 7 May 2019. https://www.gbif.org/species/3904645.

Kamari, Georgia, Dimitrios Phitos, Britt Snogerup, and Sven Snogerup. 1988. 'Flora and Vegetation of Yioura, N Sporades, Greece'. *Willdenowia* 17 (1/2): 59–85. JSTOR.

Kavci, Esra. 2020. *A study of the enzyme inhibition properties and caryologies og some taxa of Satureja L. (Lamiaceae)*. 99.

Maede, M., I. Hamzeh, D. Hossein, A. Majid, et R. K. Reza. 2011. « Bioactivity of Essential Oil from Satureja Hortensis (Laminaceae) against Three Stored-Product Insect Species ». *African Journal of Biotechnology* 10 (34): 6620-27. https://doi.org/10.4314/ajb.v10i34.

Mohammedi, Z. 2017. « Carvacrol: An Update of Biological Activities and Mechanism of Action ». 2017. https://www.sryahwapublications.com/open-access-journal-of-chemistry/pdf/v1-i1/8.pdf.

Niemeyer, H. M. 2010. « Composition of Essential Oils from *Satureja Darwinii* (Benth.) Briq. and *S. Multiflora* (R. et P.) Briq. (Lamiaceae). Relationship Between Chemotype and Oil Yield in *Satureja* Spp. » *Journal of Essential Oil Research* 22 (6): 477-82. https://doi.org/10.1080/10412905.2010.9700376.

Öztürk, Mehmet. 2012. 'Anticholinesterase and Antioxidant Activities of Savoury (Satureja Thymbra L.) with Identified Major Terpenes of the Essential Oil'. *Food Chemistry* 134 (1): 48–54. https://doi.org/10.1016/j.foodchem.2012.02.054.

Papadatou, Marilena, Catherine Argyropoulou, Katerina Grigoriadou, Eleni Maloupa, et Helen Skaltsa. 2015. « Essential oil content of cultivated Satureja spp. in Northern Greece ». *Natural Volatiles and Essential Oils* 2 (1): 37-48.

Pardavella, Iro, Eleni Nasiou, Dimitra Daferera, Panayiotis Trigas, et Ioannis Giannakou. 2020. « The Use of Essential Oil and Hydrosol Extracted from Satureja Hellenica for the Control of Meloidogyne

Incognita and M. Javanica ». *Plants* 9 (7): 856. https://doi.org/10.3390/plants9070856.

Pirini, Chrisoula B., Ioannis Tsiripidis, and Erwin Bergmeier. 2014. 'Steppe-Like Grass Land Vegetation in the Hills around the Lakes of Vegoritida and Petron, North-Central Greece'. *Hacquetia* 13 (1): 121–69. https://doi.org/10.2478/hacq-2014-0002.

Reis, Aline Chaves, Isaac Filipe Moreira Konig, Danúbia Aparecida de Carvalho Selvati Rezende, Raquel Romano Palmeira Gonçalves, Allan da Silva Lunguinho, Jenifer Caroline Silva Ribeiro, Maria das Graças Cardoso, et Rafael Neodini Remedio. 2021. « Cytotoxic Effects of Satureja Montana L. Essential Oil on Oocytes of Engorged Rhipicephalus Microplus Female Ticks (Acari: Ixodidae) ». *Microscopy Research and Technique* 84 (7): 1375-88. https://doi.org/10.1002/jemt.23693.

Shariat, Anahita, Ghasem Karimzadeh, and Mohammad Hassan Assareh. 2013. 'Karyology of Iranian Endemic Satureja (Lamiaceae) Species'. *CYTOLOGIA* 78 (3): 305–12. https://doi.org/10.1508/cytologia.78.305.

Stefanaki, Anastasia, Cüneyt Aki, Konstantinos Vlachonasios, et Stella Kokkini. 2010. « PHYTOGEOGRAPHIC VERSUS POLITICAL BORDERS: EUROPEAN UNION'S LIFELONG LEARNING PROGRAMME TOWARDS A COMMON CONCEPT IN THE EAST AEGEAN (E. GREECE, W. TURKEY) ». *Fresenius Environmental Bulletin* 19 (4): 9.

Tsioutsiou, Efthymia Eleni, Paolo Giordani, Effie Hanlidou, Marco Biagi, Vincenzo De Feo, and Laura Cornara. 2019. 'Ethnobotanical Study of Medicinal Plants Used in Central Macedonia, Greece'. Research article. Evidence-Based Complementary and Alternative Medicine. https://doi.org/10.1155/2019/4513792.

Tümen, G., N. Kirimer, N. Ermin, and K. H. C. Baser. 1998. 'The Essential Oils of Two New Satureja Species from Turkey: Satureja Pilosa and S. Icarica'. *Journal of Essential Oil Research* 10 (5): 524–26. https://doi.org/10.1080/10412905.1998.9700959.

Tümen, Gülendam, Fatih Satil, Hayri Duman, and Kemal Hüshnü Can Baser. 2000. 'Two New Records for Turkey: Satureja Icarica P.H. Davis, Satureja Pilosa Velen'. http://journals.tubitak.gov.tr/botany/issues/bot-00-24-3/bot-24-3-7-9903-2.pdf.

Tzakou, Olga, et Helen Skaltsa. 2003. « Composition and Antibacterial Activity of the Essential Oil of Satureja Parnassica Subsp. Parnassica ». *Planta Medica* 69 (3): 282-84. https://doi.org/10.1055/s-2003-38487.

Yfanti, Paraskevi, Anna Batistatou, Georgios Manos, and Marilena E. Lekka. 2015. 'The Aromatic Plant Satureja Horvatii Ssp. Macrophylla Induces Apoptosis and Cell Death to the A549 Cancer Cell Line'. *American Journal of Plant Sciences* 06 (13): 13. https://doi.org/10.4236/ajps.2015.613210.

Yfanti, Paraskevi, George Patakioutas, Dimitra Douma, and Marilena E. Lekka. 2021. 'In Vitro Antifungal Activity of Satureja Horvatii Ssp. Macrophylla Against 3 Tomato Phytopathogenic Fungi'. *Natural Product Communications* 16 (7): 1934578X211025165. https://doi.org/10.1177/1934578X211025165.

Παρδαβέλλα, Ηρώ. 2019. « Μελέτη των φυτικών εκχυλισμάτων των Satureja hellenica, Nigella sativa και Cuminum cyminum ως προς τη δράση τους εναντίον δύο ειδών κομβονηματωδών (Meloidogyne javanica, M. incognita) και ως προς τη χημική τους σύσταση », février. http://dspace.aua.gr/xmlui/handle/10329/6826.

Scutellaria

Aplada, E., Georgiadis, Th., Tiniakou, A., et Theocharopoulos, M. 2007. « PHYTOGEOGRAPHY AND ECOLOGICAL EVALUATION OF THE FLORA AND VEGETATION OF MT PARNITHA (ATTICA, GREECE) ». 2007. https://www.cambridge.org/core/services/aop-cambridge-core/content/view/F22267490E1F16F881A74BF15BC6BE6D/S096042860700087Xa.pdf/phytogeography_and_ecological_evaluation_of_the_flora_and_vegetation_of_mt_parnitha_attica_greece.pdf.

Bothmer, Roland. 2008. 'Differentiation Patterns in the Scutellaria Albida Group (Lamiaceae) in the Aegean Area'. *Nordic Journal of Botany* 5 (5): 421–39. https://doi.org/10.1111/j.1756-1051.1985.tb01672.x.

Bothmer, Roland von. 1987. 'Differentiation Patterns in the E. MediterraneanScutellaria Rubicunda Group (Lamiaceae)'. *Plant Systematics and Evolution* 155 (1–4): 219–49. https://doi.org/10.1007/BF00936301.

Cattaneo, Cristina, and Mauro Grano. 2023. 'Phenotypic Variability in Scutellaria Brevibracteata Subsp. Icarica on the Ikaria Island (Eastern Aegean)'. *Phytologia Balcanica* 29 (1). https://doi.org/10.7546/PhB.29.1.2023.7.

Cole, Michael D., Alan J. Paton, Ray M. Harleyl, and Linda E. Fellows. 1991. 'The Significance of the Iridoid Glycoside, Catalpol, in Scutellaria'. *Biochemical Systematics and Ecology* 19 (4): 333–35. https://doi.org/10.1016/0305-1978(91)90023-S.

Gousiadou, Chrysoula, Charlotte Held Gotfredsen, Søren Rosendal Jensen, and Michail Tsoukalas. 2012. 'Iridoids from Scutellaria Goulimyi Rech. f., Lamiaceae. Morphological and Chemical Relations with Scutellaria Albida L. Ssp Albida'. *Biochemical Systematics and Ecology* 43 (August): 139–41. https://doi.org/10.1016/j.bse.2012.03.004.

Greuter, Werner, and Thomas Raus. 1985. 'Med-Checklist Notulae, 10'. *Willdenowia* 14 (2): 299–308. JSTOR.

Grigoriadou, Katerina, Nikos Krigas, Virginia Sarropoulou, Eleni Maloupa, and Georgios Tsoktouridis. 2021. 'Propagation and Ex-Situ Conservation of Lomelosia Minoana Subsp. Minoana and Scutellaria Hirta - Two Ornamental and Medicinal Cretan Endemics (Greece)'. *Notulae Botanicae Horti Agrobotanici Cluj-Napoca* 49 (1): 1. https://doi.org/10.15835/nbha49112168.

Iliadou, Eleni, Ioannis Bazos, Konstantinos Kougioumoutzis, et al. 2020. 'Taxonomic and Phylogenetic Diversity Patterns in the Northern Sporades Islets Complex (West Aegean, Greece)'. *Plant Systematics and Evolution* 306 (2): 28. https://doi.org/10.1007/s00606-020-01660-0.

Kazakis, George, Dany Ghosn, Ilektra Remoundou, Panagiotis Nyktas, Michael A. Talias, and Ioannis N. Vogiatzakis. 2021. 'Altitudinal Vascular Plant Richness and Climate Change in the Alpine Zone of the Lefka Ori, Crete'. *Diversity* 13 (1): 1. https://doi.org/10.3390/d13010022.

Krigas, Nikos, Marina Panagiotidou, and Eleni Maloupa. 2017. ‘Incorporating Biogeographical Principles in Horticulture: Design and Creation of the Ionian Islands Unique Rock Garden in Thessaloniki, Greece’. *Sibbaldia: The Journal of Botanic Garden Horticulture* 0 (15): 129–46.

Mathiesen, C., A.-C. Scheen, and C. Lindqvist. 2011. ‘Phylogeny and Biogeography of the Lamioid Genus Phlomis (Lamiaceae)’. *Kew Bulletin* 66 (1): 83–99. https://doi.org/10.1007/s12225-011-9257-0.

Milla, S., G. Danalatos, E. Matiatou, and Y. Samaras. 2015. *In Vitro Propagation of the Endemic Plant Scutellaria Rupestris (Boiss & Heldr) Subsp. Cephalonica (Bornm)*. https://doi.org/10.13140/RG.2.1.4376.1367.

Phitos, D., T. Constantinidis, and G. Kamari. 2009. *Red data book of rare and threatened plants of Greece*. II. Hellenic Botanical Society. http://www.hbs.gr/images/files/publications/RDB2009-vB.pdf.

Ranjbar, Massoud, and Chonour Mahmoudi. 2013. ‘Chromosome Numbers and Biogeography of the Genus Scutellaria L. (Lamiaceae)’. *Caryologia* 66 (3): 205–14. https://doi.org/10.1080/00087114.2013.821840.

Rosselli, Sergio, Antonella Maggio, Franco Piozzi, Monique S. J. Simmonds, and Maurizio Bruno. 2004. ‘Extremely Potent Antifeedant Neo-Clerodane Derivatives of Scutecyprol A’. *Journal of Agricultural and Food Chemistry* 52 (26): 7867–71. https://doi.org/10.1021/jf048532c.

Roy, Tilottama, Nathan S. Catlin, Drake M. G. Garner, Philip D. Cantino, Anne-Cathrine Scheen, and Charlotte Lindqvist. 2016. ‘Evolutionary Relationships within the Lamioid Tribe Synandreae (Lamiaceae) Based on Multiple Low-Copy Nuclear Loci’. *PeerJ* 4 (July): e2220. https://doi.org/10.7717/peerj.2220.

Siljak-Yakovlev, Sonja, Perla Farhat, Nicolas Valentin, Pepy Bareka, and Georgia Kamari. 2019. *New Estimates of Nuclear DNA Amount for 25 Taxa from Kefallinia Island*. 23.

Skaltsa, Helen D., Diamanto M. Lazari, Panayiotis Kyriazopoulos, et al. 2005. ‘Composition and Antimicrobial Activity of the Essential Oils of *Scutellaria Sieberia* Benth. and *Scutellaria Rupestris* Boiss. et Heldr. Ssp. *Adenotricha* (Boiss. et Heldr.) Greuter et Burdet from Greece’. *Journal of Essential Oil Research* 17 (2): 232–35. https://doi.org/10.1080/10412905.2005.9698886.

Strid, Arne. 2015. *Chromosome Numbers of Angiosperms from the Aegean Islands*. 50.

Xanthakis, M., M. Panagiotis, G. Lysitsa, and G. Kamari. 2015. ‘Study of Vertical and Horizontal Forest Structure in Mt. Ainos National Park, Cephalonia Island, Greece’. *Flora Mediterranea* 25 (Special Issue). https://doi.org/10.7320/FlMedit25SI.245.

Sideritis

Aligiannis, N., E. Kalpoutzakis, I. B. Chinou, S. Mitakou, E. Gikas, and A. Tsarbopoulos. 2001. ‘Composition and Antimicrobial Activity of the Essential Oils of Five Taxa of *Sideritis* from Greece’. *Journal of Agricultural and Food Chemistry* 49 (2): 811–15. https://doi.org/10.1021/jf001018w.

Armata, M., C. Gabrieli, A. Termentzi, M. Zervou, and E. Kokkalou. 2008. ‘Constituents of Sideritis Syriaca. Ssp. Syriaca (Lamiaceae) and Their Antioxidant Activity’. *Food Chemistry* 111 (1): 179–86. https://doi.org/10.1016/j.foodchem.2008.03.061.

Bertsouklis, Konstantinos, Panagiota Theodorou, and Paraskevi-Evangelia Aretaki. 2022. ‘In Vitro Propagation of the Mount Parnitha Endangered Species Sideritis Raeseri Subsp. Attica’. *Horticulturae* 8 (12): 12. https://doi.org/10.3390/horticulturae8121114.

Dontas, IA, PP Lelovas, SK Kourkoulis, et al. 2011. ‘Protective Effect of Sideritis Euboea Extract on Bone Mineral Density and Strength of Ovariectomized Rats.’ *Menopause (New York, N.Y.)* 18 (8): 915–22.

Eva Kassi, †, † Zoi Papoutsi, ‡ Nikolaos Fokialakis, § Ioanna Messari, ‡ and Sophia Mitakou, and † Paraskevi Moutsatsou*. 2004. ‘Greek Plant Extracts Exhibit Selective Estrogen Receptor Modulator (SERM)-like Properties’. Research-article. October 16. https://doi.org/10.1021/jf0400765.

Hofrichter, Jacqueline, Markus Krohn, Toni Schumacher, et al. 2016. ‘Sideritis Spp. Extracts Enhance Memory and Learning in Alzheimer’s β-Amyloidosis Mouse Models and Aged C57Bl/6 Mice’. *Journal of Alzheimer’s Disease* 53 (3): 967–80. https://doi.org/10.3233/JAD-160301.

Karousou, R., A. M. Bosabalidis, and S. Kokkini. 1992. ‘Sideritis Syriaca Ssp. Syriaca: Glandular Trichome Structure and Development in Relation to Systematics’. *Nordic Journal of Botany* 12 (1): 31–37. https://doi.org/10.1111/j.1756-1051.1992.tb00198.x.

Kassi, Eva, Cleanthi Dimas, Marianna Dalamaga, et al. 2013. ‘Sideritis Euboea Extract Lowers Total Cholesterol but Not LDL Cholesterol in Humans: A Randomized Controlled Trial’. *Clinical Lipidology* 8 (6): 627–34. https://doi.org/10.2217/clp.13.64.

Kassi, Eva, Anna Paliogianni, Ismene Dontas, et al. 2011. ‘Effects of Sideritis Euboea (Lamiaceae) Aqueous Extract on IL-6, OPG and RANKL Secretion by Osteoblasts’. *Natural Product Communications* 6 (11): 1934578X1100601131. https://doi.org/10.1177/1934578X1100601131.

Kloukina, Charalampia, Ekaterina- Michaela Tomou, Nikos Krigas, et al. 2020. ‘Non-Polar Secondary Metabolites and Essential Oil of Ex Situ Propagated and Cultivated Sideritis Syriaca L. Subsp. Syriaca (Lamiaceae) with Consolidated Identity (DNA Barcoding): Towards a Potential New Industrial Crop’. *Industrial Crops and Products* 158 (December): 112957. https://doi.org/10.1016/j.indcrop.2020.112957.

Koedam, Arthur. 1986. « Volatile Oil Composition of Greek Mountain Tea (Sideritis Spp.) ». *Journal of the Science of Food and Agriculture* 37 (7): 681-84. https://doi.org/10.1002/jsfa.2740370712.

Koutsaviti, Aikaterini, Ioannis Bazos, Marina Milenkovi, and Milica Pavlovi. 2013. ‘Antimicrobial Activity and Essential Oil Composition of Five Sideritis Taxa of Empedoclia and Hesiodia Sect. from Greece’. *Rec. Nat. Prod.*, 9.

Krigas, Nikos, Georgios Tsoktouridis, Ioannis Anestis, et al. 2021. ‘Exploring the Potential of Neglected Local Endemic Plants of Three Mediterranean Regions in the Ornamental Sector: Value Chain Feasibility and Readiness Timescale for Their Sustainable

Exploitation'. *Sustainability* 13 (5): 5. https://doi.org/10.3390/su13052539.

Libiad, Mohamed, Abdelmajid Khabbach, Mohamed El Haissoufi, et al. 2021. 'Agro-Alimentary Potential of the Neglected and Underutilized Local Endemic Plants of Crete (Greece), Rif-Mediterranean Coast of Morocco and Tunisia: Perspectives and Challenges'. *Plants* 10 (9): 9. https://doi.org/10.3390/plants10091770.

Linardaki, Z., M. A. Papandreou, G. Iatrou, F. N. Lamari, et M. Margarity. 2008. « Antioxidant Response of Brain Regions in Adult Mice after Daily Consumption of Herbal Tea from Sideritis Clandestina ». *Planta Medica* 74 (9): PA240. https://doi.org/10.1055/s-0028-1084238.

Linardaki, Zacharoula I., Catherine G. Vasilopoulou, Caterina Constantinou, Gregoris Iatrou, Fotini N. Lamari, et Marigoula Margarity. 2011. « Differential Antioxidant Effects of Consuming Tea from Sideritis clandestina subsp. peloponnesiaca on Cerebral Regions of Adult Mice ». *Journal of Medicinal Food* 14 (9): 1060-64. https://doi.org/10.1089/jmf.2010.0187.

Margaris, Nikos, Arthur Koedam, and Despina Vokou, eds. 1982. *Aromatic Plants*. Springer Netherlands. https://doi.org/10.1007/978-94-009-7642-9.

Menteli, Viktoria, Nikos Krigas, Manolis Avramakis, Nicholas Turland, and Despoina Vokou. 2019. 'Endemic Plants of Crete in Electronic Trade and Wildlife Tourism: Current Patterns and Implications for Conservation'. *Journal of Biological Research-Thessaloniki* 26 (1): 10. https://doi.org/10.1186/s40709-019-0104-z.

Mitsou, Evdokia K., Katja Turunen, Panagiotis Anapliotis, Dimitra Zisi, Vasilis Spiliotis, and Adamantini Kyriacou. 2009. 'Impact of a Jelly Containing Short-Chain Fructo-Oligosaccharides and Sideritis Euboea Extract on Human Faecal Microbiota'. *International Journal of Food Microbiology* 135 (2): 112–17. https://doi.org/10.1016/j.ijfoodmicro.2009.08.004.

Papaefstathiou, G., P. Polychronopoulos, N. Aligiannis, A. L. Skaltsounis, and S. Mitaku. 2008. 'Comparative Study of Accelerated Solvent Extraction and Supercritical Fluid Extraction of Total Phenolics and Flavonoids from Sideritis Raeseri Subsp. Attica and Study of Antioxidant Activity'. *Planta Medica* 74 (9): PC147. https://doi.org/10.1055/s-0028-1084665.

Pappas, Christos S., Marinos Xagoraris, Athanasios Kimbaris, Georgios Korakis, and Petros A. Tarantilis. 2020. 'Chemometric-Infrared Spectroscopic Model for the Taxonomy of Medicinal Herbs - The Case of Perennial Sideritis Species'. *Biomedical Journal of Scientific & Technical Research* 32 (1). https://doi.org/10.26717/BJSTR.2020.32.005199.

Paschalidis, Konstantinos A., Dimitrios Fanourakis, Georgios Tsaniklidis, et al. 2023. 'Molecular DNA Barcoding and Fertilization Effects on Growth and Herbal Quality Features of Cultivated Sideritis Syriaca Subsp. Syriaca, a Local Endemic Plant of Crete with High Medicinal Value'. No. 2023121763. Preprint, Preprints, December 22. https://doi.org/10.20944/preprints202312.1763.v1.

Plioukas, Michael, Aikaterini Termentzi, Chrysi Gabrieli, Maria Zervou, Panagiotis Kefalas, and Eugene Kokkalou. 2010. 'Novel Acylflavones from Sideritis Syriaca Ssp. Syriaca'. *Food Chemistry* 123 (4): 1136–41. https://doi.org/10.1016/j.foodchem.2010.05.076.

Sarrou, Eirini, Lemonia Doukidou, Evangelia V. Avramidou, et al. 2022. 'Chemodiversity Is Closely Linked to Genetic and Environmental Diversity: Insights into the Endangered Populations of the Local Endemic Plant Sideritis Euboea Heldr. of Evia Island (Greece)'. *Journal of Applied Research on Medicinal and Aromatic Plants* 31 (December): 100426. https://doi.org/10.1016/j.jarmap.2022.100426.

Skouroliakou, Maria, Olympia Kastanidou, Maria Stathopoulou, and Georgia Vourli. 2009. 'Evaluation of the Antioxidant Effect of a New Functional Food Enriched with Sideritis Euboea in Healthy Subjects'. *Journal of Medicinal Food* 12 (5): 1105–10. https://doi.org/10.1089/jmf.2008.0172.

Sokolis, Dimitrios P., Constantinos A. Dimitriou, Pavlos Lelovas, Nikolaos G. Kostomitsopoulos, and Ismene A. Dontas. 2017. 'Effect of Ovariectomy and Sideritis Euboea Extract Administration on Large Artery Mechanics, Morphology, and Structure in Middle-Aged Rats'. *Biorheology* 54 (1): 1–23. https://doi.org/10.3233/BIR-16113.

Stagos, Dimitrios, Nikolaos Portesis, Chryssa Spanou, et al. 2012. 'Correlation of Total Polyphenolic Content with Antioxidant and Antibacterial Activity of 24 Extracts from Greek Domestic Lamiaceae Species'. *Food and Chemical Toxicology* 50 (11): 4115–24. https://doi.org/10.1016/j.fct.2012.08.033.

Thanos, Costas A., and Maria A. Doussi. 1995. 'Ecophysiology of Seed Germination in Endemic Labiates of Crete'. *Israel Journal of Plant Sciences* 43 (3): 227–37. https://doi.org/10.1080/07929978.1995.10676607.

Tomou, Ekaterina-Michaela, Lara Bieler, Tobias Spöttl, Sebastien Couillard-Despres, Helen Skaltsa, and Corinna Urmann. 2023. 'Metabolic Fingerprinting of Different Sideritis Taxa Infusions and Their Neurogenic Activity'. *Planta Medica* 89 (11): 1087–96. https://doi.org/10.1055/a-2072-2351.

Tomou, Ekaterina-Michaela, Maria V. Chatziathanasiadou, Paschalina Chatzopoulou, Andreas G. Tzakos, and Helen Skaltsa. 2020. 'NMR-Based Chemical Profiling, Isolation and Evaluation of the Cytotoxic Potential of the Diterpenoid Siderol from Cultivated Sideritis Euboea Heldr.' *Molecules* 25 (10): 10. https://doi.org/10.3390/molecules25102382.

Tomou, Ekaterina-Michaela, Christina D. Papaemmanouil, Dimitrios A. Diamantis, et al. 2021. 'Anti-Ageing Potential of S. Euboea Heldr. Phenolics'. *Molecules* 26 (11): 11. https://doi.org/10.3390/molecules26113151.

Tsaknis, John, and Stavros Lalas. 2005. 'Extraction and Identification of Natural Antioxidant from Sideritis Euboea (Mountain Tea)'. Research-article. July 7. https://doi.org/10.1021/jf0479261.

Tzakou, Olga. 2002. 'The Essential Oil of *Sideritis Raeseri* Boiss. et Heldr. Ssp. *Attica* (Heldr.) Pap. et Kok.' *Journal of Essential Oil Research* 14 (5): 376–77. https://doi.org/10.1080/10412905.2002.9699891.

Vasiliki, Georgakopoulou, Dimou Charalampia, and Karantonis Christos Haralabos. 2019. 'In Vitro Antioxidant, Antithrombotic, Antiatherogenic and Antidiabetic Activities of Urtica Dioica,

Sideritis Euboea and Cistus Creticus Water Extracts and Investigation of Pasta Fortification with the Most Bioactive One'. *Current Pharmaceutical Biotechnology* 20 (10): 874–80. https://doi.org/10.2174/1389201020666190328114343.

Vasilopoulou, Catherine G., Vassiliki G. Kontogianni, Zacharoula I. Linardaki, Gregoris Iatrou, Fotini N. Lamari, Alexandra A. Nerantzaki, Ioannis P. Gerothanassis, Andreas G. Tzakos, et Marigoula Margarity. 2013. « Phytochemical Composition of "Mountain Tea" from Sideritis Clandestina Subsp. Clandestina and Evaluation of Its Behavioral and Oxidant/Antioxidant Effects on Adult Mice ». *European Journal of Nutrition* 52 (1): 107-16. https://doi.org/10.1007/s00394-011-0292-2.

Venturella, P., and Aurora Bellino. 1977. 'Eubotriol and Eubol, New Diterpenes fromSideritis Euboea'. *Experientia* 33 (10): 1270–71. https://doi.org/10.1007/BF01920125.

Vogiatzakis, I. N., G. H. Griffiths, and A. M. Mannion. 2003. 'Environmental Factors and Vegetation Composition, Lefka Ori Massif, Crete, S. Aegean'. *Global Ecology and Biogeography* 12 (2): 131–46. https://doi.org/10.1046/j.1466-822X.2003.00021.x.

Μπισκίνης, Αντώνιος. 2021. « Απομόνωση και χαρακτηρισμός συστατικών του εκχυλίσματος οξικού αιθυλεστέρα του είδους Sideritis clandestina subsp. peloponnesiaca (Boiss. & Heldr.) Baden - Isolation and characterization of compounds derived from the ethyl acetate extract of the species Sideritis clandestina subsp. peloponnesiaca (Boiss. & Heldr.) Baden ». Master of Science, Patras, Greece: University of Patras. http://nemertes.library.upatras.gr/jspui//handle/10889/15145.

Σιάννη, Μαρίας. 2017. « Μελέτη του μικροπολλαπλασιασμού του Sideritis clandestina subs peloponnesiaca - Study of the micropropagation of Sideritis clandestina subsp. peloponnesiaca ». Kalamata.

Σταυρέλη, Μιχαέλα. 2020. « Χαρακτηρισμός φυσικών προϊόντων του είδους Sideritis clandestina ssp. peloponnesiaca (Boiss. & Heldr.) Baden ως προς τη δομή & τη βιοδραστικότητα - Characterization of natural products from the species Sideritis clandestina ssp. peloponnesiaca (Boiss. & Heldr.) Baden in terms of their structure and bioactivity ». Master of Science, Patras, Greece: University of Patras. http://nemertes.library.upatras.gr/jspui//handle/10889/15108.

Stachys

Afouxenidi, A., T. Milošević-Ifantis, and H. Skaltsa. 2012. 'Secondary Metabolites from the Aerial Parts of Stachys Tetragona, a Greek Endemic Species'. Planta Medica 78 (11): PI360. https://doi.org/10.1055/s-0032-1321047.

Afouxenidi, Aikaterini, Tanja Milošević-Ifantis, and Helen Skaltsa. 2018. 'Secondary Metabolites from Stachys Tetragona Boiss. & Heldr. Ex Boiss. and Their Chemotaxonomic Significance'. Biochemical Systematics and Ecology 81 (December): 83–85. https://doi.org/10.1016/j.bse.2018.09.011.

Aplada, E., Georgiadis, Th., Tiniakou, A., et Theocharopoulos, M. 2007. « PHYTOGEOGRAPHY AND ECOLOGICAL EVALUATION OF THE FLORA AND VEGETATION OF MT PARNITHA (ATTICA, GREECE) ». 2007. https://www.cambridge.org/core/services/aop-cambridge-core/content/view/F22267490E1F16F881A74BF15BC6BE6D/S096042860700087Xa.pdf/phytogeography_and_ecological_evaluation_of_the_flora_and_vegetation_of_mt_parnitha_attica_greece.pdf.

Badalamenti, Natale, Aurora Modica, Vincenzo Ilardi, and Maurizio Bruno. 2023. 'The Chemical Composition of the Aerial Parts Essential Oil of S. Spreitzenhoferi Heldr. (Lamiaceae) Growing in Kythira Island (Greece)'. Natural Product Research 37 (14): 2427–31. https://doi.org/10.1080/14786419.2022.2041008.

Badisa, R. B., O. Tzakou, M. Couladis, and E. Pilarinou. 2003. 'Cytotoxic Activities of Some Greek Labiatae Herbs'. Phytotherapy Research 17 (5): 472–76. https://doi.org/10.1002/ptr.1175.

Baliousis, Evagelos. 2013. « Flora and Vegetation of Mt Likeo (Peloponnisos, Greece) ». Flora Mediterranea 23 (décembre). https://doi.org/10.7320/FlMedit23.015.

Conforti, Filomena, Federica Menichini, Carmen Formisano, Daniela Rigano, Felice Senatore, Nelly Apostolides Arnold, et Franco Piozzi. 2009. « Comparative chemical composition, free radical-scavenging and cytotoxic properties of essential oils of six Stachys species from different regions of the Mediterranean Area ». 2009. https://www.sciencedirect.com/science/article/pii/S0308814609003495.

Constantinidis, Theophanis, Eleftherios Kalpoutzakis, and Konstantinos Kougioumoutzis. 2015. 'The Rediscovery of Stachys Virgata (Lamiaceae), a Rare Endemic of Peloponnisos, Greece: Taxonomy, Distribution, Karyology and Conservation'. Phytotaxa 218 (3): 241. https://doi.org/10.11646/phytotaxa.218.3.3.

Constantinidis, Theophanis, Eleftherios Kalpoutzakis, et Konstantinos Kougioumoutzis. 2015. « The Rediscovery of Stachys Virgata (Lamiaceae), a Rare Endemic of Peloponnisos, Greece: Taxonomy, Distribution, Karyology and Conservation ». Phytotaxa 218 (3): 241. https://doi.org/10.11646/phytotaxa.218.3.3.

Couladis, Maria, Olga Tzakou, Evmorfia Verykokidou, and Catherine Harvala. 2003. 'Screening of Some Greek Aromatic Plants for Antioxidant Activity'. Phytotherapy Research 17 (2): 194–95. https://doi.org/10.1002/ptr.1261.

Fazio, Caterina, Salvatore Passannanti, Maria Pia Paternostro, and Nelly A. Arnold. 1994. 'Diterpenoids from Stachys Mucronata'. Planta Medica 60 (05): 499–499. https://doi.org/10.1055/s-2006-959557.

Fokialakis, N., E. Kalpoutzakis, B. L. Tekwani, S. I. Khan, M. Kobaisy, A. L. Skaltsounis, et S. O. Duke. 2007. « Evaluation of the Antimalarial and Antileishmanial Activity of Plants from the Greek Island of Crete ». Journal of Natural Medicines 61 (1): 38-45. https://doi.org/10.1007/s11418-006-0013-y.

Grigorakis, Spyros, and Dimitris P. Makris. 2018. 'Characterisation of Polyphenol-Containing Extracts from Stachys Mucronata and Evaluation of Their Antiradical Activity'. Medicines 5 (1): 14. https://doi.org/10.3390/medicines5010014.

Kamari, Georgia, Dimitrios Phitos, Britt Snogerup, and Sven Snogerup. 1988. 'Flora and Vegetation of Yioura, N Sporades, Greece'. Willdenowia 17 (1/2): 59–85. JSTOR.

Kamari, Georgia, Pepy Bareka, Theophanis Constantinidis, and Dimitrios Phitos. 2003. 'Karyosystematic Studies of Plant Taxa from the East Mediterranean Region (Greece, Cyprus, Syria)'.

https://www.researchgate.net/profile/Theophanis_Constantinidis/publication/235771062_Karyosystematic_studies_of_plant_taxa_from_the_east_Mediterranean_region_Greece_Cyprus_Syria/links/55f4984208ae1d980394bff5.pdf.

Kotsos, Maria P., Nektarios Aligiannis, et Sofia Mitakou. 2007. « A new flavonoid diglycoside and triterpenoids from Stachys spinosa L. (Lamiaceae) ». Biochemical Systematics and Ecology 35 (6): 381-85. https://doi.org/10.1016/j.bse.2006.11.002.

Kotsos, Maria, Nektarios Aligiannis, Sofia Mitaku, Alxios-Leandros Skaltsounis, et Catherine Charvala. 2001. « Chemistry of Plants from Crete: Stachyspinoside, a New Flavonoid Glycoside and Iroids from Stachys Spinosa », 11.

Koutsaviti, Aikaterini, Marina Milenković, and Olga Tzakou. 2011. 'Antimicrobial Activity of the Essential Oil of Greek Endemic Stachys Spruneri and Its Main Component, Isoabienol'. Natural Product Communications 6 (2): 1934578X1100600. https://doi.org/10.1177/1934578X1100600231.

Lenherr, A., B. Meier, et O. Sticher. 1984. « Modern HPLC as a Tool for Chemotaxonomical Investigations: Iridoid Glucosides and Acetylated Flavonoids in the Group of Stachys Recta 1 ». Planta Medica 50 (05): 403-9. https://doi.org/10.1055/s-2007-969749.

Maloupa, Eleni, and Nikos Krigas. 2008. 'Botanic Garden Profile: The Balkan Botanic Garden of Kroussia, Northern Greece: A Garden Dedicated to the Conservation of the Native Plants of Greece and The Balkans'. Sibbaldia: The Journal of Botanic Garden Horticulture 0 (6): 9–27.

Marhold, Karol. 2006. « IAPT/IOPB Chromosome Data 1 ». Taxon 55 (2): 443-45. https://doi.org/10.2307/25065590.

Marhold, Karol. 2015. 'IAPT/IOPB Chromosome Data 20'. Taxon 64 (6): 1344–50. https://doi.org/10.12705/646.42.

Meremeti, A, A Karioti, H Skaltsa, J Heilmann, and O Sticher. 2004. 'Secondary Metabolites from Stachys Ionica'. Biochemical Systematics and Ecology 32 (2): 139–51. https://doi.org/10.1016/S0305-1978(03)00161-3.

Michailidou, Aikaterini-Maria, Ekaterina-Michaela Tomou, et Helen Skaltsa. 2021. « Phytochemical Study of Stachys Candida Bory & Chaubard (Lamiaceae) ». Biochemical Systematics and Ecology 94 (février): 104208. https://doi.org/10.1016/j.bse.2020.104208.

Panitsa, Maria, Panayiotis Trigas, Dimitrios Kontakos, Anna-Thalassini Valli, et Gregoris Iatrou. 2021. « Natural and cultural heritage interaction: aspects of plant diversity in three East Peloponnesian castles (Greece) and conservation evaluation ». Plant Biosystems - An International Journal Dealing with all Aspects of Plant Biology 0 (0): 1-15. https://doi.org/10.1080/11263504.2021.1889701.

Papadopoulou, Annas. 2017. « Μελέτη του μικροπολλαπλασιασμού του φυτού Stachys candida ». novembre 2017.

Piozzi, F, M.P Paternostro, O Servettaz, and N.A Arnold. 2002. 'Occurrence of (+)-6-Desoxyandalusol in Stachys Ionica and Stachys Distans'. Biochemical Systematics and Ecology 30 (9): 887–89. https://doi.org/10.1016/S0305-1978(02)00022-4.

Piozzi, Franco, and Maurizio Bruno. 2009. 'Diterpenoids in the Essential Oils from the Genus Stachys'. Rec. Nat. Prod., 6.

Piozzi, Franco, and Maurizio Bruno. 2011. 'Diterpenoids from Roots and Aerial Parts of the Genus Stachys'. Rec. Nat. Prod., 11.

Samaropoulou, S, P Bareka, R Artelan, et G Kamari. 2013. « Karyological Studies on Some Endemic and Rare Species of Kefalonia, Ionian Islands, Greece ». Flora Mediterranea 23 (décembre). https://doi.org/10.7320/FlMedit23.215.

Siljak-Yakovlev, Sonja, Perla Farhat, Nicolas Valentin, Pepy Bareka, et Georgia Kamari. 2019. « New Estimates of Nuclear DNA Amount for 25 Taxa from Kefallinia Island », 23.

Siljak-Yakovlev, Sonja, Perla Farhat, Nicolas Valentin, Pepy Bareka, and Georgia Kamari. 2019. New Estimates of Nuclear DNA Amount for 25 Taxa from Kefallinia Island. 23.

Skaltsa, H., P. Georgakopoulos, D. Lazari, A. Karioti, J. Heilmann, O. Sticher, et Th. Constantinidis. 2007. « Flavonoids as chemotaxonomic markers in the polymorphic Stachys swainsonii (Lamiaceae) ». Biochemical Systematics and Ecology 35 (5): 317-20. https://doi.org/10.1016/j.bse.2006.10.014.

Skaltsa, H., P. Georgakopoulos, D. Lazari, et al. 2007. 'Flavonoids as Chemotaxonomic Markers in the Polymorphic Stachys Swainsonii (Lamiaceae)'. Biochemical Systematics and Ecology 35 (5): 317–20. https://doi.org/10.1016/j.bse.2006.10.014.

Skaltsa, Helen D., Costas Demetzos, Diamanto Lazari, and Marina Sokovic. 2003. 'Essential Oil Analysis and Antimicrobial Activity of Eight Stachys Species from Greece'. Phytochemistry 64 (3): 743–52. https://doi.org/10.1016/S0031-9422(03)00386-8.

Skaltsa, Helen, Anna Mavrommati, and Theophanis Constantinidis. 2001. 'A Chemotaxonomic Investigation of Volatile Constituents in Stachys Subsect. Swainsonianeae (Labiatae) - ScienceDirect'. https://www.sciencedirect.com/science/article/pii/S0031942201000036.

Skaltsa, Helen, Anna Mavrommati, et Theophanis Constantinidis. 2001. « A chemotaxonomic investigation of volatile constituents in Stachys subsect. Swainsonianeae (Labiatae) - ScienceDirect ». 2001. https://www.sciencedirect.com/science/article/pii/S0031942201000036.

Skaltsa, Helen, Diamanto Lazari, Ioanna Chinou, et Anargiros Loukis. 1999. « Composition and Antibacterial Activity of the Essential Oils of Stachys Candida and S. Chrysantha from Southern Greece ». Planta Medica 65 (03): 255-56. https://doi.org/10.1055/s-2006-960471.

Skaltsa, Helen, Paulina Bermejo, Diamanto Lazari, Ana Maria Silvan, Alexios-Leandros Skaltsounis, Aurora Sanz, et Maria Jose Abad. 2000. « Inhibition of Prostaglandin E2 and Leukotriene C4 in Mouse Peritoneal Macrophages and Thromboxane B2 Production in Human Platelets by Flavonoids from Stachys Chrysantha and Stachys Candida ». Biological and Pharmaceutical Bulletin 23 (1): 47-53. https://doi.org/10.1248/bpb.23.47.

Stevanovic, V., Kit Tan, and G. Iatrou. 2003. 'Distribution of the Endemic Balkan Flora on Serpentine I. - Obligate Serpentine Endemics'. Plant Systematics and Evolution 242 (1–4): 149–70. https://doi.org/10.1007/s00606-003-0044-8.

Strid, Arne. 2015. Chromosome Numbers of Angiosperms from the Aegean Islands. 50.

Sylvestre, Muriel, André Pichette, Serge Lavoie, Angélique Longtin, et Jean Legault. 2007. « Composition and Cytotoxic Activity of the Leaf Essential Oil OfComptonia Peregrina (L.) Coulter ». Phytotherapy Research 21 (6): 536-40. https://doi.org/10.1002/ptr.2095.

Trigas, Panayiotis, Gregoris Iatrou, and Maria Panitsa. 2008. 'VASCULAR PLANT SPECIES DIVERSITY, BIOGEOGRAPHY AND VULNERABILITY IN THE AEGEAN ISLANDS AS EXEMPLIFIED BY EVVIA ISLAND (W AEGEAN, GREECE)'. Fresenius Environmental Bulletin 17 (1): 10.

КРЕСТОВСКАЯ, Т.В. 2010. 'Критический обзор видов секции Eriostomum Hoffmanns. et Link рода Stachys L. (Labiatae)'. Новости Систематики Высших Растений 42: 198–221.

Крестовская, Т.в. 2019. 'Заметка о Stachys Mucronata (Lamiaceae) и Его Месте в Системе Рода - Taxonomic Note on Stachys Mucronata (Lamiaceae) and Its Position in the Genus System'. Новости Систематики Высших Растений (Россия, Санкт-Петербург) 50: 154–57.

Teucrium

Alcázar, Roberto, Maria Torre (de la), Benjamin Rodriguez, et al. 1992. 'Neo-Clerodane Diterpenoids from Three Species of Teucrium'.

Bareka, P., E. Katopodi, G. Kamari, et D. Phitos. 2018. « Karyosystematic Study of Some Taxa from the Ionian Floristic Region (Greece). I ». Flora Mediterranea 28. https://doi.org/10.7320/FlMedit28.085.

Bellomaria, B., N. Arnold, et G. Valentini. 1998. « Essential Oil of Teucrium Flavum Subsp. Hellenicum from Greece ». Journal of Essential Oil Research 10 (2): 131-33. https://doi.org/10.1080/10412905.1998.9700863.

Bourgou, Soumaya, Imtinen Ben Haj Jilani, Olfa Karous, et al. 2021. 'Medicinal-Cosmetic Potential of the Local Endemic Plants of Crete (Greece), Northern Morocco and Tunisia: Priorities for Conservation and Sustainable Exploitation of Neglected and Underutilized Phytogenetic Resources'. Biology 10 (12): 1344. https://doi.org/10.3390/biology10121344.

Bruno, Maurizio, Gema Domi´nguez, Ana Lourenço, et al. 1991. 'Neo-Clerodane Diterpenoids fromTeucrium Gracile'. Phytochemistry 30 (11): 3693–97. https://doi.org/10.1016/0031-9422(91)80092-F.

Bruno, Maurizio, Roberto Alcázar, María C. de la Torre, et al. 1992. 'Neo- and Seco-Neo-Clerodane Diterpenoids from Teucrium Gracile and T. Fruticans'. Phytochemistry, The International Journal of Plant Biochemistry, vol. 31 (10): 3531–34. https://doi.org/10.1016/0031-9422(92)83722-B.

Chasapis, M., D. A. Samaras, K. Theodoropoulos, and E. Eleftheriadou. 2020. 'The Vascular Flora of Mt Tzena (Northern Greece)'. Flora Mediterranea 30. https://doi.org/10.7320/FlMedit30.055.

Contandriopoulos, J., et P. Quezel. 1976. « Contribution à l'étude de la flore du Taurus et de l'Amanus ». Bulletin de la Société Botanique de France 123 (7-8): 415-32. https://doi.org/10.1080/00378941.1976.10839375.

De Martino, Laura, Raffaele Coppola, Vincenzo De Feo, Lucia Caputo, Florinda Fratianni, and Filomena Nazzaro. 2020. 'Essential Oils Diversity of Teucrium Species'. In Teucrium Species: Biology and Applications, edited by Milan Stanković. Springer International Publishing. https://doi.org/10.1007/978-3-030-52159-2_7.

Diapoulis, Ch. 1959. 'Conservation Measures for the Plants of the Greek Flora.' http://documents.irevues.inist.fr/bitstream/handle/2042/59423/LATERREETLAVIE_1959_Sup_189.pdf?sequence=1.

Gandoger, M. Michel. 1915. 'A la recherche du genre Ammanthus Boiss. Découverte du Bellium minutum L'. Bulletin de la Société Botanique de France 62 (1): 8–12. https://doi.org/10.1080/00378941.1915.10832620.

Gandoger, M. Michel. 1922. 'L'ascension du mont Ida (île de Crète)'. Bulletin de la Société Botanique de France 69 (1): 16–20. https://doi.org/10.1080/00378941.1922.10833394.

Guleryuz, Gurcan, Salih Gucel, et Munir Ozturk. 2010. « Nitrogen Mineralization in a High Altitude Ecosystem in the Mediterranean Phytogeographical Region of Turkey », 12.

Krigas, Nikos, Georgios Tsoktouridis, Ioannis Anestis, et al. 2021. 'Exploring the Potential of Neglected Local Endemic Plants of Three Mediterranean Regions in the Ornamental Sector: Value Chain Feasibility and Readiness Timescale for Their Sustainable Exploitation'. *Sustainability* 13 (5): 5. https://doi.org/10.3390/su13052539.

Krigas, Nikos, Viktoria Menteli, et Despoina Vokou. 2014. « The Electronic Trade in Greek Endemic Plants: Biodiversity, Commercial and Legal Aspects ». Economic Botany 68 (1): 85-95. https://doi.org/10.1007/s12231-014-9264-9.

Kyriakopoulos, Ch., Kamari, G., Kofinas, I. & Phitos, D. 2018. « Potentilla Greuteriana (Rosaceae), a New Species from Mt. Taigetos (S Peloponnisos, Greece) ». Flora Mediterranea 28 (décembre). https://doi.org/10.7320/FlMedit28.351.

Lakušić, Branislava, Branka Stevanović, Radiša Jančić, and Dmitar Lakušić. 2010. 'Habitat-Related Adaptations in Morphology and Anatomy of Teucrium (Lamiaceae) Species from the Balkan Peninsula (Serbia and Montenegro)'. Flora - Morphology, Distribution, Functional Ecology of Plants 205 (10): 633–46. https://doi.org/10.1016/j.flora.2010.04.018.

Lyrintzis, G., and V. Papanastasis. 1995. 'Human Activities and Their Impact on Land Degradation - Psilorites Mountain in Crete: A Historical Perspective'. Land Degradation and Development 6 (2): 79–93. https://doi.org/10.1002/ldr.3400060203.

Maleš, Željan, Kroata Hazler Pilepić, Mirza Bojić, et Zoran Tatalović. 2015. « Determination of the Content of Total Polyphenols, Non-Tannin Polyphenols and Tannins in Five Species of the Genus

Teucrium L. » Periodicum Biologorum 117 (3): 453-55. https://doi.org/10.18054/pb.2015.117.3.1001.

Maloupa, E., D. Zervaki, K. Grigoriadou, et K. Papanastassi. 2003. « The development of native plant collection nursery in the Kroussia Balkan Botanic Garden. » Scripta Botanica Belgica.

Martino, Laura De, Carmen Formisano, Emilia Mancini, et al. 2010. 'Chemical Composition and Phytotoxic Effects of Essential Oils from Four Teucrium Species'. Natural Product Communications 5 (12): 1934578X1000501. https://doi.org/10.1177/1934578X1000501230.

Martino, Laura De, Emilia Mancini, Aurelio Marandino, Luiz Fernando Rolim de Almeida, and Vincenzo De Feo. 2012. 'Chemistry and Antigerminative Activity of Essential Oils and Monoterpenoids from Mediterranean Plants'. Current Bioactive Compounds 8 (1): 13–49. https://doi.org/10.2174/157340712799828179.

Menichini, Federica, Filomena Conforti, Daniela Rigano, Carmen Formisano, Franco Piozzi, and Felice Senatore. 2009. 'Phytochemical Composition, Anti-Inflammatory and Antitumour Activities of Four Teucrium Essential Oils from Greece'. Food Chemistry 115 (2): 679–86. https://doi.org/10.1016/j.foodchem.2008.12.067.

Mohan, Lalit, Charu C. Pant, Anand B. Melkani, and Vasu Dev. 2010. 'Terpenoid Composition of the Essential Oils of Teucrium Royleanum and T. Quadrifarium'. Natural Product Communications 5 (6): 1934578X1000500. https://doi.org/10.1177/1934578X1000500628.

Navarro, Teresa. 2020. 'Systematics and Biogeography of the Genus Teucrium (Lamiaceae)'. In Teucrium Species: Biology and Applications, edited by Milan Stanković. Springer International Publishing. https://doi.org/10.1007/978-3-030-52159-2_1.

Navarro, Teresa. 2020. « Systematics and Biogeography of the Genus Teucrium (Lamiaceae) ». In Teucrium Species: Biology and Applications, édité par Milan Stanković, 1-38. Cham: Springer International Publishing. https://doi.org/10.1007/978-3-030-52159-2_1.

Özcan, Taner, Tuncay Dirmenci, Esra Martin, et Fahim Altınordu. 2015. « Cytotaxonomical study in five taxa of the genus Teucrium L. (Lamiaceae) ». Caryologia 68 (1): 1-8. https://doi.org/10.1080/00087114.2014.996037.

Piozzi, Franco, Maurizio Bruno, Rosaria Ciriminna, Caterina Fazio, Nadia Vassallo, Nelly Arnold, Maria de la Torre, et Benjamin Rodriguez. 1997. « Putative Hepatotoxic Neoclerodane Diterpenoids from Teucrium Species ». Planta Medica 63 (05): 483-84. https://doi.org/10.1055/s-2006-957744.

Piozzi, Franco, Maurizio Bruno, Rosaria Ciriminna, et al. 1997. 'Putative Hepatotoxic Neoclerodane Diterpenoids from Teucrium Species'. Planta Medica 63 (05): 483–84. https://doi.org/10.1055/s-2006-957744.

Presti, Maria Lo, Maria L. Crupi, Rosaria Costa, et al. 2010. 'Seasonal Variations of Teucrium Flavum L. Essential Oil'. Journal of Essential Oil Research 22 (3): 211–16. https://doi.org/10.1080/10412905.2010.9700305.

Presti, Maria Lo, Maria L. Crupi, Rosaria Costa, G. Dugo, L. Mondello, Salvatore Ragusa, et Luca Santi. 2010. « Seasonal Variations of Teucrium Flavum L. Essential Oil ». Journal of Essential Oil Research 22 (3): 211-16. https://doi.org/10.1080/10412905.2010.9700305.

Raab-Straube, Eckhard Von. 2013. 'Euro Med-Checklist Notulae, 2'. Willdenowia 43 (2): 239–49. https://doi.org/10.3372/wi.43.43202.

Schneider, Amy, Michelle Landis, et Jennifer Bousselot. 2021. « Observations on the Survival Capacity of 118 Plant Taxa on a Green Roof in a Semi-Arid Climate: 12 Year Update ». Journal of Living Architecture 8 (1): 19-40. https://doi.org/10.46534/jliv.2021.08.01.019.

Strid, Arne. 2015. Chromosome Numbers of Angiosperms from the Aegean Islands. 50.

Tuzlacı, Ertan, et Pınar Eryaşar Aymaz. 2001. « Turkish folk medicinal plants, Part IV: Gönen (Balıkesir) ». Fitoterapia 72 (4): 323-43. https://doi.org/10.1016/S0367-326X(00)00277-X.

Thymbra

Bräuchler, Christian. 2018. 'Delimitation and Revision of the Genus Thymbra (Lamiaceae)'. *Phytotaxa* 369 (1): 15–27. https://doi.org/10.11646/phytotaxa.369.1.2.

Krigas, Nikos, Georgios Tsoktouridis, Ioannis Anestis, et al. 2021. 'Exploring the Potential of Neglected Local Endemic Plants of Three Mediterranean Regions in the Ornamental Sector: Value Chain Feasibility and Readiness Timescale for Their Sustainable Exploitation'. *Sustainability* 13 (5): 5. https://doi.org/10.3390/su13052539.

Krisilia, Violetta, Georgia Deli, Aikaterini Koutsaviti, and Olga Tzakou. 2020. *Thymbra L. and Satureja L. Essential Oils as Rich Sources of Carvacrol, a Food Additive with Health-Promoting Effects*. November 15, 12.

Libiad, Mohamed, Abdelmajid Khabbach, Mohamed El Haissoufi, et al. 2021. 'Agro-Alimentary Potential of the Neglected and Underutilized Local Endemic Plants of Crete (Greece), Rif-Mediterranean Coast of Morocco and Tunisia: Perspectives and Challenges'. *Plants* 10 (9): 9. https://doi.org/10.3390/plants10091770.

Skoula, Melpomeni, and Renée J. Grayer. 2005. 'Volatile Oils of Coridothymus Capitatus, Satureja Thymbra, Satureja Spinosa andThymbra Calostachya (Lamiaceae) from Crete'. *Flavour and Fragrance Journal* 20 (6): 573–76. https://doi.org/10.1002/ffj.1489.

Skoula, Melpomeni, Renée J. Grayer, and Geoffrey C. Kite. 2004. 'Surface Flavonoids in Coridothymus Capitatus and Thymbra Calostachya (Lamiaceae)'. *Biochemical Systematics and Ecology* 32 (12): 1197–200. https://doi.org/10.1016/j.bse.2004.05.002.

Thymus

Adamović, L. 1907. *Thymus Plasonii Adamov., eine gelblichblühende, neueThymus-Art der Balkanhalbinsel*. 2.

Badisa, R. B., O. Tzakou, M. Couladis, and E. Pilarinou. 2003. 'Cytotoxic Activities of Some Greek Labiatae Herbs'. *Phytotherapy Research* 17 (5): 472–76. https://doi.org/10.1002/ptr.1175.

Bartolucci, Fabrizio, and Lorenzo Peruzzi. 2014. 'Thymus Paronychioides (Lamiaceae), a Neglected Species from Sicily Belonging to Thymus Sect. Hypodromi'. *Folia Geobotanica* 49 (1): 83–106. https://doi.org/10.1007/s12224-013-9156-2.

Constantinidis, Theophanidis, Eleftheria-Perdiko Bareka, and Georgia Kamari. 2002. 'Karyotaxonomy of Greek Serpentine Angiosperms'. *Botanical Journal of the Linnean Society* 139 (1): 109–24. https://doi.org/10.1046/j.1095-8339.2002.00044.x.

Hanlidou, Effie, et Diamanto Lazari. 2013. « Essential oils of Thymus leucospermus Hartvig, a Greek endemic rich in phenolic monoterpenes ». *Natural Product Research* 27 (19): 1800-1803. https://doi.org/10.1080/14786419.2012.755681.

Hartvig, Per. 1987. 'A Taxonomical Revision ofThymus Sect.Teucrioides (Lamiaceae)'. *Plant Systematics and Evolution* 155 (1–4): 197–213. https://doi.org/10.1007/BF00936299.

Kakouri, Eleni, Dimitra Daferera, Anastasia Andriopoulou, Panayiotis Trigas, and Petros A. Tarantilis. 2024. 'Evaluation of the Essential Oil Composition of Five Thymus Species Native to Greece'. *Chemosensors* 12 (1): 1. https://doi.org/10.3390/chemosensors12010007.

Maloupa, E., K. Grigoriadou, K. Papanastassi, and N. Krigas. 2008. 'CONSERVATION, PROPAGATION, DEVELOPMENT AND UTILIZATION OF XEROPHYTIC SPECIES OF THE NATIVE GREEK FLORA TOWARDS COMMERCIAL FLORICULTURE'. *Acta Horticulturae*, no. 766 (March): 205–14. https://doi.org/10.17660/ActaHortic.2008.766.27.

Maloupa, Eleni, Nikos Krigas, Katerina Grigoriadou, Diamanto Lazari, and Georgios Tsoktouridis. 2008. *Conservation Strategies for Native Plant Species and Their Sustainable Exploitation: Case of the Balkan Botanic Garden of Kroussia, N. Greece*. 21.

Morales, Ramón. 1997. 'Synopsis of the Genus Thymus L. in the Mediterranean Area'. https://idus.us.es/xmlui/bitstream/handle/11441/63020/Synopsis%20Morales.pdf?sequence=1.

Negiz, M.G., and E.Ö. Aygül. 2020. 'Relationships between Alpha Plant Species Diversity and Environmental Variables in Dedegöl (YENİŞARBADEMLİ) Mountain Area.' *Applied Ecology and Environmental Research* 18 (1): 173–83. https://doi.org/10.15666/aeer/1801_173183.

Özocak, Murat, and Erkan Ünalan. 2020. 'Taxonomic Investigation of Medicinal and Aromatic Plants with Natural Growing Characteristics in Kastamonu (Hanönü) Region'. *Current Perspectives on Medicinal and Aromatic Plants (CUPMAP)*, ahead of print, November 19. https://doi.org/10.38093/cupmap.827402.

Pitarokili, D., E. Ioannou, O. Tzakou, C. Vagias, and V. Roussis. 2008. 'New Terpenoids from Thymus Teucrioides Subsp. Candilicus'. *Planta Medica* 74 (9): PB145. https://doi.org/10.1055/s-0028-1084490.

Pitarokili, Danae, Theophanis Constantinidis, Costas Saitanis, and Olga Tzakou. 2014. 'Volatile Compounds in *Thymus* Sect. *Teucrioides* (Lamiaceae): Intraspecific and Interspecific Diversity, Chemotaxonomic Significance and Exploitation Potential'. *Chemistry & Biodiversity* 11 (4): 593–618. https://doi.org/10.1002/cbdv.201300333.

Pitarokili, Danae, Antonios Michaelakis, George Koliopoulos, Athanassios Giatropoulos, and Olga Tzakou. 2011. 'Chemical Composition, Larvicidal Evaluation, and Adult Repellency of Endemic Greek Thymus Essential Oils against the Mosquito Vector of West Nile Virus'. *Parasitology Research* 109 (2): 425–30. https://doi.org/10.1007/s00436-011-2271-1.

Sarropoulou, Virginia, and Eleni Maloupa. 2019. 'Asexual Propagation of Four Medicinal Greek Endemic Plants of Lamiaceae Family With Conservation Priority From The Collection of The Balkan Botanic Garden of Kroussia, N. Greece'. *JOURNAL OF ADVANCES IN AGRICULTURE* 10 (January): 1611–22.

Siljak-Yakovlev, Sonja, Perla Farhat, Nicolas Valentin, Pepy Bareka, and Georgia Kamari. 2019. *New Estimates of Nuclear DNA Amount for 25 Taxa from Kefallinia Island*. 23.

Stahl-Biskup, Elisabeth, and Francisco Saez. 2003. *Thyme: The Genus Thymus*. CRC Press.

Tsiftsoglou, Olga S., Rafaela Stagiopoulou, Nikos Krigas, and Diamanto Lazari. 2023. 'Exploring the Ecological Preferences and Essential Oil Variability in Wild-Growing Populations of the Endangered Local Greek Endemic Thymus Holosericeus (Lamiaceae)'. *Plants* 12 (2): 2. https://doi.org/10.3390/plants12020348.

Tzakou, Olga, and Theophanis Constantinidis. 2005. 'Chemotaxonomic Significance of Volatile Compounds in Thymus Samius and Its Related Species Thymus Atticus and Thymus Parnassicus'. *Biochemical Systematics and Ecology* 33 (11): 1131–40. https://doi.org/10.1016/j.bse.2005.03.008.

Καδόγλου, Νικόλαος Π. 2015. *Μορφολογική, φαινολογική και χημειοτυπική διαφοροποίηση αυτοφυών πληθυσμών αρωματικών και φαρμακευτικών φυτών τύπου καρβακρόλης (Origanum spp., Thymbra spp. και Satureja spp.) προερχόμενων από την Ικαρία και την Κεφαλονιά.* September 18. http://dspace.aua.gr/xmlui/handle/10329/6038.

Σκροπολίθας, Ανδρέας. 2015. 'Σύγκριση Αρωματικών Φαρμακευτικών Φυτών Τύπου "Καρβακρόλης" Από Την Κεφαλονιά Και Την Ικαρία Ως Προς Τα Αναπαραγωγικά, Χημειοτυπικά Και Βιοδραστικά Τους Χαρακτηριστικά'. September 17. http://dspace.aua.gr/xmlui/bitstream/handle/10329/6036/Skropolithas_A.pdf?sequence=3.

Liliaceae

Fritillaria

Archibald, Jim, et Jenny Archibald. 1998. « The Need to Know - News from Jim and Jenny Archibald, Newsletter and seed list »,

septembre 1998. http://files.srgc.net/archibald/seedlists/JJA_seeds/JJA_seeds_1998.2_September.pdf.

Iliadou, Eleni, Ioannis Bazos, Konstantinos Kougioumoutzis, et al. 2020. 'Taxonomic and Phylogenetic Diversity Patterns in the Northern Sporades Islets Complex (West Aegean, Greece)'. *Plant Systematics and Evolution* 306 (2): 28. https://doi.org/10.1007/s00606-020-01660-0.

Johnston, J.Spencer, Alan E. Pepper, Anne E. Hall, Z. Jeffrey Chen, George Hodnett, Janice Drabek, Rebecca Lopez, et H. James Price. 2005. « Evolution of Genome Size in Brassicaceae ». *Annals of Botany* 95 (1): 229-35. https://doi.org/10.1093/aob/mci016.

Kamari, Georgia. 1984. 'Caryosystematic Studies on «Fritillaria» L. (Liliaceae) in Greece. 1'. *Webbia* 38 (1): 723–31. https://doi.org/10.1080/00837792.1984.10670343.

Kamari, Georgia. 1985. 'Fritillaria Sporadum (Liliaceae), a New Species from N Sporades (Greece)'. *Willdenowia* 14 (2): 331–33.

Kamari, Georgia. 1996. 'Fritillaria Species (Liliaceae) with Yellow or Yellowish-Green Flowers in Greece'. *Bocconea* 5: 221–38.

Kamari, Georgia, and Dimitrios Phitos. 2006. 'Karyosystematic Study of Fritillaria Messanensis s.l. (Liliaceae)'. *Willdenowia* 36 (1): 217–33. https://doi.org/10.3372/wi.36.36118.

Kamari, Georgia, Athanasios Zahos, and Ioanna Siagou. 2017. 'A New Yellow-Flowered Fritillaria Species (Liliaceae) from Mt. Tisseon, Continental Greece and Its Taxonomic Relationships'. *Phytotaxa* 328 (3): 227. https://doi.org/10.11646/phytotaxa.328.3.2.

Kokkoris, Y, and M Arianoutsou. 2004. 'Demographic Monitoring of Four Endemic Plant Taxa in the Fire Prone-Environments of Central Greece: Early Results'. Paper presented at 10th MEDECOS Conference, Rhodes, Greece. *Proceedings 10th MEDECOS Conference*, April 25. http://www.uaeco.edu.gr/files/PDF/P_MEDECOS/112.pdf.

Krigas, Nikos, Georgios Tsoktouridis, Ioannis Anestis, et al. 2021. 'Exploring the Potential of Neglected Local Endemic Plants of Three Mediterranean Regions in the Ornamental Sector: Value Chain Feasibility and Readiness Timescale for Their Sustainable Exploitation'. *Sustainability* 13 (5): 5. https://doi.org/10.3390/su13052539.

Krigas, Nikos, Viktoria Menteli, et Despoina Vokou. 2014. « The Electronic Trade in Greek Endemic Plants: Biodiversity, Commercial and Legal Aspects ». *Economic Botany* 68 (1): 85-95. https://doi.org/10.1007/s12231-014-9264-9.

Menteli, Viktoria, Nikos Krigas, Manolis Avramakis, Nicholas Turland, and Despoina Vokou. 2019. 'Endemic Plants of Crete in Electronic Trade and Wildlife Tourism: Current Patterns and Implications for Conservation'. *Journal of Biological Research-Thessaloniki* 26 (1): 10. https://doi.org/10.1186/s40709-019-0104-z.

Raab-Straube, Eckhard Von, and Thomas Raus. 2016. 'Euro Med-Checklist Notulae, 6'. *Willdenowia* 46 (3): 423–42. https://doi.org/10.3372/wi.46.46310.

Rix, Martyn. 2000. '397. Fritillaria Tuntasia: Liliaceae'. *Curtis's Botanical Magazine* 17 (3): 176–78.

Roguz, Katarzyna, Andrzej Bajguz, Agnieszka Gołębiewska, Magdalena Chmur, Laurence Hill, Paweł Kalinowski, Jürg Schönenberger, Małgorzata Stpiczyńska, et Marcin Zych. 2018. « Functional Diversity of Nectary Structure and Nectar Composition in the Genus Fritillaria (Liliaceae) ». *Frontiers in Plant Science* 0. https://doi.org/10.3389/fpls.2018.01246.

Samaropoulou, Sofia, Pepy Bareka, Dimitris L. Bouranis, and Georgia Kamari. 2019. 'Seed Morphology in the Genus Fritillaria (Liliaceae) from Greece and Its Taxonomic Significance'. *Phytotaxa* 416 (4): 223–37. https://doi.org/10.11646/phytotaxa.416.4.1.

Samaropoulou, S., P. Bareka, R. Artelari, et G. Kamari. 2013. « Karyological Studies on Some Endemic and Rare Species of Kefalonia, Ionian Islands, Greece ». *Flora Mediterranea* 23 (décembre). https://doi.org/10.7320/FlMedit23.215.

Samaropoulou, Sofia, Pepy Bareka, and Georgia Kamari. 2016. 'Karyomorphometric Analysis of Fritillaria Montana Group in Greece'. *Comparative Cytogenetics* 10 (4): 679–95. https://doi.org/10.3897/CompCytogen.v10i4.10156.

Samaropoulou, Sofia, Pepy Bareka, et Georgia Kamari. 2020. « Hybridization and karyotype variability of three endemic Fritillaria L. (Liliaceae) in Argolis Peninsula (Greece) ». *Plant Biosystems - An International Journal Dealing with all Aspects of Plant Biology* 154 (3): 348-60. https://doi.org/10.1080/11263504.2019.1612478.

Wilford, Richard. 2000. 'The Cultivation of Fritillaria Species at Kew'. *Curtis's Botanical Magazine* 17 (3): 149–54. https://doi.org/10.1111/1467-8748.00259.

Zaharof, Eugenia. 1986. 'Fritillaria Rixii (Liliaceae), a New Species from Euboea, Greece'. *Nordic Journal of Botany* 6 (6): 725–28. https://doi.org/10.1111/j.1756-1051.1986.tb00474.x.

Zaharof, Eugenia. 1987. « Fritillaria rhodocanakis subsp. argolica (Liliaceae), a New Subspecies from Peloponnese, Greece ». *Willdenowia* 16 (2): 343-48.

Zaharof, Eugenia. 1988. 'A Phenetic Study ofFritillaria (Liliaceae) in Greece'. *Plant Systematics and Evolution* 161 (1): 23–34. https://doi.org/10.1007/BF00936009.

Σαμαροπούλου, Σοφία Α. 2021. *Βιοσυστηματική μελέτη ειδών του γένους Fritillaria L. (LILIACEAE)*. May 11. http://dspace.aua.gr/xmlui/handle/10329/7335.

Gagea

Strid, Arne, and Kit Tan. 2017. 'Recent Progress In Plant Taxonomy And Floristic Studies In Greece'. *Botanica Serbica* 41 (2): 123–52. https://doi.org/10.5281/ZENODO.1026649.

Tulipa

Bareka, P., G. Kamari, N.J. Turland, and D. Phitos. 2015. 'Karyomorphological Study of Some Cretan Archeophytes'. *Flora Mediterranea* 25 (Special Issue). https://doi.org/10.7320/FlMedit25SI.127.

Basak, Nesibe, et Neriman Ozhatay. 1997. « Cytotaxonomic Notes on the Tulipa Species (Liliaceae) of European Turkey », 5.

Bergmeier, Erwin, and Arne Strid. 2014. 'Regional Diversity, Population Trends and Threat Assessment of the Weeds of Traditional Agriculture in Greece'. *Botanical Journal of the Linnean Society* 175 (4): 607–23. https://doi.org/10.1111/boj.12181.

Bustard, L. 1988. 'The Tulip Twins of Crete.' *Plantsman* 9 (3): 154–56.

Couzin, D. A., et D. P. Fox. 1973. « U-Type Exchange in Tulip Meiosis ». *Chromosoma* 41 (4): 421-36. https://doi.org/10.1007/BF00396500.

Cullen, C. J., et A. C. Fabergié. 1939. « The rate of temperature change within the plant ». *Annals of Botany* 3 (3): 759-60. https://doi.org/10.1093/oxfordjournals.aob.a085087.

Hançer, Çağla Kizilarslan, Ece Sevgi, Betül Büyükkiliç Altinbaşak, Ernaz Altundağ Çakir, et Muhammet Akkaya. 2020. « Traditional Knowledge of Wild Edible Plants of Biga (Çanakkale), Turkey ». *Acta Societatis Botanicorum Poloniae* 89 (1): 19.

Hatzilazarou, Stefanos, Elias Pipinis, Stefanos Kostas, et al. 2023. 'Influence of Temperature on Seed Germination of Five Wild-Growing Tulipa Species of Greece Associated with Their Ecological Profiles: Implications for Conservation and Cultivation'. *Plants* 12 (7): 7. https://doi.org/10.3390/plants12071574.

Kılıçaslan, Naşit, et Şirin Dönmez. 2016. « Göller bölgesinde doğal olarak yetişen soğanlı bitkilerin peyzaj mimarlığında kullanımı - Utilization of bulbous plants in landscape architecture growing in Lakes Region ». *Turkish Journal of Forestry | Türkiye Ormancılık Dergisi* 17 (1). https://doi.org/10.18182/tjf.36974.

Krigas, Nikos, Christos Lykas, Ioannis Ipsilantis, et al. 2021. 'Greek Tulips: Worldwide Electronic Trade over the Internet, Global Ex Situ Conservation and Current Sustainable Exploitation Challenges'. *Plants* 10 (3): 3. https://doi.org/10.3390/plants10030580.

Krigas, Nikos, Georgios Tsoktouridis, Ioannis Anestis, et al. 2021. 'Exploring the Potential of Neglected Local Endemic Plants of Three Mediterranean Regions in the Ornamental Sector: Value Chain Feasibility and Readiness Timescale for Their Sustainable Exploitation'. *Sustainability* 13 (5): 5. https://doi.org/10.3390/su13052539.

Lykas, Christos, Maria Zografou, Ioulietta Samartza, et al. 2023. 'Vase Life Evaluation of Three Greek Tulip Species Compared with a Commercial Cultivar'. *Horticulturae* 9 (8): 8. https://doi.org/10.3390/horticulturae9080928.

Menteli, Viktoria, Nikos Krigas, Manolis Avramakis, Nicholas Turland, and Despoina Vokou. 2019. 'Endemic Plants of Crete in Electronic Trade and Wildlife Tourism: Current Patterns and Implications for Conservation'. *Journal of Biological Research-Thessaloniki* 26 (1): 10. https://doi.org/10.1186/s40709-019-0104-z.

Meyer, Stefan, and Erwin Bergmeier. 2020. 'The Status of Arable Plant Habitats in Greece – The Cradle of Arable Farming in Europe'. In *The Changing Status of Arable Habitats in Europe: A Nature Conservation Review*, edited by Clive Hurford, Phil Wilson, and Jonathan Storkey. Springer International Publishing. https://doi.org/10.1007/978-3-030-59875-4_8.

Nagase, Ayako, et Nigel Dunnett. 2013. « Performance of Geophytes on Extensive Green Roofs in the United Kingdom ». *Urban Forestry & Urban Greening* 12 (4): 509-21. https://doi.org/10.1016/j.ufug.2013.06.005.

Oliveira, M. 1994. *Factors Affecting Seed Germination of Tulipa Cretica*. https://agris.fao.org/search/en/providers/122636/records/6471f649f762bd4124ec822f.

Sargin, Seyid Ahmet, Selami Selvi, et Ekrem Akçiçek. 2013. « Alaşehir (Manisa) ve Çevresinde Yetişen Bazı Geofitlerin Etnobotanik Açıdan İncelenmesi - Investigations of Ethnobotanical aspect of Some Geophytes Growing in Alaşehir (Manisa) and Surrounding Area ». *Erciyes University Journal of the Institute of Science and Technology* 29 (2): 9.

Satil, Fatih, Gülendam Tümen, Tuncay DiRmenci, Ali ÇeliK, Yılmaz Ari, et Hulusi Malyer. 2006. « Kazdağı Milli Parkı ve Çevresinde (Balıkesir) Etnobotanik Envanter Çalışması 2004-2006 ». *Türkiye Bilimler Akademisi Kültür Envanteri Dergisi*, nº 5 (juin): 171-203. https://doi.org/10.22520/tubaked.2006.0008.

Sik, Levent, Osman Erol, Kamuran Aktas, et Ceren Alparslan. 2015. « Manisa lalesi Tulipa orphanidea Boiss. ex Heldr. tür eylem plani - Tulipa orphanidea Boiss. ex Heldr. of Manisa action plan ». Manisa: Ministry of Forestry and Water Management. https://bolge4.tarimorman.gov.tr/Documents/Manisa%20lalesi%20t%C3%BCr%20koruma%20eylem%20plan%C4%B1.pdf.

Suzukawa, Keisuke, Takeshi Yamagami, Takayuki Ohnuma, et al. 2003. 'Mutational Analysis of Amino Acid Residues Involved in Catalytic Activity of a Family 18 Chitinase from Tulip Bulbs'. *Bioscience, Biotechnology, and Biochemistry* 67 (2): 341–46. https://doi.org/10.1271/bbb.67.341.

Yamagami, Takeshi, Toki Taira, Yoichi Aso, and Masatsune Ishiguro. 1998. 'Isolation and Characterization of Chitinase Isoforms from the Bulbs of Four Species of the Genus *Tulipa*'. *Bioscience, Biotechnology, and Biochemistry* 62 (3): 584–87. https://doi.org/10.1271/bbb.62.584.

Linaceae

Linum

Christodoulakis, D. 1995. 'A New Subspecies of Linum Gyaricum (Linaceae) from Greece'. *Nordic Journal of Botany* 15 (2): 145–47. https://doi.org/10.1111/j.1756-1051.1995.tb00131.x.

Christodoulakis, Dimitrios, et Gregory Iatrou. 1994. « Linum phitosianum spec. nova (Linaceae) aus Griechenlan ». *Phyton (Horn, Austria)* 33 (2): 289-94.

Diapoulis, Ch. 1959. 'Conservation Measures for the Plants of the Greek Flora.' http://documents.irevues.inist.fr/bitstream/handle/2042/59423/LATERREETLAVIE_1959_Sup_189.pdf?sequence=1.

Goulimis, C. 1958. *Report on Species of Plants Requiring Protection in Greece and Measures for Securing Their Protection*. Athens. http://documents.irevues.inist.fr/bitstream/handle/2042/59422/LATERREETLAVIE_1959_Sup_168.pdf?sequence=1.

Iatrou, Gregory A. 1989. « Linum hellenicum (Linaceae), a New Species from Peloponnesos, Greece ». *Willdenowia* 19 (1): 69-73.

Iliadou, Eleni, Ioannis Bazos, Konstantinos Kougioumoutzis, et al. 2020. 'Taxonomic and Phylogenetic Diversity Patterns in the Northern Sporades Islets Complex (West Aegean, Greece)'. *Plant Systematics and Evolution* 306 (2): 28. https://doi.org/10.1007/s00606-020-01660-0.

Kamari, Georgia, Dimitrios Phitos, Britt Snogerup, and Sven Snogerup. 1988. 'Flora and Vegetation of Yioura, N Sporades, Greece'. *Willdenowia* 17 (1/2): 59–85. JSTOR.

Spanou, Sofia, Eirini Aplada, Argyro Tiniakou, and Theodoros Georgiadis. 2010. *Contribution to the Study of the Flora of Attiki (Greece), New Records from the Flora of the Wider Athens International Airport Area*. 17.

Strange, Kit, and Martyn Rix. 2007. '574. Linum Doerfleri'. *Curtis's Botanical Magazine* 24 (1): 12–17. https://doi.org/10.1111/j.1467-8748.2007.00553.x.

Strid, Arne. 2015. 'Reliquiae Runemarkianae. Chromosome Numbers of Angiosperms from the Aegean Islands'. *Phytologia Balcanica* 21 (3): 245–93.

Strid, Arne. 2020. 'The Botanical Exploration of Greece'. *Plant Systematics and Evolution* 306 (2): 27. https://doi.org/10.1007/s00606-020-01637-z.

Trigas, Panayiotis, and Gregoris Iatrou. 2006. 'The Local Endemic Flora of Evvia (W Aegean, Greece)'. *Willdenowia* 36 (1): 257–70. https://doi.org/10.3372/wi.36.36121.

Onagraceae

Epilobium

Krajšek, Simona Strgulc, Marina Dermastia, and Nejc Jogan. 2006. 'Determination Key for Central European Epilobium Species Based on Trichome Morphology'. *Botanica Helvetica* 116 (2): 169–78. https://doi.org/10.1007/s00035-006-0770-y.

Snogerup, S. 1982. 'A New Species of Epilobium (Onagraceae) from Northern Greece'. *Willdenowia* 12 (2): 227–29.

Orchidaceae

Anacamptis

Cozzolino, Salvatore, Serena Aceto, Paolo Caputo, Alex Widmer, et Amots Dafni. 2001. « Speciation Processes in Eastern Mediterranean Orchis s.l. Species: Molecular Evidence and the Role of Pollination Biology ». *Israel Journal of Plant Sciences*, 13.

Delforge, Pierre. 2010. 'Un Nom Pour La Variété Égéenne de l'Orchis Papillon'. *Natural. Belges* 91 (Orchid. 23): 15–25.

Tsiftsis, Spyros, et Vladan Djordjević. 2018. « Habitat Effects and Differences in the Reproductive Success of Orchis Punctulata and Orchis Purpurea (Orchidaceae) ». *Turk J Bot*, juillet, 12.

Cephalanthera

IUCN. 2018. 'Cephalanthera Cucullata: Véla, E.: The IUCN Red List of Threatened Species 2018: E.T161912A123982623'. January 23. https://doi.org/10.2305/IUCN.UK.2018-1.RLTS.T161912A123982623.en.

Krigas, Nikos, Georgios Tsoktouridis, Ioannis Anestis, et al. 2021. 'Exploring the Potential of Neglected Local Endemic Plants of Three Mediterranean Regions in the Ornamental Sector: Value Chain Feasibility and Readiness Timescale for Their Sustainable Exploitation'. *Sustainability* 13 (5): 5. https://doi.org/10.3390/su13052539.

Thanos, Costas A., Christini Fournaraki, Kyriacos Georghiou, and Panayotis Dimopoulos. 2013. 'PMRs in Western Crete'. https://www.researchgate.net/profile/Costas_Thanos/publication/258119110_PMRs_in_Western_Crete/links/02e7e5270d8a300f04000000.pdf.

Dactylorhiza

Delforge, Pierre. 2008. 'Contribution à La Connaissance Des Orchidées de l'île de Samos (Egée Orientale, Grèce)'. *Natural. Belges* 89 (Orchid. 21): 71–251.

Dimitrov, D. 2023. 'Plant and Habitat Diversity of the Rilsko Korito Valley'. *Trakia Journal of Sciences* 21 (1): 1–11. https://doi.org/10.15547/tjs.2023.01.001.

Djordjević, Vladan, Svetlana Aćić, Eva Kabaš, Predrag Lazarević, Spyros Tsiftsis, and Dmitar Lakušić. 2023. 'The Orchids of Wetland Vegetation in the Central Balkans'. *Diversity* 15 (1): 1. https://doi.org/10.3390/d15010026.

Stefanaki, Anastasia, Aphrodite Kantsa, Thomas Tscheulin, Martha Charitonidou, and Theodora Petanidou. 2015. 'Lessons from Red Data Books: Plant Vulnerability Increases with Floral Complexity'. *PLOS ONE* 10 (9): e0138414. https://doi.org/10.1371/journal.pone.0138414.

Tsiftsis, Spyros, Vassiliki Karagiannakidou, Ioannis Tsiripidis, and I Tsiripidis. 2007. 'The Orchid Flora of East Macedonia (NE Greece)'. *J. Eur. Orch.* 39 (3–4): 489–526.

Tsiftsis, Spyros, and Pavel Kindlmann. 2023. 'Advances in Orchid Research in East Macedonia (NE Greece) and the Importance of Current Data in Furthering Our Understanding of the Orchids' Altitudinal Requirements'. *Journal for Nature Conservation* 72 (April): 126346. https://doi.org/10.1016/j.jnc.2023.126346.

Tsiftsis, Spyros, Ioannis Tsiripidis, Vassiliki Karagiannakidou, and Dimitrios Alifragis. 2008. 'Niche Analysis and Conservation of the Orchids of East Macedonia (NE Greece)'. *Acta Oecologica* 33 (1): 27–35. https://doi.org/10.1016/j.actao.2007.08.001.

Epipactis

Antonopoulos, Zissis, and Spyros Tsiftsis. 2012. 'Epipactis Purpurata SM. and Epipactis Leptochila (GODF.) GODF. Subsp. Neglecta KÜMPEL (Orchidaceae), Two New Epipactis Taxa for the Flora of Greece'. *Ber. Arbeitskrs. Heim. Orchid.* 29 (1): 81–99.

Hertel, Stefan, Spyros Tsiftsis, and Zissis Antonopoulos. 2014. 'Epipactis Pinovica (Orchidaceae), a New Orchid Species of Greece'. *J. Eur. Orch.* 46 (3–4): 701–14.

Krigas, Nikos, Georgios Tsoktouridis, Ioannis Anestis, et al. 2021. 'Exploring the Potential of Neglected Local Endemic Plants of Three

Mediterranean Regions in the Ornamental Sector: Value Chain Feasibility and Readiness Timescale for Their Sustainable Exploitation'. *Sustainability* 13 (5): 5. https://doi.org/10.3390/su13052539.

Szentpéteri, LJ, and F. Monus. 1999. 'Epipactis Degenii Szentpéteri & Mónus Spec. Nov., a New Epipactis Species from Greece'. *J. Eur. Orch* 31 (3): 644–51.

Tsiftsis, Spyros, Zuzana Štípková, and Pavel Kindlmann. 2019. 'Role of Way of Life, Latitude, Elevation and Climate on the Richness and Distribution of Orchid Species'. *Biodiversity and Conservation* 28 (1): 75–96. https://doi.org/10.1007/s10531-018-1637-4.

Tsiftsis, Spyros, Ioannis Tsiripidis, and Panayiotis Trigas. 2011. 'Identifying Important Areas for Orchid Conservation in Crete'. *European Journal of Environmental Sciences* 1 (2): 2. https://doi.org/10.14712/23361964.2015.44.

Himantoglossum

Pénzes-Kónya, Erika, Teresa Nowak, Anastasiia Holubenko, et al. 2015. Planta Europa Wild Orchid Conservation Workshop 2015. https://doi.org/10.13140/RG.2.1.1785.0961.

Χαριτωνίδου, Μ, Σ Τσιφτσής, Π Μαδέσης, and Ανδρέας Δρούζας. 2015. 'Ποικιλότητα και διαφοροποίηση των ειδών Himantoglossum jankae και H. samariense με το δείκτη ISSR UBC-811'. Paper presented at 14ο Πανελλήνιο Συνέδριο της Ελληνικής Βοτανικής Εταιρείας. Aristotle University of Thessaloniki Institutional Repository - IKEE, IKEECONFAN-2015-918. https://ikee.lib.auth.gr/record/275951.

Χαριτωνίδου, Μάρθα, Σπύρος Τσιφτσής, Ανδρέας Δρούζας, and Παναγιώτης Μαδέσης. 2016. 'Himantoglossum jankae και H. samariense (Orchidaceae): Τα φαινόμενα απατούν'; Paper presented at 8ο Πανελλήνιο Συνέδριο Οικολογίας. Aristotle University of Thessaloniki Institutional Repository - IKEE, IKEECONFAN-2020-022. https://ikee.lib.auth.gr/record/310714.

Ophrys

Delforge, Pierre. 2004. 'Un Ophrys Lacédémonien'. *Natural. Belges* 85 (Orchid. 17): 235–44.

Paulus, Hannes F. 2018. 'Ein neuer Fall von Hummelbestäubung auf der Ionischen Insel Kefalonia: Ophrys mavromata (Orchidaceae)'. *J. Eur. Orch.* 50 (2–4): 247–63.

Wood, Jeffrey. 1986. « Some European orchis in cultivation ». *The Kew Magazine* 3 (1): 14-38.

Orchis

Youssef, Sami, Ahmed Mahmood, Honar Mahdi, and Errol Vela. 2015. 'New Contribution on Orchids (Orchidaceae) of Duhok Province in Kurdistan Region (N-Iraq)'. *J. Eur. Orch.* 47 (2–4): 405–20.

Serapias

Krigas, Nikos, Georgios Tsoktouridis, Ioannis Anestis, et al. 2021. 'Exploring the Potential of Neglected Local Endemic Plants of Three Mediterranean Regions in the Ornamental Sector: Value Chain Feasibility and Readiness Timescale for Their Sustainable Exploitation'. Sustainability 13 (5): 5. https://doi.org/10.3390/su13052539.

Orobranchaceae

Melampyrum

Theodoropoulos, Konstantinos, Fotios Xystrakis, Eleni Eleftheriadou, and Dimitrios Samaras. 2011. 'Vegetation Zones and Habitat Types in the Area of Responsibility of the Management Agency of Olympus National Park'. Scientific Annals of the Faculty of Forestry and Natural Environment ME/2002/45 (January).

Odontites

Pinto-Carrasco, Daniel, Agnes Scheunert, Günther Heubl, Enrique Rico, et M. Montserrat Martínez-Ortegai. 2017. « Unravelling the Phylogeny of the Root-Hemiparasitic Genus Odontites (Tribe Rhinantheae, Orobanchaceae): Evidence for Five Main Lineages ». *TAXON* 66 (4): 886-908. https://doi.org/10.12705/664.6.

Paeoniaceae

Paeonia

Dimitropoulou, E., A. Cheilari, P. Magiatis, K. Graikou, and I. Chinou. 2022. 'Paeonia Clusii Subsp. Clusii Seeds: Phytochemical Profile (LC-MS), q-NMR Determination of Paeoniflorin and Biological Activities'. *Planta Medica* 88 (15): P-193. https://doi.org/10.1055/s-0042-1759169.

Dimitropoulou, Eleni, Konstantia Graikou, Vithleem Klontza, and Ioanna Chinou. 2023. 'Chemical Profiling on Bioactive Stilbenoids in the Seeds of Paeonia Species Growing Wild in Greece'. *Separations* 10 (10): 10. https://doi.org/10.3390/separations10100540.

Klontza, V., E. Dimitropoulou, P. Magiatis, et al. 2021. 'Phytochemical Study-Biological Activities and Application of q 1H-NMR Method to Determination of Paeoniflorin/Gnetin in Three Paeonia Species, Endemic in Greece'. *Planta Medica* 87 (15): SL19. https://doi.org/10.1055/s-0041-1736767.

Klontza, Vithleem, Konstantia Graikou, Antigoni Cheilari, et al. 2023. 'Phytochemical Study on Seeds of Paeonia Clusii Subsp. Rhodia—Antioxidant and Anti-Tyrosinase Properties'. *International Journal of Molecular Sciences* 24 (5): 5. https://doi.org/10.3390/ijms24054935.

Krigas, Nikos, Viktoria Menteli, and Despoina Vokou. 2014. 'The Electronic Trade in Greek Endemic Plants: Biodiversity, Commercial and Legal Aspects'. *Economic Botany* 68 (1): 85–95. https://doi.org/10.1007/s12231-014-9264-9.

Kritsanida, Marina, Prokopios Magiatis, Alexios-Leandros Skaltsounis, and James P. Stables. 2007. 'Phytochemical Investigation and Anticonvulsant Activity of Paeonia Parnassica Radix'. *Natural Product Communications* 2 (4): 1934578X0700200401. https://doi.org/10.1177/1934578X0700200401.

Letsiou, Sophia, Artemis Bakea, Anna Holefors, et Jadwiga Rembiesa. 2020. « In Vitro Protective Effects of Paeonia Mascula Subsp. Hellenica Callus Extract on Human Keratinocytes ». *Scientific Reports* 10 (1): 19213. https://doi.org/10.1038/s41598-020-76169-0.

Michalea, R., V.-I. Boka, E. Dina, N. Aligiannis, and A. L. Skaltsounis. 2016. 'Greek Flora as a Source of New Anti-Oxidant, Anti-Elastase, Anti-Collagenase and Anti-Hyaluronidase Natural Agents'. *Planta Medica* 82 (S 1): P435. https://doi.org/10.1055/s-0036-1596533.

Michalea, Rozalia, Konstantina Stathopoulou, Panagiotis Polychronopoulos, Dimitra Benaki, Emmanuel Mikros, and Nektarios Aligiannis. 2020. 'Efficient Identification of Acetylcholinesterase and Hyaluronidase Inhibitors from *Paeonia Parnassica* Extracts through a HeteroCovariance Approach'. *Journal of Ethnopharmacology* 257 (July): 111547. https://doi.org/10.1016/j.jep.2018.10.008.

Papandreou, Vasiliki, Prokopios Magiatis, Ioanna Chinou, Eleftherios Kalpoutzakis, Alexios-Leandros Skaltsounis, and Anthony Tsarbopoulos. 2002. 'Volatiles with Antimicrobial Activity from the Roots of Greek Paeonia Taxa'. *Journal of Ethnopharmacology* 81 (1): 101–4. https://doi.org/10.1016/S0378-8741(02)00056-9.

Papandreou, Vasiliki, Prokopios Magiatis, Eleftherios Kalpoutzakis, Alexios-Leandros Skaltsounis, and Catherine Harvala. 2002. 'Paeonicluside, A New Salicylic Glycoside From The Greek Endemic Species Paeonia Clusii'. *Zeitschrift Für Naturforschung C* 57 (3–4): 235–38. https://doi.org/10.1515/znc-2002-3-406.

Tzanoudakis, Dimitris. 1983. 'Karyotypes of Four Wild Paeonia Species from Greece'. *Nordic Journal of Botany* 3 (3): 307–18. https://doi.org/10.1111/j.1756-1051.1983.tb01943.x.

Papaveraceae

Hypecoum

Dahl, Å. E., A. -b. Wassgren, and G. Bergström. 1990. 'Floral Scents in *Hypecoum* Sect. *Hypecoum* (Papaveraceae): Chemical Composition and Relevance to Taxonomy and Mating System'. *Biochemical Systematics and Ecology* 18 (2): 157–68. https://doi.org/10.1016/0305-1978(90)90053-I.

Dahl, Aslög. 1989. 'Taxonomic and Morphological Studies inHypecoum Sect.Hypecoum (Papaveraceae)'. *Plant Systematics and Evolution* 163 (3–4): 227–80. https://doi.org/10.1007/BF00936517.

Kaynar, E., A. Emir, B. Bozkurt, G. I. Kaya, M. A. Onur, and N. U. Somer. 2015. 'Quantitative Determination of Protopine in Hypecoum Procumbens Subsp. Atropunctatum'. *Planta Medica* 81 (16): PW_237. https://doi.org/10.1055/s-0035-1565861.

Plumbaginaceae

Acantholimon

Grigoriadou, Katerina, Nikos Krigas, Virginia Sarropoulou, Eleni Maloupa, and Georgios Tsoktouridis. 2021. 'Vegetative Propagation and Ex-Situ Conservation of Acantholimon Androsaceum and Limonium Chersonesum, Two Promising Local Endemics of Crete (Greece) Available for Floricultural and Pharmaceutical Sustainable Exploitation'. *Notulae Botanicae Horti Agrobotanici Cluj-Napoca* 49 (1): 12261–12261.

Krigas, Nikos, Georgios Tsoktouridis, Ioannis Anestis, et al. 2021. 'Exploring the Potential of Neglected Local Endemic Plants of Three Mediterranean Regions in the Ornamental Sector: Value Chain Feasibility and Readiness Timescale for Their Sustainable Exploitation'. *Sustainability* 13 (5): 5. https://doi.org/10.3390/su13052539.

Menteli, Viktoria, Nikos Krigas, Manolis Avramakis, Nicholas Turland, and Despoina Vokou. 2019. 'Endemic Plants of Crete in Electronic Trade and Wildlife Tourism: Current Patterns and Implications for Conservation'. *Journal of Biological Research-Thessaloniki* 26 (1): 10. https://doi.org/10.1186/s40709-019-0104-z.

Armeria

Christodoulakis, Dimitrios. 1996. 'The Phytogeographical Distribution Patterns of the Flora of Ikaria (E Aegean, Greece) within the E Mediterranean'. *Flora* 191 (4): 393–99. https://doi.org/10.1016/S0367-2530(17)30748-X.

Goulimis, C. 1958. *Report on Species of Plants Requiring Protection in Greece and Measures for Securing Their Protection*. Athens. http://documents.irevues.inist.fr/bitstream/handle/2042/59422/LATERREETLAVIE_1959_Sup_168.pdf?sequence=1.

Papanicolaou, K., and S. Kokkini. 1982. 'A New Species of Armeria (Plumbaginaceae) from Euboea, Greece'. *Willdenowia* 12 (2): 221–25.

Goniolimon

Buzurović, Uroš, Gordana Tomović, Marjan Niketić, Sandro Bogdanović, and Jelena M. Aleksić. 2020. 'Phylogeographic and Taxonomic Considerations on Goniolimon Tataricum (Plumbaginaceae) and Its Relatives from South-Eastern Europe and the Apennine Peninsula'. *Plant Systematics and Evolution* 306 (2): 29. https://doi.org/10.1007/s00606-020-01636-0.

Kalogeropoulos, Erotokritos, and Pinelopi Delipetrou. 2015. 'Aegean Floristic Elements in Attiki: New Records for Silene Holzmannii Hekdr. Ex Boiss. and Ophrys Basilissa C. Alibertis, A. Alibertis & H.R. Reinhard'. *Parnassiana Archives* 3: 11–17.

Limonium

Agricultural University of Athens. 2019. Life Andros Park, 'Conservation of Priority Species and Habitats of Andros Island Protected Area Integrating Socioeconomic Considerations'. Action A.1 - Final Report on the Plant Communities and Their Seasonal and Spatial Variation of the Target Habitat Including the Results of the Base Study. Agricultural University of Athens. http://www.life-androspark.gr/en/wp-content/uploads/2023/01/A1_AUA-Final-Report-on-Vegetation.pdf.

Apostolopoulos, Efstathios, and Theophanis Constantinidis. 2024. 'Limonium Ophioides and L. Nichoriense (Plumbaginaceae), Two New Diploid Species from Peloponnisos, Greece'. Phytotaxa 655 (2): 2. https://doi.org/10.11646/phytotaxa.655.2.4.

Artelari, Rea. 1989. 'Biosystematic Study of the Genus Limonium (Plumbaginaceae) in the Aegean Area (Greece). II. Limonium Hierapetrae Rech. Fil. from Kriti Island'. Webbia 43 (1): 33–40. https://doi.org/10.1080/00837792.1989.10670447.

Artelari, Rea, et M Erben. 1986. « Limonium brevipetiolatum - eine neue Hexaploide suppe aus Süd-Griechenland ». *Mitt. Bot. München* 22 (décembre): 507-11.

Artelari, R., and G. Kamari. 1986. 'A Karyological Study of Ten Limonium Species (Plumbaginaceae) Endemic in the Ionian Area, Greece'. Willdenowia 15: 497–513.

Artelari, Rea, et Georgia Kamari. 1995. « Limonium Kardamylii (Plumbaginaceae), a New Species from S Peloponnisos (Greece) ». *Phyton (Horn, Austria)* 35 (1): 131-37.

Artelari, Rea, et Ourania Georgiou. 1999. « Two New Species of Limonium (Plumbaginaceae) from the Island of Kithira (Greece) ». *Botanical Journal of the Linnean Society* 131 (4): 399-415. https://doi.org/10.1111/j.1095-8339.1999.tb01522.x.

Artelari, Rea, et Ourania Georgiou. 2003. « Biosystematic Study of the Genus Limonium (Plumbaginaceae) in the Aegean Area, Greece. EO. Limonium on the Islands Kithira and Antikithira and the Surrounding Islets ». *Nordic Journal of Botany* 22 (4): 483-502. https://doi.org/10.1111/j.1756-1051.2002.tb01402.x.

Bazos, Ioannis, Ioannis P. Kokkoris, et Panayotis Dimopoulos. 2021. « Diversity of Halophytes and Salt Tolerant Plants at the Species-, Habitats- and High-Rank Syntaxa Level in Greece ». In *Handbook of Halophytes*, édité par Marius-Nicusor Grigore, 787-820. Cham: Springer International Publishing. https://doi.org/10.1007/978-3-030-57635-6_26.

Brofas, George, George Karetsos, Maria Panitsa, and Michalis Theocharopoulos. 2001. 'The Flora and Vegetation of Gyali Island, SE Aegean, Greece'. Willdenowia 31 (1): 51–70.

Brullo, S., C. Brullo, S. Cambria, G. Giusso del Galdo, and P. Minissale. 2017. 'Phytosociological Investigation on the Class Crithmo Maritimi-Limonietea in Greece'. Plant Sociology, no. 54(1) (June): 3–57. https://doi.org/10.7338/pls2017541/01.

Brullo, Salvatore, and Matthias Erben. 2016. 'The Genus Limonium (Plumbaginaceae) in Greece'. Phytotaxa 240 (1): 1. https://doi.org/10.11646/phytotaxa.240.1.1.

Cattaneo, Cristina, and Mauro Grano. 2016. Contribution to the Knowledge of Vascular Flora on Astypalea Island (Dodecanese, Greece).

Cattaneo, Cristina, and Mauro Grano. 2019. 'Checklist Updating and Analysis of the Flora of Symi Island and of the Nearby Island of Seskli (Dodecanese, Greece)'. Bocconea 28: 425–63.

Crespo, Manuel B., et Carolina Pena-Martín. 2013. « Two New Species of Limonium (Plumbaginaceae) from Rhodes Island (Eastern Aegean Area, Greece) ». *Phytotaxa* 94 (2): 30. https://doi.org/10.11646/phytotaxa.94.2.1.

Delitheos, A., E. Tiligada, A. Yannitsaros, and I. Bazos. 1997. 'Antiphage Activity in Extracts of Plants Growing in Greece'. Phytomedicine 4 (2): 117–24. https://doi.org/10.1016/S0944-7113(97)80055-4.

Dogan, Yunus, Anely M. Nedelcheva, and Celal Yarci. 2008. 'Plant Taxa Used as Brooms in Several Southeast European and West Asian Countries'. Natura Croatica : Periodicum Musei Historiae Naturalis Croatici 17 (3): 193–206.

Fenu, Giuseppe, Gianluigi Bacchetta, S. Christodoulou Charalambos, et al. 2019. 'An Early Evaluation of Translocation Actions for Endangered Plant Species on Mediterranean Islands'. Plant Diversity, Restoration of threatened plant species and their habitats, vol. 41 (2): 94–104. https://doi.org/10.1016/j.pld.2019.03.001.

Georgakopoulou, Argyro, Sofia Manousou, Rea Artelari, et Ourania Georgiou. 2006. « Breeding Systems and Cytology in Greek Populations of Five Limonium Species (Plumbaginaceae) ». *Willdenowia* 36 (2): 741-50.

Goulimis, C. 1958. Report on Species of Plants Requiring Protection in Greece and Measures for Securing Their Protection. Athens. http://documents.irevues.inist.fr/bitstream/handle/2042/59422/LATERREETLAVIE_1959_Sup_168.pdf?sequence=1.

Grigoriadou, Katerina, Nikos Krigas, Virginia Sarropoulou, Eleni Maloupa, and Georgios Tsoktouridis. 2021. 'Vegetative Propagation and Ex-Situ Conservation of Acantholimon Androsaceum and Limonium Chersonesum, Two Promising Local Endemics of Crete (Greece) Available for Floricultural and Pharmaceutical Sustainable Exploitation'. Notulae Botanicae Horti Agrobotanici Cluj-Napoca 49 (1): 12261–12261.

Koutroumpa, Konstantina. 2024. 'Limonium Artelariae (Plumbaginaceae), a New Endemic Species and Further Taxonomic and Floristic Notes on the Genus in the Island of Crete'. Willdenowia 54 (1): 65–79. https://doi.org/10.3372/wi.54.54103.

Krigas, Nikolaos, Georgios Mouflis, Katerina Grigoriadou, and Eleni Maloupa. 2010. 'Conservation of Important Plants from the Ionian Islands at the Balkan Botanic Garden of Kroussia, N Greece: Using GIS to Link the in Situ Collection Data with Plant Propagation and Ex Situ Cultivation'. Biodiversity and Conservation 19 (12): 3583–603. https://doi.org/10.1007/s10531-010-9917-7.

Krigas, Nikos, Marina Panagiotidou, and Eleni Maloupa. 2017. 'Incorporating Biogeographical Principles in Horticulture: Design and Creation of the Ionian Islands Unique Rock Garden in Thessaloniki, Greece'. Sibbaldia: The Journal of Botanic Garden Horticulture 0 (15): 129–46.

Liolios, Vasilis, Chrysoula Tananaki, Maria Dimou, et al. 2015. 'Ranking Pollen from Bee Plants According to Their Protein Contribution to Honey Bees'. Journal of Apicultural Research 54 (5): 582–92. https://doi.org/10.1080/00218839.2016.1173353.

Maloupa, Eleni, Nikos Krigas, Katerina Grigoriadou, Diamanto Lazari, and Georgios Tsoktouridis. 2008. Conservation Strategies for Native Plant Species and Their Sustainable Exploitation: Case of the Balkan Botanic Garden of Kroussia, N. Greece. 21.

Öztürk, Münir, Volkan Altay, Salih Gucel, et Aykut Guvensen. 2014. « Halophytes in the East Mediterranean – Their Medicinal and Other Economical Values ». In *Sabkha Ecosystems: Volume IV: Cash Crop Halophyte and Biodiversity Conservation*, édité par M. Ajmal Khan, Benno Böer, Münir Öztürk, Thabit Zahran Al Abdessalaam, Miguel Clüsener-Godt, et Bilquees Gul, 47:247-72. Tasks for Vegetation Science. Dordrecht: Springer Netherlands. https://doi.org/10.1007/978-94-007-7411-7_18.

Strid, Arne. 2020. 'The Botanical Exploration of Greece'. Plant Systematics and Evolution 306 (2): 27. https://doi.org/10.1007/s00606-020-01637-z.

Trigas, Panayiotis, Eleftherios Kalpoutzakis, Epaminondas Kalogiannis, et al. 2021. 'Noteworthy New Floristic Records from Greece'. Botanica Serbica 45 (2): 321–31. https://doi.org/10.2298/BOTSERB2102321T.

Tsakiri, Maria, Konstantinos Kougioumoutzis, and Gregoris Iatrou. 2016. 'Contribution to the Vascular Flora of Chalki Island (East Aegean, Greece) and Biomonitoring of a Local Endemic Taxon'. Willdenowia 46 (1): 175–90. https://doi.org/10.3372/wi.46.46114.

Valli, Anna-Thalassini, and Rea Artelari. 2015. 'Limonium Korakonisicum (Plumbaginaceae), a New Species from Zakynthos Island (Ionian Islands, Greece)'. Phytotaxa 217 (1): 1. https://doi.org/10.11646/phytotaxa.217.1.5.

Τσιριπίδης, I. 2018. « Επεξεργασία με κατάλληλες στατιστικές μεθόδους δεδομένων βλάστησης με σκοπό την κατάρτιση καταλόγου υγροτοπικών φυτικών ειδών για την ελληνική επικράτεια - Processing with appropriate statistical methods of vegetation data in order to compile a list of wetland plant species for the Greek territory », décembre. http://repository.biodiversity-info.gr/handle/11340/1969.

Poaceae

Achnatherum

Scholz, Hildemar, et Thomas Raus. 2006. « Contribution to the Flora of Greece: A New Species of Achnatherum (Poaceae) ». *Willdenowia* 36 (1): 373-78.

Aegilops

Cattaneo, Cristina, and Mauro Grano. 2018. 'Contribution to the Flora of Tilos Island (Dodecanese Islands, Greece)'. Parnassiana Archives 6: 41–53.

Cattaneo, Cristina, and Mauro Grano. 2021. Kasos: An Unexpected Island. Floristic and Ecological Analysis of Kasos Island (SE Aegean, Dodecanese, Greece), with Noteworthy Floristic Additions. 28.

Furuta, Y. 1973. 'Origin and Differentiation of Tetraploid Species in the Section Polyeides of the Genus Aegilops. 1. Cytological Variation in Ae. Biuncialis Vis.' Japanese Journal of Genetics (Idengaku Zasshi) 48 (6): 411.

Furuta, Y. 1979. 'Origin and Differentiation of Tetraploid Species in the Section Polyeides of the Genus Aegilops. VIII. Cytogenetical Analysis of the F1 of a Diallel Cross between Six Strains of Ae. Biuncialis.' Japanese Journal of Genetics (Idengaku Zasshi) 54 (6): 431.

Brachypodium

Greuter, Werner, Ursula Matthäs, and Horst Risse. 1985. 'Additions to the Flora of Crete, 1973-1983 (1984) - III'. *Willdenowia* 15 (1): 23–60.

Bromus

Greuter, Werner, and Thomas Raus. 2010. 'Med-Checklist Notulae, 29'. *Willdenowia* 40 (2): 189–204. https://doi.org/10.3372/wi.40.40205.

Dactylis

Rubio Teso, Maria Luisa, Clara Álvarez Muñiz, Hannes Gaisberger, et al. 2020. *Crop Wild Relatives in Natura 2000 Network*. Report. https://cgspace.cgiar.org/handle/10568/110717.

Elytrigia – Relatives

Rubio Teso, Maria Luisa, Clara Álvarez Muñiz, Hannes Gaisberger, et al. 2020. *Crop Wild Relatives in Natura 2000 Network*. Report. https://cgspace.cgiar.org/handle/10568/110717.

Eragrostis

Greuter, Werner, and Thomas Raus. 2011. 'Med-Checklist Notulae, 30'. *Willdenowia* 41 (2): 311–28.

Festuca

Ardenghi, Nicola M.G., Graziana Fiorini, Graziano Rossi, et Bruno Foggi. 2016. « Chromosome Numbers and Karyomorphology of Festuca Circummediterranea Patzke Group (Poaceae, Loliinae) ». *Phytotaxa* 263 (3): 255. https://doi.org/10.11646/phytotaxa.263.3.6.

Löve, Áskell. 1981. « Chromosome Number Reports LXXIII ». *Taxon* 30 (4): 829-61.

Rechinger, K. H. 1936. 'New plants from the Aegean region.' *Osterreichische Botanische Zeiitschrift* 85: 56–64.

Rubio Teso, Maria Luisa, Clara Álvarez Muñiz, Hannes Gaisberger, et al. 2020. *Crop Wild Relatives in Natura 2000 Network*. Report. https://cgspace.cgiar.org/handle/10568/110717.

Scholz, Hildemar, et Arne Strid. 1992. « Festuca stygia (Poaceae), a New Species from Peloponnisos, Greece ». *Willdenowia* 22 (1/2): 85-88.

Theodoropoulos, Konstantinos, Fotios Xystrakis, Eleni Eleftheriadou, and Dimitrios Samaras. 2011. 'Vegetation Zones and Habitat Types in the Area of Responsibility of the Management Agency of Olympus National Park'. *Scientific Annals of the Faculty of Forestry and Natural Environment* ME/2002/45 (January).

Hordeum

Scholz, H., and T. Raus. 1997. 'Two New Subspecies of Hordeum Murinum (Gramineae) from Greece and Spain.' *Feddes Repertorium* 108 (7/8): 527–31.

Melica

Hempel, W. 1971. « Die systematische Stellung von Melica uniflora Retz. und Melica rectiflora Boiss. er Heldr. (Melica L. Subgen. Bulbimelica, subgen. nov.) (Vorarbeiten zu einer Revision der Gattung Melica L.–I) ». *Feddes Repertorium* 81 (10): 657-86. https://doi.org/10.1002/fedr.19710811002.

Phleum

Scholz, Hildemar. 1990. « Neue und wenig bekannte mediterrane Gramineen-Taxa ». *Willdenowia* 19 (2): 405-12.

Poa

Frajman, Bozo, Clemens Pachschwöll, and Peter Schönswetter. 2014. 'Contributions to the Knowledge of the Flora of the Dinarides (Balkan Peninsula)'. *Phyton (Horn, Austria)* 54 (1): 27–46.

Krigas, Nikolaos, Georgios Mouflis, Katerina Grigoriadou, and Eleni Maloupa. 2010. 'Conservation of Important Plants from the Ionian Islands at the Balkan Botanic Garden of Kroussia, N Greece: Using GIS to Link the in Situ Collection Data with Plant Propagation and Ex Situ Cultivation'. *Biodiversity and Conservation* 19 (12): 3583–603. https://doi.org/10.1007/s10531-010-9917-7.

Panitsa, Maria, and Eleni Iliadou. 2011. 'Flora and Phytogeography of the Ionian Islands (Greece)'. *Illes i Plantes: Conservacio i Coneixement de La Flora a Les Illes de La Mediterrania*, April, 21.

Seslaria

Krigas, Nikos, Georgios Tsoktouridis, Ioannis Anestis, et al. 2021. 'Exploring the Potential of Neglected Local Endemic Plants of Three Mediterranean Regions in the Ornamental Sector: Value Chain Feasibility and Readiness Timescale for Their Sustainable Exploitation'. *Sustainability* 13 (5): 5. https://doi.org/10.3390/su13052539.

Kuzmanović, Nevena, Dmitar Lakušić, Božo Frajman, Antun Alegro, et Peter Schönswetter. 2017. « Phylogenetic Relationships in Seslerieae (Poaceae) Including Resurrection of Psilathera and Sesleriella, Two Monotypic Genera Endemic to the Alps ». *TAXON* 66 (6): 1349-70. https://doi.org/10.12705/666.5.

Lazarević, Maja, Nevena Kuzmanović, Dmitar Lakušić, Antun Alegro, Peter Schönswetter, et Božo Frajman. 2015. « Patterns of Cytotype Distribution and Genome Size Variation in the Genus Sesleria Scop. (Poaceae): Genome Size in Sesleria ». *Botanical Journal of the Linnean Society* 179 (1): 126-43. https://doi.org/10.1111/boj.12306.

Stipa

Scholz, H. 1989. 'New Taxa of the Genus Stipa Sect. Stipa (Gramineae) from the Mediterranean Area.' *Willdenowia* 19 (1): 127–32.

Scholz, Hildemar. 1993. 'Festuca Rivularis, Poa Maroccana Und Stipa Monticola (Gramineae) in Griechenland'. *Willdenowia* 23 (1/2): 113–19.

Strid, Arne. 1981. 'New Species of Cephalaria (Dipsacaceae) and Stipa (Gramineae) from the Greek Mountains'. *Willdenowia* 11 (2): 301–5.

Polygalaceae

Polygala

De Montmollin, Bertrand, et Wendy Strahm. 2005. *The Top 50 Mediterranean Island Plants: Wild Plants at the Brink of Extinction, and What Is Needed to Save Them*. IUCN.

Greuter, Werner. 1994. « Extinctions in Mediterranean Areas ». *Philosophical Transactions: Biological Sciences* 344 (1307): 41-46.

Raabe, Uwe, Kit Tan, Gregoris Iatroú, Gert Vold, et Gerald Parolly. 2009. « Polygala rausiana (Polygalaceae), a new species from the northern Peloponnese, Greece ». *Willdenowia* 39 (1): 69-75. https://doi.org/10.3372/wi.39.39107.

Κολοκυθάς, Ευάγγελος. 2016. 'Ανάπλαση στην περιοχή της Πάρνηθας μετά την πυρκαγιά'. Bachelor Thesis, Ανώτατο Εκπαιδευτικό Ίδρυμα Πειραια. http://okeanis.lib2.uniwa.gr/xmlui/handle/123456789/3476.

Polygonaceae

Polygonum

Baliousis, E. 2014. 'Recent Data from the Flora of the Island of Limnos (NE Aegean, Greece): New Alien Invasive Species Affecting the Agricultural Economy of the Island'. *Edinburgh Journal of Botany* 71 (2): 275–85. https://doi.org/10.1017/S0960428614000110.

Gemici, Yusuf, and Kit Tan. 2014. 'Polygonum Melihae Sp. Nov. (Polygonaceae) from Inner West Anatolia, Turkey'. *Nordic Journal of Botany* 32 (5): 540–42. https://doi.org/10.1111/j.1756-1051.2013.00296.x.

Hartvig, Per. 1989. 'Polygonum Papillosum (Polygonaceae), a New Species from Central Greece'. *Willdenowia* 19 (1): 75–78.

Strid, Arne, and Kit Tan. 2009. 'A New Species of Centaurea (Asteraceae) from the Island of Samothraki (NE Greece)'. *Phytologia Balcanica* 15 (2): 185–89.

Primulaceae

Cyclamen

Affre, Laurence, and John D. Thompson. 1997. 'Population Genetic Structure and Levels of Inbreeding Depression in the Mediterranean Island Endemic Cyclamen Creticum (Primulaceae)'. *Biological Journal of the Linnean Society* 60 (4): 527–49. https://doi.org/10.1111/j.1095-8312.1997.tb01511.x.

Altay, Hamit, et Nuray Mucella Muftuoglu. 2004. « The Effects of Varying Applications of Nitrogen, Phosphorus and Potassium on the Size of Cyclamen Hederifolium Corms Grown in Peat Medium ». In . Erzurum, Turkey.

Dusen, Serdar, Cigdem Aydin, Hesna Yaka Gul, Cennet Ozay, Olcay Dusen, and Ramazan Mammadov. 2016. 'In Vitro Cytotoxic Activities of Cyclamen L. (Primulaceae) Ethanol Extracts from Turkey'. *Fresenius Environmental Bulletin* 25 (12a): 6224–28.

Greilhuber, Johann. 1989. 'Karyotype Structure and Evolution in *Cyclamen* L. Subgen. *Psilanthum* Schwz.*(Primulaceae)*1)'. *Flora* 183 (1): 103–13. https://doi.org/10.1016/S0367-2530(17)31545-1.

Krigas, Nikos, Viktoria Menteli, and Despoina Vokou. 2014. 'The Electronic Trade in Greek Endemic Plants: Biodiversity, Commercial and Legal Aspects'. *Economic Botany* 68 (1): 85–95. https://doi.org/10.1007/s12231-014-9264-9.

Krigas, Nikos, Georgios Tsoktouridis, Ioannis Anestis, et al. 2021. 'Exploring the Potential of Neglected Local Endemic Plants of Three Mediterranean Regions in the Ornamental Sector: Value Chain Feasibility and Readiness Timescale for Their Sustainable

Exploitation'. *Sustainability* 13 (5): 5. https://doi.org/10.3390/su13052539.

Menteli, Viktoria, Nikos Krigas, Manolis Avramakis, Nicholas Turland, and Despoina Vokou. 2019. 'Endemic Plants of Crete in Electronic Trade and Wildlife Tourism: Current Patterns and Implications for Conservation'. *Journal of Biological Research-Thessaloniki* 26 (1): 10. https://doi.org/10.1186/s40709-019-0104-z.

Samaropoulou, Sofia, Eleni Liveri, et Pepy Bareka. 2020. « Karyological Analysis of Cyclamen Hederifolium s.l. in Greece ». *CYTOLOGIA* 85 (3): 219-22. https://doi.org/10.1508/cytologia.85.219.

Schwarz, Otto, et Lothab Lepper. 1975. « Zwei neue Cyclamen aus dem östlichen Mittelmeergebiet ». *Feddes Repertorium* 86 (9-10): 491-97. https://doi.org/10.1002/fedr.19750860902.

Seyring, Martina, Aloma Ewald, Anke Mueller, and Klaus-Thomas Haensch. 2009. 'Screening for Propagation Suitability in Vitro of Different Cyclamen Species'. *Electronic Journal of Biotechnology* 12 (4): 10–11. https://doi.org/10.4067/S0717-34582009000400010.

Tan, Kit, and Konstantinos Giannopoulos. 2022. 'Contributions to the Bulb Flora of Ilias (NW Peloponnese, Greece): Oxalidaceae, Primulaceae and Ranunculaceae (Dicotyledons)'. *Phytologia Balcanica* 28 (2). https://doi.org/10.7546/PhB.28.2.2022.5.

Zare, Golshan, Emre Çilden, and Ömür Gençay Çelemli. 2023. 'Comparative Anatomical Survey of Cyclamen L. (Primulaceae) Taxa and Their Traditional Medicinal Uses in Turkey'. *Microscopy Research and Technique* 86 (4): 414–30. https://doi.org/10.1002/jemt.24282.

Lysimachia

Anderberg, Arne A., Ulrika Manns, et Mari Källersjö. 2007. « Phylogeny and floral evolution of the Lysimachieae (Ericales, Myrsinaceae): evidence from ndhF sequence data ». *Willdenowia* 37 (2): 407-21. https://doi.org/10.3372/wi.37.37202.

Brofas, G., Panayiotis Trigas, G. Mantakas, G. Karetsos, Costas A. Thanos, Kyriacos Georghiou, et Chr. Mermiris. 2007. « Rehabilitation of disturbed areas by mining activities in high floristic diversity areas: the case of Mt Giona ». In . Milos island, Greece. http://publicationslist.org/data/kgeorghi/ref-41/%CE%9419.%20Floristic%20diversity%20Giona.pdf.

Manns, Ulrika, et Arne A. Anderberg. 2005. « Molecular Phylogeny of Anagallis (Myrsinaceae) Based on ITS, trnL-F, and ndhF Sequence Data ». *International Journal of Plant Sciences* 166 (6): 1019-28. https://doi.org/10.1086/449318.

Ranunculaceae

Adonis

Delitheos, A., E. Tiligada, A. Yannitsaros, et I. Bazos. 1997. « Antiphage Activity in Extracts of Plants Growing in Greece ». *Phytomedicine* 4 (2): 117-24. https://doi.org/10.1016/S0944-7113(97)80055-4.

Greuter, Werner. 1994. « Extinctions in Mediterranean Areas ». *Philosophical Transactions: Biological Sciences* 344 (1307): 41-46.

Löve, Áskell. 1986. « Chromosome Number Reports XCIII ». *Taxon* 35 (4): 897-903.

Anemone

Bareka, P., G. Kamari, N.J. Turland, and D. Phitos. 2015. 'Karyomorphological Study of Some Cretan Archeophytes'. *Flora Mediterranea* 25 (Special Issue). https://doi.org/10.7320/FlMedit25SI.127.

Muzychuk, G. M., and O. P. Pereboichuk. 2009. 'Ornamental Plants of the Genus Anemone L. in the World Cultivated Flora and the Perspectives of Their Introduction in Ukraine'. *Plant Introduction* 44 (December): 29–41. https://doi.org/10.5281/zenodo.2555371.

Turland, Nicholas, Dimitrios Phitos, Georgia Kamari, and Pepy Bareka. 2004. 'Weeds of the Traditional Agriculture of Crete'. *Willdenowia* 34 (2): 381–406. https://doi.org/10.3372/wi.34.34206.

Aquilegia

Fior, Simone, Mingai Li, Bengt Oxelman, Roberto Viola, Scott A. Hodges, Lino Ometto, et Claudio Varotto. 2013. « Spatiotemporal Reconstruction of the Aquilegia Rapid Radiation through Next-Generation Sequencing of Rapidly Evolving CpDNA Regions ». *New Phytologist* 198 (2): 579-92. https://doi.org/10.1111/nph.12163.

Kyriakopoulos, Charalampos, et Georgia Kamari. 2016. « The Rediscovery of Aquilegia Ottonis Subsp. Taygetea (Ranunculaceae), an Endemic Taxon of S. Peloponnisos, Greece », 9.

Tan, Kit, Giannis Kofinas, and Gioula Drolapa. 2024. 'The Taxonomic Status of an Aquilegia (Ranunculaceae) Rediscovered in Sterea Ellas, South Central Greece'. *Phytologia Balcanica* 30 (2): 167–72.

Μπάντη, Αντωνία Δημητρίου. 2012. *Σπάνια και απειλούμενα φυτά της Ελλάδας: κατανομή, απειλές και κατάσταση διατήρησης in situ και ex situ - Rare and threatened plants of Greece: distribution, threats and conservation status in situ and ex situ*.

Consolida

Baliousis, Evagelos, and Artemios Yannitsaros. 2011. 'Vascular Plant Diversity of Mt Pendelikon (Sterea Ellas, Greece): A Recent Inventory Reflecting Contemporary Dynamics'. *Willdenowia* 41 (1): 151–65. https://doi.org/10.3372/wi.41.41119.

Bergmeier, E., M. Ristow, S. Meyer, and M. Panitsa. 2021. 'Phytodiversity of Limnos (North Aegean, Greece)—an Update and Evaluation'. *Flora Mediterranea* 31: 233–46. https://doi.org/10.7320/FlMedit31.233.

Carlström, Annette. 1984. 'New Species of Alyssum, Consolida, Origanum and Umbilicus from the SE Aegean Sea'. *Willdenowia* 14 (1): 15–26. JSTOR.

De Montmollin, Bertrand, and Wendy Strahm. 2005. *The Top 50 Mediterranean Island Plants: Wild Plants at the Brink of Extinction, and What Is Needed to Save Them*. IUCN.

Minareci, E., Y. Altan, and T. Aktan. 2011. 'A New Record from Turkey: Consolida Samia PH Davis (Ranunculaceae).' *JAPS, Journal of Animal and Plant Sciences* 21 (3): 552–55.

Strid, Arne. 2007. 'Lost and Found in the Greek Flora'. 16–20.

Thanos, Costas A. 2014. 'In Situ and Ex Situ Plant Conservation in Greece in the Framework of the Global Strategy for Plant Conservation'. *Bullettin of the Gioenia Academy of Natural Sciences of Catania* 47 (377/SFE): 377/SFE.

Delphinium

Kamari, Georgia, Pepy Bareka, Theophanis Constantinidis, et Dimitrios Phitos. 2003. « Karyosystematic studies of plant taxa from the East Mediterranean region (Grece, Cyprus, Syria) ». *Phytologia balcanica* 9 (3): 487-502.

Siljak-Yakovlev, Sonja, Perla Farhat, Nicolas Valentin, Pepy Bareka, et Georgia Kamari. 2019. « New Estimates of Nuclear DNA Amount for 25 Taxa from Kefallinia Island », 23.

Nigella

Bazos, Ioannis, Ioannis P. Kokkoris, et Panayotis Dimopoulos. 2021. « Diversity of Halophytes and Salt Tolerant Plants at the Species-, Habitats- and High-Rank Syntaxa Level in Greece ». In *Handbook of Halophytes*, édité par Marius-Nicusor Grigore, 787-820. Cham: Springer International Publishing. https://doi.org/10.1007/978-3-030-57635-6_26.

Marks, G. E. 1977. « The Nature of Centromeric Dots in Nigella Chromosomes ». *Chromosoma* 62 (4): 369-73. https://doi.org/10.1007/BF00327035.

Strid, Arne. 1968. « Stable Telocentric Chromosomes formed by spontaneous misdivision in Nigella doerfleri (Ranunculaceae) ». *Bot. Notiser.* 121.

Ranunculus

Baltisberger, Matthias. 1994. 'Ranunculus Cacuminis andR. Crenatus, Representatives of theR. Alpestris-Group (Ranunculaceae) on the Balkan Peninsula'. *Plant Systematics and Evolution* 190 (3): 231–44. https://doi.org/10.1007/BF00986195.

Baltisberger, Matthias, and Elvira Hörandl. 2016. 'Karyotype Evolution Supports the Molecular Phylogeny in the Genus *Ranunculus* (Ranunculaceae)'. *Perspectives in Plant Ecology, Evolution and Systematics* 18 (February): 1–14. https://doi.org/10.1016/j.ppees.2015.11.001.

Baltisberger, Matthias, and Alex Widmer. 2005. 'Cytological Investigations on Some Ranunculus-Species from Crete.' *Candollea* 60 (2): 335–44.

Böhling, Niels. 2000. 'Ranunculus Veronicae (Ranunculaceae), a New Species from W Crete (Greece)'. *Willdenowia* 30 (2): 245–50. https://doi.org/10.3372/wi.30.30203.

Dunkel, Franz G. 2015. 'Ranunculus Pindicola Sp. Nov., the Only Species of the R. Auricomus Complex (Ranunculaceae) in Greece'. *Willdenowia* 45 (2): 223–30. https://doi.org/10.3372/wi.45.45208.

Greuter, W., and A. Strid. 1981. 'Notes on Cardaegean Plants. 2. A New Species of Ranunculus Sect. Ranunculus from the Mountains of W Kriti'. *Willdenowia* 11 (2): 267–69.

Jopek, Magdalena, Gerhard Wiegleb, Wiesław Babik, and Joanna Zalewska-Gałosz. 2023. '*Ranunculus Dahlgreniae* (Section *Batrachium*, Ranunculaceae), a New Species from Crete, Greece, with Remarks on Taxonomy and Phylogenetic Relations within the Section'. *Acta Societatis Botanicorum Poloniae*, ahead of print, July 18. https://doi.org/10.5586/asbp/167462.

Kamari, Georgia, Dimitrios Phitos, Britt Snogerup, and Sven Snogerup. 1988. 'Flora and Vegetation of Yioura, N Sporades, Greece'. *Willdenowia* 17 (1/2): 59–85. JSTOR.

Krigas, Nikos, Eleftherios Karapatzak, Marina Panagiotidou, et al. 2022. 'Prioritizing Plants around the Cross-Border Area of Greece and the Republic of North Macedonia: Integrated Conservation Actions and Sustainable Exploitation Potential'. *Diversity* 14 (7): 7. https://doi.org/10.3390/d14070570.

Krigas, Nikos, Georgios Tsoktouridis, Ioannis Anestis, et al. 2021. 'Exploring the Potential of Neglected Local Endemic Plants of Three Mediterranean Regions in the Ornamental Sector: Value Chain Feasibility and Readiness Timescale for Their Sustainable Exploitation'. *Sustainability* 13 (5): 5. https://doi.org/10.3390/su13052539.

Turland, Nicholas J., and Lance Chilton. 1994. 'Studies on the Cretan Flora 3. Additions to the Flora of Karpathos'. *Bulletin of the Natural History Museum London (Botany)* 24 (1): 91–99.

Rhamnaceae

Rhamnus

Korakaki, E., A. Legakis, S. Katsanevakis, P. P. Koulelis, E. V. Avramidou, N. Soulioti, et P. V. Petrakis. 2021. « Invasive Alien Species of Greece ». In *Invasive Alien Species*, 124-89. John Wiley & Sons, Ltd. https://doi.org/10.1002/9781119607045.ch29.

Rosaceae

Amelanchier

Bavela, S., S. M. Dimitrakoudi, A. Angelis, M. Michailidou, E. Loutrari, A. L. Skaltsounis, et N. Aligiannis. 2014. « Phytochemical Analysis of Amelanchier Parviflora Subsp. Chelmea and Identification of Their Anti-Angiogenic Secondary Metabolites ». *Planta Medica* 80 (16): P1L119. https://doi.org/10.1055/s-0034-1394776.

Crataegus

Agalou, Adamantia, Michael Thrapsianiotis, Apostolis Angelis, Athanasios Papakyriakou, Alexios-Leandros Skaltsounis, Nektarios Aligiannis, et Dimitris Beis. 2018. « Identification of Novel Melanin Synthesis Inhibitors From Crataegus Pycnoloba Using an in Vivo Zebrafish Phenotypic Assay ». *Frontiers in Pharmacology* 0. https://doi.org/10.3389/fphar.2018.00265.

Drymocallis

Doumas, Panayiotis, Katerina Goula, and Theophanis Constantinidis. 2022. 'Thirty-Two New and Noteworthy Floristic Records from North-Eastern Greece'. *Biodiversity Data Journal* 10 (April): e81817. https://doi.org/10.3897/BDJ.10.e81817.

Potentilla

Brofas, G., Panayiotis Trigas, G. Mantakas, et al. 2007. 'Rehabilitation of Disturbed Areas by Mining Activities in High Floristic Diversity Areas: The Case of Mt Giona'. Paper presented at 3rd International Conference on Sustainable Development Indicators in the Mineral Industry, Milos Island, Greece. June. http://publicationslist.org/data/kgeorghi/ref-41/%CE%9419.%20Floristic%20diversity%20Giona.pdf.

Iatrou, Gregoris. 1985. « A new species of Potentilla (Rosaceae) from Peloponnesus, Greece ». *Candollea* 40 (1): 121-28.

Kyriakopoulos, Ch., G. Kamari, I. Kofinas, and D. Phitos. 2018. 'Potentilla Greuteriana (Rosaceae), a New Species from Mt. Taigetos (S Peloponnisos, Greece)'. *Flora Mediterranea* 28 (December): 351–64. https://doi.org/10.7320/FlMedit28.351.

Sanguisorba

Sarropoulou, Virginia, Nikos Krigas, Georgios Tsoktouridis, Eleni Maloupa, and Katerina Grigoriadou. 2022. 'Seed Germination Trials and Ex Situ Conservation of Local Prioritized Endemic Plants of Crete (Greece) with Commercial Interest'. *Seeds* 1 (4): 4. https://doi.org/10.3390/seeds1040024.

Rubiaceae

Asperula

Aplada, E., Th Georgiadis, A. Tiniakou, and M. Theocharopoulos. 2007. 'Phytogeography and Ecological Evaluation of the Flora and Vegetation of Mt Parnitha (Attica, Greece)'. *Edinburgh Journal of Botany* 64 (2): 185–207. https://doi.org/10.1017/S096042860700087X.

Bagkou, Evangelia, Nikos Krigas, et Despoina Vokou. 2014. « Rare and Threatened Plants of Greece Not Protected in Situ ». In , 234-35. Ioannina.

Carlström, A. 1986. 'New Taxa and Notes from the SE Aegean Area and SW Turkey'. *Willdenowia* 16: 73–78.

Christodoulakis, Dimitris, and Theodoros Georgiadis. 1984. 'Eine Neue Asperula-Art (Rubiaceae) von Der Insel Samos, Griechenland'. *Willdenowia* 13 (2): 341–44.

Constantinidis, Theophanis, Georgia Kamari, and Dimitrios Phitos. 1997. 'A Cytological Study of 28 Phanerogams from the Mountains of SE Sterea Ellas, Greece'. *Willdenowia* 27 (1/2): 121–42.

Gargiulo, Roberta. 2013. 'Phylogeny of Asperula L. Sect. Cynanchicae (DC.) Boiss. (Rubiaceae)'. PhD in biology, University of Naples 'Frederico II'.

Goulimis, C. 1958. *Report on Species of Plants Requiring Protection in Greece and Measures for Securing Their Protection*. Athens. http://documents.irevues.inist.fr/bitstream/handle/2042/59422/LATERREETLAVIE_1959_Sup_168.pdf?sequence=1.

Govaerts, Rafaël H.A. 2018. « 101 nomenclatural corrections in preparation for the Plants of the World Online (POWO) ». *Skovortsovia* 4 (3): 74-99.

Gregor, Thomas, Lenz Meierott, and Thomas Raus. 2016. 'Asperula Tymphaea (Rubiaceae) – a New Species from Northern Pindus, Greece'. *Phytologia Balcanica* 22 (2): 255–58.

Greuter, Werner, Ursula Matthäs, and Horst Risse. 1985. 'Additions to the Flora of Crete, 1973-1983 (1984) - II'. *Willdenowia* 14 (2): 269–97.

Gutermann, Walter, Tae-Soo Jang, Arndt Kästner, et al. 2024. 'Thliphthisa Sapphus (Rubiaceae, Rubieae), a New Species from Lefkada (Ionian Islands, Greece) and Its Ecological Position'. *PhytoKeys* 241 (April): 65. https://doi.org/10.3897/phytokeys.241.119144.

Iatrou, Gregory, and Theodore Georgiadis. 1984. 'A New Species of Asperula (Rubiaceae) from Peloponnesos, Greece'. *Willdenowia* 14 (1): 55–59.

Kalpoutzakis, Eleftherios, et Theophanis Constantinidis. 2005. « New data on the distribution of endemic and rare taxa in the flora of east Peloponnisos, Greece ». *Botanika Chronika* 18 (2).

Krigas, Nikos, Georgios Tsoktouridis, Ioannis Anestis, et al. 2021. 'Exploring the Potential of Neglected Local Endemic Plants of Three Mediterranean Regions in the Ornamental Sector: Value Chain Feasibility and Readiness Timescale for Their Sustainable Exploitation'. *Sustainability* 13 (5): 5. https://doi.org/10.3390/su13052539.

Löve, Áskell. 1981. « Chromosome Number Reports LXXIII ». *Taxon* 30 (4): 829-61.

Özdemir, Ayse. 2007. « KAYA BAHÇESİ: DOĞRU TESİS, BİTKİLENDİRME VE BAKIM - Rock garden: the right plant, growing conditions and care. », 9.

Quezel, P. 1967. 'La Végétation Des Hauts Sommets Du Pinde et de l'Olympe de Thessalie'. *Vegetatio*, 127–226.

Schönbeck-Temesy, Eva, and Friedrich Ehrendorfer. 1980. 'New Endemic Taxa of Asperula Sect. Cynanchicae (Rubiaceae) from the East Aegean Islands'. *Plant Systematics and Evolution* 134 (1/2): 133–35.

Trigas, Panayiotis, and Gregoris Iatrou. 2006. 'The Local Endemic Flora of Evvia (W Aegean, Greece)'. *Willdenowia* 36 (1): 257–70. https://doi.org/10.3372/wi.36.36121.

Trigas, Panayiotis, and Gregory Iatrou. 2003. 'Asperula (Sect. Cynanchicae) Brachyphylla, Spec, Nova (Rubiaceae) from the Island of Evvia (Greece)'. *Phyton (Horn, Austria)* 43 (1): 29–37.

Tzakou, Olga, Konstantinos Lempesis, et Anargyros Loukis. 2011. « Secondary Metabolites from Asperula Lutea Subsp. Rigidula ». *Natural Product Communications* 6 (2): 1934578X1100600. https://doi.org/10.1177/1934578X1100600220.

Valli, Anna-Thalassini, Vassiliki Lila Koumandou, Gregoris Iatrou, Marios Andreou, Vasileios Papasotiropoulos, and Panayiotis Trigas. 2021. 'Conservation Biology of Threatened Mediterranean Chasmophytes: The Case of Asperula Naufraga Endemic to Zakynthos Island (Ionian Islands, Greece)'. *PLOS ONE* 16 (2): e0246706. https://doi.org/10.1371/journal.pone.0246706.

Van Den Beuken, Ger, Cliff Booker, Thelma Hewitt, Hugh MacMillan, David Sellars, Abbie Zabar, et Kees Jan van Zwienen. 2010. « Rock Garden Quarterly ». *Bulletin of the North American Rock Garden Society*, 2010. https://www.nargs.org/sites/default/files/free-rgq-downloads/VOL_69_NO_1.pdf.

Ανδριόπουλος, Παύλος. 2011. 'Η καταλληλότητα ενδιαιτήματος ως οικολογική παράμετρος στη διατήρηση ειδών προτεραιότητας προστασίας: η περίπτωση της Asperula baenitzii Heldr. ex Boiss'. Διδακτορική Διατριβή, Εθνικό και Καποδιστριακό Πανεπιστήμιο Αθηνών (ΕΚΠΑ). Σχολή Θετικών Επιστημών. Τμήμα Βιολογίας. Τομέας Οικολογίας και Ταξινομικής. http://hdl.handle.net/10442/hedi/35400.

Cruciata

Trigas, Panayiotis, Gregoris Iatrou, et Maria Panitsa. 2008. « Vascular Plant Species Diversity, Biogeography and Vulnerability in the Aegean Islands as Exemplified by Evvia Island (West Aegean, Greece) ». *Fresenius Environmental Bulletin* 17 (1): 11.

Galium

Burton, Rodney. 1996. 'Two New Flowering Plant Species for the Flora of Crete'. *Flora Mediterranea* 6: 69–70.

Greuter, Werner, and Thomas Raus. 2010. 'Med-Checklist Notulae, 28'. *Willdenowia* 39 (2): 335–45. https://doi.org/10.3372/wi.39.39211.

Kougioumoutzis, K., A. Tiniakou, O. Georgiou, and T. Georgiadis. 2015. 'Contribution to the Flora and Biogeography of the Kiklades: Folegandros Island (Kiklades, Greece)'. *Edinburgh Journal of Botany* 72 (3): 391–412. https://doi.org/10.1017/S0960428615000128.

Krigas, Nikos, Marina Panagiotidou, et Eleni Maloupa. 2017. « Incorporating biogeographical principles in horticulture: design and creation of the ionian islands unique rock garden in Thessaloniki, Greece. » *Sibbaldia: The Journal of Botanic Garden Horticulture*, 2017, 15 édition.

Sobhian, R., A. McClay, S. Hasan, M. Peterschmitt, and R. B. Hughes. 2004. 'Safety Assessment and Potential of Cecidophyes Rouhollahi (Acari, Eriophyidae) for Biological Control of Galium Spurium (Rubiaceae) in North America'. *Journal of Applied Entomology* 128 (4): 258–66. https://doi.org/10.1111/j.1439-0418.2004.00818.x.

Strid, Arne. 2015. 'Reliquiae Runemarkianae. Chromosome Numbers of Angiosperms from the Aegean Islands'. *Phytologia Balcanica* 21 (3): 245–93.

Tzakou, Olga, Philippos Mylonas, Constantinos Vagias, et Panos V. Petrakis. 2007. « Iridoid Glucosides with Insecticidal Activity from Galium Melanantherum ». *Zeitschrift Für Naturforschung C* 62 (7-8): 597-602. https://doi.org/10.1515/znc-2007-7-823.

Τζάκου, Όλγα. 1988. « ΜΕΛΕΤΗ ΤΩΝ ΧΗΜΙΚΩΝ ΣΥΣΤΑΤΙΚΩΝ ΤΟΥ ΦΥΤΟΥ GALIUM MELANANTHERUM BOISS - Study of chemical components of the Galium melanantherum Boiss. species ». Διδακτορική Διατριβή, Εθνικό και Καποδιστριακό Πανεπιστήμιο Αθηνών (ΕΚΠΑ). Σχολή Επιστημών Υγείας. Τμήμα Φαρμακευτικής. http://hdl.handle.net/10442/hedi/0746.

Zogaris, Stamatis, and Basiliki Blami. 2011. *Προτάσεις Οικοτουριστικής Ανάδειξης Με Σκοπό Τη Διατήρηση Της Παράκτιας Βιοποικιλότητας Της Θάσου. Μοσίευτη Έκθεση Προς Δήμο Θάσου & Περιφέρεια Ανατολικής Μακεδονίας Και Θράκης. - Ecotourism Promotion Proposals in Order to Preserve the Coastal Biodiversity of Thassos*. Municipality of Thassos. https://www.researchgate.net/profile/Stamatis-Zogaris/publication/259292233_Ecotourism_proposals_for_the_promotion_of_coastal_biodiversity_conservation_on_Thassos_Greece/data/0046352aca7afa51a6000000/thassos-z-v-final2011.pdf.

Πλαϊτη, Ειρηνη. 2018. 'Μελετη πρασινου των κοινοχρηστων χωρων του οικισμου τραπεζοϋπαλληλων νομου Ρεθυμνου'. Bachelor Thesis, TEI of Epirus.

Salicaceae

Salix

Cambria, Salvatore, Cristian Brullo, and Salvatore Brullo. 2019. 'Salix Kaptarae Sp. Nov. (Salicaceae) from Crete'. *Nordic Journal of Botany* 37 (7). https://doi.org/10.1111/njb.02335.

Sciandrello, Giuseppe Siracusa, and Salvatore Brullo. 2020. 'Salix Aegaea (Salicaceae), a New Species from Island of Ikaria (Greece)'. *Phytotaxa* 447 (2): 127–36.

Wagner, Natascha D., Pia Marinček, Loïc Pittet, and Elvira Hörandl. 2023. 'Insights into the Taxonomically Challenging Hexaploid Alpine Shrub Willows of Salix Sections Phylicifoliae and Nigricantes (Salicaceae)'. *Plants* 12 (5): 5. https://doi.org/10.3390/plants12051144.

Scrophulariaceae

Scrophularia

Bigazzi, Massimo, and Federico Selvi. 2000. 'Anchusa Samothracica (Boraginaceae), a New Species from the Island of Samothraki, Greece'. *Nordic Journal of Botany* 20 (2): 141–48. https://doi.org/10.1111/j.1756-1051.2000.tb01557.x.

Goulimis, C. 1958. *Report on Species of Plants Requiring Protection in Greece and Measures for Securing Their Protection*. Athens. http://documents.irevues.inist.fr/bitstream/handle/2042/59422/LATERREETLAVIE_1959_Sup_168.pdf?sequence=1.

Verbascum

Axiotis, Evangelos, Maria Halabalaki, and Leandros A. Skaltsounis. 2018. 'An Ethnobotanical Study of Medicinal Plants in the Greek Islands of North Aegean Region'. *Frontiers in Pharmacology* 9. https://doi.org/10.3389/fphar.2018.00409.

Catara, Stefania, Antonia Cristaudo, Andrea Gualtieri, Rosario Galesi, Carmen Impelluso, and Andrea Onofri. 2016. 'Threshold Temperatures for Seed Germination in Nine Species of Verbascum (Scrophulariaceae)'. *Seed Science Research* 26 (1): 30–46. https://doi.org/10.1017/S0960258515000343.

Ganatsas, Petros, Marianthi Tsakaldimi, Christos Damianidis, et al. 2019. 'Regeneration Ecology of the Rare Plant Species Verbascum

Dingleri: Implications for Species Conservation’. *Sustainability* 11 (12): 12. https://doi.org/10.3390/su11123305.

Goulimis, C. 1958. « Report on species of plants requiring protection in Greece and measures for securing their protection ». Athens. http://documents.irevues.inist.fr/bitstream/handle/2042/59422/LATERREETLAVIE_1959_Sup_168.pdf?sequence=1.

Hameed, Ayesha, Muhammad Zafar, Riaz Ullah, Abdelaaty A. Shahat, Mushtaq Ahmad, Saman I. Cheema, Lubna, et al. 2020. « Systematic Significance of Pollen Morphology and Foliar Epidermal Anatomy of Medicinal Plants Using SEM and LM Techniques ». *Microscopy Research and Technique* 83 (8): 1007-22. https://doi.org/10.1002/jemt.23493.

Hopper, Stephen D. 2023. ‘Ocbil Theory as a Potential Unifying Framework for Investigating Narrow Endemism in Mediterranean Climate Regions’. *Plants* 12 (3): 645.

Kalpoutzakis, Elefterios, Nektarios Aligiannis, Sofia Mitakou, and Alexios-Leandros Skaltsounis. 1999. ‘Verbaspinoside, a New Iridoid Glycoside from Verbascum Spinosum’. *Journal of Natural Products* 62 (2): 342–44. https://doi.org/10.1021/np980351f.

Manetas, Y. 2003. « The Importance of Being Hairy: The Adverse Effects of Hair Removal on Stem Photosynthesis of Verbascum speciosum Are Due to Solar UV-B Radiation ». *The New Phytologist* 158 (3): 503-8.

Pachschwöll, Clemens, Dieter Reich, and Kit Tan. 2019. ‘The Botanical Legacy of Mihael Dimonie (1870–1935), an Almost Forgotten Plant Collector in the Southern Balkan Peninsula before the First World War’. *Willdenowia* 49 (2): 257–79. https://doi.org/10.3372/wi.49.49213.

Park, Jong Cheol. 2017. « 파리식물원 982종 약용식물의 조사 연구 - Research Study on 982 Medicinal Plants in Paris Plant Garden ». *Korean Herb. Med. Inf.* http://herba.kr/khmi/j/05(1)/KHMI-05(1)-01.pdf.

Paschalidis, Konstantinos, Dimitrios Fanourakis, Georgios Tsaniklidis, et al. 2021. ‘Pilot Cultivation of the Vulnerable Cretan Endemic Verbascum Arcturus L. (Scrophulariaceae): Effect of Fertilization on Growth and Quality Features’. *Sustainability* 13 (24): 24. https://doi.org/10.3390/su132414030.

Regel, Constantin. 1948. ‘Floristische Notizen aus Griechenland: Florae Graecae Notulae III’. *Österreichische Botanische Zeitschrift* 95 (3): 243–50. https://doi.org/10.1007/BF01249966.

Tan, Kit, and Aris Zografidis. 2014. ‘On the Identity of Carduus Euboicus (Asteraceae) from Mt Dirphys, Evvia, Greece’. *Phytotaxa* 161 (3): 194. https://doi.org/10.11646/phytotaxa.161.3.2.

Trigas, Panayiotis, and Gregoris Iatrou. 2006. ‘The Local Endemic Flora of Evvia (W Aegean, Greece)’. *Willdenowia* 36 (1): 257–70. https://doi.org/10.3372/wi.36.36121.

Turland, Nicholas J. 2006. ‘Lectotypification of Campanula Saxatilis, Phyteuma Pinnatum and Verbascum Arcturus, Linnaean Names of Three Taxa Endemic to Crete’. *Willdenowia* 36 (1): 303–9.

Vladimirov, Vladimir, Mehmet Aybeke, Vlado Matevski, and Kit Tan. 2017. ‘New Floristic Records in the Balkans: 34’. *Phytologia Balcanica* 23 (3): 413–44.

Voliotis, Dimitrios T. 1987. ‘Die natürliche flora von Griechenland’. *Acta Botanica Croatica* 46: 213–24.

Zografidis, Aris. 2016. « Two new infraspecific taxa of Verbascum delphicum (Scrophulariaceae, Scrophularieae) from mainland Greece and the island of Evvia ». *PhytoKeys*, n° 74 (novembre): 107-22. https://doi.org/10.3897/phytokeys.74.10381.

Zografidis, Aris, Eleni Liveri, Vasilis Ioannidis, and Panayotis Dimopoulos. 2022. ‘Verbascum Salicifolium (Scrophulariaceae) a New Species from Central Macedonia, Greece’. *Phytotaxa* 552 (3): 3. https://doi.org/10.11646/phytotaxa.552.3.2.

Zografidis, Aris, Kostas Polymenakos, George Zarkos, et Panayotis Dimopoulos. 2020. « Notes on Verbascum (Scrophulariaceae) from Greece » 26 (3): 485-94.

Zografidis, Aris, et Arne Strid. 2017. « A Taxonomic Revision of the Verbascum Daenzeri Group (Scrophulariaceae) » 23 (1): 65-71.

Tamaricaceae

Tamarix

Villar, Jose L., Nicholas J. Turland, Ana Juan, John F. Gaskin, Ángeles Alonso, and Manuel B. Crespo. 2015. ‘Tamarix Minoa (Tamaricaceae), a New Species from the Island of Crete (Greece) Based on Morphological and Plastid Molecular Sequence Data’. *Willdenowia* 45 (2): 161–72.

Thesicaceae

Thesium

Aldén, Björn. 1981. ‘Thesium Vlachorum Sp. Nov.’ *Nordic Journal of Botany* 1 (6): 709–10. https://doi.org/10.1111/j.1756-1051.1981.tb01156.x.

Selina Wamuccii. 2024. ‘Thesium Vlachorum - Uses, Benefits & Care’. Agrocompany website. *Selina Wamucii*. https://www.selinawamucii.com/plants/santalaceae/thesium-vlachorum/.

Ulmaceae

Zelkova

Kozlowski, Gregor, David Frey, Laurence Fazan, et al. 2014. ‘The Tertiary Relict Tree Zelkova Abelicea (Ulmaceae): Distribution, Population Structure and Conservation Status on Crete’. *Oryx* 48 (1): 80–87. https://doi.org/10.1017/S0030605312001275.

Krigas, Nikos, Viktoria Menteli, and Despoina Vokou. 2014. ‘The Electronic Trade in Greek Endemic Plants: Biodiversity, Commercial and Legal Aspects’. *Economic Botany* 68 (1): 85–95. https://doi.org/10.1007/s12231-014-9264-9.

Mediterranean Agronomic Institute of Chania (MAICH). 2024. 'Zelkova Abelicea Conservation of Zelkova Abelicea in Crete (2017–2020).' Abelitsia.Gr. http://www.abelitsia.gr/en/.

Menteli, Viktoria, Nikos Krigas, Manolis Avramakis, Nicholas Turland, and Despoina Vokou. 2019. 'Endemic Plants of Crete in Electronic Trade and Wildlife Tourism: Current Patterns and Implications for Conservation'. *Journal of Biological Research-Thessaloniki* 26 (1): 10. https://doi.org/10.1186/s40709-019-0104-z.

Valerianaceae

Centranthus

Krigas, Nikos, Georgios Tsoktouridis, Ioannis Anestis, et al. 2021. 'Exploring the Potential of Neglected Local Endemic Plants of Three Mediterranean Regions in the Ornamental Sector: Value Chain Feasibility and Readiness Timescale for Their Sustainable Exploitation'. *Sustainability* 13 (5): 5. https://doi.org/10.3390/su13052539.

Teofilovski, Aco. 2018. « Chorological Data for Some New, Doubtfully Known and Rare Plants in the Flora of the Republic of Macedonia ». *Acta Musei Macedonici Scientiarum Naturalium* 21 (1): 13-22.

Valeriana

Mahmutaj, Ermelinda, Lulzim Shuka, Murat Xhulaj, Petrit Hoda, et Mersin Mersinllari. 2014. « Rare and Endemic Plants in the Southern Mountain Ecosystems of Albania, Their Threats and Diversity », 10.

Raab-Straube, Eckhard Von, and Thomas Raus. 2015. 'Euro Med-Checklist Notulae, 5'. *Willdenowia* 45 (3): 449–64. https://doi.org/10.3372/wi.45.45312.

Veronicaceae

Cymbalaria

Krigas, Nikos, Marina Panagiotidou, et Eleni Maloupa. 2017. « Incorporating biogeographical principles in horticulture: design and creation of the ionian islands unique rock garden in Thessaloniki, Greece. » *Sibbaldia: The Journal of Botanic Garden Horticulture*, 2017, 15 édition.

Maloupa, E, N Krigas, et A Karydas. 2007. « The in Situ Plant Conservation Actions of the Balkan Botanic Garden of Kroussia in Greece », 5.

Panitsa, Maria, Panayiotis Trigas, Dimitrios Kontakos, Anna-Thalassini Valli, et Gregoris Iatrou. 2021. « Natural and cultural heritage interaction: aspects of plant diversity in three East Peloponnesian castles (Greece) and conservation evaluation ». *Plant Biosystems - An International Journal Dealing with all Aspects of Plant Biology* 0 (0): 1-15. https://doi.org/10.1080/11263504.2021.1889701.

Samaropoulou, S., P. Bareka, R. Artelari, et G. Kamari. 2013. « Karyological Studies on Some Endemic and Rare Species of Kefalonia, Ionian Islands, Greece ». *Flora Mediterranea* 23 (décembre). https://doi.org/10.7320/FlMedit23.215.

Veronica

Bardy, Katharina E., Dirk C. Albach, Gerald M. Schneeweiss, Manfred A. Fischer, et Peter Schönswetter. 2010. « Disentangling Phylogeography, Polyploid Evolution and Taxonomy of a Woodland Herb (Veronica Chamaedrys Group, Plantaginaceae s.l.) in Southeastern Europe ». *Molecular Phylogenetics and Evolution* 57 (2): 771-86. https://doi.org/10.1016/j.ympev.2010.06.025.

Jensen, Søren Rosendal, Dirk C. Albach, Takao Ohno, et Renée J. Grayer. 2005. « Veronica: Iridoids and Cornoside as Chemosystematic Markers ». *Biochemical Systematics and Ecology* 33 (10): 1031-47. https://doi.org/10.1016/j.bse.2005.03.001.

Krigas, Nikos, Marina Panagiotidou, et Eleni Maloupa. 2017. « Incorporating biogeographical principles in horticulture: design and creation of the ionian islands unique rock garden in Thessaloniki, Greece. » *Sibbaldia: The Journal of Botanic Garden Horticulture*, 2017, 15 édition.

Meudt, Rojas-Andrés, Prebble, Low, Garnock-Jones, et Albach. 2015. « Is genome downsizing associated with diversification in polyploid lineages of Veronica (Plantaginaceae) - Supplementary Table S1. Veronica vouchers for genome size and DNA sequence data used in this study and in previous studies. » *Bot. J. Linn. Soc.* https://www.researchgate.net/profile/Heidi-Meudt/publication/276919921_Meudt_et_al_2015_Botanical_Journal_of_the_Linnean_Society_Tab_S1/links/555b8d8c08ae8f66f3ad7ab1/Meudt-et-al-2015-Botanical-Journal-of-the-Linnean-Society-Tab-S1.pdf.

Violaceae

Viola

Ballard, H. E., J. de Paula-Souza, et G. A. Wahlert. 2014. « Violaceae ». In *Flowering Plants. Eudicots: Malpighiales*, édité par Klaus Kubitzki, 303-22. The Families and Genera of Vascular Plants. Berlin, Heidelberg: Springer. https://doi.org/10.1007/978-3-642-39417-1_25.

Bareka, P., E. Katopodi, G. Kamari, and D. Phitos. 2018. 'Karyosystematic Study of Some Taxa from the Ionian Floristic Region (Greece). I'. *Flora Mediterranea* 28. https://doi.org/10.7320/FlMedit28.085.

Davis, Peter H. 1938. 'The Flowers of Lasithi'. *The Annual of the British School at Athens* 38 (November): 146–48. https://doi.org/10.1017/S0068245400012107.

Diapoulis, Ch. 1959. 'Conservation Measures for the Plants of the Greek Flora.' http://documents.irevues.inist.fr/bitstream/handle/2042/59423/LATERREETLAVIE_1959_Sup_189.pdf?sequence=1.

Goulimis, C. 1958. *Report on Species of Plants Requiring Protection in Greece and Measures for Securing Their Protection*. Athens. http://documents.irevues.inist.fr/bitstream/handle/2042/59422/LATERREETLAVIE_1959_Sup_168.pdf?sequence=1.

Grigoriadis, Nikolaos, and Elena Kmetova. 2006. 'Rhodope Mountains: The Green Belt between Greece and Bulgaria'. In *The*

Green Belt of Europe: From Vision to Reality, edited by Andrew Terry, Karin Ullrich, and Uwe Riecken. IUCN.

Franzén, R., et L.-Å. Gustavsson. 1983. « Chromosome Numbers in Flowering Plants from the High Mountains of Sterea Ellas, Greece ». *Willdenowia* 13 (1): 101-6.

Karydas, Antony, and Georgia Kamari. 2019. 'Monitoring Six Local Endemic Taxa of the Mt Athos and Assessment According to the IUCN Red List Categories and Criteria'. *Botanika Chronika* 22: 195–208.

Krigas, Nikos, Eleftherios Karapatzak, Marina Panagiotidou, et al. 2022. 'Prioritizing Plants around the Cross-Border Area of Greece and the Republic of North Macedonia: Integrated Conservation Actions and Sustainable Exploitation Potential'. *Diversity* 14 (7): 7. https://doi.org/10.3390/d14070570.

Krigas, Nikos, Marina Panagiotidou, and Eleni Maloupa. 2017. 'Incorporating Biogeographical Principles in Horticulture: Design and Creation of the Ionian Islands Unique Rock Garden in Thessaloniki, Greece'. *Sibbaldia: The Journal of Botanic Garden Horticulture* 0 (15): 129–46.

Maloupa, Eleni, Nikos Krigas, Katerina Grigoriadou, Diamanto Lazari, and Georgios Tsoktouridis. 2008. *Conservation Strategies for Native Plant Species and Their Sustainable Exploitation: Case of the Balkan Botanic Garden of Kroussia, N. Greece*. 21.

Mertzanis, Aristeidis, Stavroula Syleouni, Konstantinos Mertzanis, et Stamatis Zogaris. 2016. « Ecotourism Promotion in a Greek National Part: The Development and Management of Farmakides Trail on Mt Oiti » 10: 26.

Niketić, Marjan, Pavle Cikovac, Zoltán Barina, Dániel Pifkó, Ljupčo Melovski, Šemija Duraki, et Gordana Tomović. 2015. « Viola chelmea and Viola jooi (Violaceae), new species for the flora of Serbia and their distribution in the Balkan Peninsula and the Carpathians ». *Bulletin of the Natural History Museum*, nº 8: 49-74. https://doi.org/10.5937/hnhmb1508049N.

Papanicolaou, Kostas. 1978. 'On the Flora of the Athos Peninsula I. Biosystematics of Viola Athois'. *Sci. Annals, Fac. Phys. & Mathem., Univ. Thessaloniki* 18 (3): 3–10.

Psaras, George K., and Theophanidis Constantinidis. 2009. 'Two New Nickel Hyperaccumulators from the Greek Serpentine Flora'. *Fresenius Environmental Bulletin* 18 (5): 798–803.

Shuka, Lulëzim, and Kit Tan. 2013. 'New Records for Albania Based on Taxa from the Prespa National Park'. *Biodiversity Data Journal*, no. 1 (December): e1014. https://doi.org/10.3897/BDJ.1.e1014.

Strid, Arne, and Kit Tan. 2009. 'A New Species of Centaurea (Asteraceae) from the Island of Samothraki (NE Greece)'. *Phytologia Balcanica* 15 (2): 185–89.

Tan, Kit, George Sfikas, et Gert Vold. 1997. « Viola parnonia (Violaceae), a new species from southern Greece ». *Annales Botanici Fennici* 34 (3): 149-52.

Tiniakou, A. 1991. 'Viola Dirphya (Violaceae), a New Species from Evvia Island, Greece'. *Viola Dirphya (Violaceae), a New Species from Evvia Island, Greece* (Genève) 46 (1): 119–24.

Trigas, Panayiotis, and Gregoris Iatrou. 2006. 'The Local Endemic Flora of Evvia (W Aegean, Greece)'. *Willdenowia* 36 (1): 257–70. https://doi.org/10.3372/wi.36.36121.

Varsamis, Georgios, Theodora Merou, Katerina Tseniklidou, Katerina Goula, and Spyros Tsiftsis. 2023. 'How Different Reproduction Protocols Can Affect the Germination of Seeds: The Case of Three Stenoendemic Species on Mt. Olympus (NC Greece)'. *EUROPEAN JOURNAL OF ENVIRONMENTAL SCIENCES* 13 (1): 23–30.

Viscaceae

Viscum

Böhling, Niels, Werner Greuter, Thomas Raus, Britt Snogerup, and Sven Snogerup. 2002. 'Notes on the Cretan Mistletoe, Viscum Album Subsp. Creticum Subsp. Nova (Loranthaceae/Viscaceae)'. *Israel Journal of Plant Sciences* 50 (sup1): 77–84. https://doi.org/10.1560/RRJ4-HU15-8BFM-WAUK.
