# Supplementary material for: The First Functional Traits Dataset for the Endemic Flora of Greece: morphology, ecology and ecosystem services
Source: Biodivers Data J. 2026 Feb 10;14:e180342. doi: 10.3897/BDJ.14.e180342 (PMC13291638; doi:10.3897/BDJ.14.e180342)
Supplement: Supplementary material 3 — Bibliography Traits & Ecology [file bdj-14-e180342-s003.pdf]

The First Functional Traits Dataset for the Endemic Flora of Greece: morphology, ecology and ecosystem services

Article type: Data Paper (Biodiversity)

Authors: Alexian Cheminal[1]*, Elpida Karadimou[1], Elisa Aubourg[2], Ioannis P. Kokkoris[3], Athanasios Kallimanis[4], Panayotis Dimopoulos[1]

[1] Laboratory of Botany, Department of Biology, University of Patras, 26504 Patras, Greece

[2] L'Institut Agro Dijon (ex-Agrosup Dijon), University of Burgundy, 21000 Dijon, France

[3] Department of Sustainable Agriculture, University of Patras, 2 G. Seferi St., 30131 Agrinio, Greece

[4] School of Biology, Aristotle University of Thessaloniki, 54124 Thessaloniki, Greece

Corresponding author: Alexian Cheminal (alexian.cheminal@upatras.gr)

Supplementary material 3: Functional traits of the Greek endemic taxa – Resources on the impact of functional traits on plants' metabolism and ecology

General

Erben, M. 1985. "Cytotaxonomische Untersuchungen an Süd-Osteuropäischen Viola-Arten Der Sektion Melanium." *Mitteilungen Der Botanischen Staatssammlung München* v.21 (1985):339–740.

Herbier Boissier., and Herbier Boissier. 1901a. *Bulletin de l'Herbier Boissier*. Vol. ser.2 v.2 1901-1902. Genève: Impr. Romet. https://www.biodiversitylibrary.org/item/105265.

———. 1901b. *Bulletin de l'Herbier Boissier*. Vol. ser.2 v.2 1901-1902. Genève: Impr. Romet. https://www.biodiversitylibrary.org/item/105265.

Merxmüller, H., and W. Lippert. 1977. "Veilchenstudien V-VII." *Mitteilungen Der Botanischen Staatssammlung München* 13:503–35.

Tiniakou, A. 1991. "Viola Dirphya (Violaceae), a New Species from Evvia Island, Greece." *CANDOLLEA* 46 (1): 119.

Trigas, Panayiotis, and Gregoris Iatrou. 2006. "The Local Endemic Flora of Evvia (W Aegean, Greece)." *Willdenowia*, 257–70.

Plant Height (H)

Díaz, Sandra, Jens Kattge, Johannes H. C. Cornelissen, et al. 2022. 'The Global Spectrum of Plant Form and Function: Enhanced Species-Level Trait Dataset'. *Scientific Data* 9 (1): 755. https://doi.org/10.1038/s41597-022-01774-9.

He, Dong, En-Rong Yan, Li-Ting Zheng, et al. 2025. 'Importance of the Actual Plant Height in Modulating the Within-Community Spectrum of Plant Form and Function'. *Frontiers in Plant Science* 16 (July). https://doi.org/10.3389/fpls.2025.1616656.

Jin, Dongmei, Xuecui Cao, and Keping Ma. 2014. 'Leaf Functional Traits Vary with the Adult Height of Plant Species in Forest Communities'. *Journal of Plant Ecology* 7 (1): 68–76. https://doi.org/10.1093/jpe/rtt023.

Ottaviani, Gianluigi, Timothy Harris, Mathieu Millan, Adam Klimeš, James L. Tsakalos, and Jiři Doležal. 2025. 'Size Isn't Age: Decoupled and Interacting Effects of Height and Age on Functional Traits in Grassland Plants'. *Journal of Ecology* 113 (9): 2712–25. https://doi.org/10.1111/1365-2745.70127.

Leaf Length (LL) and Leaf Width (LW)

Cao, Chenchen, Shufen Cui, Xinyu Guan, et al. 2024. 'Plant Leaf Functional Adaptions along Urban–Rural Gradients of Jinhua City'. *Plants* 13 (12): 1586. https://doi.org/10.3390/plants13121586.

Kanta, Chandra, Abhishek Kumar, Anjali Chauhan, Hukum Singh, and Ishwar Prakash Sharma. 2024. 'The Interplay Between Plant Functional Traits and Climate Change'. In *Plant Functional Traits for Improving Productivity*, edited by Narendra Kumar and Hukum Singh. Springer Nature. https://doi.org/10.1007/978-981-97-1510-7_3.

Khan, Ariful, Md Rezaul Karim, Mohammed A. S. Arfin-Khan, Md. Shamim Reza Saimun, Fahmida Sultana, and Sharif A. Mukul. 2025. 'How Do Leaf Functional Traits Influence Above-Ground Tree Carbon in Tropical Hill Forests of Bangladesh?' *Ecological Indicators* 171 (February): 113131. https://doi.org/10.1016/j.ecolind.2025.113131.

Xing, Yuting, Shiqin Deng, Yuanyin Bai, Zhengjie Wu, and Jian Luo. 2024. 'Leaf Functional Traits and Their Influencing Factors in Six Typical Vegetation

Communities'. *Plants* 13 (17): 2423. https://doi.org/10.3390/plants13172423.

Life Form (LF)

Dalke, I. V., A. B. Novakovskiy, S. P. Maslova, and Y. A. Dubrovskiy. 2018. 'Morphological and Functional Traits of Herbaceous Plants with Different Functional Types in the European Northeast'. *Plant Ecology* 219 (11): 1295–305. https://doi.org/10.1007/s11258-018-0879-2.
Díaz, Sandra, Andy Purvis, Johannes H. C. Cornelissen, et al. 2013. 'Functional Traits, the Phylogeny of Function, and Ecosystem Service Vulnerability'. *Ecology and Evolution* 3 (9): 2958–75. https://doi.org/10.1002/ece3.601.
Li, Jiaze, and Iain Colin Prentice. 2024. 'Global Patterns of Plant Functional Traits and Their Relationships to Climate'. *Communications Biology* 7 (1): 1136. https://doi.org/10.1038/s42003-024-06777-3.

Flowering Period (FP)

Craine, Joseph M., Elizabeth M. Wolkovich, E. Gene Towne, and Steven W. Kembel. 2012. 'Flowering Phenology as a Functional Trait in a Tallgrass Prairie'. *New Phytologist* 193 (3): 673–82. https://doi.org/10.1111/j.1469-8137.2011.03953.x.
Pareja-Bonilla, Daniel, Pedro Luis Ortiz, Leonor Patrícia Cerdeira Morellato, and Montserrat Arista. 2025. 'Functional Traits Predict Changes in Floral Phenology under Climate Change in a Highly Diverse Mediterranean Community'. *Functional Ecology*, 1–16. https://doi.org/10.1111/1365-2435.70062.
Wang, Yan, Xiao-Dong Yang, Arshad Ali, et al. 2020. 'Flowering Phenology Shifts in Response to Functional Traits, Growth Form, and Phylogeny of Woody Species in a Desert Area'. *Frontiers in Plant Science* 11 (May). https://doi.org/10.3389/fpls.2020.00536.

Flower Size (FS)

Cappellari, Andree, Giovanna Bonaldi, Maurizio Mei, Dino Paniccia, Pierfilippo Cerretti, and Lorenzo Marini. 2022. 'Functional Traits of Plants and Pollinators Explain Resource Overlap between Honeybees and Wild Pollinators'. *Oecologia* 198 (4): 1019–29. https://doi.org/10.1007/s00442-022-05151-6.
Pareja-Bonilla, Daniel, Pedro Luis Ortiz, Leonor Patrícia Cerdeira Morellato, and Montserrat Arista. 2025. 'Functional Traits Predict Changes in Floral Phenology under Climate Change in a Highly Diverse Mediterranean Community'. *Functional Ecology*, 1–16. https://doi.org/10.1111/1365-2435.70062.
Wang, Yan, Xiao-Dong Yang, Arshad Ali, et al. 2020. 'Flowering Phenology Shifts in Response to Functional Traits, Growth Form, and Phylogeny of Woody Species in a Desert Area'. *Frontiers in Plant Science* 11 (May). https://doi.org/10.3389/fpls.2020.00536.

Longevity (L)

Adler, Peter B., Roberto Salguero-Gómez, Aldo Compagnoni, et al. 2014. 'Functional Traits Explain Variation in Plant Life History Strategies'. *Proceedings of the National Academy of Sciences* 111 (2): 740–45. https://doi.org/10.1073/pnas.1315179111.

Reproduction strategy (RS)

Brunialti, Giorgio, Paolo Giordani, Sonia Ravera, and Luisa Frati. 2021. 'The Reproductive Strategy as an Important Trait for the Distribution of Lower-Trunk Epiphytic Lichens in Old-Growth vs. Non-Old Growth Forests'. *Forests* 12 (1): 27. https://doi.org/10.3390/f12010027.

Habitat (Ha)

Díaz, Sandra, Andy Purvis, Johannes H. C. Cornelissen, et al. 2013. "Functional Traits, the Phylogeny of Function, and Ecosystem Service Vulnerability." *Ecology and Evolution* 3 (9): 2958–75. https://doi.org/10.1002/ece3.601.

Altitude (A)

Bresson, Caroline C., Yann Vitasse, Antoine Kremer, and Sylvain Delzon. 2011. 'To What Extent Is Altitudinal Variation of Functional Traits Driven by Genetic Adaptation in European Oak and Beech?' *Tree Physiology* 31 (11): 1164–74. https://doi.org/10.1093/treephys/tpr084.
Di Biase, Letizia, Simone Fattorini, Maurizio Cutini, and Alessandro Bricca. 2021. 'The Role of Inter- and Intraspecific Variations in Grassland Plant Functional Traits along an Elevational Gradient in a Mediterranean Mountain Area'. *Plants* 10 (2): 359. https://doi.org/10.3390/plants10020359.
Xiang, Xiang, Huang Yong-Mei, Yang Chong-Yao, et al. 2021. 'Effect of Altitude on Community-Level Plant Functional Traits in the Qinghai Lake Basin, China'. *Chinese Journal of Plant Ecology* 45 (5): 456. https://doi.org/10.17521/cjpe.2020.0140.
Zhou, Fangfang, Cory Matthew, Pengfei Yang, Yafeng Huang, Bin Nie, and Zhibiao Nan. 2023. 'Leaf Morphology, Functional Trait and Altitude Response in Perennial Vetch (Vicia Unijuga A. Braun), Alfalfa (Medicago Sativa L.) and Sainfoin (Onobrychis Viciifolia Scop.)'. *Planta* 257 (4): 75. https://doi.org/10.1007/s00425-023-04098-z.

Chromosome number (2n)

Carta, Angelino, Gianni Bedini, and Lorenzo Peruzzi. 2018. 'Unscrambling Phylogenetic Effects and Ecological Determinants of Chromosome Number in Major Angiosperm Clades'. *Scientific Reports* 8 (1): 14258. https://doi.org/10.1038/s41598-018-32515-x.
Granse, Dirk, Paul Wendt, Sigrid Suchrow, et al. 2025. 'When Genetic Diversity Is Low: The Effects of Ploidy Level on Plant Functional Trait Expression in Spartina Under Global Change'. *Ecology and Evolution* 15 (3): e71022. https://doi.org/10.1002/ece3.71022.
Wei, Na, Richard Cronn, Aaron Liston, and Tia-Lynn Ashman. 2019. 'Functional Trait Divergence and Trait Plasticity Confer Polyploid Advantage in Heterogeneous Environments'. *New Phytologist* 221 (4): 2286–97. https://doi.org/10.1111/nph.15508.
